# Selective Acetalization in Pyridine: A Sustainable 5′‑*O*‑(2-Methoxypropyl) Protecting Group in the Synthesis of Nucleic Acid Analogs

**DOI:** 10.1021/acs.orglett.5c02400

**Published:** 2025-07-20

**Authors:** Verneri Saari, Aino Eerola, Mikko Ora, Alejandro Gimenez Molina, Andras Horvath, Yogesh S. Sanghvi, Pasi Virta

**Affiliations:** † Department of Chemistry, 8058 University of Turku, 20500 Turku, Finland; ‡ 50148 Janssen Pharmaceutica N.V., 30 Turnhoutseweg, B-2340 Beerse, Belgium; § Rasayan Inc., 2802 Crystal Ridge Road, Encinitas, California 92024-6615, United States

## Abstract

A mixture of 2-methoxypropene and an acid catalyst in pyridine results in an efficient 5′-*O*-(methoxyisopropyl) (MIP) acetalization of nucleosides, including 2′-deoxy, 2′-OH, 2′-*O*-methyl, 2′-*O*-methoxyethyl (MOE) and 2′-F-variants, in 44–77% isolated yields. For the reaction mechanism, we propose a pyridinium 2-methoxyprop-2-yl preassociation complex, which improves regioselectivity for the primary (5′-OH) over secondary (2′-OH and 3′-OH) hydroxy groups. The developed protocol makes the 5′-*O*-MIP-acetal an attractive protecting group for the sustainable synthesis of nucleosides and oligonucleotides in solution.

## Graphical abstract

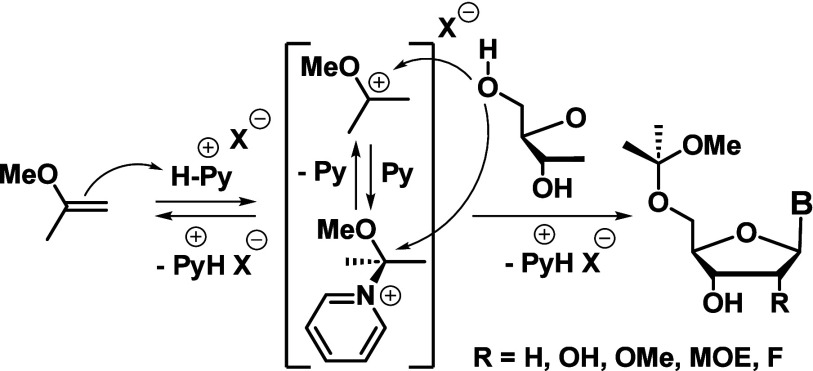

Acetals are important acid-labile protecting groups in nucleic acid chemistry. For example, 2′,3′-*O*,*O*-isopropylidene and 2′-*O*-tetrahydropyranyl (THP) protected ribonucleosides are common key intermediates for various nucleic acid products.^1,2^ Recently, acetone acetals (2-methoxyisopropyl = MIP and 2-isopropoxyprop-2-yl = IIP) have received interest as alternative protecting groups^3^ for the 5′-*O*-(4,4′-dimethoxytrityl) (DMTr) group in liquid-phase oligonucleotide synthesis (LPOS).^4–8^ The facile irreversible acid-catalyzed removal of the 5′-*O*-(2-alkoxypropyl) group and the released volatile byproducts acetone and methanol or isopropanol can reduce depurination and facilitate purification and isolation of growing oligonucleotide products in LPOS-compatible workups: extraction,^9–11^ precipitation,^12–16^ and organic solvent nanofiltration (OSN).^17–19^ This effort has culminated in sustainable oligonucleotide synthesis.^20^

The current choice of preparation of 5′-*O*-(2-alkoxyprop-2-yl) protected nucleoside building blocks consists of, however, a multistep synthesis via 3′-*O*-silyl protected intermediates,^4–8^ which is both time and reagent consuming. Careful stoichiometric control of reagents can increase regioselectivity of the acetalization,^21^ but the direct acid-catalyzed reaction between nucleosides and 2-alkoxypropene or 2,2-dialkoxypropane often result in a complex mixture of products, including 5′-*O*-, 3′-*O*-, and bis-3′,5′-*O*-(2-alkoxyprop-2-yl) protected nucleosides and cyclic acetal-bridged dinucleosides (Scheme 1). To overcome this challenge, herein, we present an efficient and regioselective acetalization in pyridine expanding opportunities for the acetal-based protecting group manipulation of nucleosides and carbohydrates^22^ and offer 5′-*O*-MIP-protected nucleosides as ideal building blocks for LPOS.^23–27^

**Scheme 1:**
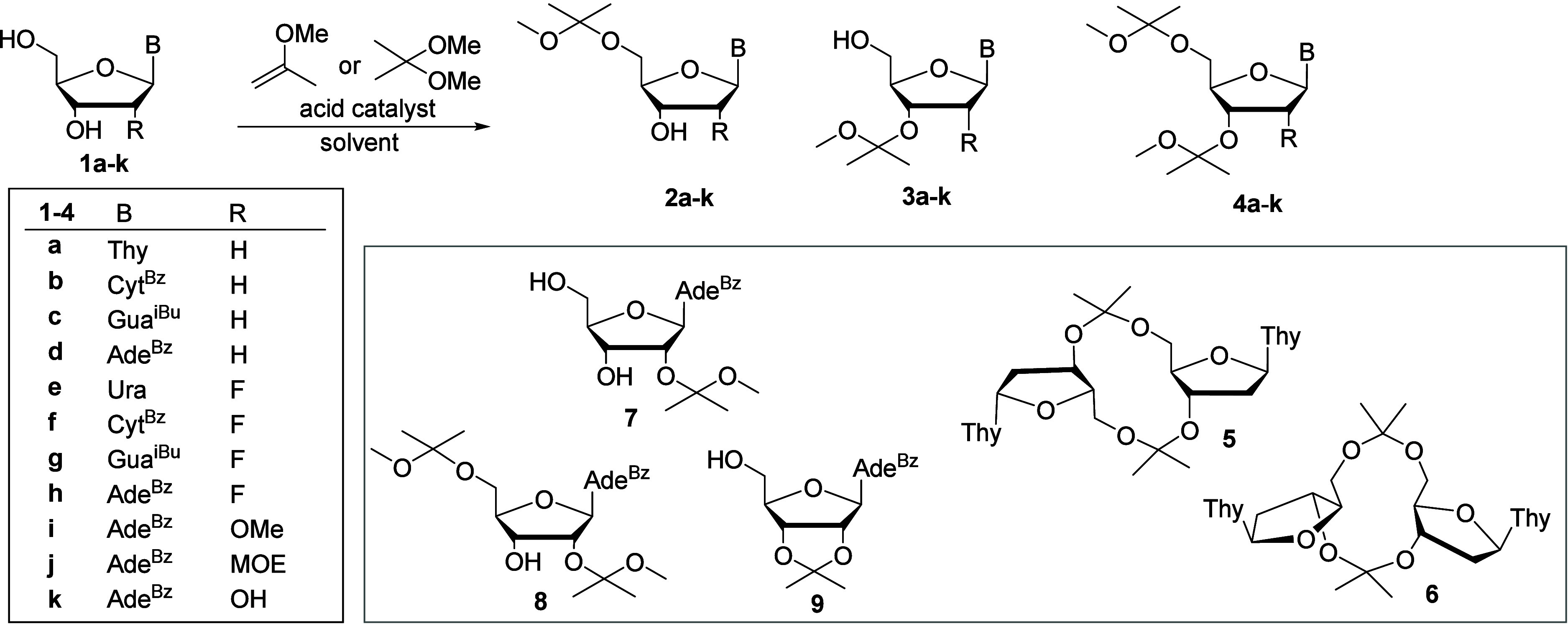
Acetalization of Nucleosides with 2-Methoxypropene or 2,2-Dimethoxypropane

We hypothesized that the required regioselectivity between the primary (5′-OH) and secondary (3′-OH) hydroxy groups in a nucleoside (**1**–**4**) could be achieved if the acetalization does not follow the standard acid-catalyzed mechanism. In the electrophilic addition between an alcohol and 2-methoxypropene, the first step of the reaction is protonation of the double bond, resulting in a stable oxocarbenium cation. Therefore, carbocation reacts with an alcohol that yields the acetal product, or it can react with a temporary nucleophile,^28^ which then undergoes a leaving group-dependent SN1-replacement with an alcohol. By exposing 2-methoxypropene to an acid catalyst in the presence of pyridine (in the absence of alcohol), we could observe a 2-methoxy-prop-2-ylpyridinium (MIPPY) preassociation complex by NMR (^1^H–^15^N-HMBC spectrum shown in Figure 1, cf. additional NMR data in Figures S90–S109). The MIPPY complex could act as a potential intermediate of the acetalization and affect the regioselectivity. A similar preassociation complex has been suggested to contribute to selective removal of aldehyde acetals using triethylsilyltriflate-base combinations.^29^

**Figure 1:**
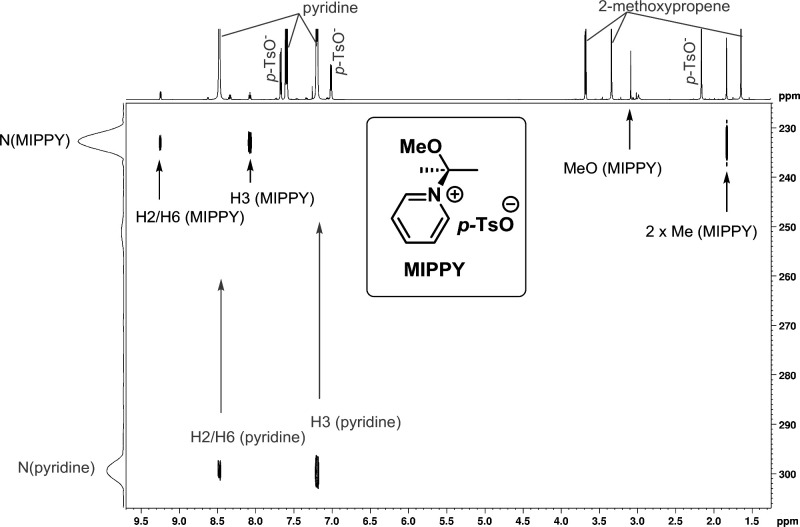
^1^H–^15^N-HMBC spectrum of the 2-methoxy-prop-2-ylpyridinium preassociation complex. Conditions: 2-methoxypropene, 0.31 mol L^–1^; pyridine (4 equiv), pyH^+^ *p*TsO^–^ (0.5 equiv), TMSCl (0.25 equiv. to remove residual water) in CDCl_3_. Measured at 258 K.

The observations that protonation of 2-methoxypropene could occur under slightly basic conditions with the formation of a preassociation complex encouraged us to study reaction parameters of the acetalization in different solvent/nucleophile environments in greater detail (Table 1). Thymidine (T, **1a**) was selected as a preliminary model diol. Reactions were performed in a 0.12 mmol scale (0.28 or 0.55 mol L^–1^ of **1a**) and monitored over 24 h by reversed phase HPLC (Figure 2). The reaction with 2-methoxypropene (2 equiv) in DMF in the presence of *p*-TsOH (0.1 equiv) at 25 °C (entry 1) led to a dynamic multicomponent mixture, consisting of cyclic isopropylidene-bridged dithymidines (**5** and **6**, existing as major products after 2 h, Figures S2 and S59–S62). Acetalization with 2-methoxypropene (1–4 equiv. 0.55–1.1 mol L^–1^) in the presence of *p*-TsOH (0.1 equiv) in pyridine at 25 °C, in turn, resulted in a good regioselectivity between the primary (5′-OH) and secondary (3′-OH) hydroxy groups (**2a**:**3a**, ranging from 6:1 to 19:1, *n*/*n*) (entries 2–4). Formation of 5′,3′-*O*,*O*-bis-MIP-T (**4a**) but not **5** and **6** was also observed in each case. Decreasing temperature from 25 to 4 °C led to a slow reaction, but no marked improvement in selectivity was found (entry 5, a higher excess and concentration of 2-methoxypropene used). No marked difference in yields and regioselectivity of the acetalization was found when the *p*-TsOH·H_2_O catalyst was replaced by pyridinium chloride, pyridinium tosylate, 2,6-lutidinium tosylate, or 2,4,6-collidinium tosylate (0.1 equiv each) (entries 7–9). Thus, the conjugate acid (HCl vs *p*-TsOH) or hydrate of *p*-TsOH did not interfere with the reaction. Polymer-bound pyridinium tosylate was also tested in various concentrations, but yields of 5′-*O*-acetalization remained modest (entry 10). In addition to pyridinium salts, tetrazole (0.1 equiv) as an acid catalyst (p*K*_a_ 4.9) was also examined. No product was observed in pyridine or in DMF (entries 11 and 12). DMAP (0.05 equiv) and *p*-methoxypyridine (0.1 equiv) as potential nucleophilic catalysts in pyridine did not affect the reaction (entries 13 and 14). Interestingly, reaction in 2,6-lutidine or in 2,4,6-collidine in the presence of their *p*-TsOH-salts (0.1 equiv) did not yield products (entries 15 and 16). This may be related to different p*K*_a_ values of the pyridine derivatives (2,4,6-collidine: 7.4; 2,6-lutidine: 6.7; pyridine: 5.3) but may also indicate that pyridine had a specific nucleophilic role in the reaction, referring to the hypothesized preassociation complex (similar complexation could not be observed with 2,6-lutidine or 2,4,6-collidine in corresponding NMR experiments, Figures S110–S119). The acid-catalyzed (*p*-TsOH·H_2_O, 0.1 equiv) acetalization with 2-methoxypropene was performed also in DMF in the presence of pyridine, *p*-methoxypyridine (p*K*_a_ 6.5), 2,6-lutidine, and 2,4,6-collidine (2 equiv of each pyridine derivative). In the presence of pyridine, 5′-*O*-MIP-T (**2a**) was obtained in 28% yield, but no product was observed with other pyridine derivatives (entries 17–19).

**Figure 2:**
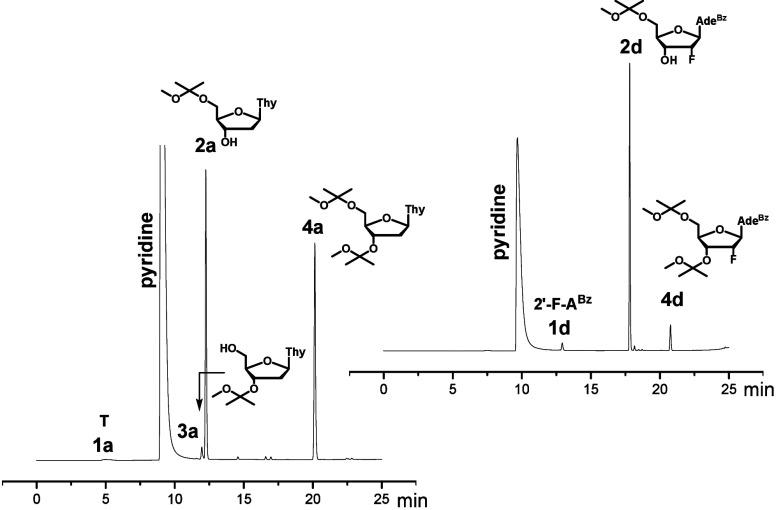
Examples of RP HPLC profiles of the acetalization. Reaction conditions: cf. entries 3 and 26 in Table 1.

**Table 1:** Molar Ratios of the Products and Starting Materials under Variable Acetalization Conditions Monitored by RP-HPLC^a^

	reaction conditions				molar ratios of compounds/%				
entry	**1** (conc./mol L^–1^)	2-methoxypropene (conc./mol L^–1^)	acid catalyst (0.1 equiv) (+ additive)	solvent	**1**	**2**	**3**	**4**	**2**/**3**, *n*/*n*
1*	**a** (0.55)	2 eq (1.1)	*p*-TsOH·H_2_O	DMF	45	15	24	3	2:1
2	**a** (0.55)	1 eq (0.55)	*p*-TsOH·H_2_O	Py	40	48	8	4	6:1
3	**a** (0.55)	2 eq (1.1)	*p*-TsOH·H_2_O	Py	1	51	3	45	17:1
4	**a** (0.28)	4 eq (1.1)	*p*-TsOH·H_2_O	Py	1	56	3	40	19:1
5**	**a** (0.28)	8 eq (2.2)	*p*-TsOH·H_2_O	Py	38	8	50	4	6:1
6	**a** (0.55)	2 eq (1.1)	PyH^+^Cl^–^	Py	5	60	6	29	10:1
7	**a** (0.55)	2 eq (1.1)	PyH^+^ *p*-TsO^–^	Py	10	64	8	18	8:1
8	**a** (0.55)	2 eq (1.1)	LutH^+^ *p*-TsO^–^	Py	4	63	6	27	11:1
9	**a** (0.55)	2 eq (1.1)	CollH^+^ *p*-TsO^–^	Py	11	65	8	16	8:1
10	**a** (0.55)	2 eq (1.1)	PyH^+^ *p*-TsO^–^(PS)	Py	84	13	3	0	4:1
11	**a** (0.55)	2 eq (1.1)	Tetrazole	Py	100	0	0	0	n.a.
12	**a** (0.55)	2 eq (1.1)	Tetrazole	DMF	100	0	0	0	n.a.
13	**a** (0.55)	2 eq (1.1)	*p*-TsOH·H_2_O + 0.05 eq DMAP	Py	11	65	8	16	8:1
14	**a** (0.55)	2 eq (1.1)	*p*-TsOH·H_2_O + 0.1 eq 4-MeOPy	Py	8	65	7	20	9:1
15	**a** (0.55)	2 eq (1.1)	LutH^+^TsO^–^	Lut	100	0	0	0	n/a
16	**a** (0.55)	2 eq (1.1)	CollH^+^TsO^–^	Coll	100	0	0	0	n.a.
17	**a** (0.55)	2 eq (1.1)	*p*-TsOH·H_2_O + 2 eq Py	DMF	66	28	5	1	6:1
18	**a** (0.55)	2 eq (1.1)	*p*-TsOH·H_2_O + 2 eq Lut	DMF	99	1	0	0	n.a.
19	**a** (0.55)	2 eq (1.1)	*p*-TsOH·H_2_O + 2 eq Coll	DMF	100	0	0	0	n.a.
20	**b** (0.28)	4 eq (1.1)	*p*-TsOH·H_2_O	Py	6	64	7	23	9:1
21	**c** (0.55)	2 eq (1.1)	*p*-TsOH·H_2_O	Py	13	75	6	6	13:1
22	**d** (0.28)	4 eq (1.1)	*p*-TsOH·H_2_O	Py	1	53	2	44	27:1
23	**e** (0.55)	2 eq (1.1)	*p*-TsOH·H_2_O	Py	5	83	3	9	28:1
24	**f** (0.55)	2 eq (1.1)	*p*-TsOH·H_2_O	Py	2	79	3	16	26:1
25	**g** (0.28)	4 eq (1.1)	*p*-TsOH·H_2_O	Py	13	82	3	2	27:1
26	**h** (0.55)	2 eq (1.1)	*p*-TsOH·H_2_O	Py	0	90	0	10	1:0
27	**i** (0.55)	2 eq (1.1)	*p*-TsOH·H_2_O	Py	0	83	0	17	1:0
28	**j** (0.55)	2 eq (1.1)	*p*-TsOH·H_2_O	Py	0	85	3	12	28:1
29	**k** (0.55)	2 eq (1.1)	*p*-TsOH·H_2_O	Py	3	62	4***	31****	n.a.

a In the reaction in entry 1, (*) cyclic isopropylidene-bridged dinucleosides **5** (12%) and **6** (1%) have been observed as byproducts. The reactions are performed at 25 °C, but for (**), the reaction in entry 5 is performed at 4 °C. In the reaction in entry 29, (***) sum (4%) of 2′-*O*-MIP- and 3′-*O*-MIP-A^Bz^ reported; (****) sum (31%) of 3′,5′-*O*,*O*-MIP- and 2′,5′-*O*,*O*-MIP-A^Bz^ reported. Py = pyridine, Lut = 2,6-lutidine, Coll = 2,4,6-collidine, 4-MeOPy = 4-methoxypyridine, and *p*-TsO^–^(PS) = polystyrene-bound toluenesulfonic acid.

Next, a more detailed NMR analysis of the acetalization was carried out (Figure 3). Thymidine was exposed to 2-methoxypropene (2 equiv) in the presence of *p*-TsOH·H_2_O (0.1 equiv) in pyridine-*d*_5_ at 25 °C in an NMR tube (0.55 mol L^–1^ of T). From the molar ratios of the products, relative formation rates of 85:15 for 5′-*O*-MIP-T (**2a**) and 3′-*O*-MIP-T (**3a**) were extracted from the beginning of the acetalization. The maximal accumulation of 5′-*O*-MIP-T (**2a**) was observed after 10 h (**2a**:**3a**, 12:1, *n*/*n*). Conversion of both 5′-*O*-MIP-T (**2a**) and 3′-*O*-MIP-T (**3a**) to 3′,5′-*O*,*O*-bis-MIP T (**4a**) was observed, when the acetalization progressed (cf. molar ratio of **2a**:**3a** during reaction in Figure S1). Hydrolysis of 2-methoxypropene to acetone and 2,2-dimethoxypropane can be seen in the reaction mixture.

**Figure 3:**
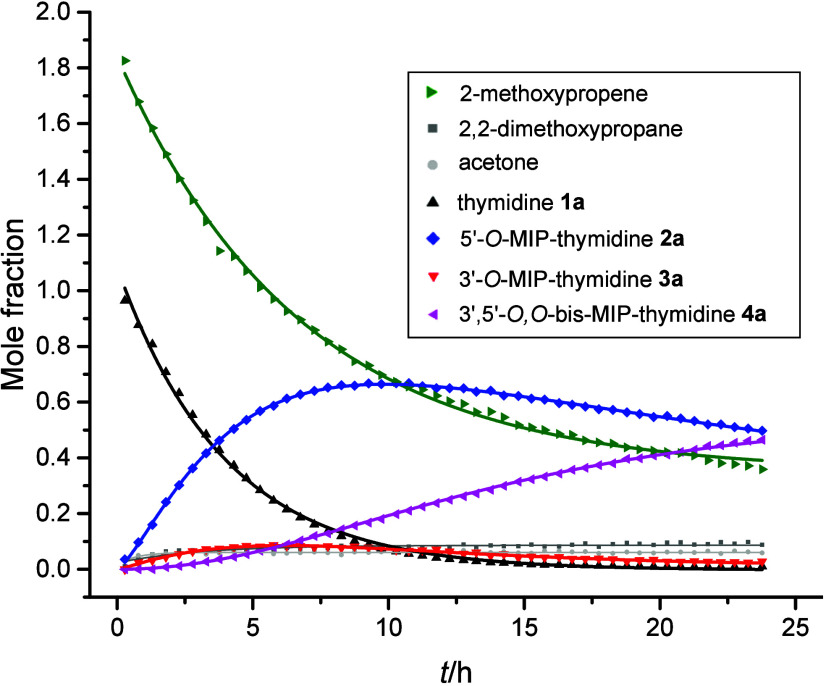
Time-dependent product distribution of acetalization of thymidine (0.55 mol L^–1^) using 2 equiv of 2-methoxypropene (1.1 mol L^–1^) and 0.1 equiv of *p*-TsOH·H_2_O in pyridine-*d*_5_ at 25 °C, monitored by ^1^H NMR.

The reversibility of the reaction and acidity of the catalyst can affect the regioselectivity of the acetalization. When 2-methoxypropene was replaced by 2,2-dimethoxypropane (1.1 mol L^–1^, 2 equiv) in pyridine in the presence of *p*-TsOH·H_2_O (0.1 equiv), a 15% conversion to 5′-*O*-MIP-T was observed. When isolated 5′-*O*-MIP-T (**2a**) was dissolved in pyridine in the presence of *p*-TsOH·H_2_O (0.1 equiv) and methanol, slow degradation (16% over 24 h) to thymidine (**1a**) was observed. These observations demonstrate that protonation of acetal oxygens can, in fact, occur under the given conditions. Hydrolysis products acetone and 2,2-dimethoxypropane can act as side reagents, affecting the regioselectivity.

The sugar puckering and potential electronic influence of the 2′-group of nucleosides can affect the product ratio.^30^ Acetalization of *N* ^4^-benzoyl 2′-deoxyadenosine (dA^Bz^, **1d**), *N* ^4^-benzoyl adenosine (A^Bz^, **1k**), and 2′-*O*-modified variants, viz., *N* ^4^-benzoyl-2′-deoxy-2′-F (2′-F-A^Bz^, **1h**), -2′-*O*-methyl (2′-OMe-A^Bz^, **1i**), and -2′-*O*-methoxyethyl adenosine (2′-MOE-A^Bz^, **1j**), was next examined due to their utility in therapeutically relevant oligonucleotides. For solubility reasons, a 0.28 mol L^–1^ initial concentration of dA^Bz^ (**1d**) was used, but otherwise, the reactions were performed in pyridine at 0.55 mol L^–1^ of the adenosine derivative and 1.1 mol L^–1^ 2-methoxypropene (4 equiv. compared to **1d** and 2 equiv. compared to **1h**–**k**) in the presence of *p*-TsOH·H_2_O (0.1 equiv) (entries 22 and 26–29). After 24 h at 25 °C, the yields of 5′-*O*-acetalization varied from 53% to 90%, being the lowest (53%) with dA^Bz^ (**2d**) (entry 22) and the highest (90%) with 2′-F-A^Bz^ (**2h**) (entry 26). The high regioselectivity with **1h** and **1i** (**2**:**3**, 1:0, *n*/*n*) was notable. The higher yield (83–90%) of 5′-*O*-MIP-2′-modified adenosines (**2h**–**j**) in comparison to that of 5′-*O*-MIP-dA^Bz^ (**2d**) may be attributed to the favored *N*-conformation of ribose, which increases the reactivity of the 5′-OH vs 3′-OH groups.^30^ Interestingly, 5′-*O*-MIP-A^Bz^ (**2k**) was obtained in 62% yield (entry 29). 3′-*O*-MIP (**3k**), 2′-*O*-MIP (**7**), and bis-MIP-A^Bz^ (**4k** and **8**), but not 2′,3′-*O*,*O*-isopropylidene A^Bz^ (**9**), were detected, despite the potential protonation of the methoxy oxygen of the 2′/3′-*O*-MIP group (**3k** and **7**) and subsequent nucleophilic attack of the neighboring OH group to the acetal carbon.

Acetalization of the other nucleobase variants of 2′-deoxyribonucleosides (dC^Bz^ and dG^iBu^) and 2′-deoxy-2′-F-ribonucleosides (2′-F-U, 2′-F-dC^Bz^, and 2′-F-dG^iBu^) was examined with the same conditions (entries 20, 21, 23–25). In general, higher yields were obtained for 5′-*O*-MIP-2′-deoxy-2′-F-ribonucleotides **2e**–**h** (79–90%) than for 5′-*O*-MIP-2′-deoxyribonucleotides **2a**–**d** (58–75%). The base protecting groups did not influence the outcome of the acetalization reactions studied.

After the small-scale studies, a gram-scale synthesis of various 5′-*O*-MIP-protected nucleosides was performed (Scheme 2). Accordingly, each of the aforementioned nucleosides were treated with 2-methoxypropene (2–4 equiv) in the presence of *p*-toluenesulfonic acid (0.1 equiv) in pyridine at 25 °C (**S12**–**S44**). The 5′-*O*-MIP products (**2a**–**k**), as well as 3′-*O*-MIP and 3′,5′-*O*,*O*-bis-MIP side products, were isolated and characterized by ^1^H and ^13^C NMR (Figures S17–S66) and HRMS spectroscopy. 5′-*O*-MIP-dA^Bz^, 5′-*O*-MIP-dG^iBu^, and 5′-*O*-MIP protected 2′-modified nucleosides **6c**–**k** were purified by silica gel column chromatography in 64–77% yields (Scheme 2). 5′-*O*-MIP-T (**2a**) and 5′-*O*-MIP-dC^Bz^ (**2b**) were purified by precipitation in a mixture of EtOAc and hexane (1:4, *v*/*v*) in 44% and 47% yields, respectively. When precipitation was used for purification, partial overacetalization of nucleosides to 3′,5′-*O*,*O*-bis-MIP-products (**4a** and **4b**) was preferred to consume the starting material (**1a** and **1b**); otherwise, they were precipitated with the desired 5′-*O*-MIP product (**2a** and **2b**) (Figure S3). The 5′-*O*-MIP protected nucleosides were also phosphorylated via a conventional protocol to give the corresponding 3′-*O*-phosphoramidites, useful building blocks for LPOS,^4–8^ in 71–93% yields (Scheme 2, Figures S45–SS60).

**Scheme 2:**
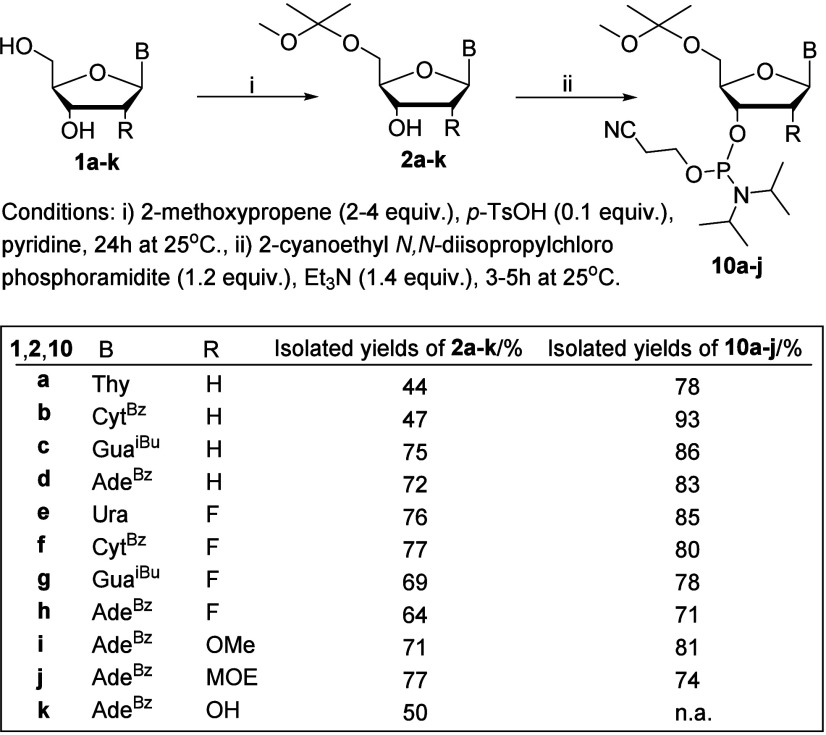
Synthesis of 5′-*O*-MIP-Protected Phosphoramidite Building Blocks of Nucleosides

In conclusion, for the first time, a regioselective 5′-*O*-(2-methoxyprop-2-yl) (MIP) acetalization of nucleosides using 2-methoxypropene in pyridine in the presence of an acid catalyst is described. Pyridine (but not its methylated analogs 2,6-lutidine and 2,4,6-collidine) was observed to have an important role in the reaction, presumably via formation of the pyridinium 2-methoxyprop-2-yl preassociation complex, which improves regioselectivity of the acetalization reaction. This facile protection protocol is expected to allow large-scale production of 5′-*O*-MIP-protected nucleosides useful for liquid-phase oligonucleotide synthesis (LPOS). The demonstrated ability of regioselective 5′-*O*-MIP protection, combined with its traceless removal, resulting in volatile byproducts acetone and methanol, may also open new opportunities for synthetic design of novel nucleosides and carbohydrates in a broader area of complex chemistry. Further understanding of the reaction mechanism, acetalization with different sugar configurations, and further scalability of the reaction are planned as next steps leading to a more sustainable manufacturing of therapeutic oligonucleotides.

## Supplementary Material



## Data Availability

The data underlying this study are available in the published article and its Supporting Information.

## Notes

Experimental details, HPLC chromatograms, and NMR spectra of the compounds presented in this article (PDF)
