# Supplementary material for: Selective Acetalization in Pyridine: A Sustainable 5′‑O‑(2-Methoxypropyl) Protecting Group in the Synthesis of Nucleic Acid Analogs
Source: Org Lett. 2025 Jul 20;27(30):8251–6. doi: 10.1021/acs.orglett.5c02400 (PMC12322960; doi:10.1021/acs.orglett.5c02400)
Supplement: Supplementary file 1 [file ol5c02400_si_001.pdf]

Selective Acetalization in Pyridine: A Sustainable 5′-O-(2-Methoxyisopropyl) Protecting Group in the Synthesis of Nucleic Acid Analogs

Verneri Saari[a], Aino Eerola[a], Mikko Ora[a], Alejandro Gimenez Molina[b], Andras Horvath[b], Yogesh Sanghvi[c], Pasi Virta*[a]

[a]Department of Chemistry, University of Turku, 20500 Turku, Finland

[b]Janssen Pharmaceutica N.V., 30 Turnhoutseweg, B-2340 Beerse, Belgium

[c] Rasayan Inc., 2802 Crystal Ridge Road, Encinitas, CA 92024-6615, U.S.A.

Contents

General methods S1

Milligram scale test acetalizations of different nucleosides S1

Monitoring acetalization of thymidine by ^{1}H NMR S1

Monitoring acetalization of thymidine in DMF by RP-HPLC S2

Precipitation of *N*^{4}-Benzoyl-5´-*O*-(2-methoxyprop-2-yl)-2´-deoxycytidine (**2b**) in the mixture of ethyl acetate and hexane S3

Stability of 5´-MIP-protected phosphoramidite building blocks in acetonitrile S3

Protection of exocyclic amino groups S3

General procedure for benzoylation of exocyclic amino groups S3

N^{4}-Benzoyl-2´-fluorocytidine (**1f**). S4

N^{2}-Isobutyryl-2´-fluoroguanosine (**1g**). S4

N^{6}-Benzoyl-2´-fluoroadenosine (**1h**). S4

N^{6}-Benzoyl-2´-*O*-methyladenosine (**1i**). S5

N^{6}-Benzoyl-2´-*O*-methoxyethyladenosine (**1j**). S5

NMR spectra of exocyclic amino group protected nucleosides S6

Acetalizations S12

General procedure for selective 5´-acetalization S12

5´-*O*-(2-methoxyprop-2-yl)-thymidine (**2a**). S12

3´-*O*-(2-methoxyprop-2-yl)-thymidine (**3a**) and 5´,3´-*O*-bis-(2-methoxyprop-2-yl)-thymidine (**4a**). S13

N^{4}-Benzoyl-5´-*O*-(2-methoxyprop-2-yl)-2´-deoxycytidine (**2b**). S13

N^{4}-Benzoyl-5´,3´-*O*-bis-(2-methoxyprop-2-yl)-2´-deoxycytidine (**4b**). S13

N^{2}-Isobutyryl-5´-*O*-(2-methoxyprop-2-yl)-2´-deoxyguanosine (**2c**). S14

N^{2}-Isobutyryl-5´,3´-*O*-bis-(2-methoxyprop-2-yl)-2´-deoxyguanosine (**4c**). S14

N^{6}-Benzoyl-5´-*O*-(2-methoxyprop-2-yl)-2´-deoxyadenosine (**2d**). S14

N^{6}-Benzoyl-5´,3´-*O*-bis-(2-methoxyprop-2-yl)-2´-deoxyadenosine (**4d**). S14

5´-*O*-(2-methoxyprop-2-yl)-2´-fluorouridine (**2e**). S15

5´,3´-*O*-bis-(2-methoxyprop-2-yl)-2´-fluorouridine (**4e**). S15

N^{4}-Benzoyl-5´-*O*-(2-methoxyprop-2-yl)-2´-fluorocytidine (**2f**). S15

N^{4}-Benzoyl-5´,3´-*O*-bis-(2-methoxyprop-2-yl)-2´-fluorocytidine (**4f**). S16

N^{2}-Isobutyryl-5´-(2-methoxyprop-2-yl)-2´-fluoroguanosine (**2g**). S16

N^{2}-Isobutyryl-5´,3´-*O*-bis-(2-methoxyprop-2-yl)-2´-fluoroguanosine (**4g**). S16

N^{6}-Benzoyl-5´-(2-methoxyprop-2-yl)-2´-fluoroadenosine (**2h**). S17

N^{6}-Benzoyl-5´,3´-*O*-bis-(2-methoxyprop-2-yl)-2´-fluoroadenosine (**4h**). S17

N^{6}-Benzoyl-5´-*O*-(2-methoxyprop-2-yl)-2´-*O*-methylyadenosine (**2i**). S17

N^4-Benzoyl-5´,3´-*O*-bis-(2-methoxyprop-2-yl)-2´-*O*-methyladenosine (**4i**)....S18
N^6-Benzoyl-5´-*O*-(2-methoxyprop-2-yl)-2´-*O*-methoxyethyladenosine (**2j**)....S18
N^6-Benzoyl-5´,3´-*O*-bis-(2-methoxyprop-2-yl)-2´-*O*-methoxyethyladenosine (**4j**). S18
5´,3´:3´,5´-Di-*O*-isopropylidine-dithymidine (**5**) and 5´,5´:3´,3´-Di-*O*-isopropylidine-dithymidine (**6**)....S19
N^6-Benzoyl-5´-*O*-(2-methoxyprop-2-yl)-adenosine (**2k**)....S19
Mixture of N^6-Benzoyl-5´,3´-*O*-bis-(2-methoxyprop-2-yl)-adenosine (**4k**) and N^6-Benzoyl-5´,2´-*O*-bis-(2-methoxyprop-2-yl)-adenosine (**8**)....S19
NMR spectra of acetalized nucleosides S20
Synthesis of phosphoramidite building blocks (**10a-j**) of 5'-*O*-MIP-protected nucleosides S46
General procedure....S46
5´-*O*-(2-methoxyprop-2-yl)-thymidine-3´-*O*-(2-cyanoethyl-*N*,*N*-diisopropyl)phosphoramidite (**10a**). S47
N^4-Benzoyl-5´-*O*-(2-methoxyprop-2-yl)-2´-deoxycytidine-3´-*O*-(2-cyanoethyl-*N*,*N*-diisopropyl)phosphoramidite (**10b**). S47
N^2-Isobutyryl-5´-*O*-(2-methoxyprop-2-yl)-2´-deoxyguanosine-3´-*O*-(2-cyanoethyl-*N*,*N*-diisopropyl)phosphoramidite (**10c**)....S47
N^6-Benzoyl-5´-*O*-(2-methoxyprop-2-yl)-2´-deoxyadenosine-3´-*O*-(2-cyanoethyl-*N*,*N*-diisopropyl)phosphoramidite (**10d**). S47
5´-*O*-(2-methoxyprop-2-yl)-2´-fluorouridine-3´-*O*-(2-cyanoethyl-*N*,*N*-diisopropyl)phosphoramidite (**10e**). S48
N^4-Benzoyl-5´-*O*-(2-methoxyprop-2-yl)-2´-fluorocytidine-3´-(2-cyanoethyl-*N*,*N*-diisopropyl)phosphoramidite (**10f**). S48
N^2-Isobutyryl-5´-(2-methoxyprop-2-yl)-2´-fluoroguanosine-3´-(2-cyanoethyl-*N*,*N*-diisopropyl)phosphoramidite (**10g**)....S48
N^6-Benzoyl-5´-*O*-(2-methoxyprop-2-yl)-2´-fluoroadenosine-3´-(2-cyanoethyl-*N*,*N*-diisopropyl)phosphoramidite (**10h**). S49
N^6-Benzoyl-5´-*O*-(2-methoxyprop-2-yl)-2´-*O*-methyladenosine-3´-(2-cyanoethyl-*N*,*N*-diisopropyl)phosphoramidite (**10i**). S49
N^6-Benzoyl-5´-*O*-(2-methoxyprop-2-yl)-2´-*O*-methoxyethyladenosine-3´-(2-cyanoethyl-*N*,*N*-diisopropyl)phosphoramidite (**10j**). S50
NMR spectra of 5´-*O*-MIP phosphoramidite building blocks S51
NMR studies of the 2-methoxy-prop-2-yl pyridium (MIPPY) pre-association complexes....S62
Purity documentation for known compounds **2a-d** & **10a-d**....S78
References....S82

General methods

^{1}H, ^{13}C, ^{15}N, ^{19}F and ^{31}P NMR spectra were recorded on Bruker Avance 500 MHz and 600 MHz instruments. Structural assignments were made with additional information from gCOSY, gHSQC, and gHMBC experiments. Mass spectra were recorded on Waters RDa or HESI-Q-Orbitrap. An analytical Thermo ODS Hypersil C18 (4.6 × 250 mm, 5 µm, flow rate 1 mL/min, detection at λ = 260 nm) and gradient elution A, B and C were used for the RP-HPLC analyses. Aqueous triethylammonium acetate (TEAA) buffer (50 mM) and acetonitrile with 50 mM TEAA were used as mobile phases. Gradient A: 10–55 % of ACN over 20 min. Gradient B: 10–27 % of ACN over 10 min and 27–100 % of ACN over 10 min. Gradient C: 10–17 % of ACN over 10 min and 17–100 % of ACN over 10 min. Pyridine, 2,4-lutidine, 2,4,6-collidine and DMF were dried with 4 Å molecular sieves. All used acid catalysts were dried in solution with 4 Å molecular sieves. TEA was dried over CaH_2.

Milligram scale test acetalizations of different nucleosides

Nucleoside (1.0 eq., 0.12 mmol) was dried with P_2O_5 overnight and dissolved in anhydrous pyridine, 2,6-lutidine, 2,4,6-collidine or DMF (1.88 or 3.75 L/mol of nucleoside). Acid catalyst (0.1 eq., 0.012 mmol) and 2-methoxypropene (1–8 eq., 0.12–0.96 mmol) were added and the mixture was shaken for 24 h. The reaction was monitored with RP-HPLC immediately and after 24 h by using gradient A.

Monitoring acetalization of thymidine by ^{1}H NMR

Thymidine (80 mg, 0.33 mmol) was dried under vacuum over P_2O_5 overnight, dissolved in anhydrous pyridine-*d*5 (535 µL) and 2-methoxypropene (66 µL, 0.66 mmol) was added. The reaction mixture was transferred to an NMR tube. Reaction was initiated by adding dried *p*TsOH·H_2O/pyridine-*d*5 solution (65 µL from 0.1 mg/mL, 0.03 mmol). Reaction was monitored by ^{1}H NMR with 30 min intervals for 24 h.

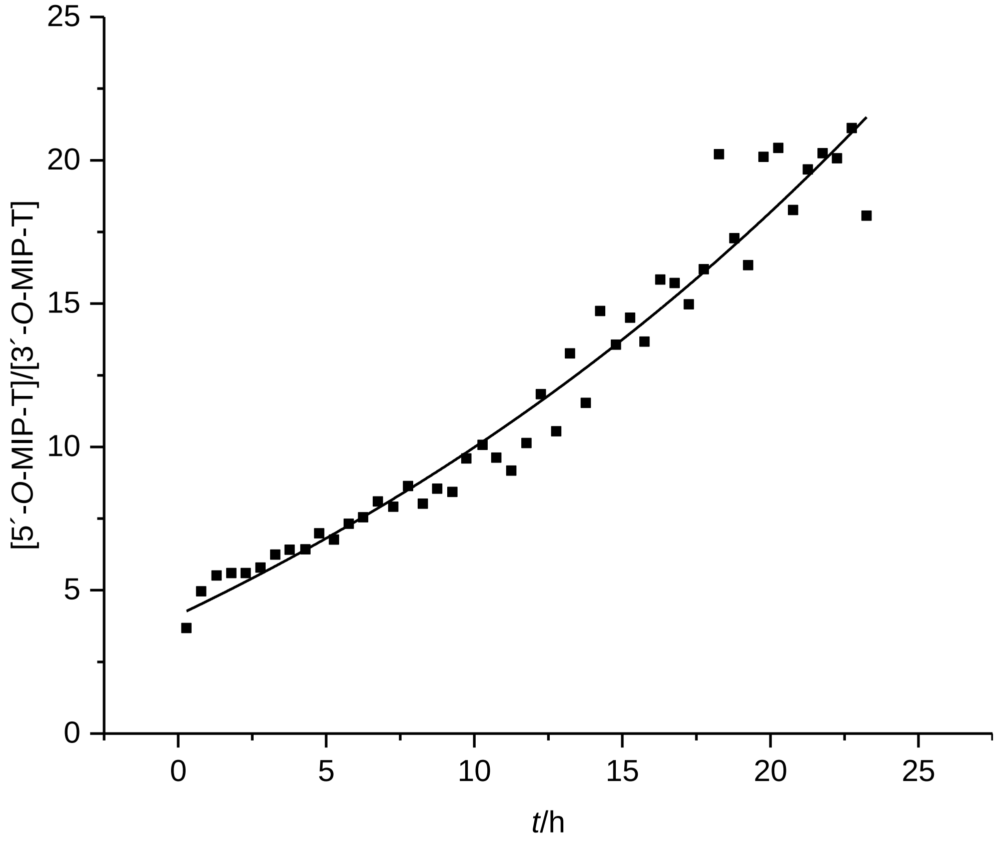

Figure S1. Ratio of 5´-*O*-MIP-T and 3´-*O*-MIP-T during the reaction. Relative amount of 5´-*O*-MIP-T increases during the reaction, because 3´-*O*-MIP-T reacts faster than 5´-*O*-MIP-T to 3,5'-*O,O*-bis-MIP-T product.

Monitoring acetalization of thymidine in DMF by RP-HPLC

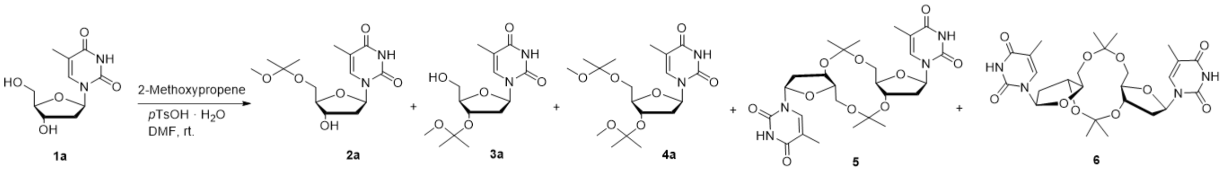

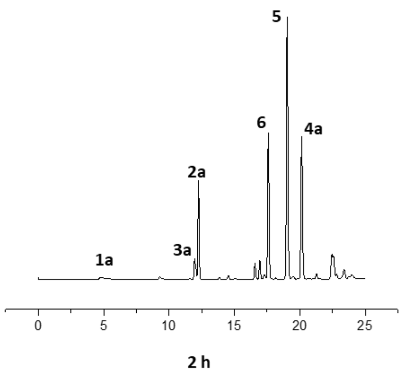

Figure S2. RP-HPLC profile for acetalization of thymidine after 2 h using 2-methoxypropene (2.0 eq.), *p*TsOH·H_2O (0.1 eq.) and DMF.

Precipitation of N^4-Benzoyl-5´-*O*-(2-methoxyprop-2-yl)-2´-deoxycytidine (2b) in the mixture of ethyl acetate and hexane

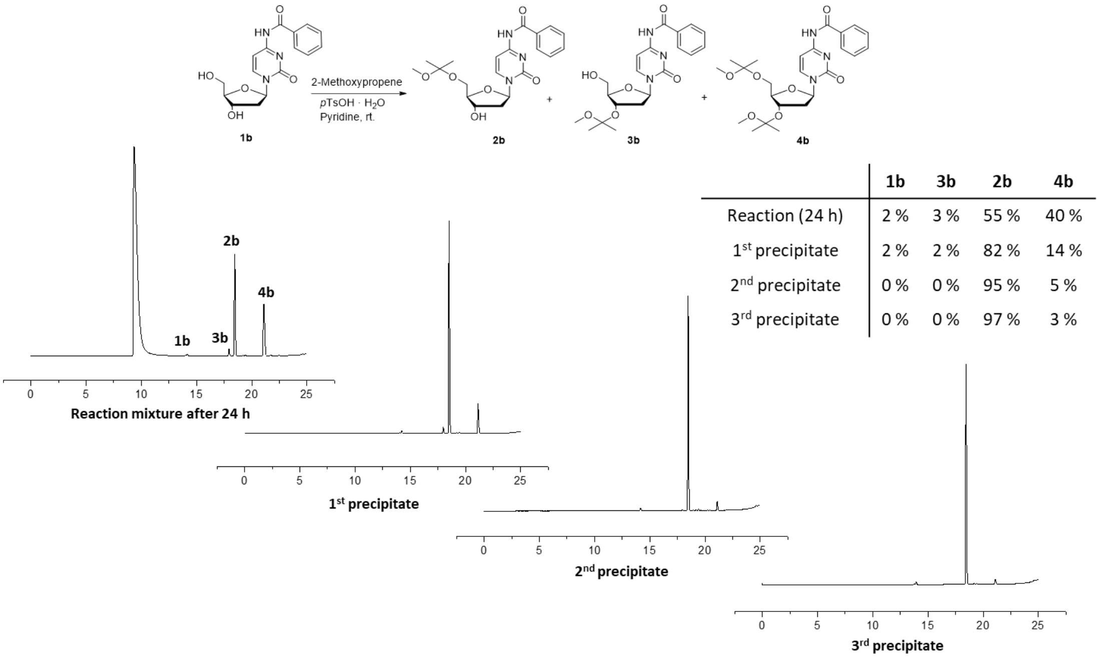

	1b	3b	2b	4b
Reaction (24 h)	2 %	3 %	55 %	40 %
1st precipitate	2 %	2 %	82 %	14 %
2nd precipitate	0 %	0 %	95 %	5 %
3rd precipitate	0 %	0 %	97 %	3 %

Figure S3. RP HPLC profile for acetalization of dC^{Bz} (**1b**) after 24h using 2-methoxypropene (4.0 eq.), *p*TsOH·H_2O (0.1 eq.) and pyridine (45.1 eq) and RP HPLC profiles for the precipitates after each precipitation in the mixture of ethyl acetate and hexane (1:4) showing the product purity.

Stability of 5´-MIP-protected phosphoramidite building blocks in acetonitrile

The phosphoramidite building blocks were dried under vacuum over P_2O_5 overnight and dissolved in anhydrous deuterated acetonitrile (0.1 mol L^{-1} solution). Samples were analyzed immediately and after three and seven days by ^1H NMR. Degradation was not observed.

Protection of exocyclic amino groups

General procedure for benzoylation of exocyclic amino groups

Nucleoside (1.0 eq.) was coevaporated three times with dry pyridine. Dried nucleoside was dissolved in dry pyridine (7.7 mL/mmol of nucleoside) and trimethylchlorosilane (8.0 eq.) was added to the mixture under nitrogen atmosphere at the room temperature. After 2 h, benzoyl chloride (5.0 eq.) was added to the mixture and reaction was stirred for 16 h. The mixture was cooled with ice-bath and water (1.7 mL/mmol of nucleoside) was added to the mixture. After 10 min, NH_4OH (25%) (3.4 mL/mmol of nucleoside) was added and the reaction mixture was stirred for 30 min at the room temperature. The reaction mixture was evaporated to dryness under reduced pressure and purified by column chromatography.

N^4-Benzoyl-2´-fluorocytidine (1f).

The synthesis was carried out as described in general procedure by using 2´-fluorocytidine (1.5 g, 4.30 mmol). The crude product was purified by silica gel column chromatography (EtOAc/MeOH, 90:10, *v*/*v*) to give **1f** as white solid (1.34 g, yield: 63%). ^{1}H NMR (500 MHz, DMSO-*d*6): δ 8.44 (d, J = 7.5 Hz, 1H), 8.01–7.99 (m, 2H), 7.62–7.59 (m, 1H), 7.51–7.48 (m, 2H), 7.28 (d, J = 7.5 Hz, 1H), 5.95 (d, J = 17.2 Hz, 1H), 4.98 (dd, J = 53.0 and 4.1 Hz, 1H), 4.21–4.13 (m, 1H), 3.96–3.94 (m, 1H), 3.86 (dd, J = 12.5 and 2.0 Hz, 1H), 3.66 (dd, J = 12.6 and 2.9 Hz, 1H). ^{13}C{^{1}H}NMR (126 MHz, DMSO-*d*6): δ 168.0, 164.1, 154.4, 144.4, 133.8, 132.5, 128.5, 128.4, 96.6, 94.1 (d, $J_{C–F}$ = 184.9 Hz), 88.6 (d, $J_{C–F}$ = 33.7 Hz), 83.1, 66.8 (d, $J_{C–F}$ = 16.4 Hz), 58.8. ^{19}F NMR (471 MHz, DMSO-*d*6): δ -201.24. HRMS (ESI): *m/z* [M + H]$^+$ calcd for $C_{16}H_{17}FN_3O_5^+$ 350.1147, found 350.1141.

N^2-Isobutyryl-2´-fluoroguanosine (1g).

The synthesis was carried out as described in general procedure by using 2´-fluoroguanosine (1.5 g, 5.3 mmol) and isobutyryl anhydride (4.36 mL, 26.9 mmol) instead of benzoyl chloride. The crude product was purified by silica gel column chromatography (EtOAc/MeOH, 85:15, *v*/*v*) to give **1g** as white solid (1.51 g, yield: 85%). ^{1}H NMR (500 MHz, DMSO-*d*6): δ 12.03 (br s, 2H), 8.26 (s, 1H), 6.12 (dd, J =16.2 and 2.5 Hz, 1H), 5.72 (br s, 1H), 5.36–5.24 (m, 1H), 5.20 (br s, 1H), 4.44–4.38 (m, 1H), 3.97–3.95 (m, 1H), 3.77–3.75 (m, 1H), 3.62–3.60 (m, 1H), 2.78 (septet, J = 6.8 Hz, 1H), 1.13 (s, 3H), 1.11 (s, 3H). ^{13}C{^{1}H}NMR (126 MHz, DMSO-*d*6): δ 180.3, 154.9, 148.4, 148.2, 137.3, 120.3, 93.8 (d, $J_{C–F}$ = 186.7 Hz), 85.3 (d, $J_{C–F}$ = 32.8 Hz), 83.8, 67.9 (d, $J_{C–F}$ = 16.0 Hz), 59.9, 34.8, 18.9, 18.9. ^{19}F NMR (471 MHz, DMSO-*d*6): δ -203.79. HRMS (ESI): *m/z* [M + H]$^+$ calcd for $C_{14}H_{19}FN_5O_5^+$ 356.1365, found 356.1374.

N^6-Benzoyl-2´-fluoroadenosine (1h).

The synthesis was carried out as described in general procedure by using 2´-fluoroadenosine (1.5 g, 4.0 mmol). The crude product was purified by silica gel column chromatography (EtOAc/MeOH, 93:7, *v*/*v*) to give **1h** as white solid (1.47 g, yield: 70%). ^{1}H NMR (500 MHz, DMSO-*d*6): δ 11.24 (br s, 1H), 8.77 (s, 1H), 8.71 (s, 1H), 8.05 (d, J = 7.3 Hz, 2H), 7.66–7.63 (m, 1H), 7.57–7.54 (m, 2H), 6.38 (dd, J = 17.2 and 2.4 Hz, 1H), 5.76 (d, J = 5.2 Hz, 1H), 5.58–5.47 (m, 1H), 5.17 (s, 1H), 4.56–4.53 (m, 1H), 4.03–4.01 (m, 1H), 3.80–3.78 (m, 1H), 3.63–3.61 (m, 1H). ^{13}C{^{1}H}NMR (126 MHz, DMSO-*d*6): δ 165.6, 151.8, 151.7, 150.5, 142.8, 132.5, 129.3, 128.5, 128.5, 125.8, 93.5 (d, $J_{C–F}$ = 186.5 Hz), 86.0 (d, $J_{C–F}$ = 33.5 Hz), 84.0, 68.2 (d, $J_{C–F}$ = 16.0 Hz), 60.1. ^{19}F NMR (471 MHz, DMSO-*d*6): δ -203.56. HRMS (ESI): *m/z* [M + H]$^+$ calcd for $C_{17}H_{17}FN_5O_4^+$ 374.1259, found 374.1267.

N^6-Benzoyl-2´-*O*-methyladenosine (1i).

The synthesis was carried out as described in general procedure by using 2´-*O*-methyladenosine (1.5 g, 3.9 mmol). The crude product was purified by silica gel column chromatography (EtOAc/MeOH, 93:7, *v/v*) to give **1i** as white solid (1.46 g, yield: 94%). ^{1}H NMR (500 MHz, DMSO-*d*6): δ 11.19 (br s, 1H), 8.71 (s, 1H), 8.70 (s, 1H), 8.06–8.05 (m, 2H), 7.63–7.60 (m, 1H), 7.55–7.52 (m, 2H), 6.15 (d, J = 5.8 Hz, 1H), 5.27 (m, 2H), 4.44–4.42 (m, 1H), 4.39–4.37 (m, 1H), 4.02–4.00 (m, 1H), 3.70 (dd, J = 12.0 and 3.9 Hz, 1H), 3.60 (dd, J = 12.0 and 3.8 Hz, 1H), 3.35 (s, 3H). ^{13}C{^{1}H}NMR (126 MHz, DMSO-*d*6): δ 166.1, 151.8, 151.8, 142.5, 134.2, 132.1, 128.5, 128.4, 125.9, 86.3, 85.6, 82.6, 68.7, 61.2, 57.6. HRMS (ESI): *m/z* $[M + H]^+$ calcd for $C_{18}H_{20}N_5O_5^+$ 386.1459, found 386.1457.

N^6-Benzoyl-2´-*O*-methoxyethyladenosine (1j).

The synthesis was carried out as described in general procedure by using 2´-*O*-methoxyadenosine (1.5 g, 3.5 mmol). The crude product was purified by silica gel column chromatography (EtOAc/MeOH, 90:10, *v/v*) to give **1j** as white solid (1.79 g, yield: 90%). ^{1}H NMR (500 MHz, $CDCl_3$): δ 9.19 (s, 1H), 8.76 (s, 1H), 8.06 (s, 1H), 8.02–8.00 (m, 2H), 7.62–7.59 (m, 1H), 7.53–7.50 (m, 2H), 6.12 (d, J = 11.4 Hz, 1H), 5.94 (d, J = 7.7 Hz, 1H), 4.80 (dd, J = 7.7 and 4.4 Hz, 1H), 4.53 (d, J = 4.4 Hz, 1H), 4.36 (m, 1H), 4.12 (s, 1H), 3.98–3.96 (m, 1H), 3.78–3.76 (m, 1H), 3.75–3.72 (m, 1H), 3.58–3.49 (m, 2H), 3.42–3.39 (m, 1H), 3.36 (s, 3H). ^{13}C{^{1}H}NMR (126 MHz, $CDCl_3$): δ 164.7, 152.2, 150.8, 150.5, 143.5, 133.5, 133.1, 129.0, 128.0, 124.9, 89.8, 88.2, 81.9, 71.6, 70.7, 69.7, 63.5, 59.1. HRMS (ESI): *m/z* $[M + H]^+$ calcd for $C_{20}H_{24}N_5O_6^+$ 430.1721, found 430.1736.

NMR spectra of exocyclic amino group protected nucleosides

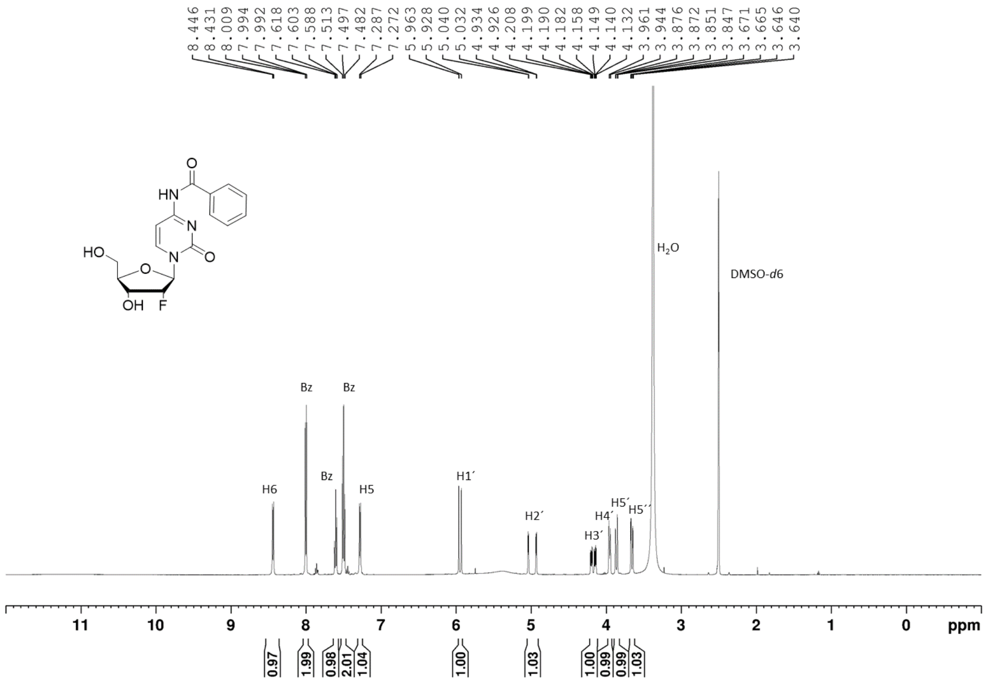

Figure S4. ^{1}H NMR (500 MHz, DMSO-*d*6) spectrum of **1f**

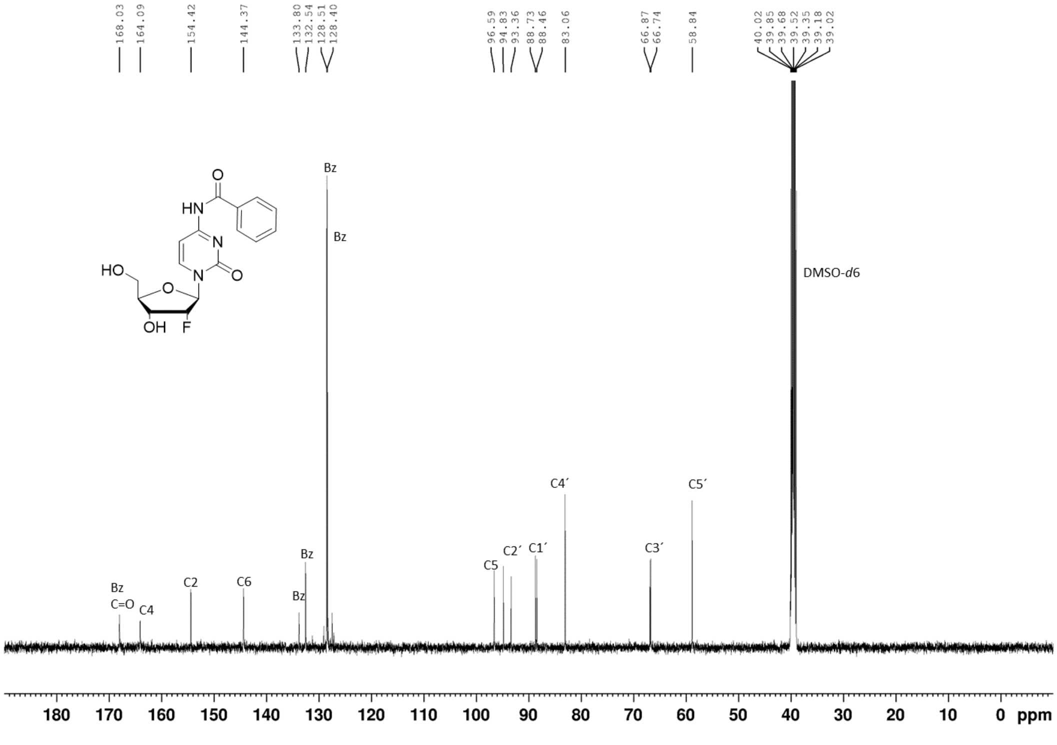

Figure S5. ^{13}C NMR (126 MHz, DMSO-*d*6) spectrum of **1f**

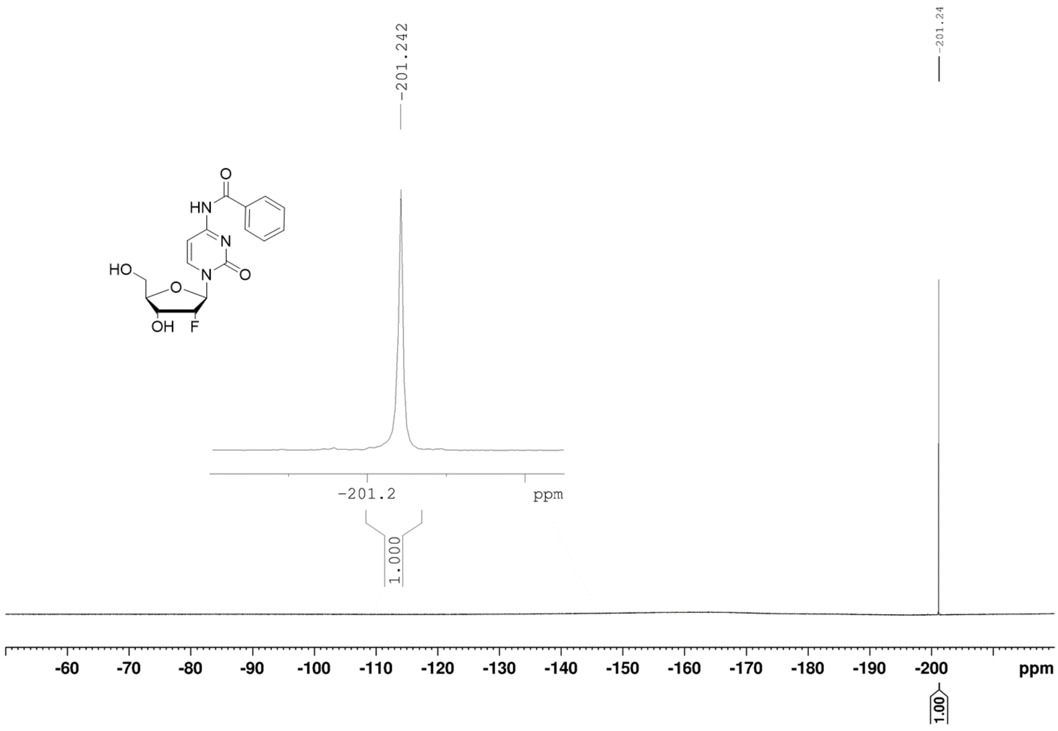

Figure S6. ^{19}F NMR (471 MHz, DMSO-*d*6) spectrum of **1f**

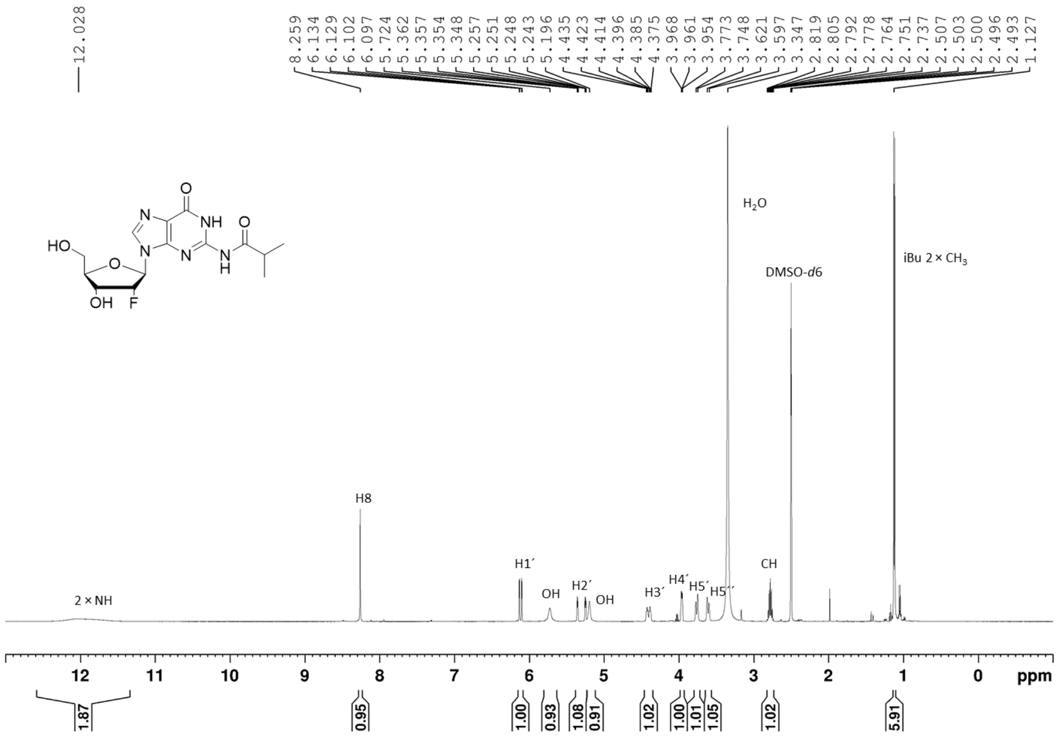

Figure S7. ^{1}H NMR (500 MHz, DMSO-*d*6) spectrum of **1g**

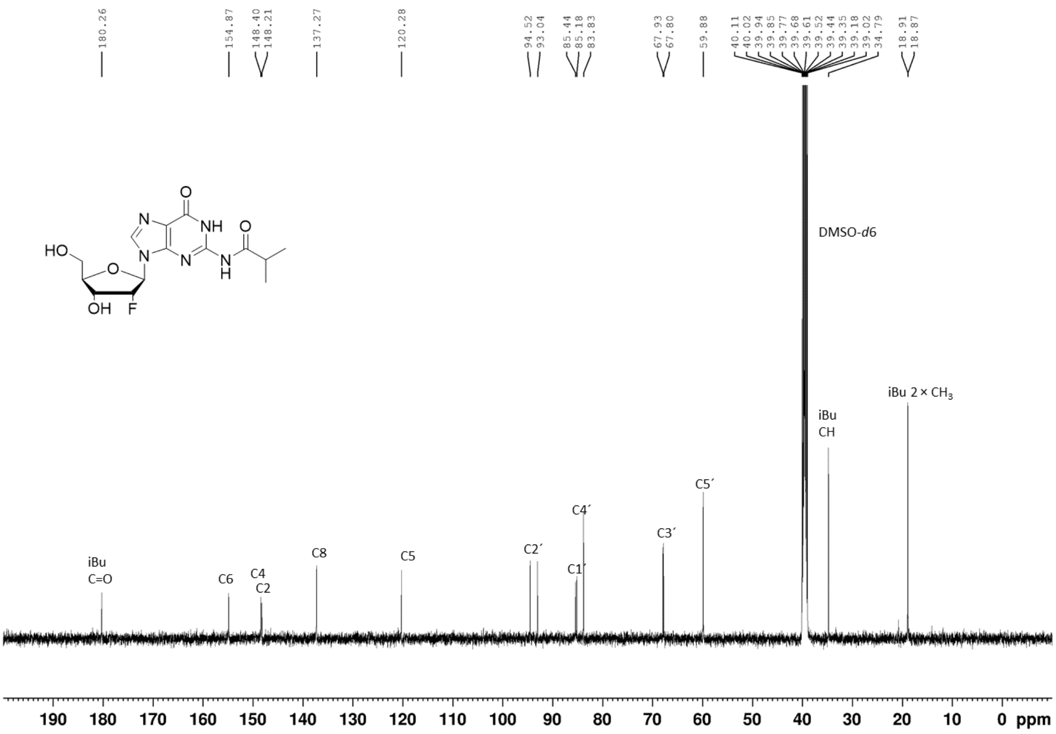

Figure S8. ^{13}C NMR (126 MHz, DMSO-*d*6) spectrum of **1g**

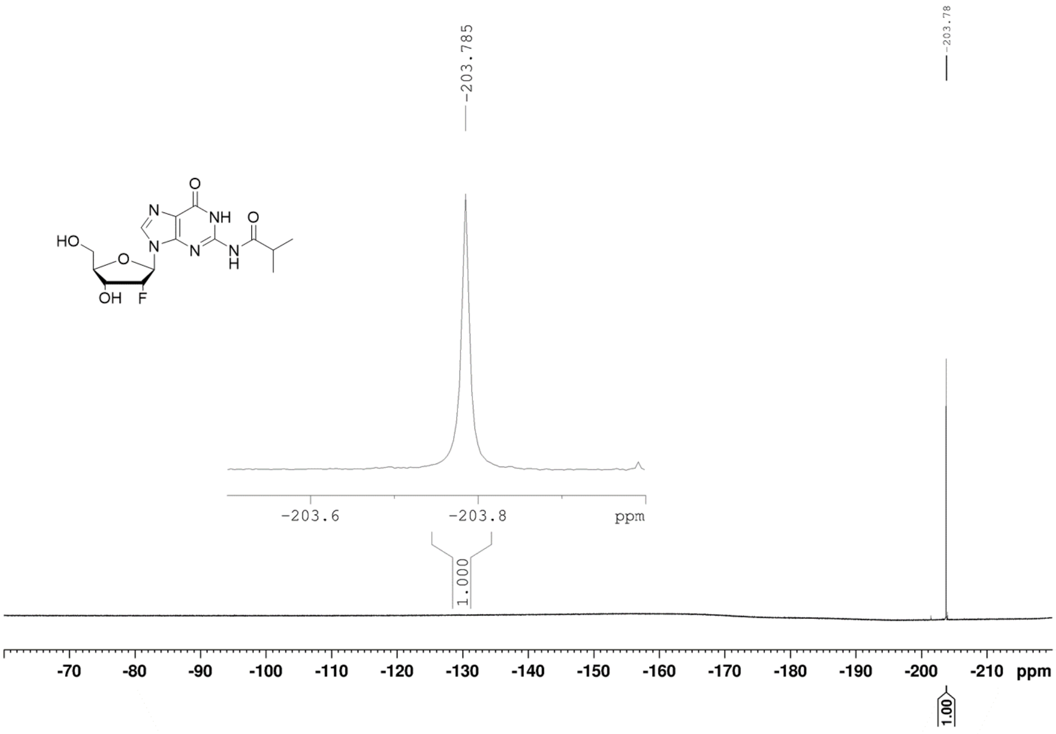

Figure S9. ^{19}F NMR (471 MHz, DMSO-*d*6) spectrum of **1g**

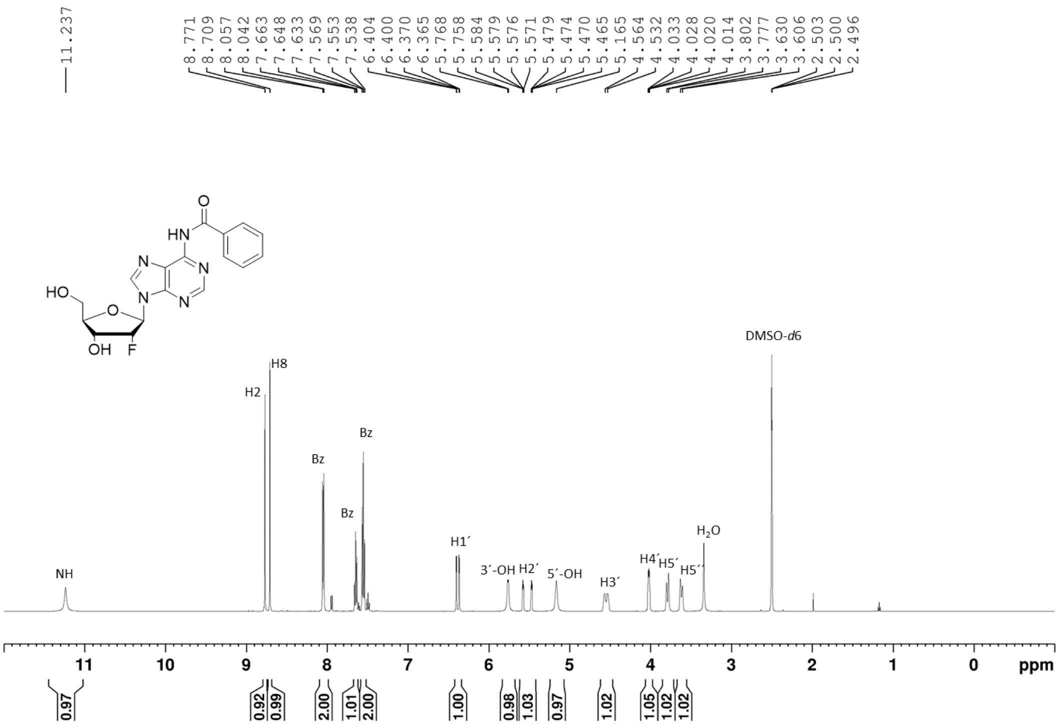

Figure S10. ^{1}H NMR (500 MHz, DMSO-*d*6) spectrum of **1h**

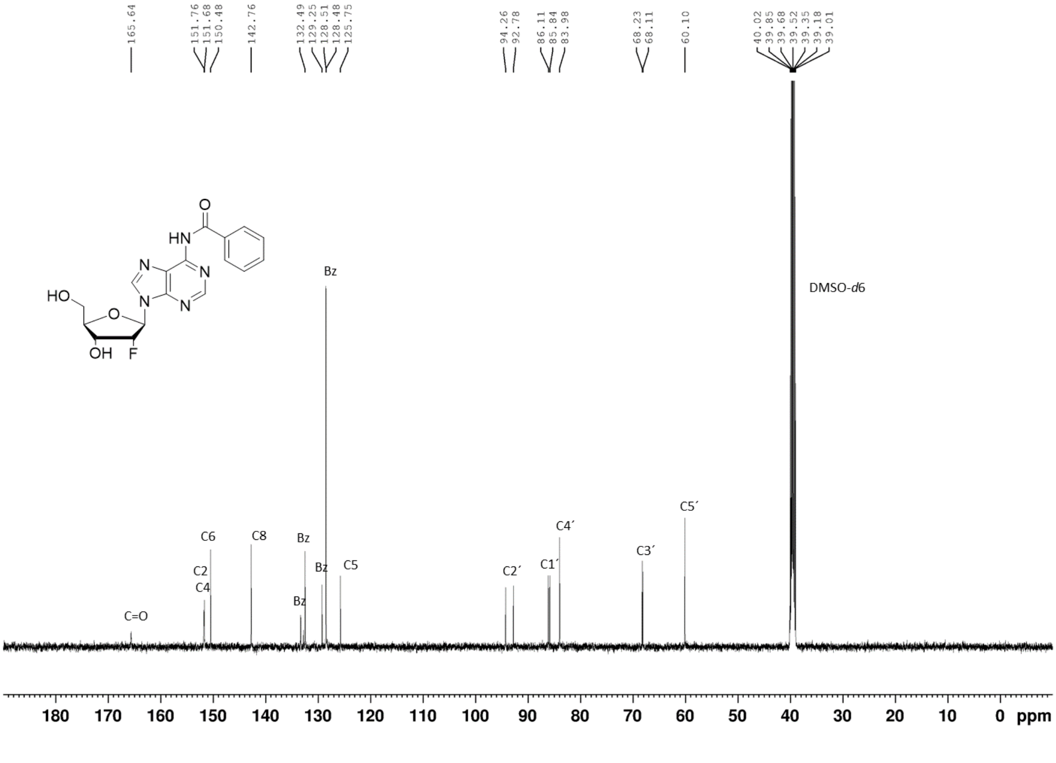

Figure S11. ^{13}C NMR (126 MHz, DMSO-*d*6) spectrum of **1h**

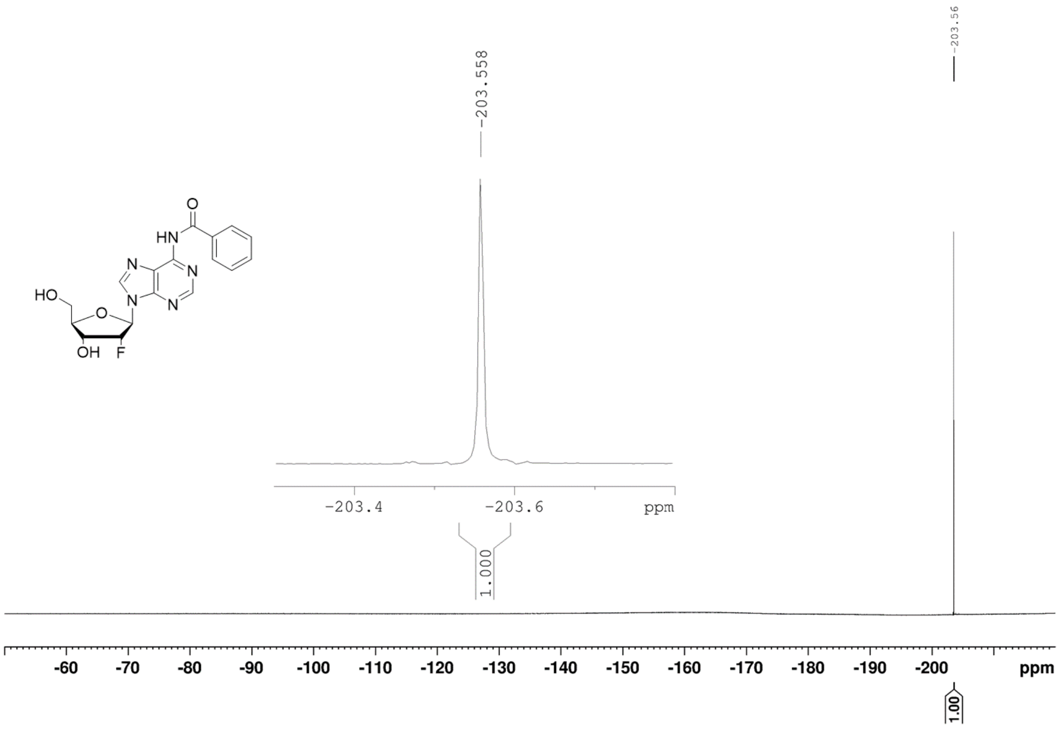

Figure S12. ^{19}F NMR (471 MHz, DMSO-*d*6) spectrum of **1h**

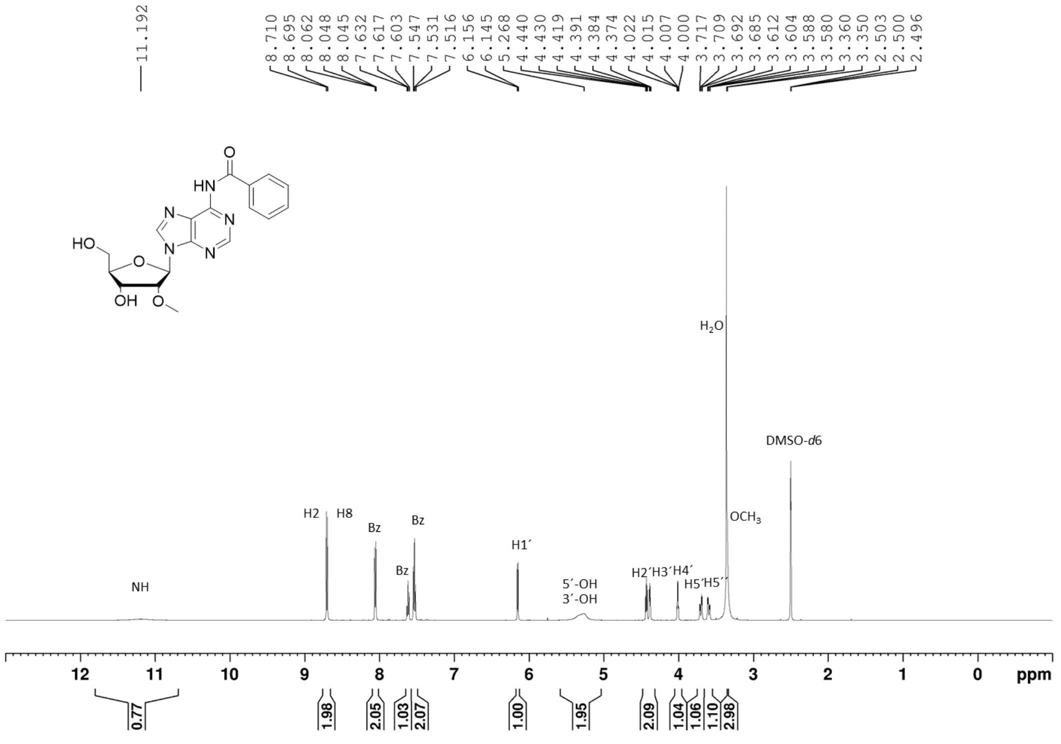

Figure S13. ^{1}H NMR (500 MHz, DMSO-*d*6) spectrum of **1i**

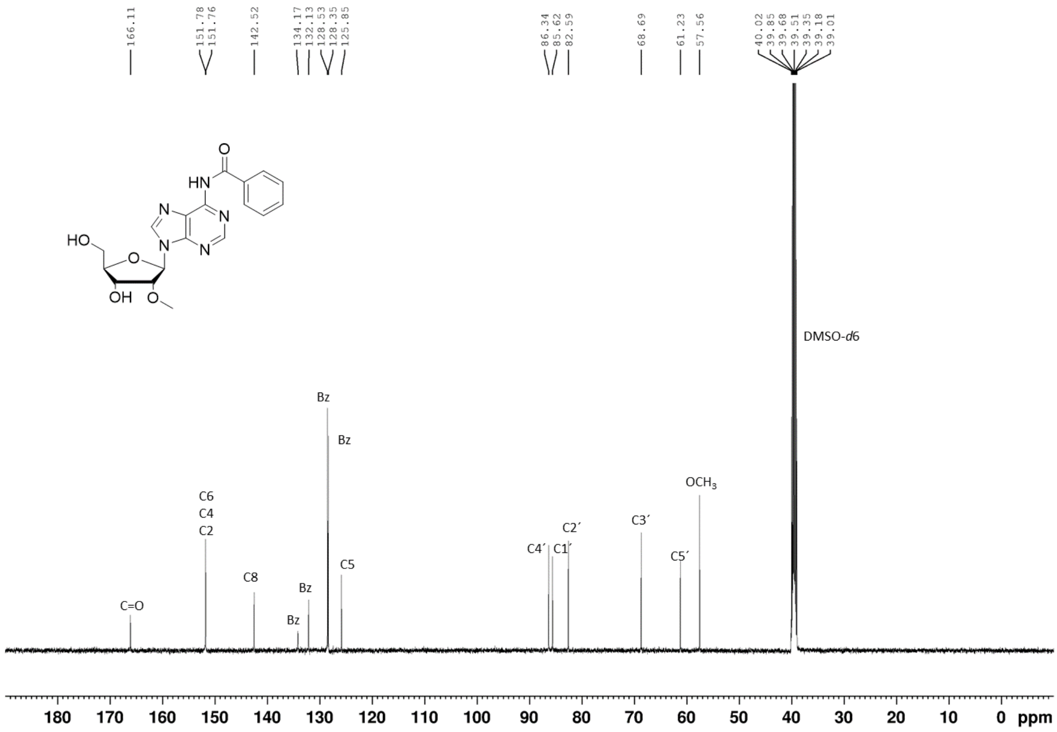

Figure S14. ^{13}C NMR (126 MHz, DMSO-*d*6) spectrum of **1i**

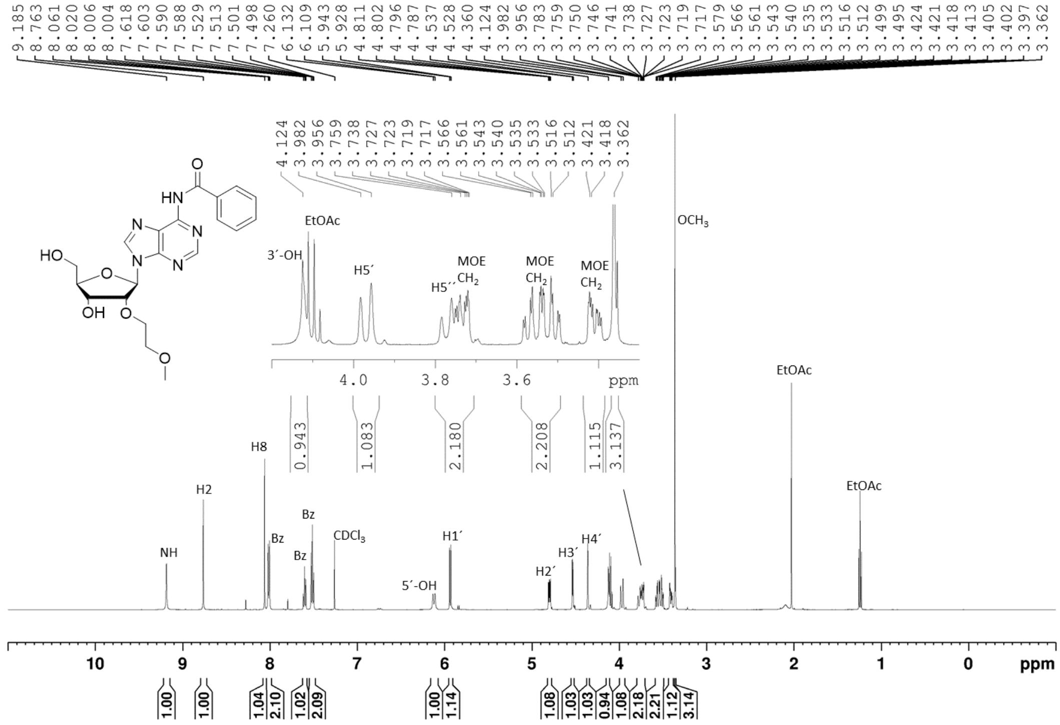

Figure S15. ^{1}H NMR (500 MHz, $CDCl_3$) spectrum of **1j**

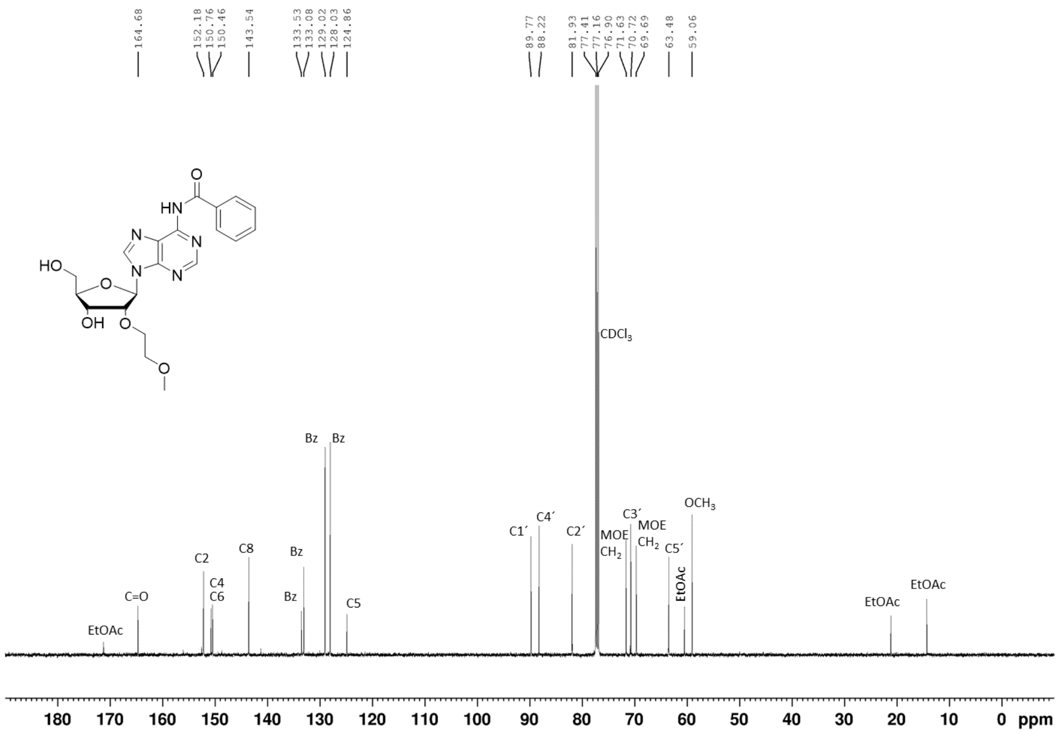

Figure S16. ^{13}C NMR (126 MHz, $CDCl_3$) spectrum of **1j**

Acetalizations

General procedure for selective 5´-acetalization

2-Methoxypropene (2–4 eq.) was added to a mixture of nucleoside (1.0 eq., dried under vacuum over P_2O_5 overnight) in the presence of *p*TsOH·H_2O (0.1 eq.) in anhydrous pyridine (0.55 or 0.28 L/mol of nucleoside). The reaction was stirred for 24 h at the room temperature. Completeness of the reaction was confirmed with RP HPLC-analysis. The mixture was diluted with DCM and washed with saturated aqueous $NaHCO_3$. Organic phase was separated, dried over Na_2SO_4 and concentrated under reduced pressure. The crude product was purified by silica gel chromatography or precipitation.

5´-*O*-(2-methoxyprop-2-yl)-thymidine (2a).

The synthesis was carried out as described in general procedure by using thymidine (1.00 g, 4.13 mmol), 2-methoxypropene (1.22 mL, 12.4 mmol), *p*TsOH·H_2O (81 mg, 0.41 mmol) and pyridine (15 mL). The reaction was monitored by RP HPLC using gradient A. The crude product was precipitated twice in EtOAc/hexane (2×65 mL) (1:4, *v*/*v*) and filtered to give **2a** as white solid (0.57 g, yield: 44%). The ^{1}H and ^{13}C NMR spectra of **2a** were identical to those previously reported in literature.[1] (cf. purity documentation of the product in Figure 120.)

3´-*O*-(2-methoxyprop-2-yl)-thymidine (3a) and 5´,3´-*O*-bis-(2-methoxyprop-2-yl)-thymidine (4a).

Analytical samples of side products **3a** and **4a** in the supernatant were purified by column chromatography (DCM/MeOH/Pyridine, 91:8:1, *v*/*v*/*v*). **3a** was obtained as white solid (conversion yield based on RP HPLC analysis: 2%) and **4a** as yellowish foam (conversion yield based on RP HPLC analysis: 44%). **3a**: ^{1}H NMR (500 MHz, $CDCl_3$): δ 9.88 (s, 1H), 7.51 (d, *J* = 1.1 Hz, 1H), 6.20 (dd, *J* = 7.0 Hz, 1H), 4.53–4.50 (m, 1H), 3.99–3.97 (m, 1H), 3.87 (dd, *J* = 12.1 and 2.7 Hz, 1H), 3.75 (dd, *J* = 12.1 and 3.1 Hz, 1H), 3.16 (s, 3H), 2.28–2.19 (m, 2H), 1.81 (d, *J* = 1 Hz, 3H), 1.31 (s, 6H). ^{13}C{^{1}H}NMR (126 MHz, $CDCl_3$): δ 164.4, 150.7, 136.7, 110.9, 101.1, 86.2, 85.9, 69.9, 62.0, 48.9, 39.7, 25.3, 25.0, 12.5. HRMS (ESI): *m/z* $[M + Na]^+$ calcd for $C_{14}H_{22}N_2NaO_6^+$ 337.13701, found 337.13756. **4a**: ^{1}H NMR (500 MHz, $CDCl_3$): δ 9.02 (br s, 1H), 7.53 (d, *J* = 1.1 Hz, 1H), 6.34 (dd, *J* = 6.2 and 1.3 Hz, 1H), 4.45–4.43 (m, 1H), 4.12–4.11 (m, 1H), 3.67 (dd, *J* = 10.8 an 2.9 Hz, 1H), 3.56 (dd, *J* = 10.8 and 2.9 Hz, 1H), 3.23 (s, 3H), 3.19 (s, 3H), 2.34–2.29 (m, 1H), 2.10–2.04 (m, 1H), 1.92 (d, *J* = 1.0 Hz), 1.39 (d, *J* = 4.9 Hz, 6H), 1.34 (s, 1H). ^{13}C{^{1}H}NMR (126 MHz, $CDCl_3$): δ 163.9, 150.5, 135.7, 110.9, 101.1, 100.4, 85.2, 85.1, 70.8, 60.8, 49.0, 48.9, 40.2, 25.4, 25.2, 24.6, 24.5, 12.7. HRMS (ESI): *m/z* $[M + Na]^+$ calcd for $C_{18}H_{30}N_2NaO_7^+$ 409.1945, found 409.1949.

***N*⁴-Benzoyl-5´-*O*-(2-methoxyprop-2-yl)-2´-deoxycytidine (2b).**

The synthesis was carried out as described in general procedure by using N^4-benzoyl-2´-deoxycytidine (1.00 g, 2.96 mmol), 2-methoxypropene (1.17 mL, 11.83 mmol), *p*TsOH · H_2O (58 mg, 0.30 mmol) and pyridine (11 mL). The reaction was monitored by RP HPLC using gradient B. The crude product was precipitated three times in EtOAc/hexane (3×65 mL) (1:4, *v*/*v*) and filtered to give **2b** as white solid (0.56 g, yield: 47%). The ^{1}H and ^{13}C NMR spectra of **2b** were identical to those previously reported in literature.[1] (cf. purity documentation of the product in Figure 121.)

***N*⁴-Benzoyl-5´,3´-*O*-bis-(2-methoxyprop-2-yl)-2´-deoxycytidine (4b).**

An analytical sample of **4b** in the supernatant was purified by column chromatography (DCM/MeOH/Pyridine, 96:3:1, *v*/*v*/*v*) to give yellowish foam (conversion yield based on RP HPLC analysis: 40%). ^{1}H NMR (500 MHz, $CDCl_3$): δ 8.69 (br s, 1H), 8.48 (d, *J* = 7.5 Hz, 1H), 7.89–7.88 (m, 2H), 7.62–7.59 (m, 1H), 7.52–7.49 (m, 3H), 6.29 (dd, *J* = 5.9 and 5.9 Hz, 1H), 4.45–4.42 (m, 1H), 4.18–4.16 (m, 1H), 3.75 (dd, *J* = 10.8 and 3.2 Hz, 1H), 3.59 (dd, *J* = 10.8 and 2.8 Hz), 3.23 (s, 3H), 3.19 (s, 3H), 2.62–2.57 (m, 1H), 2.21–2.16 (m, 1H), 1.40 (s, 3H), 1.37 (s, 3H), 1.33 (s, 6H). ^{13}C{^{1}H}NMR (126 MHz, $CDCl_3$): δ 162.1, 144.95, 133.3, 129.18, 127.61, 101.1, 100.5, 96.0, 87.4, 85.4, 69.5, 59.8, 49.1, 49.0, 41.4, 25.4, 25.1, 24.5, 24.5. HRMS (ESI): *m/z* $[M + H]^+$ calcd for $C_{24}H_{34}N_3O_7^+$ 476.2391, found 476.2390.

N^2-Isobutyryl-5´-*O*-(2-methoxyprop-2-yl)-2´-deoxyguanosine (2c).

The synthesis was carried out as described in general procedure by using N^2-isobutyryl-2´-deoxyguanoside (1.00 g, 2.91 mmol), 2-methoxypropene (0.57 mL, 5.81 mmol), *p*TsOH · H_2O (57 mg, 0.29 mmol) and pyridine (11 mL). The reaction was monitored by RP HPLC using gradient C. The crude product was purified by silica gel column chromatography (EtOAc/MeOH/Pyridine, 91:8:1, *v*/*v*/*v*) to give **2c** as white solid (0.89 g, yield: 75%). The ^{1}H and ^{13}C NMR spectra of **2c** were identical to those previously reported in literature.[2] (cf. purity documentation of the product in Figure 122.)

N^2-Isobutyryl-5´,3´-*O*-bis-(2-methoxyprop-2-yl)-2´-deoxyguanosine (4c).

An analytical sample of **4c** was purified by column chromatography (EtOAc/MeOH/Pyridine, 91:8:1, *v*/*v*/*v*) to give yellowish foam (conversion yield based on RP HPLC analysis: 6%). ^{1}H NMR (500 MHz, $CDCl_3$): δ 12.09 (s, 1H), 9.53 (s, 1H), 7.96 (s, 1H), 6.17 (dd, *J* = 6.5 and 6.5 Hz, 1H), 4.64–4.61 (m, 1H), 4.15–4.12(m, 1H), 3.73 (dd, *J* = 10.6 and 4.3 Hz, 1H), 3.50 (dd, *J* = 10.6 and 3.9 Hz, 1H), 3.18 (s, 3H). 3.11 (s, 3H), 2.75–2.69 (m, 1H), 2.66–2.60 (m, 1H), 2.40–2.35 (m, 1H), 1.33–1.31 (4 × s, 12H), 1.22 (d, *J* = 6.9 Hz, 3H), 1.20 (d, *J* = 6.9 Hz, 3H). ^{13}C{^{1}H}NMR (126 MHz, $CDCl_3$): δ 179.1, 155.8, 148.1, 147.7, 137.5, 137.5, 121.5, 101.2, 100.6, 85.3, 84.3, 70.6, 60.5, 49.0, 48.7, 39.8, 36.4, 25.4, 25.1, 24.5, 24.3, 19.1, 19.0. HRMS (ESI): *m/z* $[M + H]^+$ calcd for $C_{22}H_{36}N_5O_7^+$ 482.2609, found 482.2595.

N^6-Benzoyl-5´-*O*-(2-methoxyprop-2-yl)-2´-deoxyadenosine (2d).

The synthesis was carried out as described in general procedure by using N^6-benzoyl-2´-deoxyadenoside (1.00 g, 2.76 mmol), 2-methoxypropene (1.09 mL, 11.03 mmol), *p*TsOH · H_2O (54 mg, 0.28 mmol) and pyridine (10 mL). The reaction was monitored by RP HPLC using gradient B. The crude product was purified by silica gel column chromatography (DCM/MeOH/Pyridine, 94:4:1, *v*/*v*/*v*) to give **2d** as white solid (0.85 g, yield: 72%). The ^{1}H and ^{13}C NMR spectra of **2d** were identical to those previously reported in literature.[1] (cf. purity documentation of the product in Figure 123.)

N^6-Benzoyl-5´,3´-*O*-bis-(2-methoxyprop-2-yl)-2´-deoxyadenosine (4d).

An analytical sample of **4d** was purified by column chromatography (DCM/MeOH/Pyridine, 94:4:1, *v*/*v*/*v*) to give yellowish foam (conversion yield based on RP HPLC analysis: 21%). ^{1}H NMR (500 MHz, $CDCl_3$): δ 9.19 (br s, 1H), 8.76 (s, 1H), 8.39 (s, 1H), 8.01 (d, *J* = 7.6 Hz, 2H), 7.59–7.56 (m, 1H), 7.50–7.47 (m, 2H), 6.53 (dd, *J* = 6.5 and 6.5 Hz, 1H), 4.65–4.63 (m, 1H), 4.23–4.21(m, 1H), 3.53 (dd, *J* = 10.6 and 3.5 Hz, 1H), 3.58 (10.7 and 3.7 Hz, 1H), 3.21 (s, 1H), 3.15 (s, 3H), 2.71–2.65 (m, 1H), 2.55–2.51 (m, 1H), 1.37 (s, 6H), 1.35 (s, 3H), 1.34 (s, 3H). ^{13}C{^{1}H}NMR (126 MHz, $CDCl_3$): δ 164.8, 152.6, 151.7, 149.5, 141.6, 133.8, 132.8, 128.9, 128.0, 123.5, 101.2, 100.4, 85.5, 84.7, 70.7, 60.7, 49.1, 48.8, 40.6, 25.4, 25.2, 24.5, 24.4. HRMS (ESI): *m/z* $[M + H]^+$ calcd for $C_{25}H_{34}N_5O_6^+$ 500.2504, found 500.2502.

5´-*O*-(2-methoxyprop-2-yl)-2´-fluorouridine (2e).

The synthesis was carried out as described in general procedure by using 2´-F-uridine (0.83 mg, 3.36 mmol), 2-methoxypropene (0.66 mL, 6.73 mmol), *p*TsOH · H_2O (65 mg, 0.34 mmol) and pyridine (6.1 mL). The reaction was monitored by RP HPLC using gradient A. The crude product was purified by silica gel column chromatography (DCM/MeOH/Pyridine, 95:4:1 → 93:6:1, *v*/*v*/*v*) to give **2e** as white solid (0.82 g, yield: 76%). ^{1}H NMR (500 MHz, $CDCl_3$): δ 9.65 (br s, 1H), 7.97 (d, J = 8.2 Hz, 1H), 6.04 (dd, J = 15.6 and 1.4 Hz, 1H), 5.69 (d, J = 8.2 Hz, 1H), 4.99 (ddd, J = 52.5, 4.2 and 1.3 Hz, 1H), 4.42–4.35 (m, 1H), 4.20–4.18 (m, 1H), 3.85 (dd, J = 11.3 and 1.3 Hz, 1H), 3.68 (dd, J = 11.3 and 2.7 Hz, 1H), 3.20 (s, 3H), 1.39 (s, 3H), 1.38 (s, 3H). ^{13}C{^{1}H}NMR (126 MHz, $CDCl_3$): δ 163.6, 150.26, 140.32, 102.2, 100.6, 94.0 (d, $J_{C–F}$ = 187.1 Hz), 88.0 (d, $J_{C–F}$ = 34.0 Hz), 82.4, 68.9 (d, $J_{C–F}$ = 17.0 Hz), 58.7, 48.9, 24.4, 24.4. ^{19}F NMR (471 MHz, $CDCl_3$): -203.36. HRMS (ESI): *m/z* [M + Na]$^+$ calcd for $C_{13}H_{19}FN_2NaO_6^+$ 341.11194, found 341.1128.

5´,3´-*O*-bis-(2-methoxyprop-2-yl)-2´-fluorouridine (4e).

An analytical sample of **4e** was purified by column chromatography (DCM/MeOH/Pyridine, 95:4:1 → 93:6:1, *v*/*v*/*v*) to give yellowish foam (conversion yield based on RP HPLC analysis: 23%). ^{1}H NMR (500 MHz, $CDCl_3$): δ 9.27 (s, 1H), 8.04 (d, J = 8.1 Hz, 1H), 6.04 (d, J = 16.7 Hz, 1H), 5.68 (d, J = 8.1 Hz, 1H), 4.95–4.84 (m, 1H), 4.38-4.32 (m, 1H), 4.22–4.21 (m, 1H), 3.90–3.88 (m, 1H), 3.56–3.53 (m, 1H), 3.27 (s, 3H), 3.20 (s, 3H), 1.42 (s, 3H), 1.39 (s, 3H), 1.37 (s, 6H). ^{13}C{^{1}H}NMR (126 MHz, $CDCl_3$): δ 163.3, 150.1, 140.1, 102.1, 101.9, 100.6, 93.0 (d, $J_{C–F}$ = 191.2 Hz), 88.7 (d, $J_{C–F}$ = 34.8 Hz), 80.9, 67.2 (d, $J_{C–F}$ = 16.2 Hz), 58.1, 49.6, 49.6, 48.9, 25.0, 24.9, 24.6, 24.2.^{19}F NMR (471 MHz, $CDCl_3$): -200.16. HRMS (ESI): *m/z* [M + Na]$^+$ calcd for $C_{17}H_{27}FN_2NaO_7^+$ 413.16945, found 413.1683.

***N*^{4}-Benzoyl-5´-*O*-(2-methoxyprop-2-yl)-2´-fluorocytidine (2f).**

The synthesis was carried out as described in general procedure by using *N*^{4}-benzoyl-2´-F-cytidine (420 mg, 1.20 mmol), 2-methoxypropene (0.24 mL, 2.40 mmol), *p*TsOH · H_2O (24 mg, 0.12 mmol) and pyridine (2.2 mL). The reaction was monitored by RP HPLC using gradient B. The crude product was purified by silica gel column chromatography (DCM/MeOH/Pyridine, 95:4:1, *v*/*v*/*v*) to give **2f** as white solid (0.39 g, yield: 77%). ^{1}H NMR (500 MHz, $CDCl_3$): δ 9.35 (br s, 1H), 8.56 (d, J = 7.5 Hz, 1H), 7.95 (d, J = 7.8 Hz, 2H), 7.60–7.58 (m, 1H), 7.54–7.48 (m, 3H), 6.57 (br d, J = 141.9 Hz, 1H), 6.08 (d, J = 15.5 Hz, 1H), 5.13–5.03 (m, 1H), 4.41–4.34 (m, 1H), 4.27–4.25 (m, 1H), 3.93–3.91 (m, 1H), 3.74–3.72 (m, 1H), 3.22 (s, 3H), 1.42 (s, 3H), 1.40 (s, 3H). ^{13}C{^{1}H}NMR (126 MHz, $CDCl_3$): δ 167.1, 163.1, 155.0, 145.0, 133.5, 133.4, 129.0, 128.0, 100.6, 96.7, 94.1 (d, $J_{C–F}$ = 186.7 Hz), 89.2 (d, $J_{C–F}$ = 33.6 Hz), 82.1, 68.0 (d, $J_{C–F}$ = 17.2 Hz), 57.9, 48.9, 24.5, 24.4. ^{19}F NMR (471 MHz, $CDCl_3$): δ -202.80 HRMS (ESI): *m/z* [M + H]$^+$ calcd for $C_{20}H_{25}FN_3O_6^+$ 422.1722, found 422.1733.

N^4-Benzoyl-5´,3´-*O*-bis-(2-methoxyprop-2-yl)-2´-fluorocytidine (4f).

An analytical sample **4f** was purified by column chromatography (DCM/MeOH/Pyridine, 95:4:1, *v*/*v*/*v*) to give yellowish foam (conversion yield based on RP HPLC analysis: 8%). ^{1}H NMR (500 MHz, $CDCl_3$): δ 8.98 (s, 1H), 8.65 (d, J = 7.5 Hz, 1H), 7.91 (d, J = 7.4 Hz, 2H), 7.59 (t, J = 7.5 Hz, 1H), 7.51–7.48 (m, 3H), 6.11 (d, J = 16.1 Hz, 1H), 5.02–4.91 (m, 1H), 4.34–4.28 (m, 2H), 3.97 (dd, J = 11.2 and 1.0 Hz), 3.57 (dd, J = 11.5 and 1.0 Hz), 3.23 (s, 3H), 3.23 (s, 3H), 1.42 (s, 3 H), 1.39 (s, 6H), 1.35 (s, 3H). ^{13}C{^{1}H}NMR (126 MHz, $CDCl_3$): δ 166.8, 162.7, 144.7, 133.3, 133.0, 129.1, 127.7, 101.8, 100.5, 96.3, 93.7, 96.3, 92.9 (d, $J_{C–F}$ = 190.9 Hz), 89.7 (d, $J_{C–F}$ = 34.1 Hz) , 80.6, 66.5 (d, $J_{C–F}$ = 16.5 Hz), 57.5, 49.6, 49.6, 48.9, 24.9, 24.9, 24.6, 24.3. ^{19}F NMR (471 MHz, $CDCl_3$): δ -200.22. HRMS (ESI): *m/z* $[2M + H]^+$ calcd for $C_{24}H_{33}FN_3O_7^+$ 987.4521, found 987.4557.

N^2-Isobutyryl-5´-(2-methoxyprop-2-yl)-2´-fluoroguanosine (2g).

The synthesis was carried out as described in general procedure by using N^2-isobutyryl-2´-F-guanosine (0.50 g, 1.41 mmol), 2-methoxypropene (0.56 mL, 5.63 mmol), *p*TsOH · H_2O (28 mg, 0.14 mmol) and pyridine (5.1 mL). The reaction was monitored by RP HPLC using gradient C. The crude product was purified by silica gel column chromatography (DCM/MeOH/Pyridine, 89:10:1, *v*/*v*/*v*) to give **2g** as white solid (420 mg, yield: 69%). ^{1}H NMR (500 MHz, $CDCl_3$): δ 12.47 (s, 1H), 10.91 (s, 1H), 8.04 (s, 1H), 5.88 (d, J = 17.4 Hz, 1H), 5.17–5.06 (m, 1H), 4.72–4.65 (m, 1H), 4.22–4.22 (m, 1H), 3.77 (dd, J = 9.8 and 0.0 Hz, 1H), 3.62 (dd, J = 11.1 and 3.8 Hz, 1H), 3.12 (s, 3H), 2.95–2.90 (m, 1H), 1.33 (s, 3H), 1.32 (s, 3H), 1.28 (d, J = 6.7 Hz, 3H), 1.23 (d, J = 6.7 Hz, 3H). ^{13}C{^{1}H}NMR (126 MHz, $CDCl_3$): δ 180.4, 156.0, 148.5, 148.3, 137.8, 120.8, 100.5, 94.2 (d, $J_{C–F}$ = 187.2 Hz), 86.9 (d, $J_{C–F}$ = 33.7 Hz), 82.4, 69.1 (d, $J_{C–F}$ = 16.2 Hz), 59.6, 48.6, 36.2, 30.9, 24.3, 24.2, 19.1, 19.0. ^{19}F NMR (471 MHz, $CDCl_3$): δ -203.34. HRMS (ESI): *m/z* $[M + H]^+$ calcd for $C_{18}H_{27}FN_5O_6^+$ 428.1940, found 428.1936.

N^2-Isobutyryl-5´,3´-*O*-bis-(2-methoxyprop-2-yl)-2´-fluoroguanosine (4g).

An analytical sample of **4g** was purified by column chromatography (DCM/MeOH/Pyridine, 89:10:1, *v*/*v*/*v*) to give yellowish foam (conversion yield based on RP HPLC analysis: 3%). ^{1}H NMR (500 MHz, $CDCl_3$): δ 12.04 (s, 1H), 9.01 (s, 1H), 8.07 (s, 1H), 6.05 (dd, J = 16.6 and 0.5 Hz, 1H), 5.12–5.11 (m, 1H), 4.83–4.76 (m, 1H), 4.25–4.23 (m, 1H), 3.86 (dd, J = 11.2 and 2.3 Hz, 1H), 3.51 (dd, J = 11.3 and 2.8 Hz, 1H), 3.27 (s, 3H), 3.10 (s, 3H), 2.72–2.66 (m, 1H), 1.43 (s, 3H), 1.40 (s, 3H), 1.33 (s, 3H), 1.31 (s, 3H), 1.28–1.26 (m, 6H). ^{13}C{^{1}H}NMR (126 MHz, $CDCl_3$): δ 178.8, 155.6, 147.8, 147.5, 137.5, 121.6, 102.1, 100.5, 93.4 (d, $J_{C–F}$ = 190.1 Hz), 86.9 (d, $J_{C–F}$ = 33.6 Hz), 80.9, 67.8 (d, $J_{C–F}$ = 16.1 Hz), 58.4, 49.5, 48.8, 36.6, 25.1, 24.9, 24.6, 24.1, 19.1, 19.0. ^{19}F NMR (471 MHz, $CDCl_3$): δ -201.74. HRMS (ESI): *m/z* $[M + H]^+$ calcd for $C_{22}H_{35}FN_5O_7^+$ 500.2515, found 500.2502.

N^6-Benzoyl-5´-(2-methoxyprop-2-yl)-2´-fluoroadenosine (2h).

The synthesis was carried out as described in general procedure by using N^6-benzoyl-2´-F-adenoside (0.66 g, 1.78 mmol), 2-methoxypropene (0.35 mL, 3.56 mmol), *p*TsOH · H_2O (35 mg, 0.18 mmol) and pyridine (3.2 mL). The reaction was monitored by RP HPLC using gradient B. The crude product was purified by silica gel column chromatography (DCM/MeOH/Pyridine, 95:4:1, *v*/*v*/*v*) to give **2h** as white solid (0.50 g, yield: 64%). ^{1}H NMR (500 MHz, $CDCl_3$): δ 9.22 (br s, 1H), 8.75 (s, 1H), 8.43 (s, 1H), 6.39 (dd, J = 15.9 and 2.0 Hz, 1H), 5.43–5.32 (m, 1H), 4.75–4.69 (m, 1H), 4.29–4.28 (m, 1H), 3.84 (dd, J = 11.1 and 2.3 Hz, 1H), 3.66 (dd, J = 11.2 and 3.3 Hz, 1H), 3.15 (s, 3H), 1.37 (s, 3H), 1.35 (s, 3H). ^{13}C{^{1}H}NMR (126 MHz, $CDCl_3$): δ 164.9, 152.9, 151.4, 151.4, 149.7, 141.6, 133.6, 133.0, 129.0, 128.0, 124.0, 123.4, 100.6, 94.1 (d, $J_{C–F}$ = 187.9 Hz), 86.8 (d, $J_{C–F}$ = 33.2 Hz), 82.7, 69.7 (d, $J_{C–F}$ = 16.7 Hz), 59.3, 48.8, 24.4. ^{19}F NMR (471 MHz, $CDCl_3$): δ -204.15. HRMS (ESI): *m/z* $[M + Na]^+$ calcd for $C_{21}H_{24}FN_5NaO_5^+$ 468.1654, found 468.1655.

N^6-Benzoyl-5´,3´-*O*-bis-(2-methoxyprop-2-yl)-2´-fluoroadenosine (4h).

An analytical sample of **4h** was purified by column chromatography (DCM/MeOH/Pyridine, 95:4:1, *v*/*v*/*v*) to give yellowish foam (conversion yield based on RP HPLC analysis: 20%). ^{1}H NMR (500 MHz, $CDCl_3$): δ 9.54 (s, 1H), 8.65 (s, 1H), 8.41 (s, 1H), 7.96–7.94 (m, 2H), 7.50–7.47 (m, 1H), 7.41–7.38 (m, 2H), 6.33 (dd, J = 16.2 and 0.9 Hz, 1H), 5.29–5.18 (m, 1H), 4.70–4.64 (m, 1H), 4.26–4.25 (m, 1H), 3.81 (dd, J = 11.2 and 2.1 Hz, 1H), 3.51 (dd, J = 11.2 and 3.1 Hz, 1H), 3.20 (s, 1H), 3.08 (s, 1H), 1.37 (s, 3H), 1.35 (s, 3H), 1.29 (s, 3H), 1.29 (s, 3H). ^{13}C{^{1}H}NMR (126 MHz, $CDCl_3$): δ 165.0, 152.6, 151.3, 149.7, 141.4, 133.5, 132.6, 128.6, 128.0, 123.7, 101.7, 100.3, 92.8 (d, $J_{C–F}$ = 191.2 Hz), 87.2 (d, $J_{C–F}$ = 34.1 Hz), 81.2, 67.8 (d, $J_{C–F}$ = 15.7 Hz), 58.6, 49.4, 38.6, 24.9, 24.8, 24.4, 24.1. ^{19}F NMR (471 MHz, $CDCl_3$): δ -201.36. HRMS (ESI): *m/z* $[M + H]^+$ calcd for $C_{25}H_{33}FN_5O_6^+$ 518.2409, found 518.2399.

N^6-Benzoyl-5´-*O*-(2-methoxyprop-2-yl)-2´-*O*-methylyadenosine (2i).

The synthesis was carried out as described in general procedure by using N^6-benzoyl-2´-*O*-methyladenosine (0.46 g, 1.20 mmol), 2-methoxypropene (0.24 mL, 2.40 mmol), *p*TsOH · H_2O (24 mg, 0.12 mmol) and pyridine (2.2 mL). The reaction was monitored by RP HPLC using gradient B. The crude product was purified by silica gel column chromatography (DCM/MeOH/Pyridine, 95:4:1, *v*/*v*/*v*) to give **2i** as white solid (388 mg, yield: 71%). ^{1}H NMR (500 MHz, $CDCl_3$): δ 9.17 (s, 1H), 8.77 (s, 1H), 8.50 (s, 1H), 8.01 (d, J = 7.6 Hz, 2H), 7.60–7.57 (m, 1H), 7.51–7.48 (m, 2H), 6.28 (d, J = 2.8 Hz, 1H), 4.50–4.47 (m, 1H), 4.21–4.19 (m, 1H), 4.14–4.13 (m, 1H), 3.83 (dd, J = 11.1 and 2.5 Hz, 1H), 3.67 (dd, J = 11.0 and 3.0 Hz, 1H), 3.60 (s, 3H), 3.19 (s, 3H), 3.05 (d, J = 6.9 Hz, 1H), 1.41 (s, 3H), 1.40 (s, 3H). ^{13}C{^{1}H}NMR (126 MHz, $CDCl_3$): δ 164.8, 152.8, 151.5, 149.6, 141.5, 133.7, 132.9, 128.9, 128.0, 123.5, 100.6, 86.6, 84.4, 83.8, 69.3, 59.5, 58.9, 48.8, 24.5, 24.5. HRMS (ESI): *m/z* $[M + Na]^+$ calcd for $C_{22}H_{27}N_5NaO_6^+$ 480.1854, found 480.1857.

N^4-Benzoyl-5´,3´-*O*-bis-(2-methoxyprop-2-yl)-2´-*O*-methyladenosine (4i).

An analytical sample of **4i** was purified by column chromatography (DCM/MeOH/Pyridine, 95:4:1, *v*/*v*/*v*) to give yellowish foam (conversion yield based on RP HPLC analysis: 8%). ^{1}H NMR (500 MHz, $CDCl_3$): δ 9.21 (s, 1H), 8.75 (s, 1H), 8.58 (s, 1H), 8.00 (d, J = 7.4 Hz, 2H), 7.58–7.55 (m, 1H), 7.49–7.43 (m, 1H), 6.30 (d, J = 1.6 Hz, 1H), 4.56–4.54 (m, 1H), 4.29–4.26 (m, 1H), 4.00 (dd, J = 4.9 and 1.7 Hz, 1H), 3.88 (dd, J = 11.0 and 2.6 Hz, 1H), 3.60 (s, 1H), 3.56 dd, J = 11.0 and 2.6 Hz, 1H), 3.24 (s, 3H), 3.17 (s, 3H), 1.40 (s, 3H), 1.39 (s, 3H), 1.38 (s, 3H), 1.37 (s, 3H). ^{13}C{^{1}H}NMR (126 MHz, $CDCl_3$): δ 164.8, 152.6, 151.4, 149.5, 141.5, 133.8, 132.8, 128.9, 128.0, 123.7, 101.7, 100.5, 86.8, 83.9, 81.5, 67.8, 58.7, 58.5, 49.3, 48.8, 25.1, 25.0, 24.6, 24.3. HRMS (ESI): *m/z* $[M + H]^+$ calcd for $C_{26}H_{36}N_5O_7^+$ 530.2609, found 530.2630.

N^6-Benzoyl-5´-*O*-(2-methoxyprop-2-yl)-2´-*O*-methoxyethyladenosine (2j).

The synthesis was carried out as described in general procedure by using N^6-benzoyl-2´-*O*-methoxyethyladenosine (0.39 g, 0.90 mmol), 2-methoxypropene (0.18 mL, 1.80 mmol), *p*TsOH · H_2O (18 mg, 0.09 mmol) and pyridine (1.6 mL). The reaction was monitored by RP HPLC using gradient B. The crude product was purified by silica gel column chromatography (DCM/MeOH/Pyridine, 95:4:1, *v*/*v*/*v*) to give **2j** as white solid (348 mg, yield: 77%). ^{1}H NMR (500 MHz, $CDCl_3$): δ 9.25 (s, 1H), 8.79 (s, 1H), 8.48 (s, 1H), 8.03 (d, J = 7.4 Hz, 2H), 7.61–7.58 (m, 1H), 7.52–7.49 (m, 2H), 6.28–6.27 (m, 1H), 4.46–4.45 (m, 2H), 4.29–4.27 (m, 1H), 4.01–3.98 (m, 1H), 3.87 (br s, 1H), 3.81 (dd, J = 11.0 and 2.7 Hz, 1H), 3.78–3.73 (m, 1H), 3.68 (dd, J = 11.0 and 3.2 Hz, 1H), 3.64–3.60 (m, 1H), 3.53–3.49 (m, 1H), 3.37 (s, 3H), 3.20 (s, 3H), 1.41 (s, 6H). ^{13}C{^{1}H}NMR (126 MHz, $CDCl_3$): δ 164.9, 152.7, 151.8, 149.6, 141.8, 133.7, 132.8, 128.9, 128.0, 123.6, 100.5, 87.2, 84.0, 83.4, 71.8, 70.5, 69.8, 60.1, 59.1, 48.8, 24.5. HRMS (ESI): *m/z* $[M + H]^+$ calcd for $C_{24}H_{32}N_5O_7^+$ 502.2296, found 502.2293.

N^6-Benzoyl-5´,3´-*O*-bis-(2-methoxyprop-2-yl)-2´-*O*-methoxyethyladenosine (4j).

An analytical sample of **4j** was purified by column chromatography (DCM/MeOH/Pyridine, 95:4:1, *v*/*v*/*v*) to give yellowish foam (conversion yield based on RP HPLC analysis: 20%). ^{1}H NMR (500 MHz, $CDCl_3$): δ 9.06 (s, 1H), 8.79 (s, 1H), 8.55 (s, 1H), 8.02 (d, J = 7.5 Hz, 2H), 7.61–7.58 (m, 1H), 7.53–7.49 (m, 2H), 6.32 (d, J = 2.5 Hz, 1H), 4.58–4.56 (m, 1H), 4.34–4.29 (m, 2H), 3.97–3.93 (m, 1H), 3.87 (dd, J = 11.0 and 2.7 Hz, 1H), 3.82–3.78 (m, 1H), 3.60–3.52 (m, 3H), 3.30 (s, 3H), 3.27 (s, 3H), 3.19 (s, 3H), 1.41 (s, 6H), 1.40 (s, 3H), 1.39 (s, 3H). ^{13}C{^{1}H}NMR (126 MHz, $CDCl_3$): δ 164.7, 152.8, 151.5, 149.5, 141.8, 133.8, 132.9, 129.0, 128.0, 123.6, 101.7, 100.5, 87.4, 82.8, 82.0, 72.1, 70.3, 68.3, 59.1, 59.1, 49.4, 48.8, 25.2, 25.1, 24.6, 24.4. HRMS (ESI): *m/z* $[M + H]^+$ calcd for $C_{28}H_{40}N_5O_8^+$ 574.2871, found 574.2876.

5´,3´:3´,5´-Di-*O*-isopropylidine-dithymidine (5) and 5´,5´:3´,3´-Di-*O*-isopropylidine-dithymidine (6)

2-Methoxypropene (1.22 mL, 12.4 mmol) was added to a mixture of thymidine (1.00 g, 4.13 mmol) and *p*TsOH·H_2O (81 mg, 0.41 mmol) in DMF (7.5 mL). Reaction was allowed to proceed for 4h. The reaction was monitored by RP HPLC using gradient A. Analytical samples of **5** and **6** were isolated by silica gel column chromatography (DCM/MeOH/Pyridine, 96:3:1 → 93:6:1, *v*/*v*/*v*) to give both as white solid. Conversion yields based on RP HPLC analysis: **5**; 28% and **6**; 15%. **5**: ^{1}H NMR (500 MHz, $CDCl_3$): δ 9.31 (s, 2H), 7.61 (m, 2H), 6.13 (dd, J = 7.9 and 2.3 Hz, 2H), 4.66–4.61 (m, 2H), 4.00–3.98 (m, 2H), 3.86 (dd, J = 11.1 and 1.5 Hz, 2H), 3.72 (dd, J = 9.8 and 1.2 Hz, 2H), 2.50–2.34 (m, 4H), 1.91 (m, 6H), 1.44 (s, 6H), 1.42 (s, 6H). ^{13}C{^{1}H}NMR (126 MHz, $CDCl_3$): δ 164.1, 150.4, 135.7, 110.3, 100.7, 84.6, 83.4, 65.8, 58.1, 42.2, 27.4, 25.3, 12.9. HRMS (ESI): *m/z* [M + Na]$^+$ calcd for $C_{26}H_{36}N_4NaO_{10}^+$ 587.23236, found 587.23440. **6**: ^{1}H NMR (500 MHz, $CDCl_3$): δ 9.02 (s, 2H), 7.72 (d, J = 0.9 Hz, 2H), 6.11 (dd, J = 6.9 and 2.3 Hz, 2H), 4.42–4.37 (m, 2H), 3.93–3.91 (m, 4H), 3.86–3.84 (m, 2H), 2.42–2.34 (m, 4H), 1.92 (s, 6H), 1.56 (s, 6H), 1.30 (s, 6H). ^{13}C{^{1}H}NMR (126 MHz, $CDCl_3$): δ 164.1, 150.4, 135.5, 110.4, 100.9, 85.0, 83.8, 65.5, 58.3, 42.1, 27.8, 25.5, 12.8. HRMS (ESI): *m/z* [M + Na]$^+$ calcd for $C_{26}H_{36}N_4NaO_{10}^+$ 587.2324, found 587.2338.

***N*^{6}-Benzoyl-5´-*O*-(2-methoxyprop-2-yl)-adenosine (2k).**

The synthesis was carried out as described in general procedure by using *N*^{6}-benzoyl-adenosine (394 mg, 1.06 mmol), 2-methoxypropene (0.21 mL, 2.1 mmol), *p*TsOH·H_2O (21 mg, 0.11 mmol) and pyridine (1.9 mL). The reaction was monitored by RP HPLC using gradient B. The crude product was purified by silica gel column chromatography (DCM/MeOH/Pyridine, 95:4:1 → 93:6:1, *v*/*v*/*v*) to give **2k** as white solid (0.23 g, yield: 50%). ^{1}H NMR (500 MHz, DMSO-d_6): δ 11.20 (s, 1H), 8.77 (s, 1H), 8.67 (s, 1H), 8.05 (d, J = 7.4 Hz, 2H), 7.66–7.63 (m, 1H), 7.57–7.54 (m, 2H), 6.07 (d, J = 5.0 Hz, 1H), 5.63 (d, J = 5.6 Hz, 1H), 5.31 (d, J = 4.9 Hz, 1H), 4.67–4.64 (m, 1H), 4.25–4.24 (m, 1H), 4.09–4.07 (m, 1H), 3.63 (dd, J =10.8 and 3.6 Hz, 1H), 3.53 (dd, J = 10.7 and 4.9 Hz, 1H), 3.04 (s, 3H), 1.28 (s, 6H). ^{13}C{^{1}H}NMR (126 MHz, DMSO-d_6): δ 165.6, 152.2, 151.7, 150.4, 143.2, 142.8, 133.3, 132.5, 128.5, 128.5, 125.8, 99.7, 87.6, 83.3, 73.5, 70.4, 60.6, 47.9, 24.2, 24.2. HRMS (ESI): *m/z* [M + H]$^+$ calcd for $C_{21}H_{26}N_5O_6^+$ 444.1878, found 444.1878.

Mixture of *N*^{6}-Benzoyl-5´,3´-*O*-bis-(2-methoxyprop-2-yl)-adenosine (4k) and *N*^{6}-Benzoyl-5´,2´-*O*-bis-(2-methoxyprop-2-yl)-adenosine (8).

An analytical sample as a mixture of **4k** and **8** was purified by column chromatography (DCM/MeOH/Pyridine, 95:4:1 → 93:6:1, *v*/*v*/*v*) to give yellowish foam (conversion yields based on RP HPLC analysis: **4k**; 16% and **8**; 16%). (cf. assignments in Figures 65 and 66). ^{1}H NMR (500 MHz, $CDCl_3$): δ 9.67 (br s, 2H), 8.66 (s, 1H), 8.61 (s, 1H), 8.39 (s, 1H), 8.34 (s, 1H), 7.93 (d, J = 7.4 Hz, 4H), 7.46–7.43 (m, 2H), 7.37–7.34 (m, 4H), 6.21 (d, J = 5.6 Hz, 1H), 6.11 (d, J = 3.8 Hz, 1H), 4.81 (dd, J = 5.3 and 5.3 Hz, 1H), 4.56–4.52 (m, 2H), 4.29–4.27 (m,

1H), 4.24–4.20 (m, 2H), 3.68 (dd, *J* = 11.0 and 2.8 Hz, 1H), 3.64 (dd, *J* = 10.9 and 2.7 Hz, 1H), 3.55 (dd, *J* = 10.8 and 3.1 Hz, 1H), 3.50 (dd, *J* = 10.9 and 3.4 Hz, 1H), 3.18 (s, 3H), 3.08 (s, 3H), 3.05 (s, 3H), 2.75 (s, 3H), 1.33 (s, 6H), 1.31–1.26 (m, 15H), 1.12 (s, 3H). $^{13}C\{^1H\}$NMR (126 MHz, $CDCl_3$): δ 165.0, 152.5, 152.3, 152.0, 151.6, 149.5, 141.6, 141.5, 133.6, 132.5, 132.5, 128.5, 128.5, 127.9, 127.9, 123.5, 123.2, 101.8, 101.8, 100.2, 100.2, 89.0, 86.6, 84.3, 82.5, 74.7, 74.4, 71.3, 69.8, 60.7, 59.7, 49.4, 48.9, 48.6, 48.5, 25.0, 24.9, 24.8, 24.4, 24.3, 24.2, 24.1. HRMS (ESI): *m/z* $[M + H]^+$ calcd for $C_{25}H_{34}N_5O_7^+$ 516.2453, found 516.2451.

NMR spectra of acetalized nucleosides

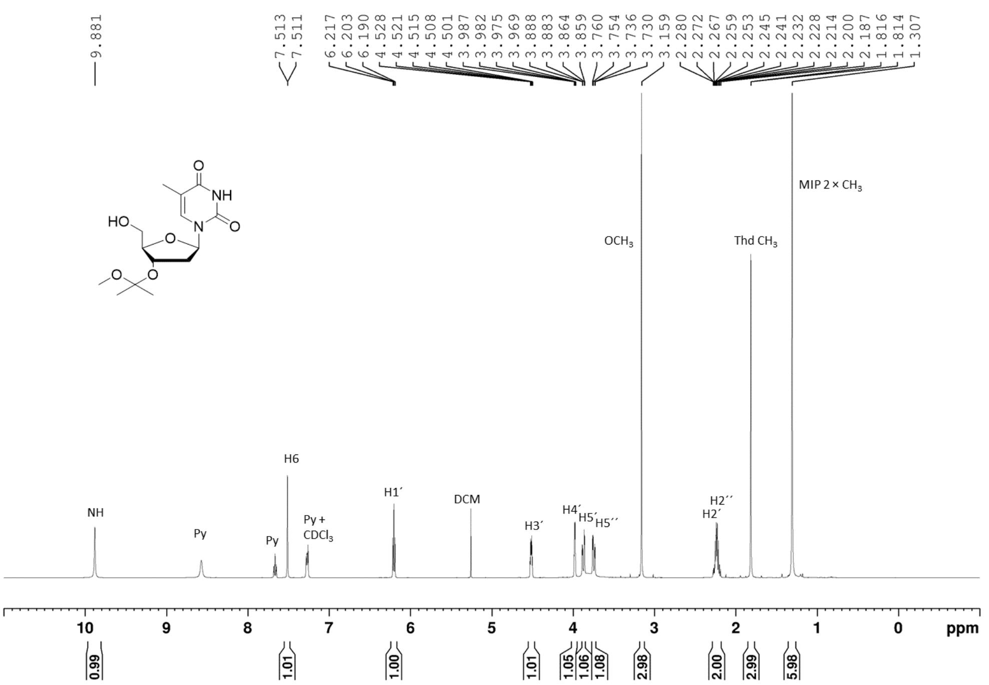

Figure S17. ^{1}H NMR (500 MHz, $CDCl_3$) spectrum of **3a**

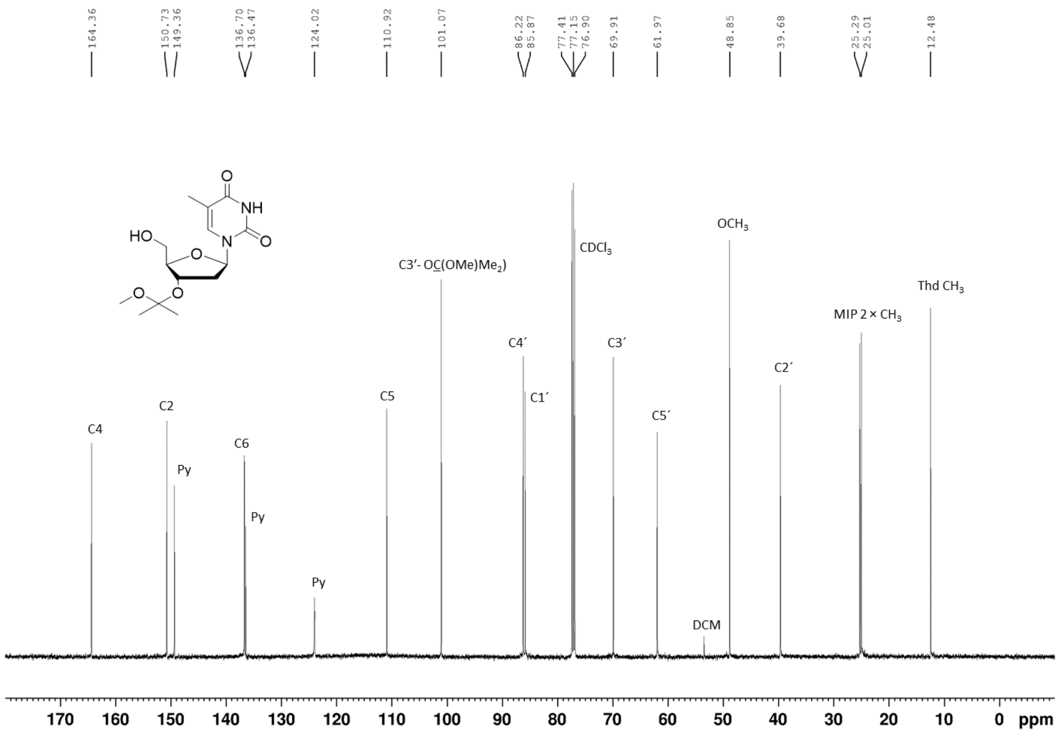

Figure S18. ^{13}C NMR (126 MHz, CDCl$_3$) spectrum of **3a**

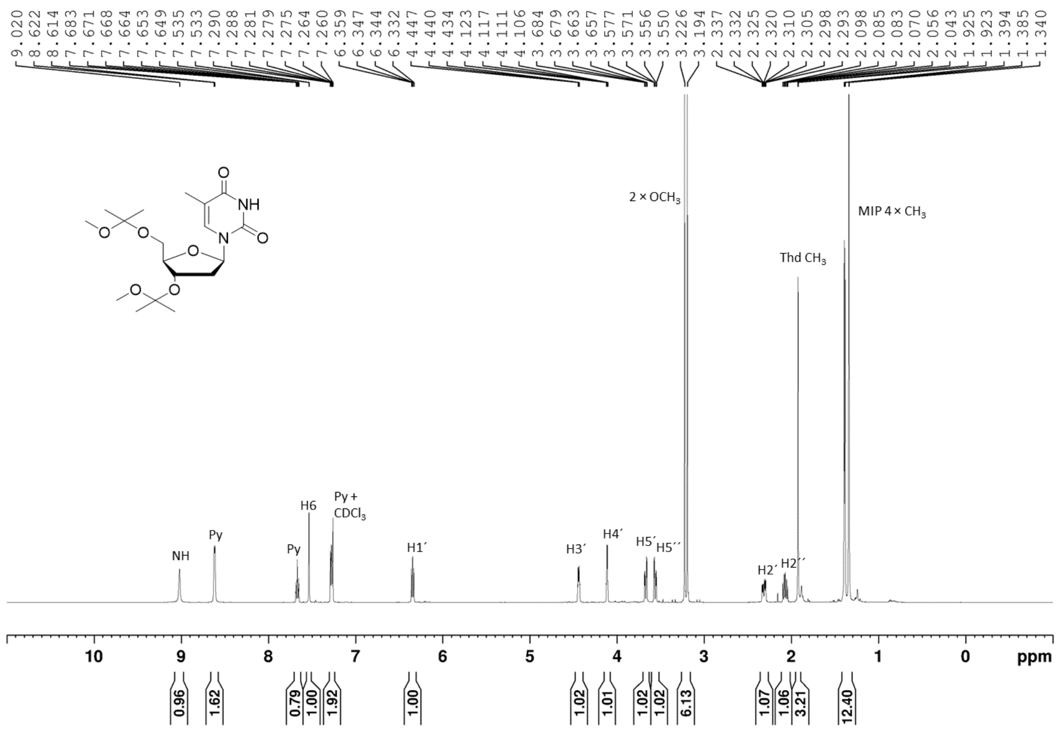

Figure S19. ^{1}H NMR (500 MHz, CDCl$_3$) spectrum of **4a**

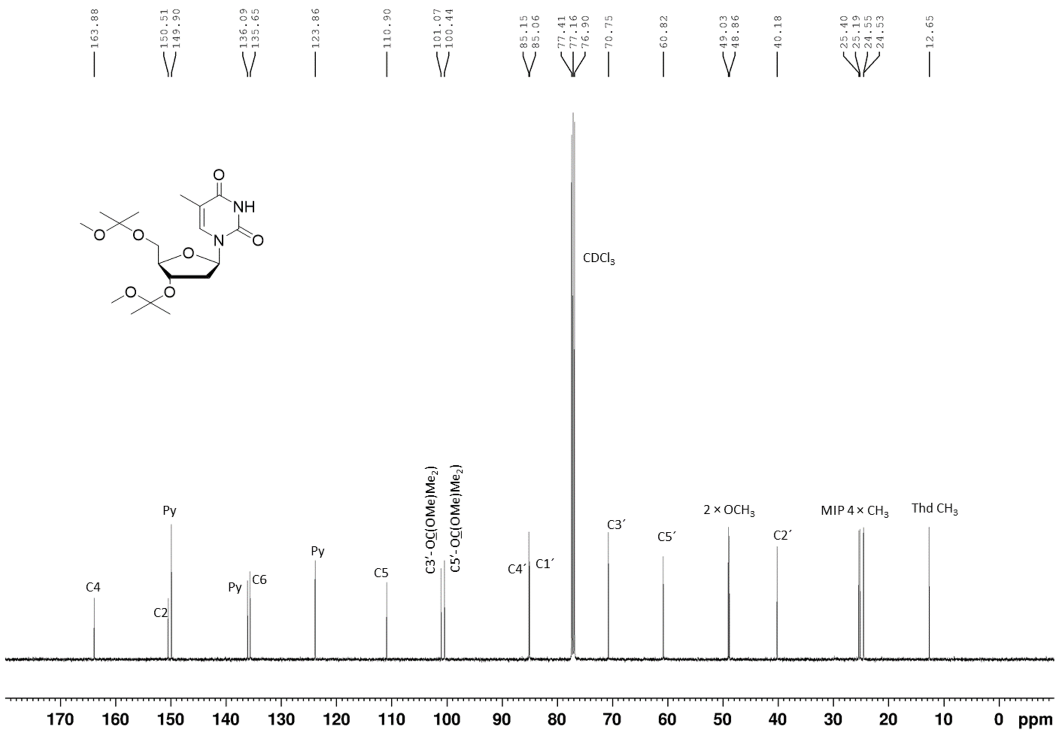

Figure S20. ^{13}C NMR (126 MHz, $CDCl_3$) spectrum of **4a**

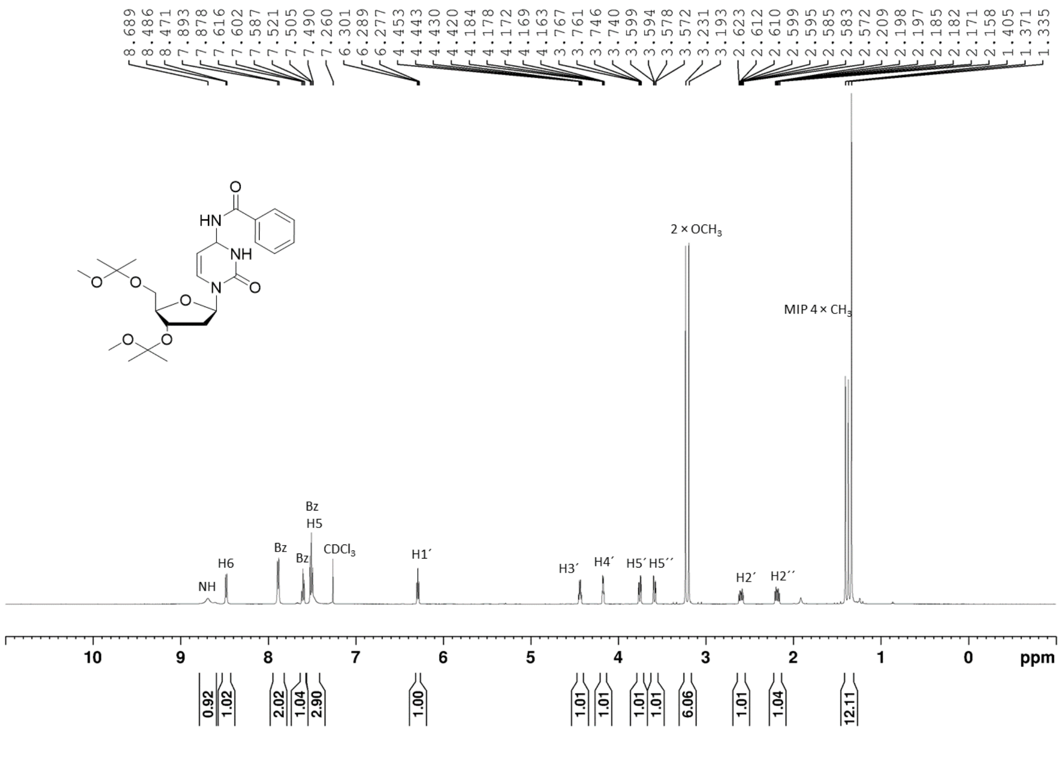

Figure S21. ^{1}H NMR (500 MHz, $CDCl_3$) spectrum of **4b**

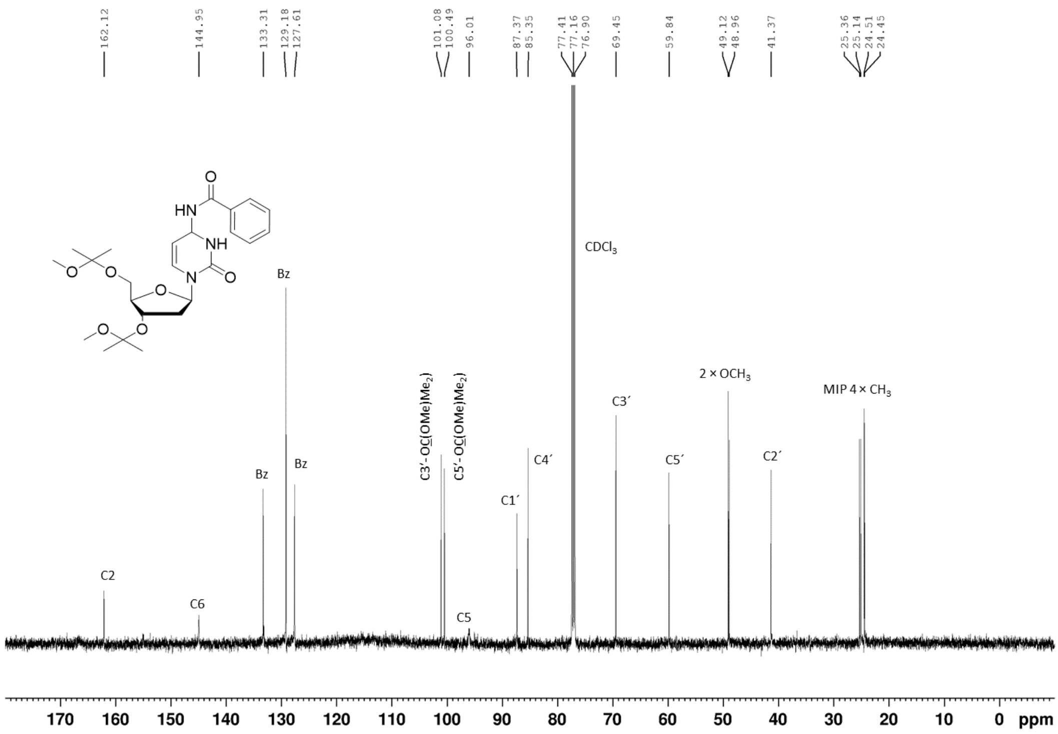

Figure S22. ^{13}C NMR (126 MHz, $CDCl_3$) spectrum of **4b**

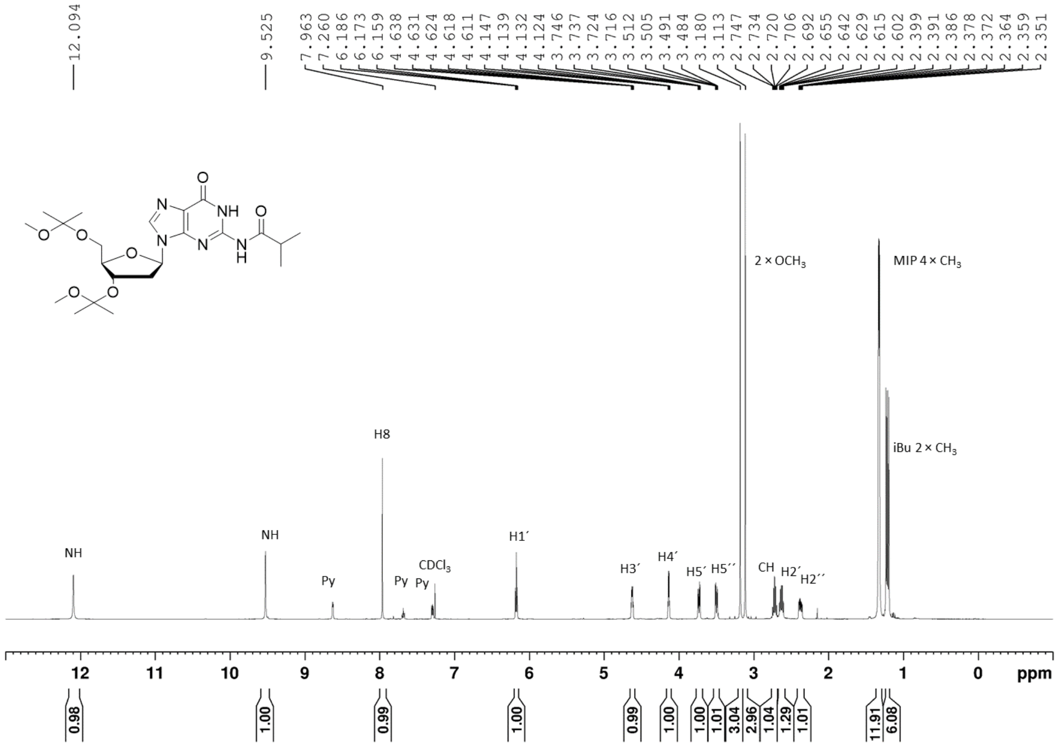

Figure S23. ^{1}H NMR (500 MHz, $CDCl_3$) spectrum of **4c**

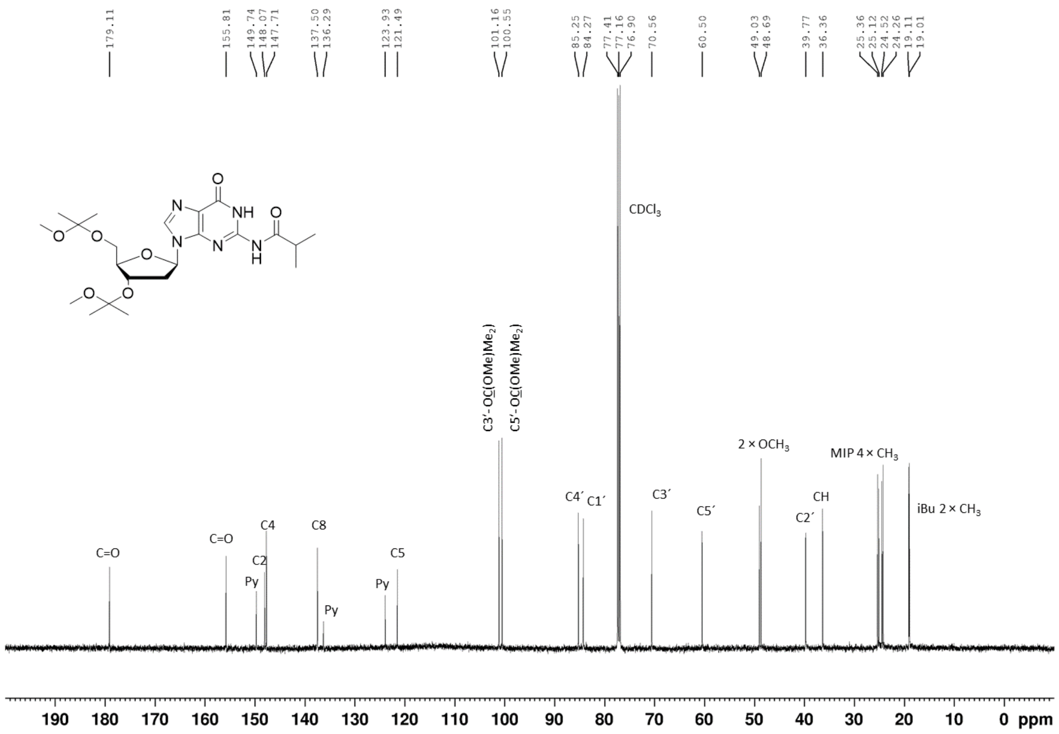

Figure S24. ^{13}C NMR (126 MHz, $CDCl_3$) spectrum of **4c**

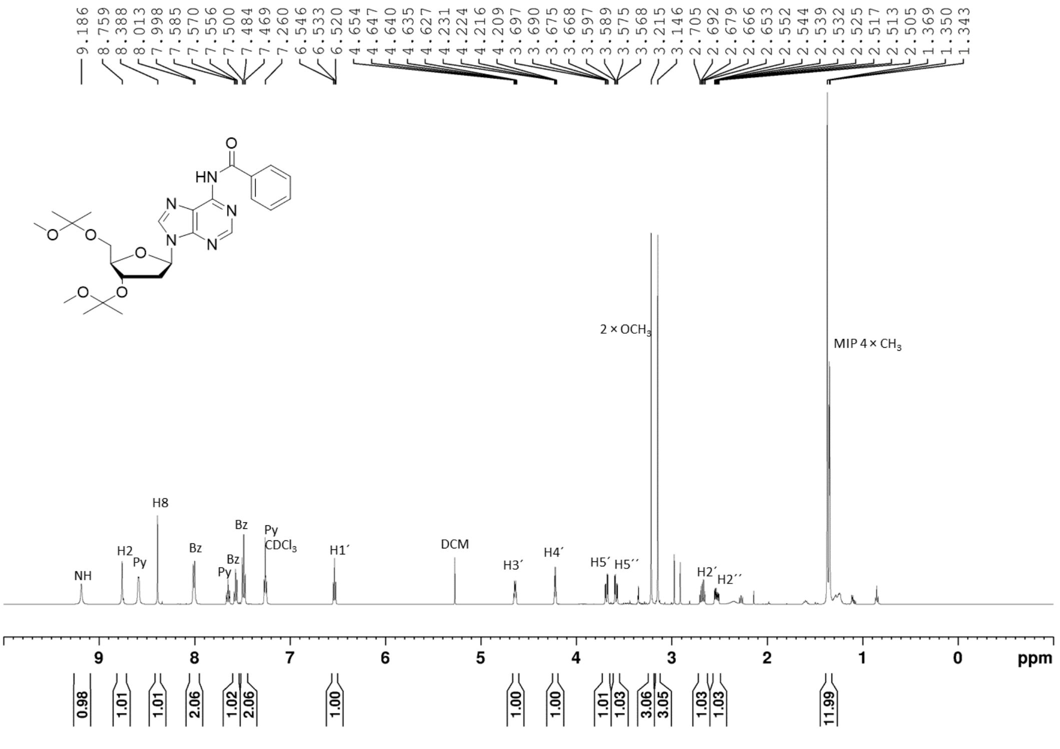

Figure S25. ^{1}H NMR (500 MHz, $CDCl_3$) spectrum of **4d**

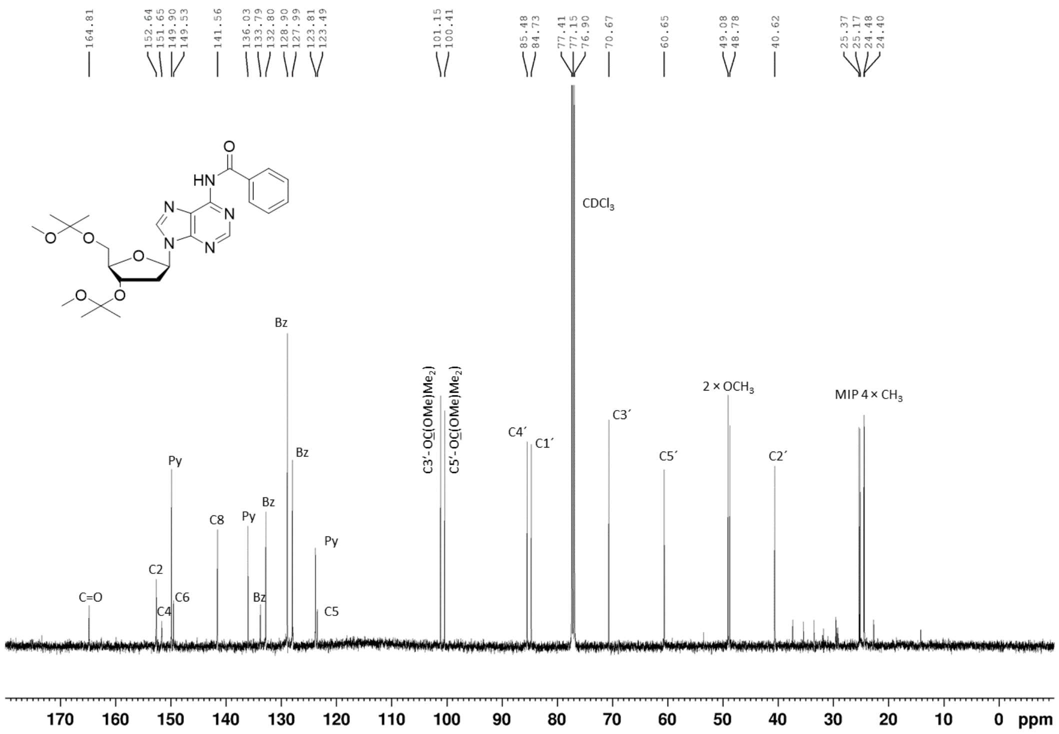

Figure S26. ^{13}C NMR (126 MHz, $CDCl_3$) spectrum of **4d**

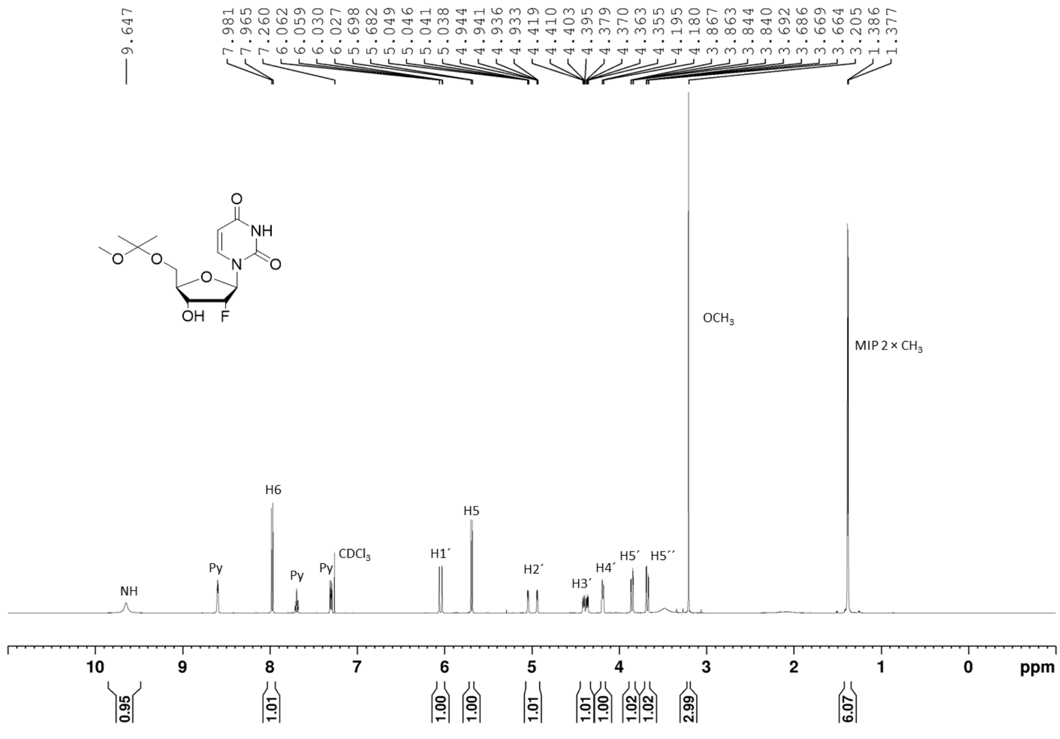

Figure S27. ^{1}H NMR (500 MHz, $CDCl_3$) spectrum of **2e**

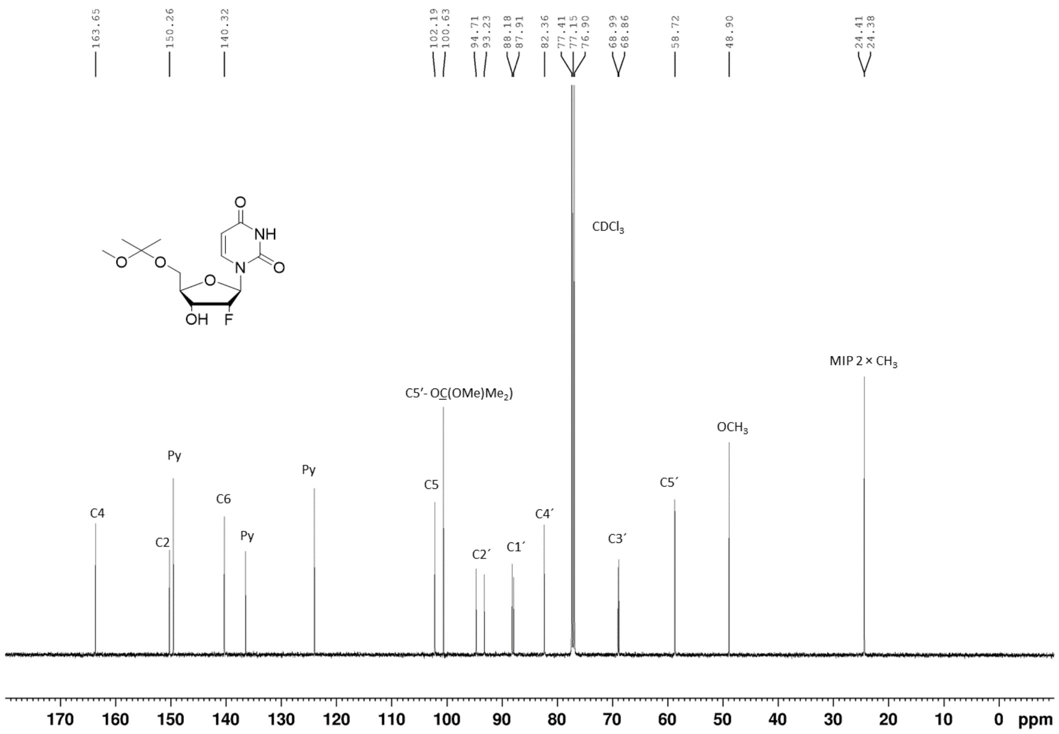

Figure S28. ^{13}C NMR (126 MHz, $CDCl_3$) spectrum of **2e**

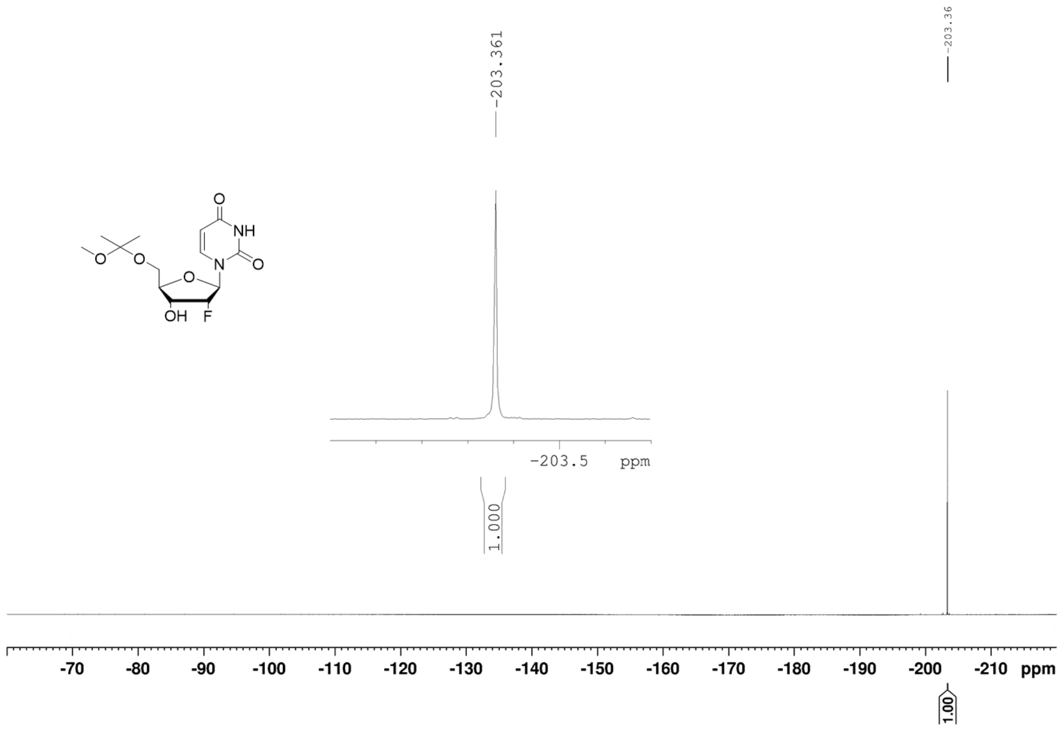

Figure S29. ^{19}F NMR (471 MHz, $CDCl_3$) spectrum of **2e**

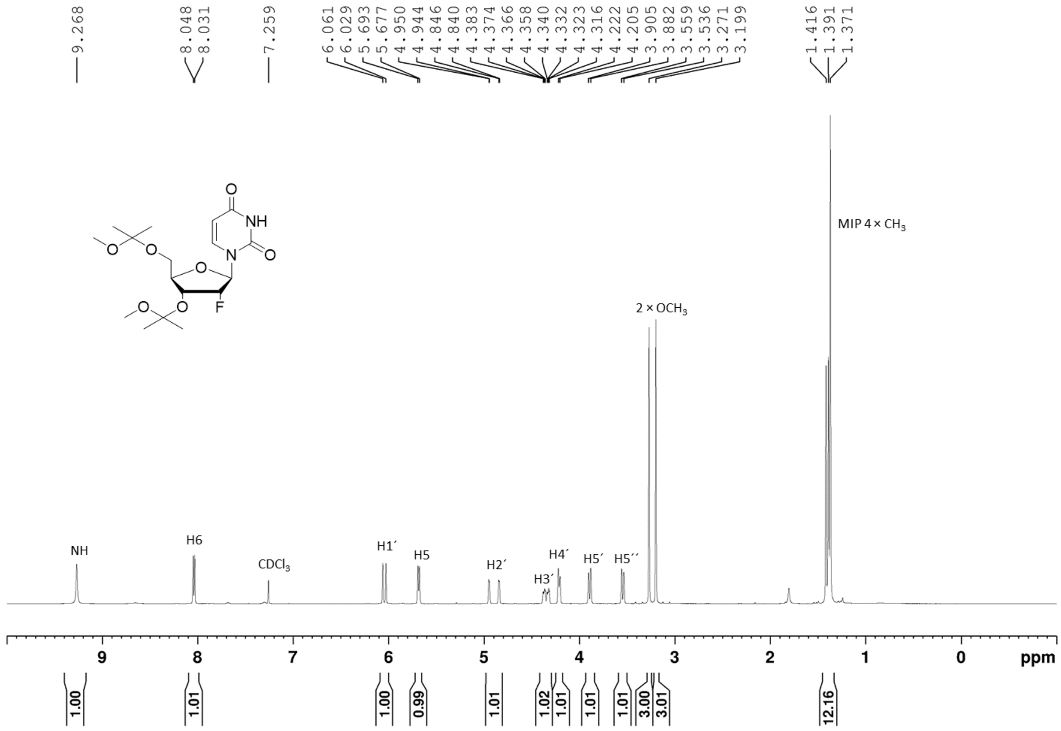

Figure S30. ^{1}H NMR (500 MHz, $CDCl_3$) spectrum of **4e**

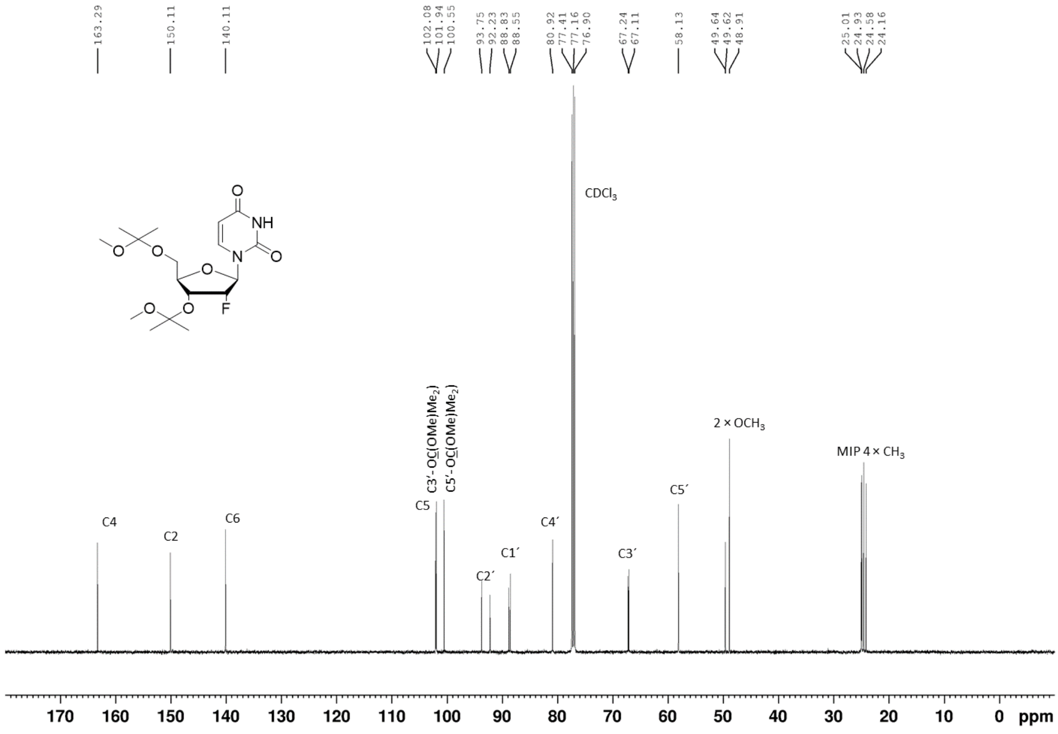

Figure S31. ^{13}C NMR (126 MHz, $CDCl_3$) spectrum of **4e**

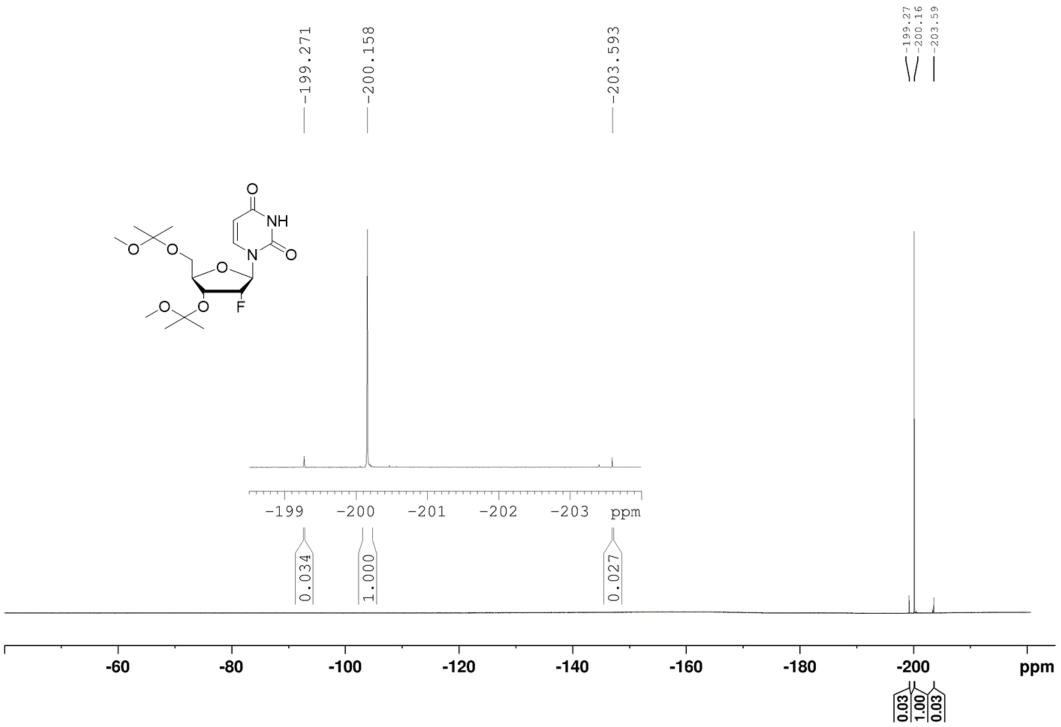

Figure S32. ^{19}F NMR (471 MHz, $CDCl_3$) spectrum of **4e**

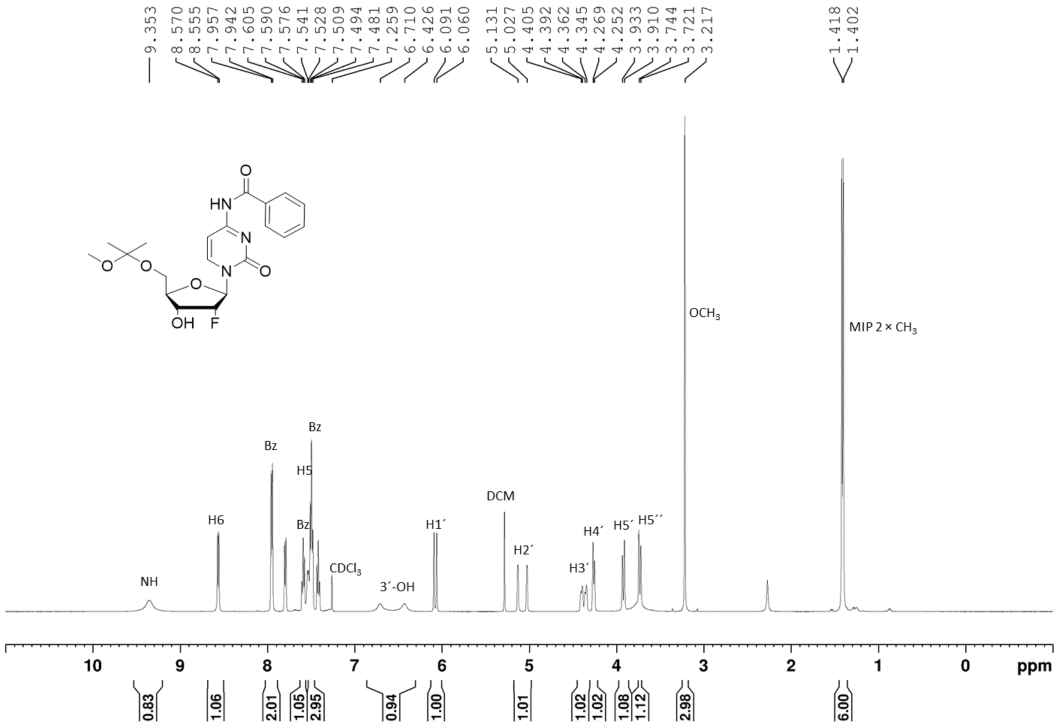

Figure S33. ^{1}H NMR (500 MHz, $CDCl_3$) spectrum of **2f**

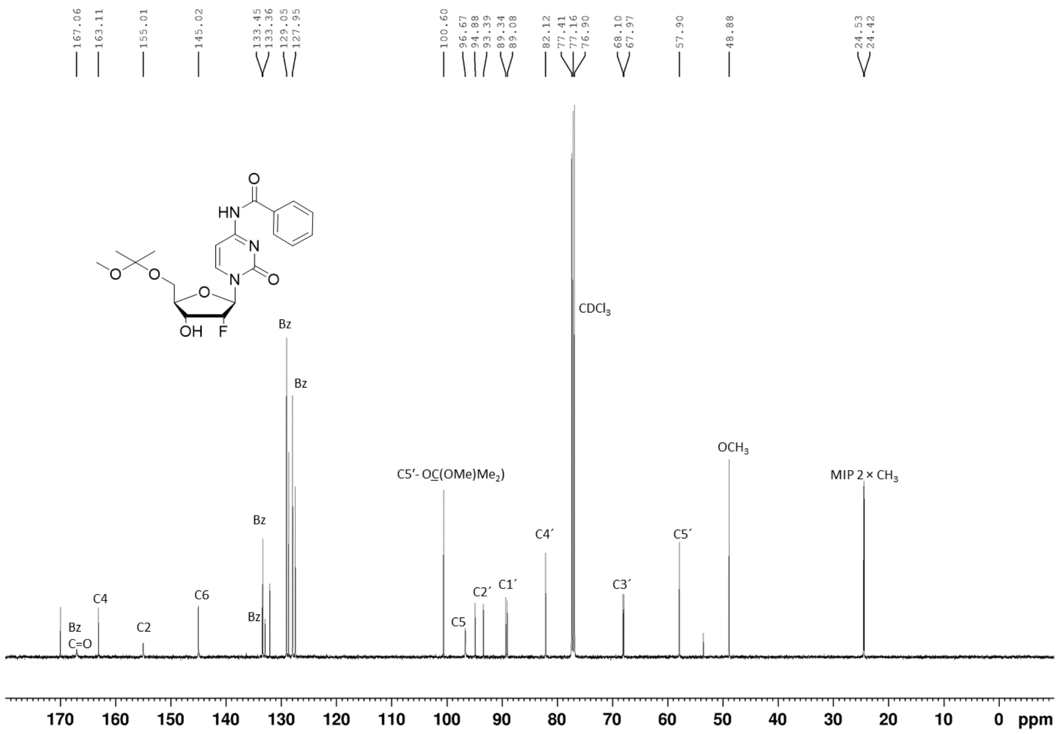

Figure S34. ^{13}C NMR (126 MHz, $CDCl_3$) spectrum of **2f**

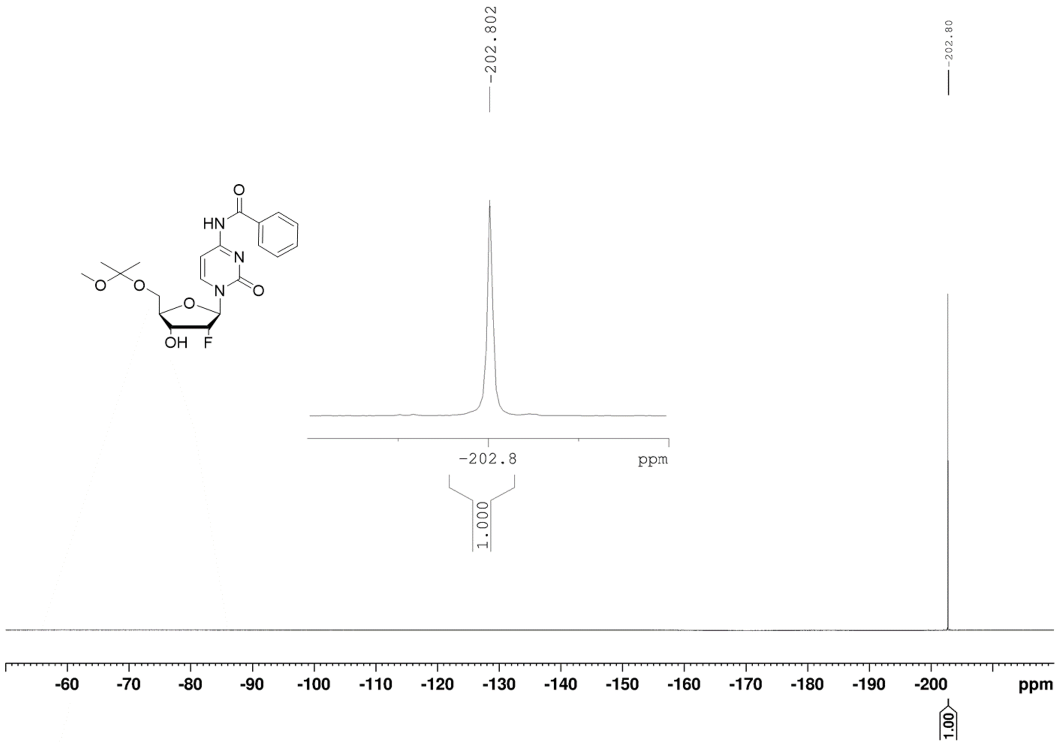

Figure S35. ^{19}F NMR (471 MHz, $CDCl_3$) spectrum of **2f**

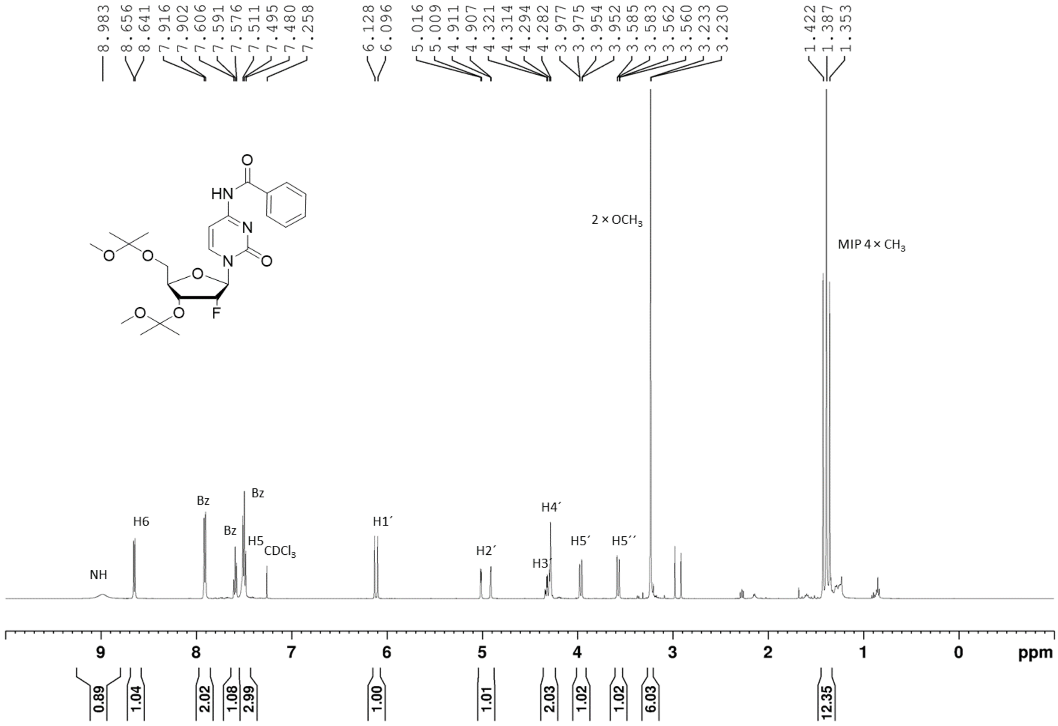

Figure S36. ^{1}H NMR (500 MHz, $CDCl_3$) spectrum of **4f**

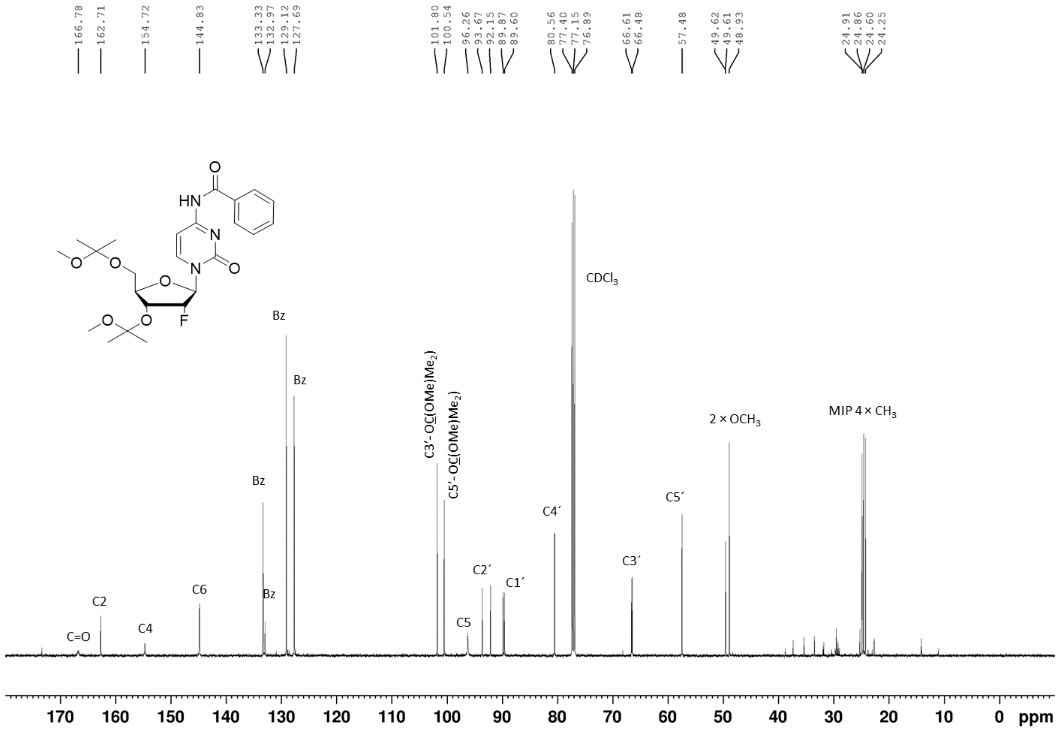

Figure S37. ^{13}C NMR (126 MHz, $CDCl_3$) spectrum of **4f**

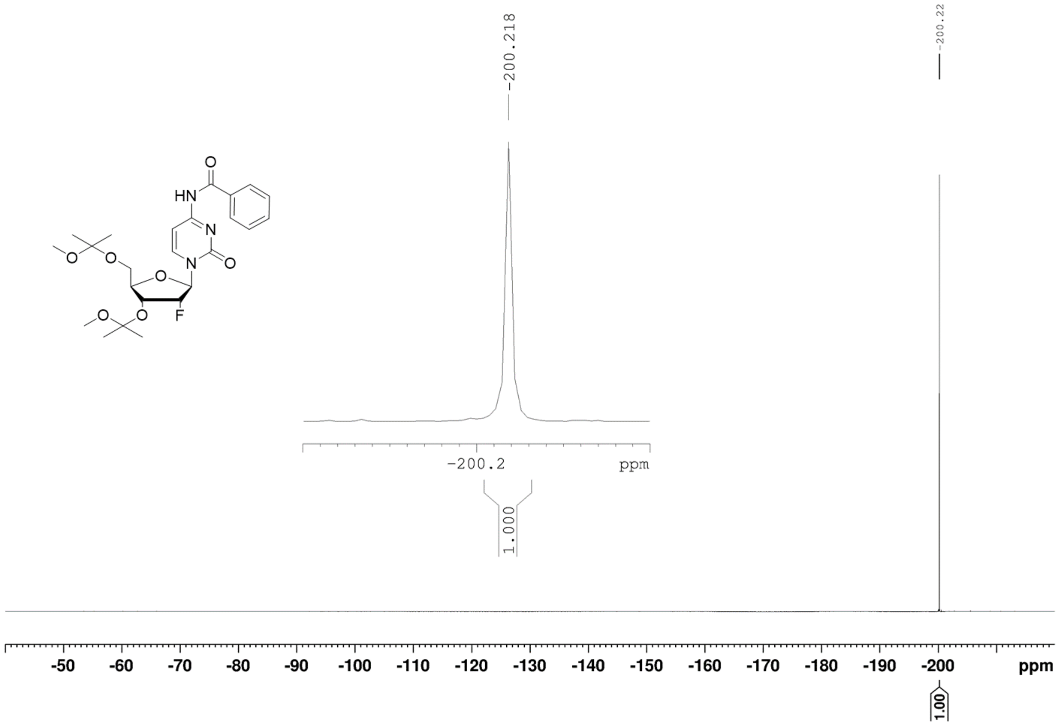

Figure S38. ^{19}F NMR (471 MHz, $CDCl_3$) spectrum of **4f**

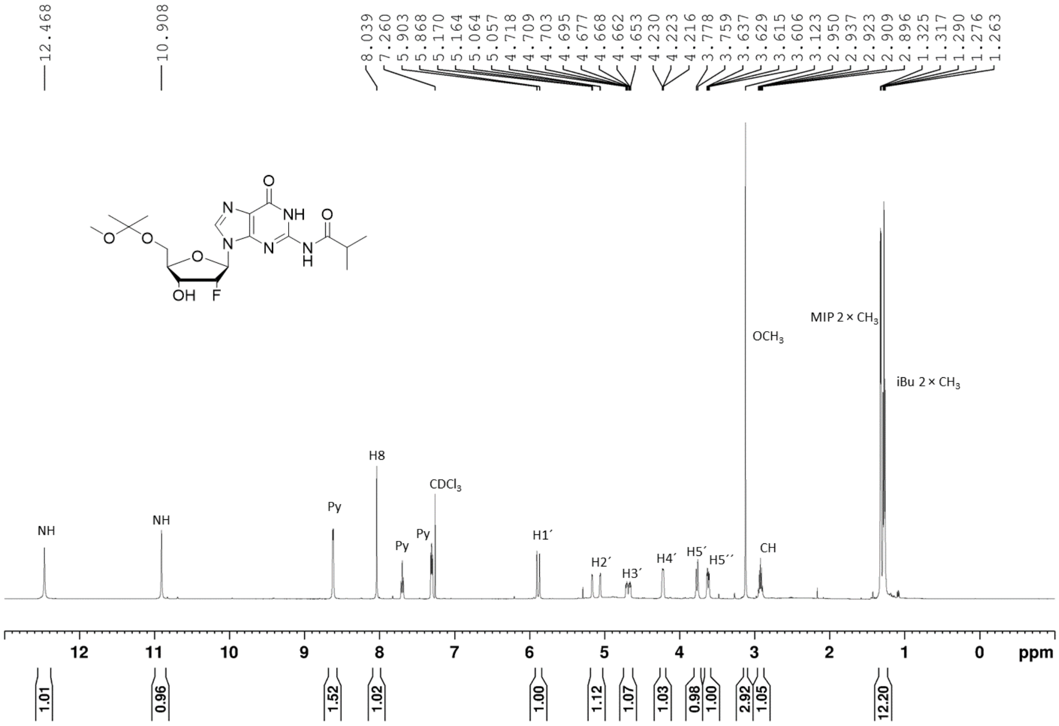

Figure S39. ^{1}H NMR (500 MHz, $CDCl_3$) spectrum of **2g**

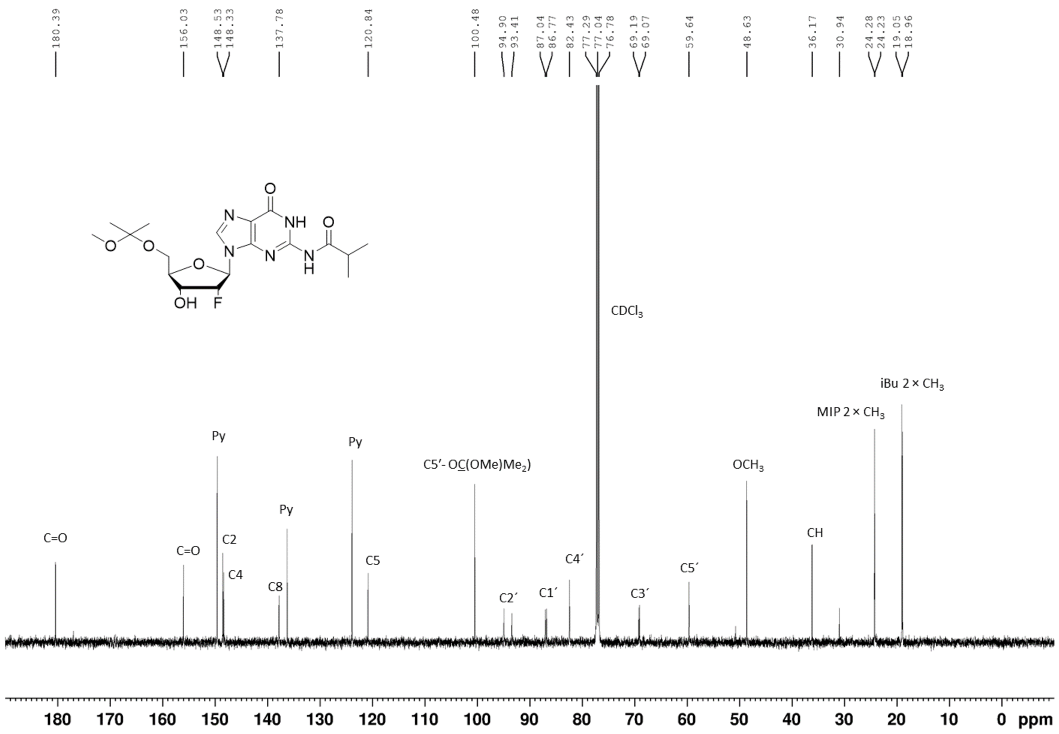

Figure S40. ^{13}C NMR (126 MHz, $CDCl_3$) spectrum of **2g**

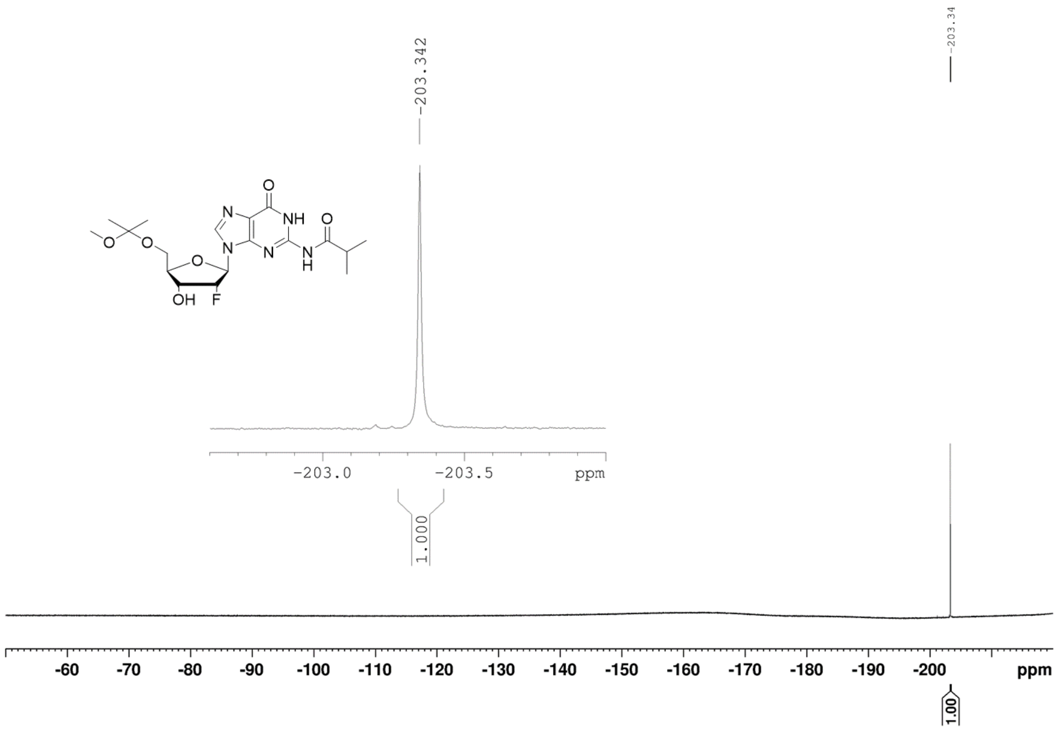

Figure S41. ^{19}F NMR (471 MHz, $CDCl_3$) spectrum of **2g**

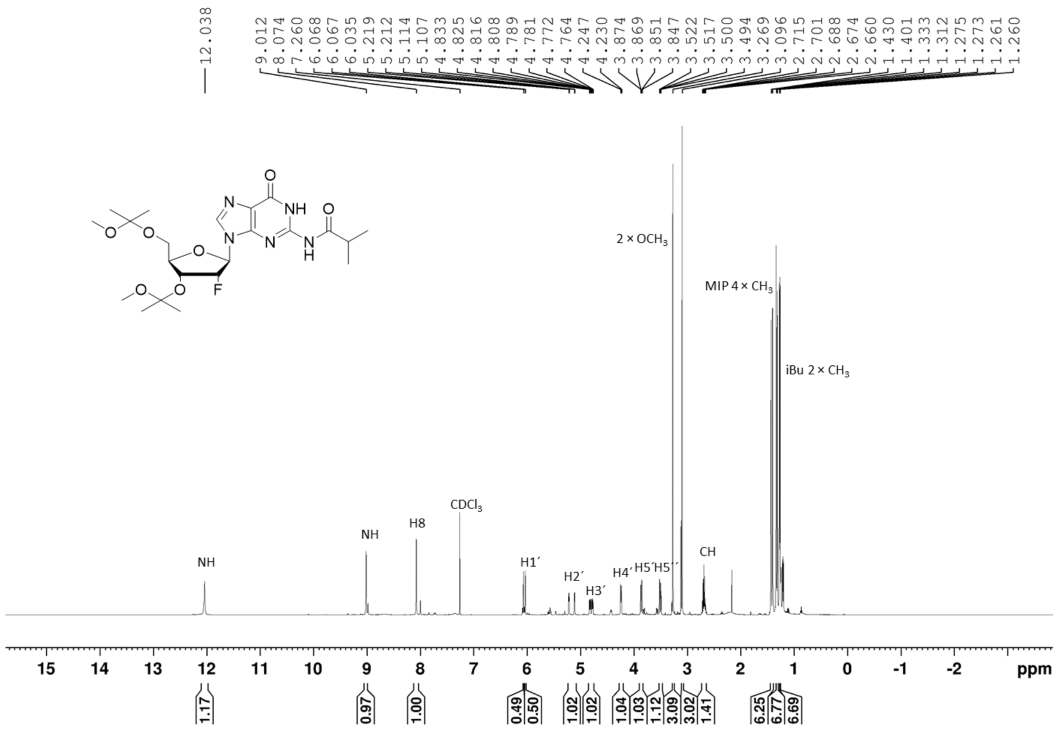

Figure S42. ^{1}H NMR (500 MHz, $CDCl_3$) spectrum of **4g**

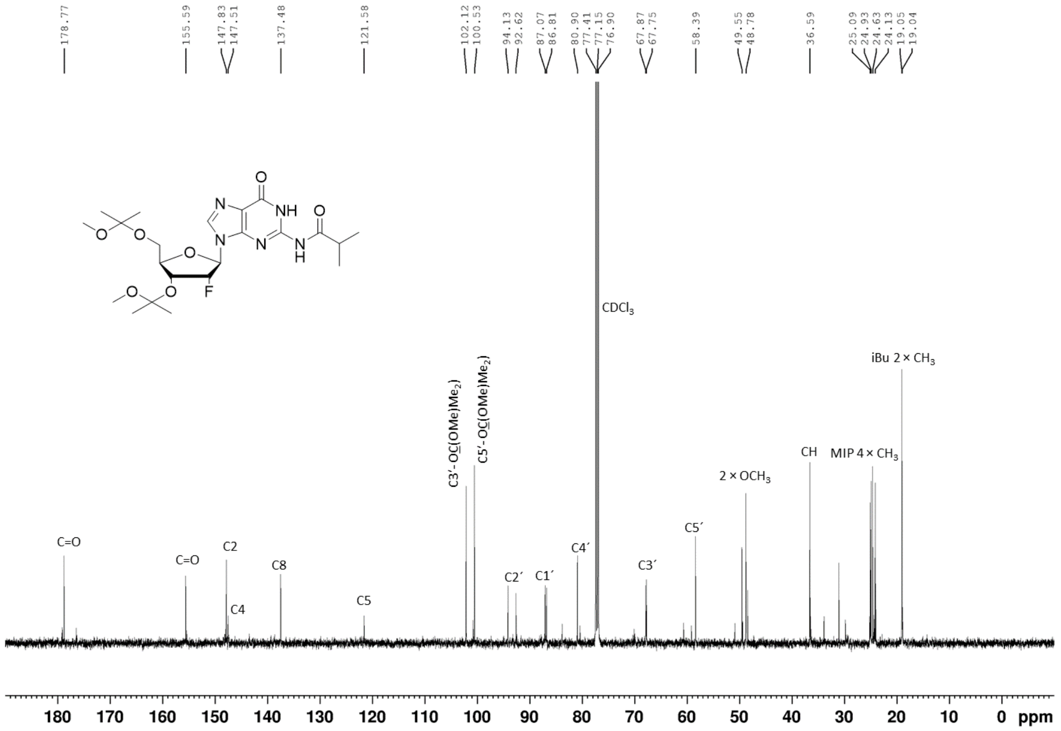

Figure S43. ^{13}C NMR (126 MHz, $CDCl_3$) spectrum of **4g**

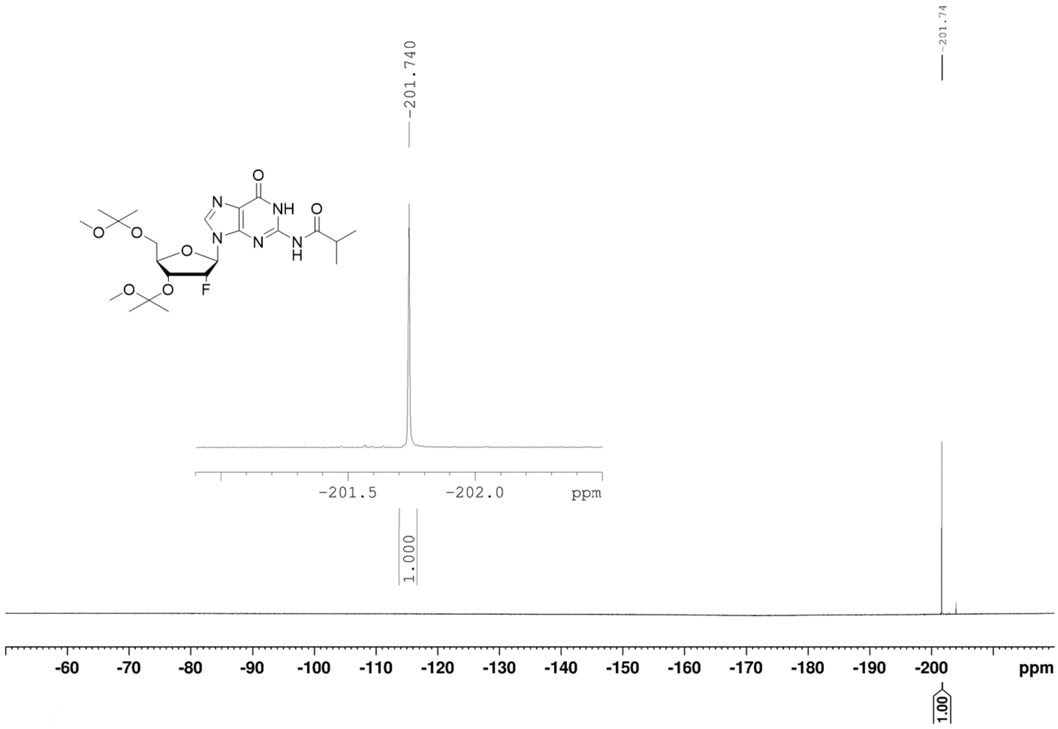

Figure S44. ^{19}F NMR (471 MHz, $CDCl_3$) spectrum of **4g**

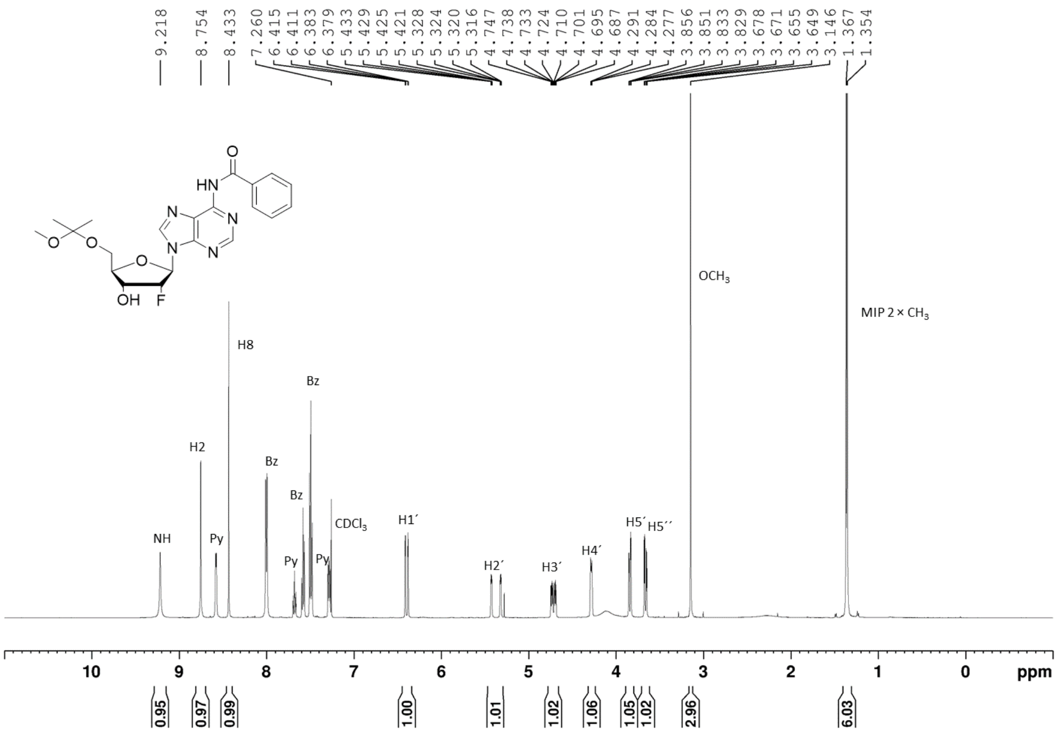

Figure S45. ^{1}H NMR (500 MHz, $CDCl_3$) spectrum of **2h**

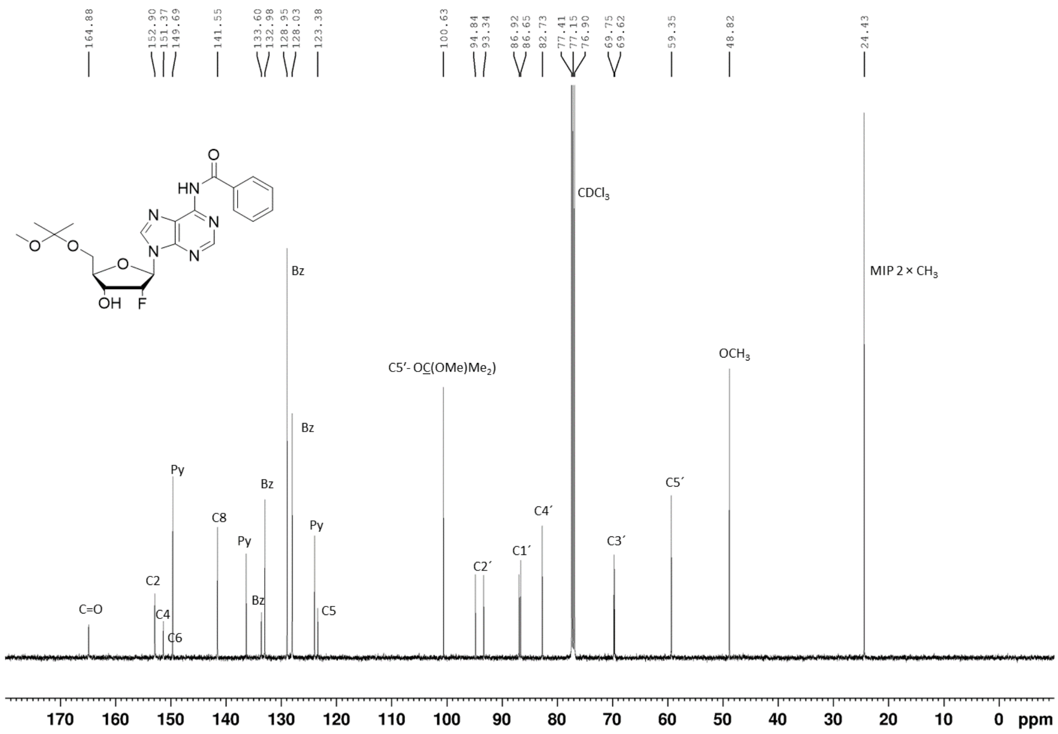

Figure S46. ^{13}C NMR (126 MHz, $CDCl_3$) spectrum of **2h**

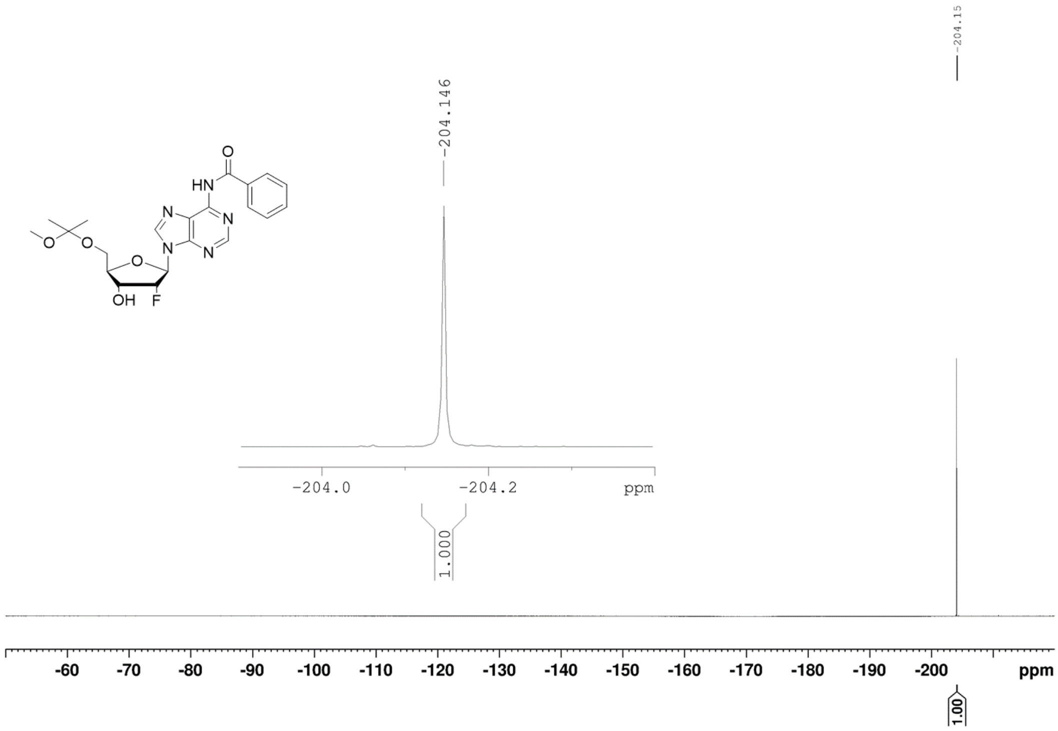

Figure S47. ^{19}F NMR (471 MHz, $CDCl_3$) spectrum of **2h**

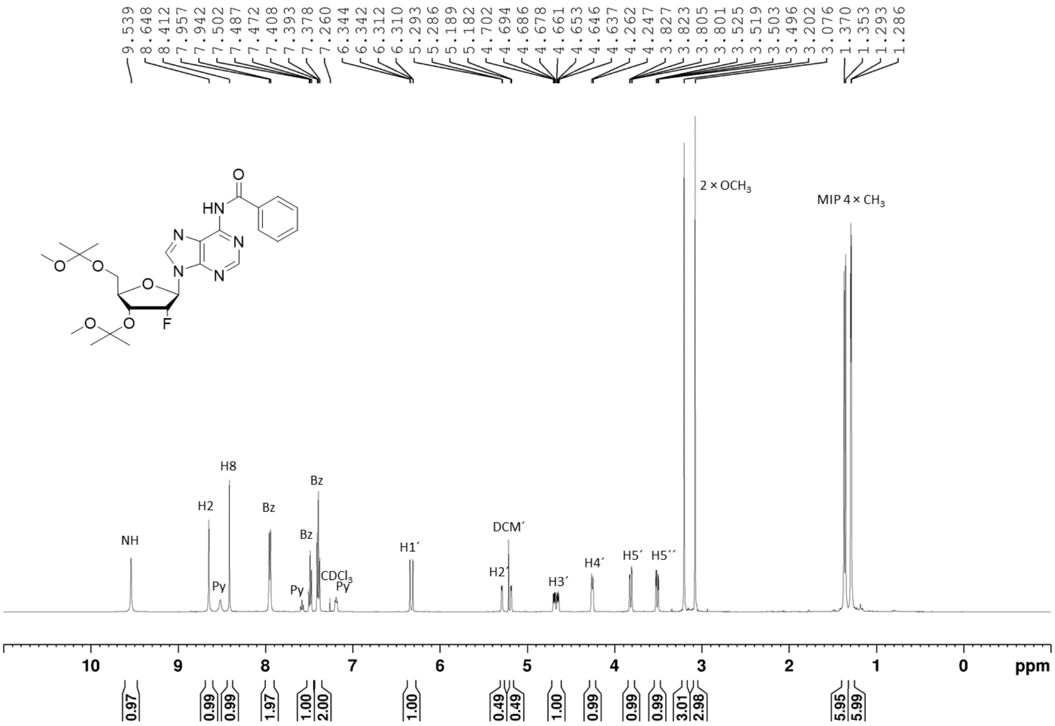

Figure S48. ^{1}H NMR (500 MHz, $CDCl_3$) spectrum of **4h**

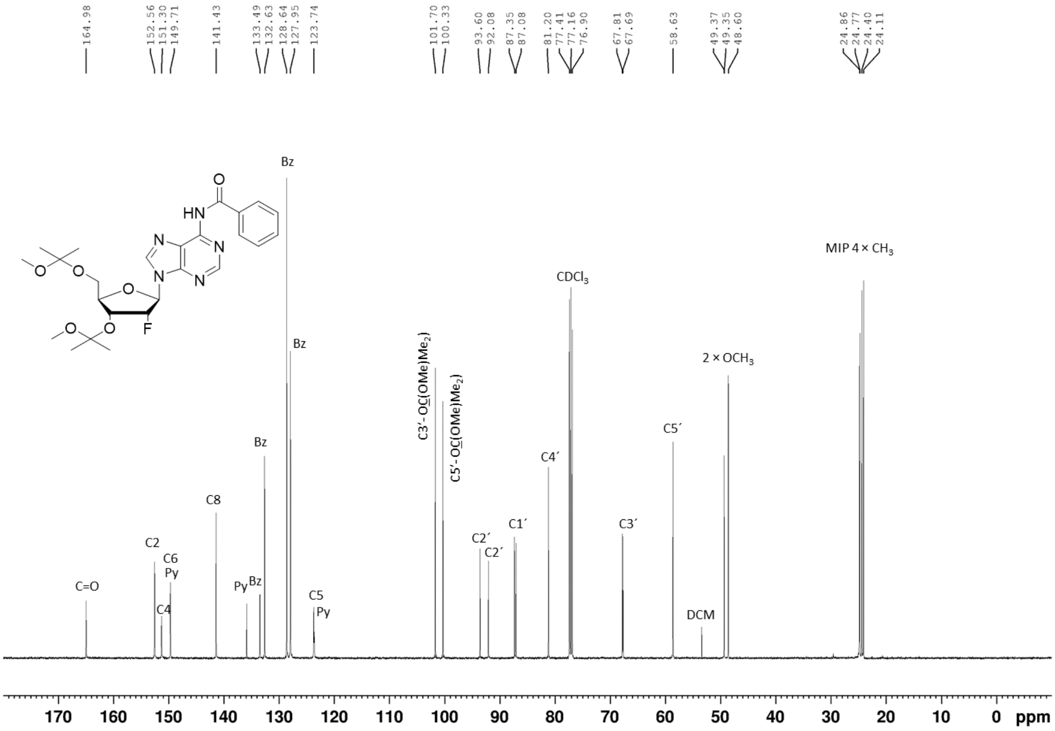

Figure S49. ^{13}C NMR (126 MHz, $CDCl_3$) spectrum of **4h**

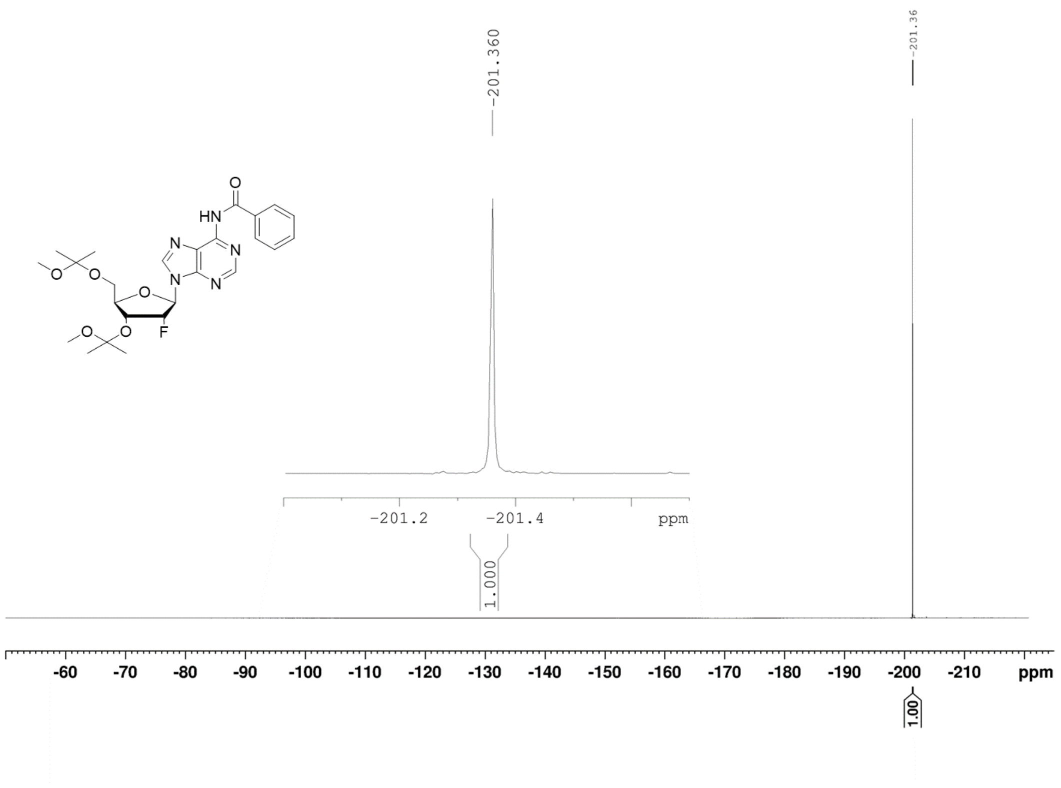

Figure S50. ^{19}F NMR (471 MHz, $CDCl_3$) spectrum of **4h**

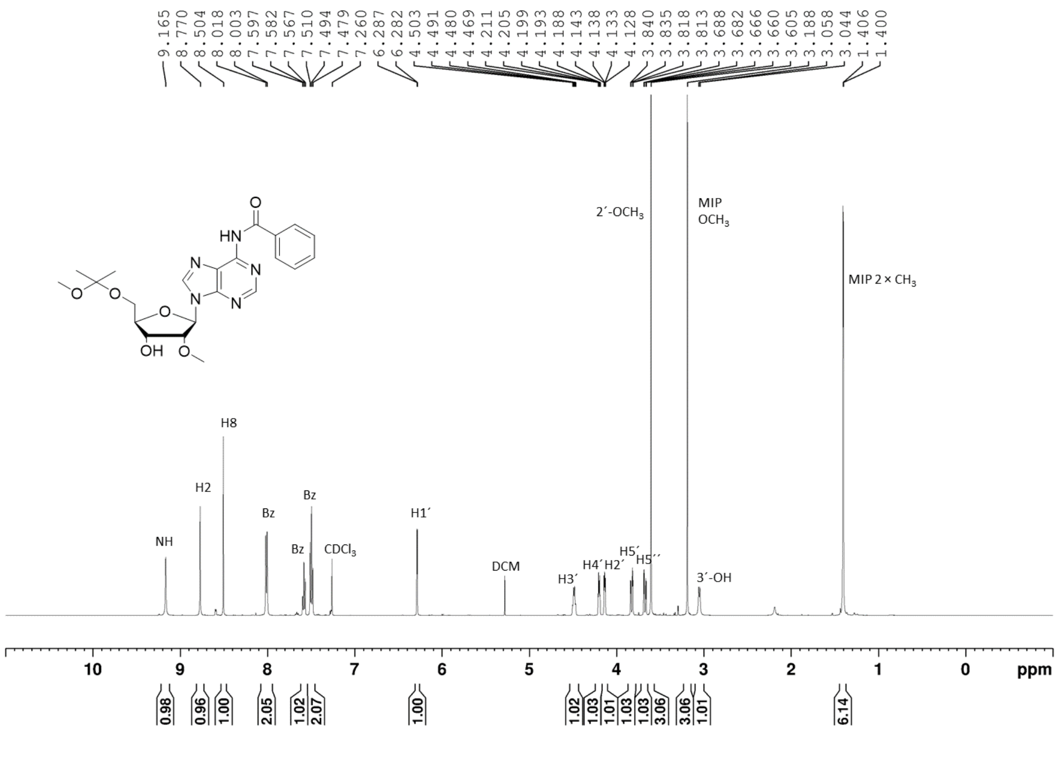

Figure S51. ^{1}H NMR (500 MHz, $CDCl_3$) spectrum of **2i**

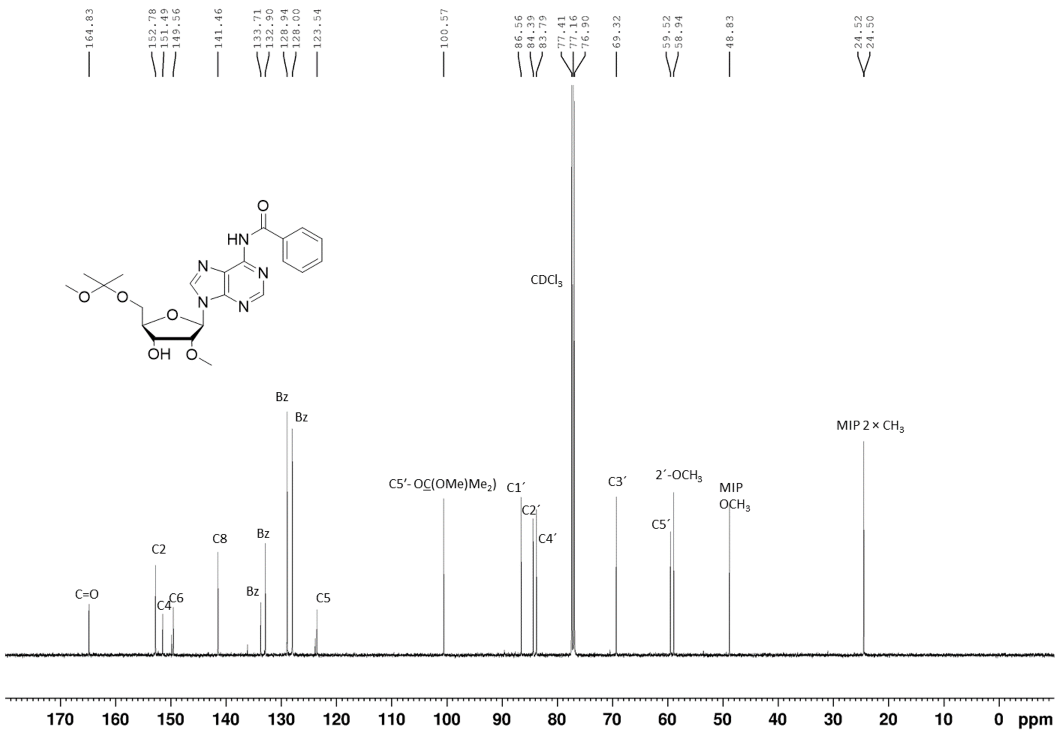

Figure S52. ^{13}C NMR (126 MHz, $CDCl_3$) spectrum of 2**i**

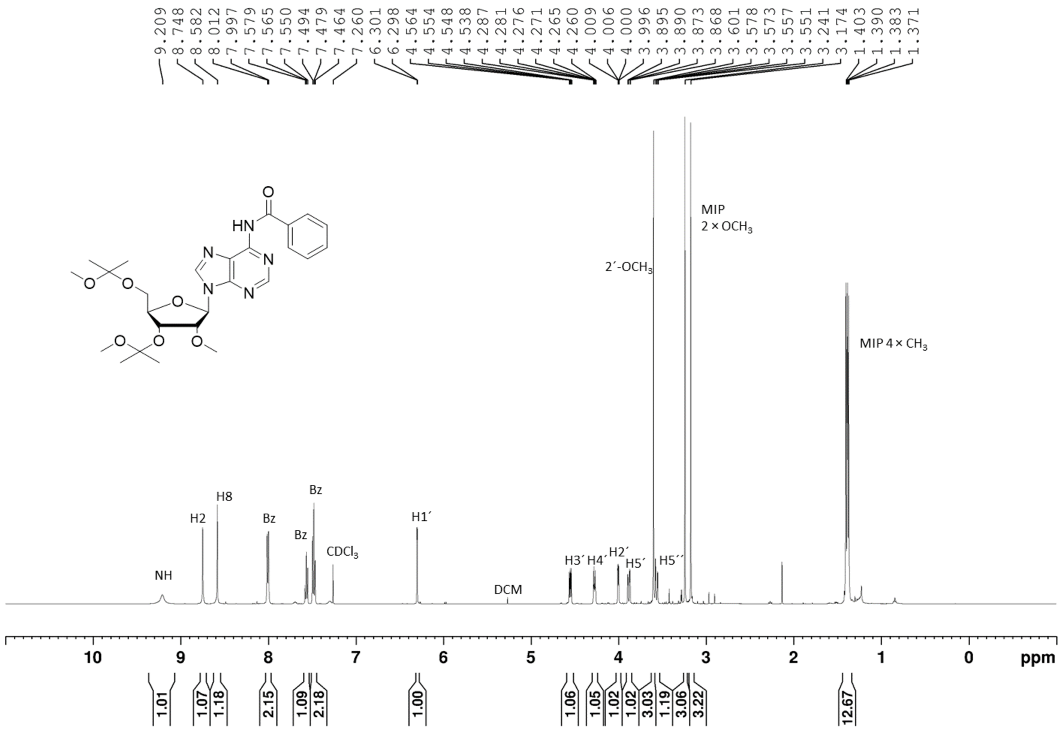

Figure S53. ^{1}H NMR (500 MHz, $CDCl_3$) spectrum of **4i**

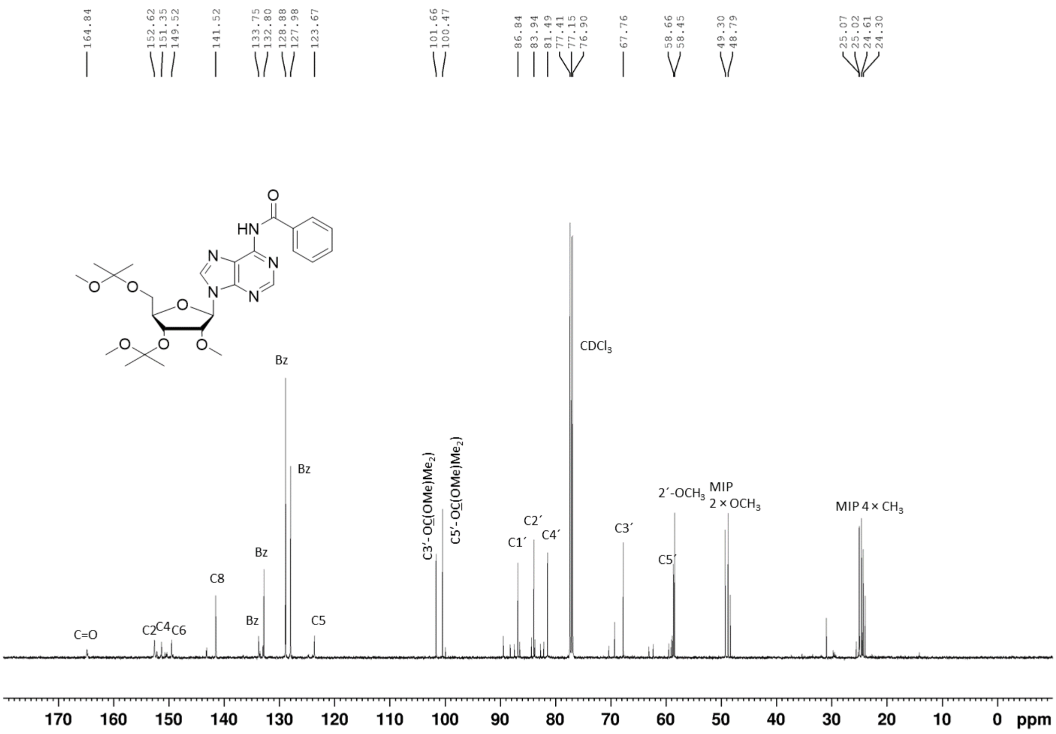

Figure S54. ^{13}C NMR (126 MHz, $CDCl_3$) spectrum of **4i**

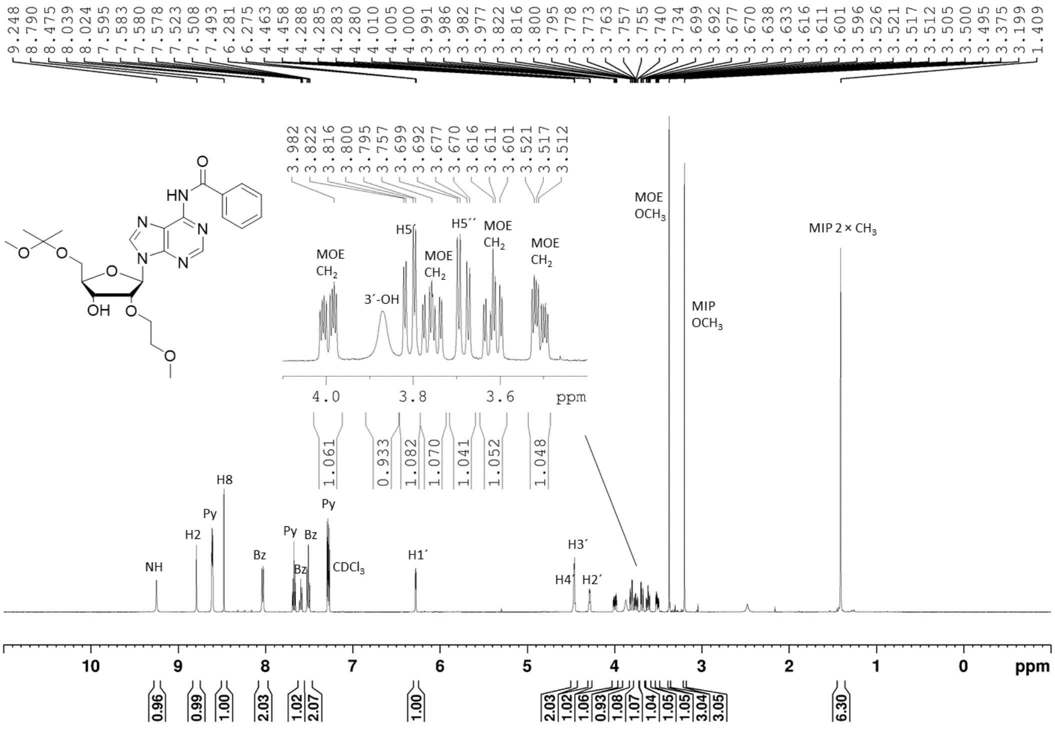

Figure S55. ^{1}H NMR (500 MHz, $CDCl_3$) spectrum of **2j**

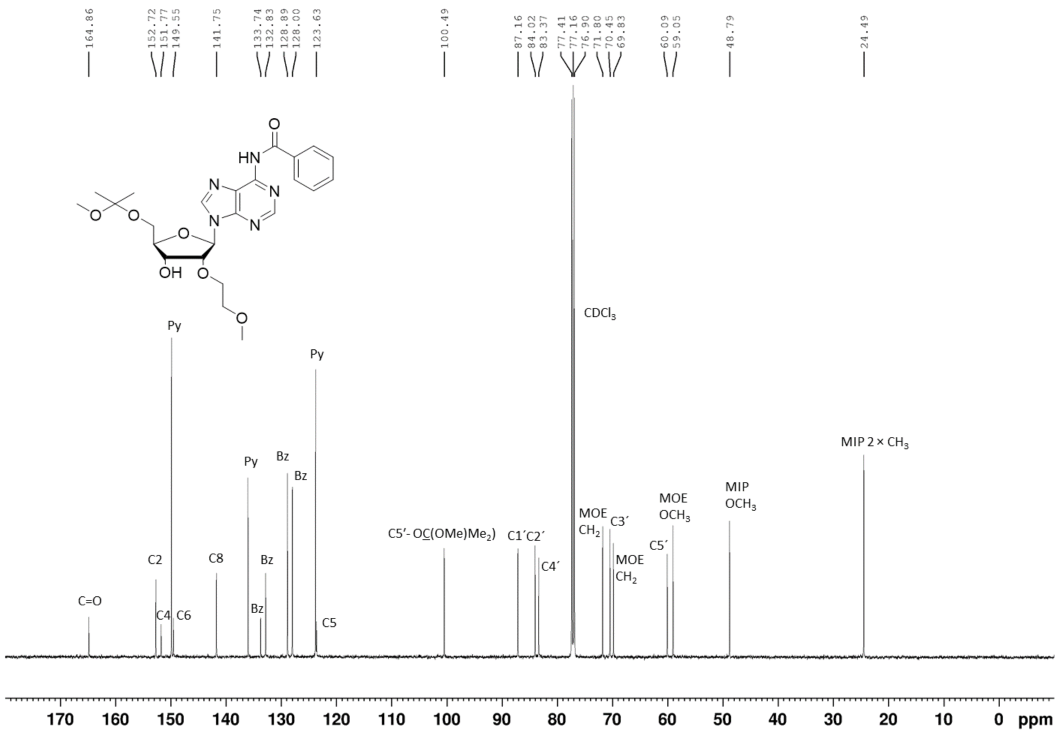

Figure S56. ^{13}C NMR (126 MHz, $CDCl_3$) spectrum of **2j**

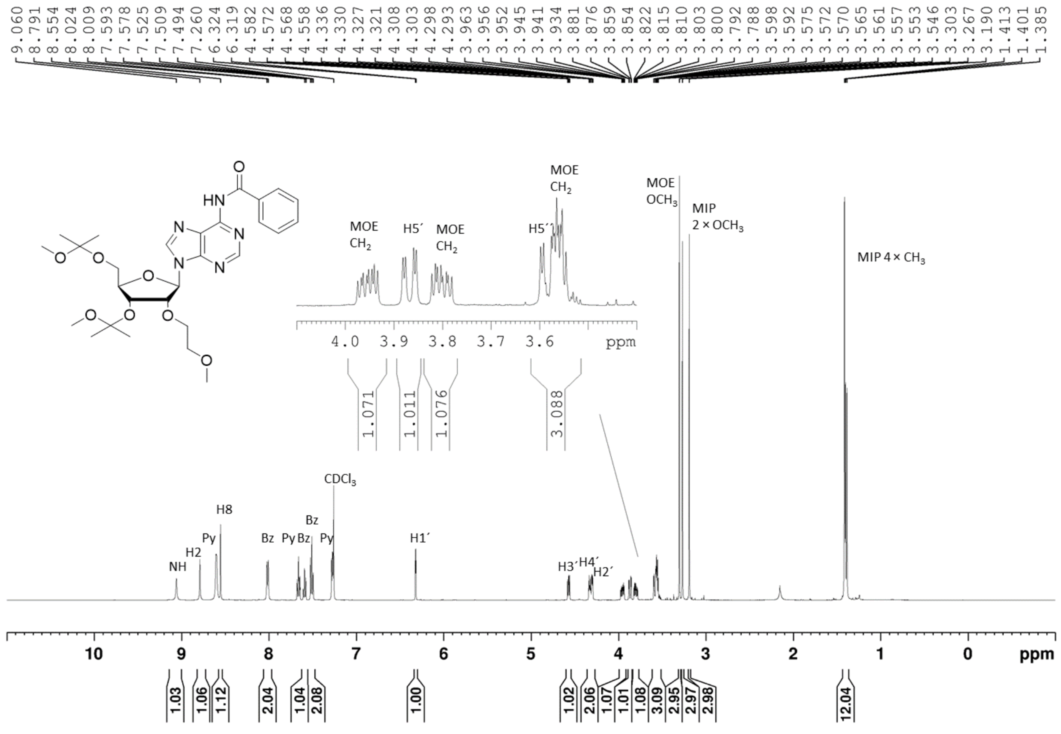

Figure S57 ^{1}H NMR (500 MHz, $CDCl_3$) spectrum of **4j**

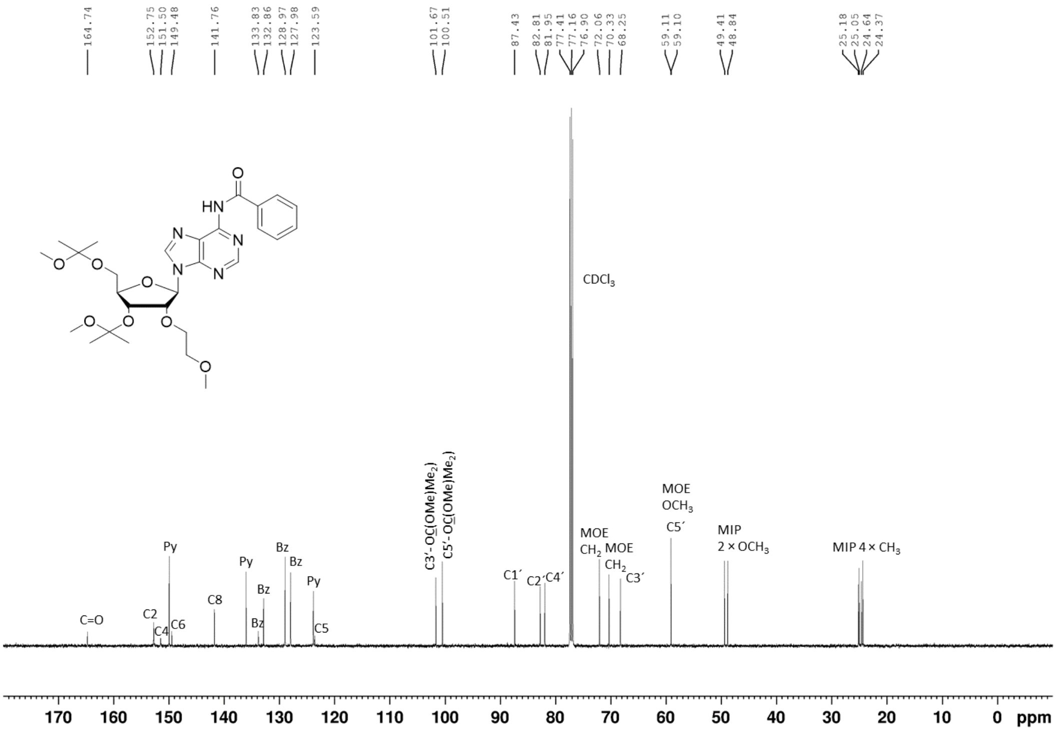

Figure S58. ^{13}C NMR (126 MHz, $CDCl_3$) spectrum of **4j**

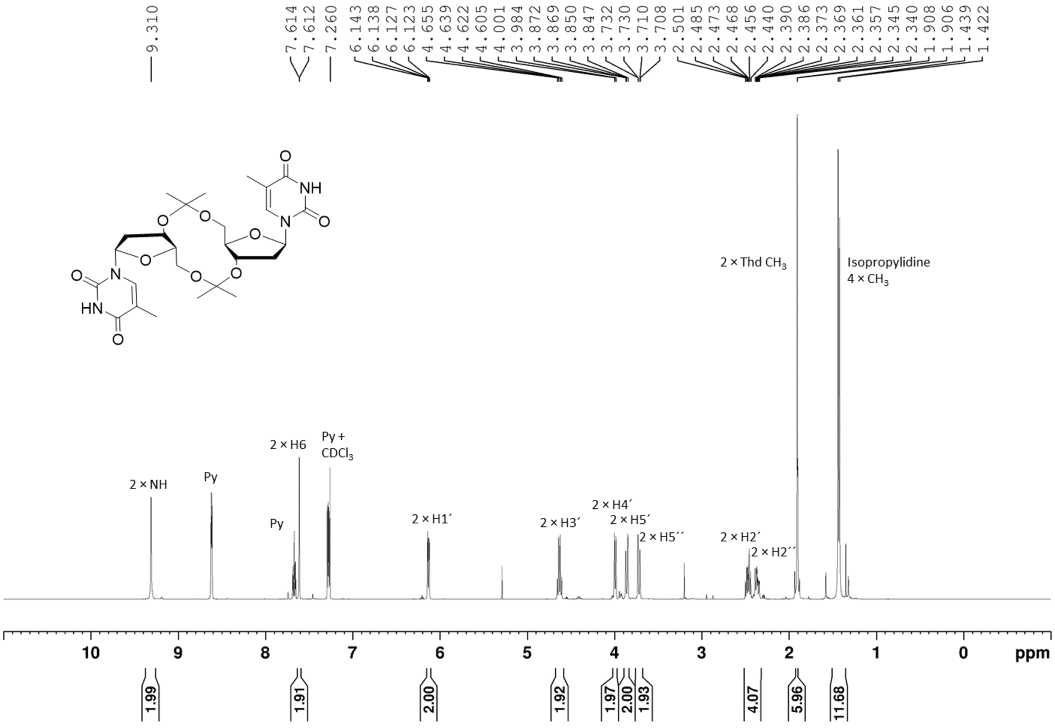

Figure S59. ^{1}H NMR (500 MHz, $CDCl_3$) spectrum of **5**

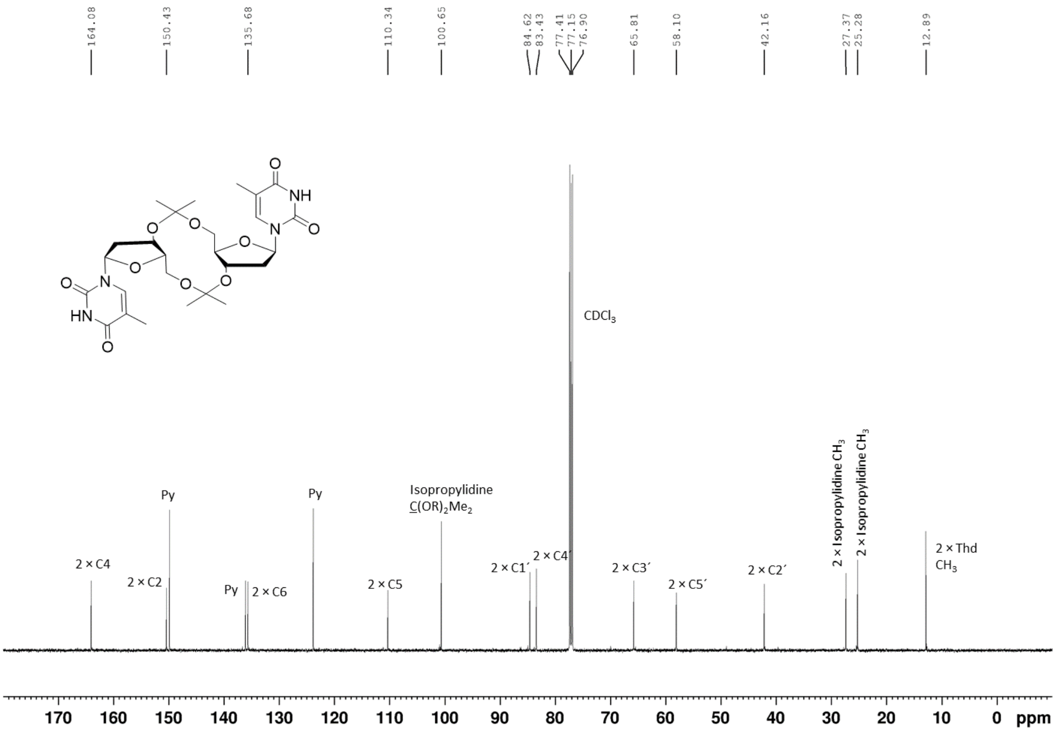

Figure S60. ^{13}C NMR (126 MHz, CDCl$_3$) spectrum of **5**

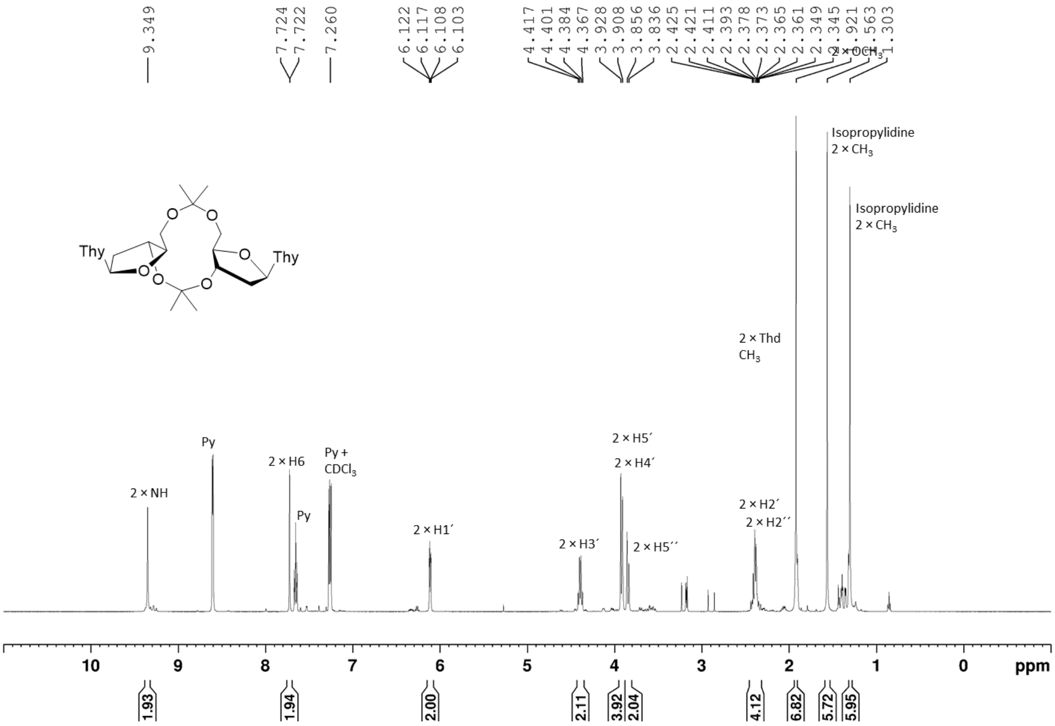

Figure S61. ^{1}H NMR (500 MHz, CDCl$_3$) spectrum of **6**

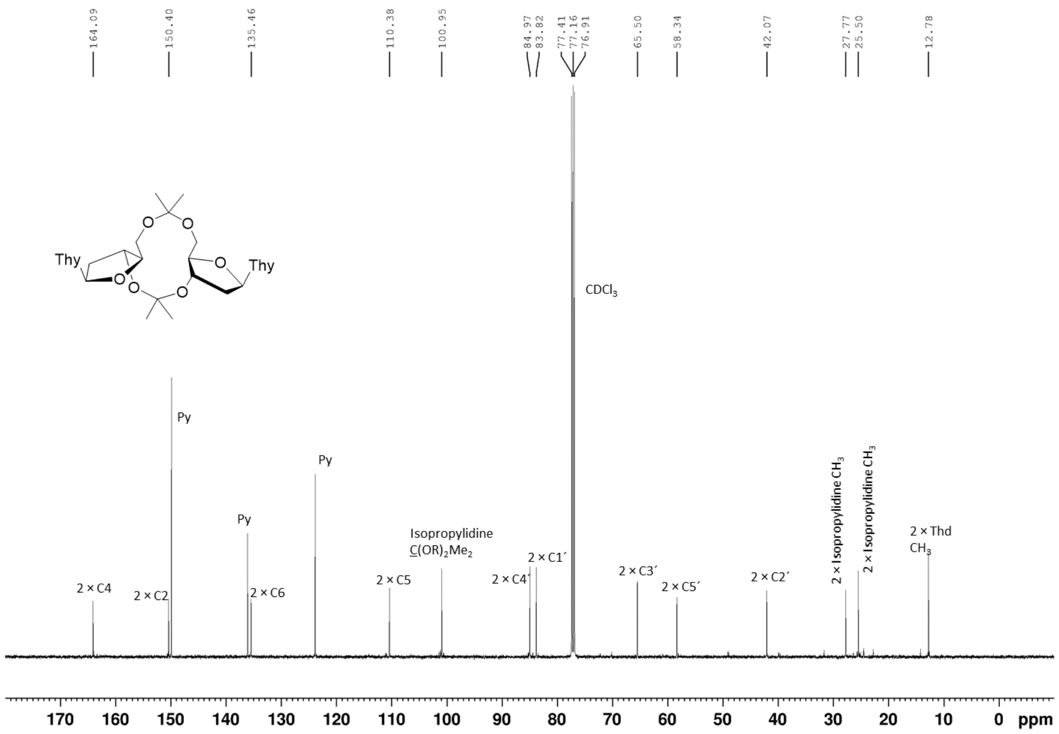

Figure S62. ^{13}C NMR (126 MHz, $CDCl_3$) spectrum of **6**

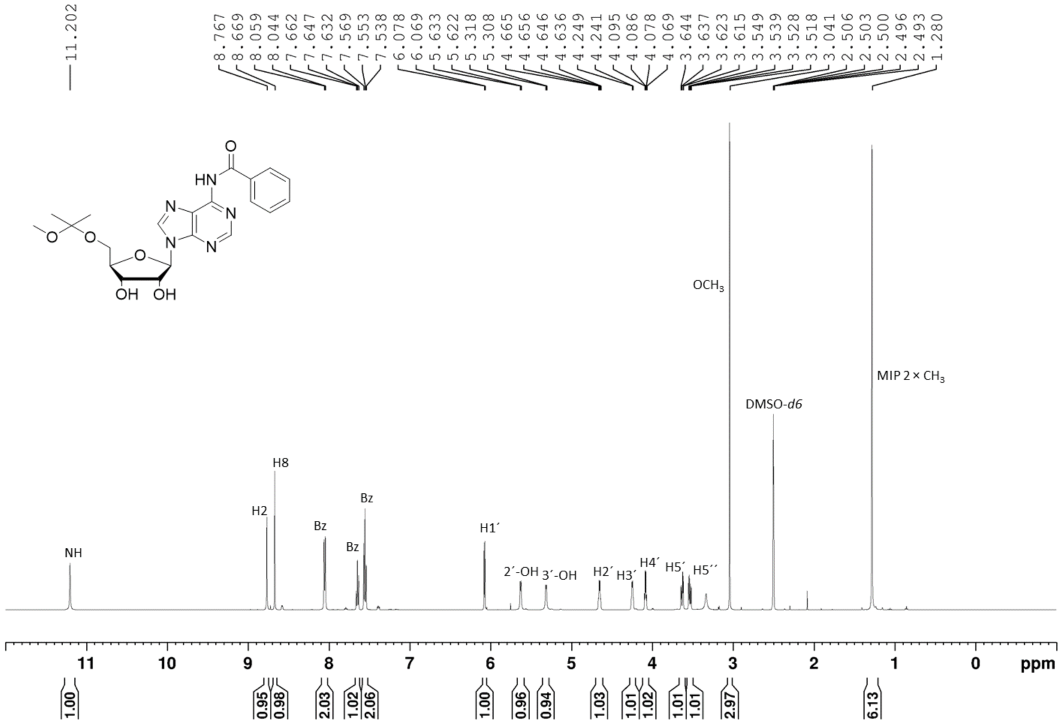

Figure S63. ^{1}H NMR (500 MHz, DMSO-*d*6) spectrum of **2k**

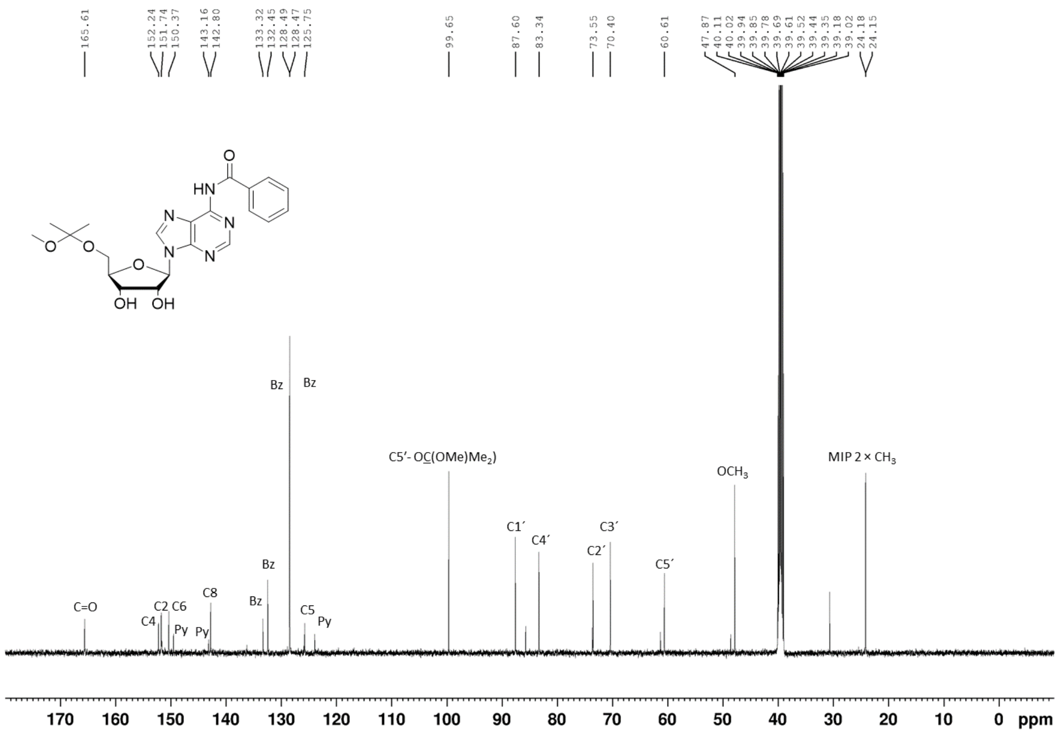

Figure S64. ^{13}C NMR (126 MHz, DMSO-*d*6) spectrum of **2k**

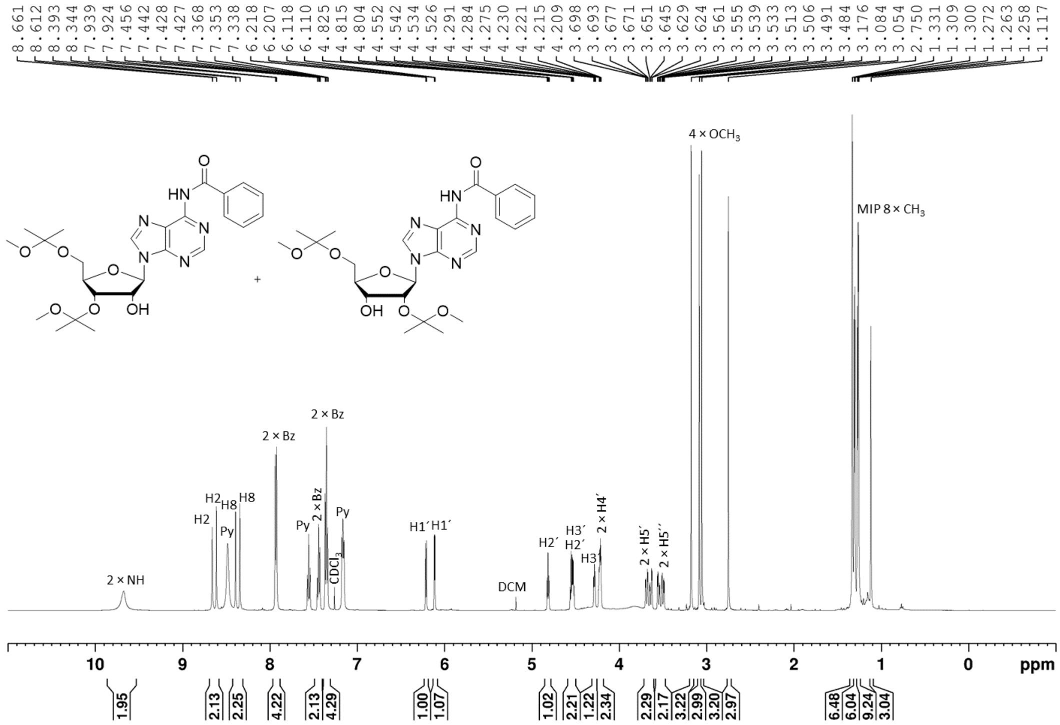

Figure S65. ^{1}H NMR (500 MHz, $CDCl_3$) spectrum of **4k** and **8**

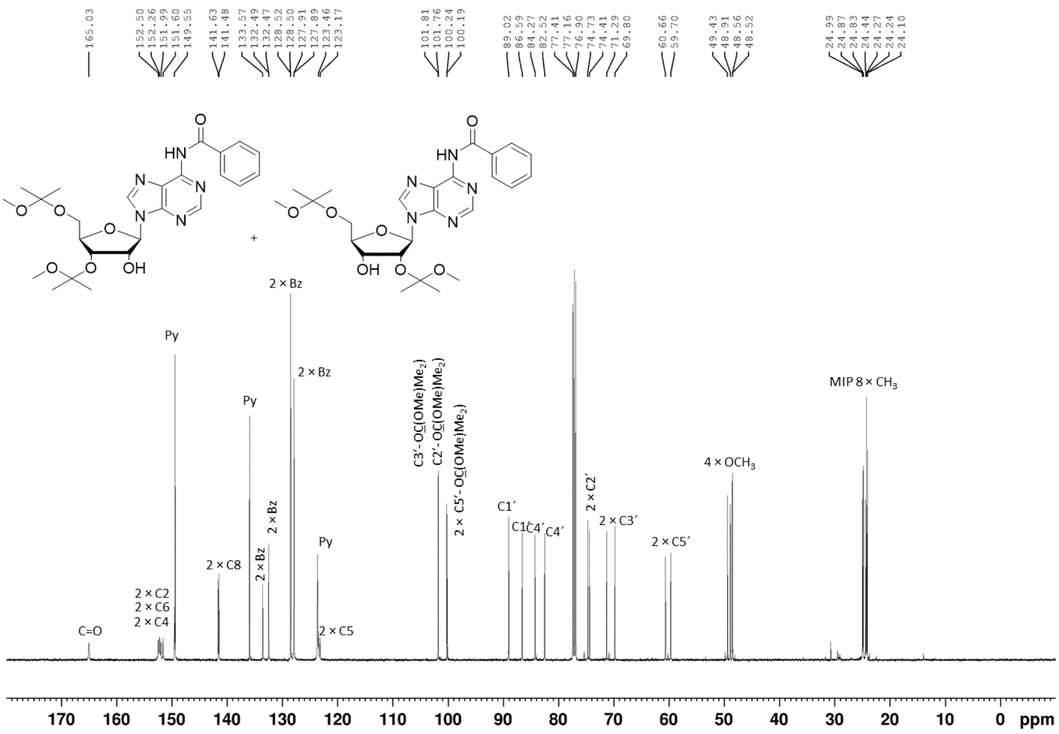

Figure S66. ^{13}C NMR (126 MHz, $CDCl_3$) spectrum of **4k** and **8**

Synthesis of phosphoramidite building blocks (10a-j) of 5’-*O*-MIP-protected nucleosides

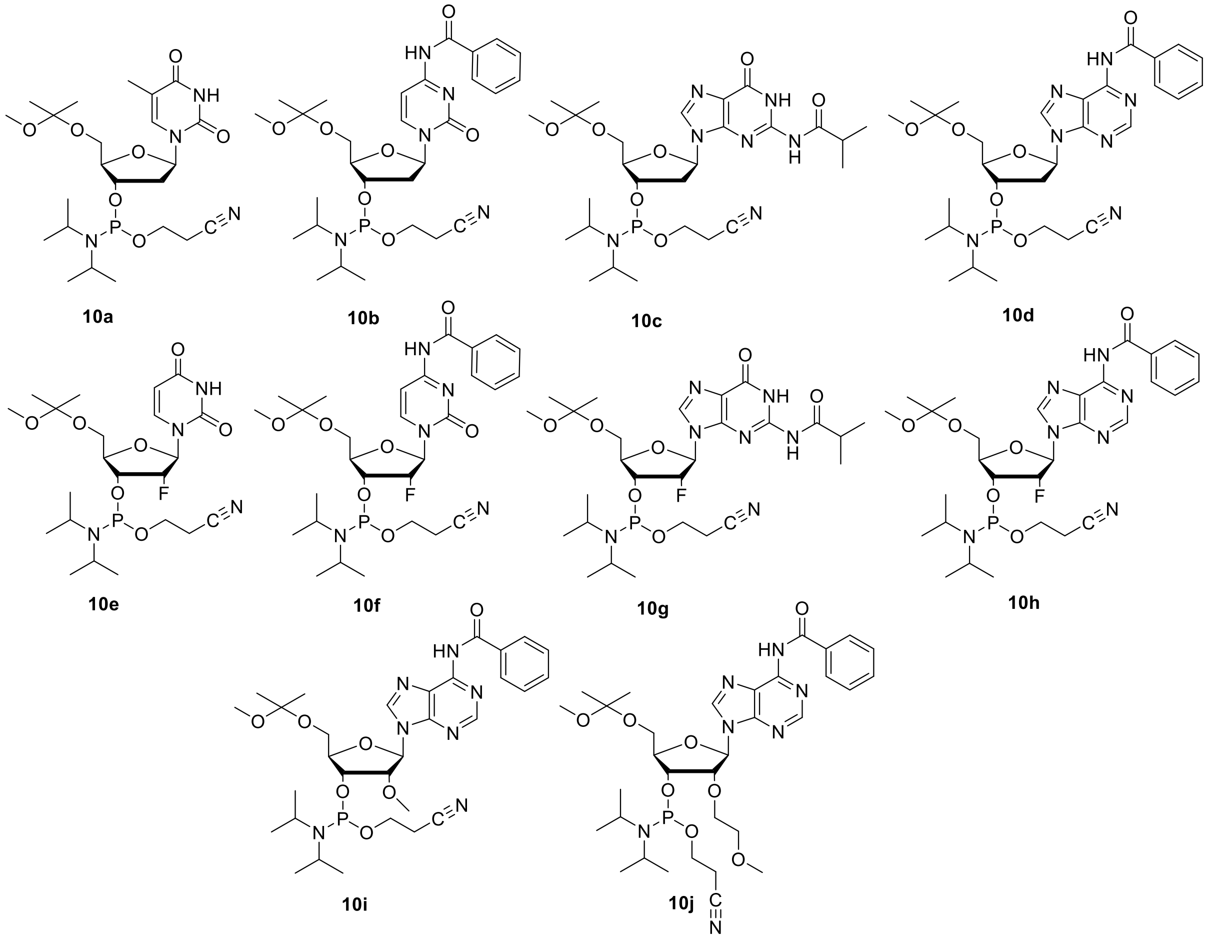

Figure 67. Synthesized phosphoramidite building blocks (**10a–j**) of 5´-*O*-MIP-protected nucleosides.

General procedure

5´-*O*-(2-methoxyprop-2-yl)-nucleoside (1.0 eq.) was dried with P_2O_5 overnight and it was dissolved in a mixture of anhydrous DCM (5.2 mL/mmol of nucleoside) and TEA (1.43 eq.) under nitrogen atmosphere at room temperature. 2-Cyanoethyl *N*,*N*-diisopropylchloro-phosphoramidite (1.2 eq.) was added to the mixture and the reaction was monitored by TLC. After 3-5h, the reaction mixture was directly loaded to a silica gel column to isolate the desired product.

5´-*O*-(2-methoxyprop-2-yl)-thymidine-3´-*O*-(2-cyanoethyl-*N,N*-diisopropyl)phosphoramidite (10a).

The synthesis was carried out as described in general procedure by using 5´-*O*-(2-methoxyprop-2-yl)-thymidine (**2a**) (0.48 g, 1.50 mmol). The crude product was purified by silica gel column chromatography (EtOAc/DCM/Et_3N, 49:49:2, *v*/*v*/*v*) to give **10a** as white solid (0.61 g, yield: 78%). The ^{1}H, ^{13}C and ^{31}P NMR spectra were identical to those previously reported in literature.[1] (cf. purity documentation of the product in Figure 124.)

***N*⁴-Benzoyl-5´-*O*-(2-methoxyprop-2-yl)-2´-deoxycytidine-3´-*O*-(2-cyanoethyl-*N,N*-diisopropyl)phosphoramidite (10b).**

The synthesis was carried out as described in general procedure by using N^4-Benzoyl-5´-*O*-(2-methoxyprop-2-yl)-2´-deoxycytidine (**2b**) (0.43 g, 1.04 mmol). The crude product was purified by silica gel column chromatography (EtOAc/DCM/Et_3N, 75:23:2, *v*/*v*/*v*) to give **10b** as white solid (0.58 g, yield: 93%). The ^{1}H, ^{13}C and ^{31}P NMR spectra were identical to those previously reported in literature.[1] (cf. purity documentation of the product in Figure 125.)

N^2-Isobutyryl-5´-*O*-(2-methoxyprop-2-yl)-2´-deoxyguanosine-3´-*O*-(2-cyanoethyl-*N,N*-diisopropyl)phosphoramidite (10c).

The synthesis was carried out as described in general procedure by using N^2-Isobutyryl-5´-*O*-(2-methoxyprop-2-yl)-2´-deoxyguanosine (**2c**) (0.66 g, 1.49 mmol). The crude product was purified by silica gel column chromatography (EtOAc/DCM/Et_3N, 49:49:2, *v*/*v*/*v*) to give **10c** as white solid (0.78 g, yield: 86%). The ^{1}H, ^{13}C and ^{31}P NMR spectra were identical to those previously reported in literature.[3] (cf. purity documentation of the product in Figure 126.)

N^6-Benzoyl-5´-*O*-(2-methoxyprop-2-yl)-2´-deoxyadenosine-3´-*O*-(2-cyanoethyl-*N,N*-diisopropyl)phosphoramidite (10d).

The synthesis was carried out as described in general procedure by using N^6-Benzoyl-5´-*O*-(2-methoxyprop-2-yl)-2´-deoxyadenosine (**2d**) (0.51 g, 1.15 mmol). The crude product was purified by silica gel column chromatography (EtOAc/DCM/Et_3N, 49:49:2, *v*/*v*/*v*) to give **10d** as white solid (0.59 g, yield: 83%). The ^{1}H, ^{13}C and ^{31}P NMR spectra were identical to those previously reported in literature.[1] (cf. purity documentation of the product in Figure 127.)

5´-*O*-(2-methoxyprop-2-yl)-2´-fluorouridine-3´-*O*-(2-cyanoethyl-*N,N*-diisopropyl)phosphoramidite (10e).

The synthesis was carried out as described in general procedure by using 5´-*O*-(2-methoxyprop-2-yl)-2´-fluorouridine (**2e**) (0.55 g, 1.73 mmol). The crude product was purified by silica gel column chromatography (EtOAc/DCM/Et_3N, 49:49:2, *v*/*v*/*v*) to give **10e** as white solid (0.77 g, yield: 85%). ^{1}H NMR (500 MHz, CD_3CN): δ 9.18 (br s, 1H), 7.92 (d, *J* = 8.0 Hz, 1H), 5.97 (d, *J* = 16.9 Hz, 1H), 5.64–5.62 (m, 1H), 5.07 (d, *J* = 53.1 Hz, 1H), 4.49–4.37 (m, 1H), 4.24–4.18 (m, 1H), 3.83–3.56 (m, 6H), 3.19 and 3.17 (2×s, 3H), 2.66–2.65 (m, 2H), 1.37–1.35 (m, 6H), 1.18 (d, *J* = 6.7 Hz, 12H). (note: multiplicity of some signals is due to the C-P and C-F couplings and the presence of the diastereomers) ^{13}C{^{1}H}NMR (126 MHz, CD_3CN): δ 164.0, 164.0, 151.2,151.2, 141.0, 119.5, 119.4, 102.5, 102.4, 101.2, 101.2, 94.9, 94.9, 94.2, 94.2, 93.4, 93.4, 92.7, 92.7, 89.3, 89.3, 89.1, 89.0, 88.9, 82.6, 82.5, 82.1, 82.0, 70.6, 70.5, 70.5, 70.4, 70.3, 59.7, 59.5, 59.4, 59.3, 59.3, 59.1, 49.2, 49.2, 44.2, 42.1, 44.1, 44.0, 24.9, 24.9, 24.9, 24.8, 24.8, 24.8, 24.7, 24.6, 24.5, 21.0, 21.0, 20.9, 20.9. ^{19}F NMR (471 MHz, CD_3CN): δ -201.64, -201.66, -202.39, -202.41. ^{31}P NMR (202 MHz, CD_3CN): δ 150.42, 150.38, 149.91, 149.87. HRMS (ESI): *m/z* [M + H]$^+$ calcd for $C_{22}H_{36}FN_4NaO_7P^+$ 541.2198, found 541.2200.

***N*⁴-Benzoyl-5´-*O*-(2-methoxyprop-2-yl)-2´-fluorocytidine-3´-(2-cyanoethyl-*N,N*-diisopropyl)phosphoramidite (10f).**

The synthesis was carried out as described in general procedure by using N^4-Benzoyl-5´-*O*-(2-methoxyprop-2-yl)-2´-fluorocytidine (**2f**) (0.28 g, 0.65 mmol). The crude product was purified by silica gel column chromatography (EtOAc/DCM/Et_3N, 49:49:2, *v*/*v*/*v*) to give **10f** as white solid (0.33 g, yield: 80%). ^{1}H NMR (500 MHz, CD_3CN): δ 9.27 (br s, 1H), 8.47 (d, *J* = 7.4 Hz, 1H), 7.96 (d, *J* = 7.4 Hz, 2H), 7.64–7.61 (m, 1H), 7.53–7.56 (m, 2H), 7.41 (br s, 1H), 6.02–5.98 (m, 1H), 5.16–5.03 (m, 1H), 4.53–4.42 (m, 1H), 4.31–4.26 (m, 1H), 3.91–3.61 (m, 6H), 3.22 and 3.21 (2×s, 3H), 2.64–2.63 (m, 2H), 1.40–1.39 (m, 6H), 1.18–1.17 (m, 12H). (note: multiplicity of some signals is due to the C-P and C-F couplings and the presence of the diastereomers) ^{13}C{^{1}H}NMR (126 MHz, CD_3CN): δ 168.3, 164.0, 164.0, 155.5, 145.8, 134.4, 133.9, 129.6, 129.1, 119.5, 119.4, 101.3, 96.9, 96.8, 95.1, 94.5, 94.5, 93.6, 93.0, 93.0, 90.8, 90.4, 90.5, 90.5, 82.4, 82.4, 82.1, 82.0, 70.3, 70.2, 70.1, 69.9, 69.7, 69.6, 59.7, 59.6, 59.3, 59.1, 58.9, 58.7, 49.3, 49.3, 44.2, 44.1, 44.0, 24.9, 24.9, 24.9, 24.8, 24.8, 24.7, 24.7, 24.6, 21.0, 20.9, 20.9, 20.9. ^{19}F NMR (471 MHz, CD_3CN): δ -201.04, -201.06, -201.40, -201.41. ^{31}P NMR (202 MHz, CD_3CN): δ 150.51, 150.47, 149.83, 149.79. HRMS (ESI): *m/z* [M + H]$^+$ calcd for $C_{29}H_{42}FN_5O_7P^+$ 622.2800, found 622.2803.

***N*²-Isobutyryl-5´-(2-methoxyprop-2-yl)-2´-fluoroguanosine-3´-(2-cyanoethyl-*N,N*-diisopropyl)phosphoramidite (10g).**

The synthesis was carried out as described in general procedure by using N^2-isobutytyl-5´-*O*-(2-methoxyprop-2-yl)-2´-fluoroguanosine (**2g**) (0.33 g, 0.77 mmol). The crude product was

purified by silica gel column chromatography (DCM/MeOH/Et_3N, 94:4:2, *v*/*v*/*v*) to give **10g** as white solid (0.38 g, yield: 78%). ^{1}H NMR (500 MHz, CD_3CN): δ 7.99 and 7.98 (s, 1H), 6.13–6.09 (m, 1H), 5.47–5.34 (m, 1H), 4.73–4.62 (m, 1H), 4.29–4.24 (m, 1H), 3.89–3.58 (m, 6H), 3.13 and 3.11 (s, 3H), 2.77–2.70 (m, 1H), 2.70–2.64 (m, 2H), 1.35–1.32 (m, 6H), 1.20–1.17 (m, 18H). (note: multiplicity of some signals is due to the C-P and C-F couplings and the presence of the diastereomers) ^{13}C{^{1}H}NMR (126 MHz, CD_3CN): δ 181.0, 180.9, 156.3, 149.3, 149.3, 149.3, 149.2, 138.1, 138.0, 122.1, 122.1, 119.5, 101.2, 101.2, 94.9, 94.3, 93.4, 92.7, 87.7, 87.7, 87.5, 87.4, 83.3, 83.2, 82.8, 82.7, 71.4, 71.3, 71.3, 71.2, 60.3, 60.0, 59.6, 59.5, 59.4, 59.3, 49.1, 49.0, 44.2, 44.2, 44.1, 44.1, 36.6, 36.6, 24.9, 24.9, 24.8, 24.8, 24.8, 24.7, 24.6, 24.5, 21.0, 21.0, 20.9, 19.8, 19.2, 19.2, 19.2. ^{19}F NMR (471 MHz, CD_3CN): δ -202.61, -202.64, -203.2, -203.2. ^{31}P NMR (202 MHz, CD_3CN): δ 150.40, 150.34, 149.94, 149.90. HRMS (ESI): *m/z* $[M + H]^+$ calcd for $C_{27}H_{44}FN_7O_7P^+$ 628.3018, found 628.3021.

N^6-Benzoyl-5´-*O*-(2-methoxyprop-2-yl)-2´-fluoroadenosine-3´-(2-cyanoethyl-*N*,*N*-diisopropyl)phosphoramidite (10h).

The synthesis was carried out as described in general procedure by using N^6-benzoyl-5´-*O*-(2-methoxyprop-2-yl)-2´-fluoroadenosine (**2h**) (0.44 g, 0.98 mmol). The crude product was purified by silica gel column chromatography (EtOAc/DCM/Et_3N, 49:49:2, *v*/*v*/*v*) to give **10h** as white solid (0.45 g, yield: 71%). ^{1}H NMR (500 MHz, CD_3CN): δ 9.34 (br s, 1H), 8.66 (s, 1H), 8.41 (s, 1H), 8.00 (d, J = 6.9 Hz, 2H), 7.65–7.63 (m, 1H), 7.56–7.53 (m, 2H), 6.39–6.34 (m, 1H), 5.65–5.54 (m, 1H), 5.02–4.92 (m, 1H), 4.33–4.28 (m, 1H), 3.90–3.58 (m, 6H), 3.08 and 3.05 (2 × s, 3H), 2.69–2.65 (m, 2H), 1.29–1.20 (m, 18H). (note: multiplicity of some signals is due to the C-P and C-F couplings and the presence of the diastereomers) ^{13}C{^{1}H}NMR (126 MHz, CD_3CN): δ 166.3, 152.8, 152.6, 151.0. 143.3, 143.3, 134, 8, 133.6, 125.8, 119.5, 119.5, 101.1. 101.1. 94.9, 94.5, 94.5, 93.4, 93.0, 93.0, 88.5, 88.4, 88.2, 88.1, 83.1, 83.0, 82.7. 82.6, 71.4, 71.3, 71.3, 71.2, 71.0, 60.2, 60.0, 59.9, 59.7, 59.7, 59.5, 49.0, 48.9, 44.2, 44.2, 44.1, 44.1, 25.0, 25.0, 24.9, 24.8, 24.8, 24.8, 24.7, 24.7, 24.5, 24.5, 21.0, 21.0, 21.0, 21.0. ^{19}F NMR (471 MHz, CD_3CN): δ -201.64, -201.66, -202.22, -202.24. ^{31}P NMR (202 MHz, CD_3CN): δ 150.09, 150.04, 150.01. HRMS (ESI): *m/z* $[M + H]^+$ calcd for $C_{30}H_{42}FN_7O_6P^+$ 646.2913, found 646.2916.

N^6-Benzoyl-5´-*O*-(2-methoxyprop-2-yl)-2´-*O*-methyladenosine-3´-(2-cyanoethyl-*N*,*N*-diisopropyl)phosphoramidite (10i).

The synthesis was carried out as described in general procedure by using N^6-Benzoyl-5´-*O*-(2-methoxyprop-2-yl)-2´-*O*-methyladenosine (**2i**) (0.21 g, 0.47 mmol). The crude product was purified by silica gel column chromatography (EtOAc/DCM/Et_3N, 49:49:2, *v*/*v*/*v*) to give **10i** as white solid (0.25 g, yield: 81%). ^{1}H NMR (500 MHz, CD_3CN): δ 9.74 (br s, 1H), 8.64 (s, 1H), 8.47 and 8.45 (s, 1H), 8.00–7.98 (m, 2H), 7.62–7.59 (m, 1H), 7.52–7.49 (m, 2H), 6.19 (d, J = 5.0 Hz, 1H), 4.70–4.65 (m, 1H), 4.51 and 4.47 (dd, J = 4.8 Hz, 1H), 4.38–4.28 (m, 1H), 3.93–3.57 (m, 6H), 3.48 and 3.44 (s, 3H), 3.13 and 3.01 (s, 3H), 2.72–2.66 (m, 2H), 1.34–1.30 (m, 6H), 1.23–1.19 (m, 12H). (note: multiplicity of some signals is due to the C-P couplings

and the presence of the diastereomers) $^{13}C\{^{1}H\}$NMR (126 MHz, CD_3CN): δ 166.6, 153.0, 153.0, 152.7, 150.9, 143.0, 142.9, 134.9, 133.5, 129.6, 129.1, 125.6, 125.5, 119.6, 119.5, 101.2, 101.1, 87.5, 85.3, 84.7, 84.7, 84.3, 84.3, 83.8, 83.8, 83.7, 83.6, 72.6, 72.5, 71.9, 71.8, 91.1, 61.0, 59.9, 59.7, 59.3, 59.1, 59.0, 59.0, 58.7, 58.7, 49.1, 49.0, 44.2, 44.1, 44.0, 25.0, 24.9, 24.9, 24.8, 24.8, 24.7, 24.6, 24.6, 21.0, 21.0, 21.0, 21.0. ^{31}P NMR (202 MHz, CD_3CN): δ 150.07, 149.60. HRMS (ESI): *m/z* $[M + H]^+$ calcd for $C_{31}H_{45}N_7O_7P^+$ 658.3113, found 658.3115.

N^6-Benzoyl-5´-*O*-(2-methoxyprop-2-yl)-2´-*O*-methoxyethyladenosine-3´-(2-cyanoethyl-*N*,*N*-diisopropyl)phosphoramidite (10j).

The synthesis was carried out as described in general procedure by using N^6-Benzoyl-5´-*O*-(2-methoxyprop-2-yl)-2´-*O*-methoxyethyladenosine (**2j**) (0.18 g, 0.36 mmol). The crude product was purified by silica gel column chromatography (EtOAc/DCM/Et_3N, 49:49:2, *v*/*v*/*v*) to give **10j** as white solid (0.25 g, yield: 74%). ^{1}H NMR (500 MHz, CD_3CN): δ 9.67 (br s, 1H), 8.66 (s, 1H), 8.47 and 8.45 (s, 1H), 8.01–7.99 (m, 2H), 7.64–7.60 (m, 1H), 7.54–7.51 (m, 2H), 6.19–6.18 (m, 1H), 4.71–4.62 (m, 2H), 4.37–4.27 (m, 1H), 3.95–3.43 (m, 10H), 3.21 and 3.19 (s, 3H), 3.13 and 3.10 (s, 3H), 2.70–2.66 (m, 2H), 1.35–1.30 (m, 6H), 1.24–1.19 (m, 12H). (note: multiplicity of some signals is due to the C-P couplings and the presence of the diastereomers)$^{13}C\{^{1}H\}$NMR (126 MHz, CD_3CN): δ 166.6, 153.1, 153.0, 152.7, 150.9, 143.1, 142.9, 134.9, 133.5, 129.6, 129.1, 125.6, 125.5, 119.6, 119.5, 101.1, 87.8, 87.5, 84.7, 84.7, 84.3, 84.3, 82.5, 82.4, 82.4, 72.7, 72.6, 72.6, 72.0, 71.8, 71.0. 71.0, 71.7, 70.7, 61.1, 61.0, 59.9, 59.7, 59.2, 59.1, 59.0, 48.0, 49.0, 46.9, 44.1, 44.0, 44.0, 43.9, 25.1, 25.0, 24.9, 24.9, 24.9, 24.8, 24.7, 24.6, 24.6, 21.0, 21.0. ^{31}P NMR (202 MHz, CD_3CN): δ 149.73, 149.35. HRMS (ESI): *m/z* $[M + H]^+$ calcd for $C_{33}H_{49}N_7O_8P^+$ 702.3375, found 702.3377.

NMR spectra of 5´-*O*-MIP phosphoramidite building blocks

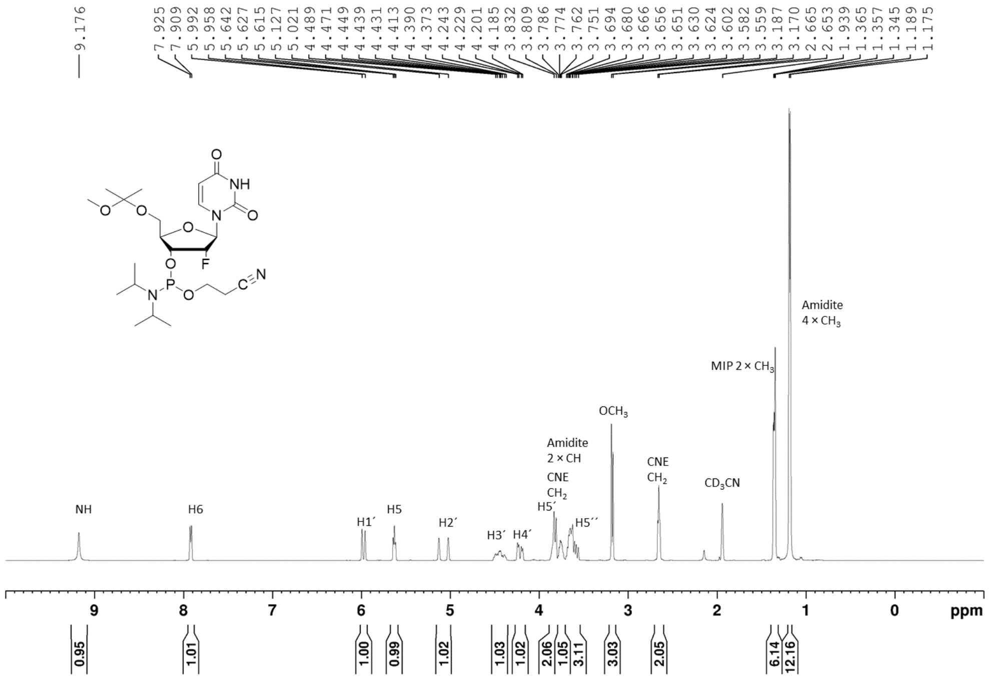

Figure S68. ^{1}H NMR (500 MHz, CD_3CN) spectrum of **10e**

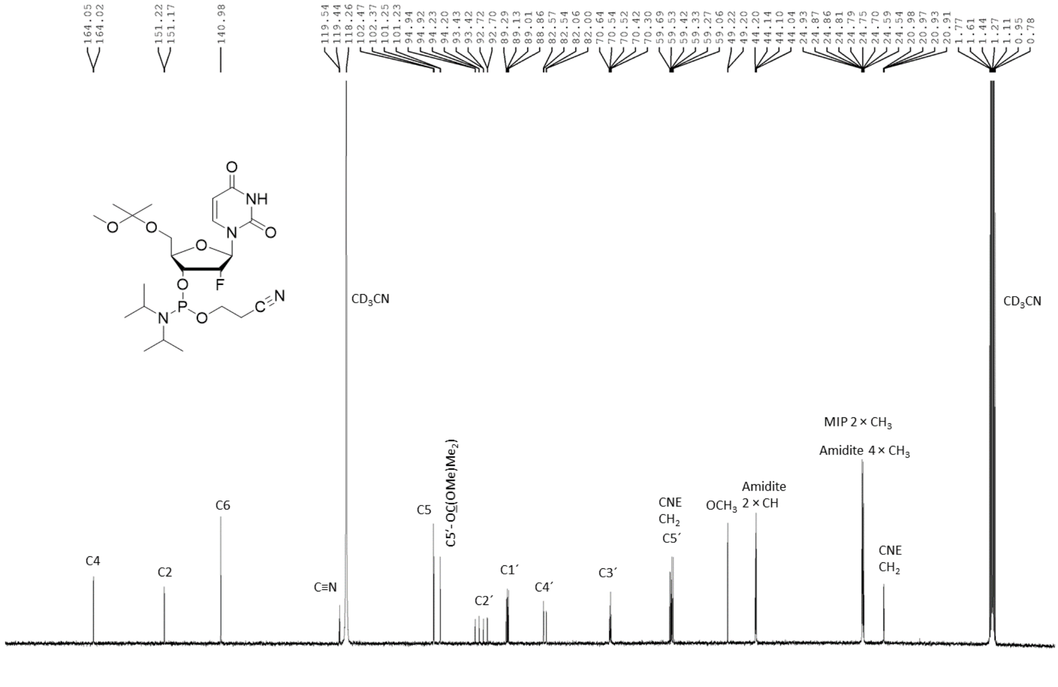

Figure S69. ^{13}C NMR (126 MHz, CD_3CN) spectrum of **10e**

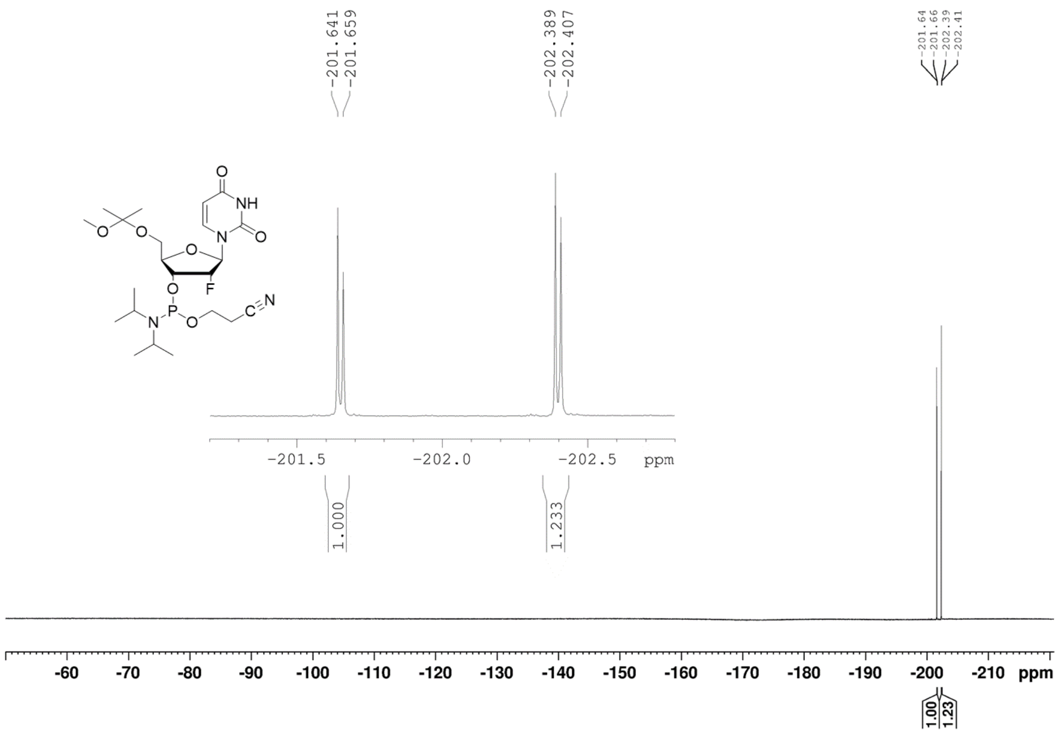

Figure S70. ^{19}F NMR (471 MHz, CD_3CN) spectrum of **10e**

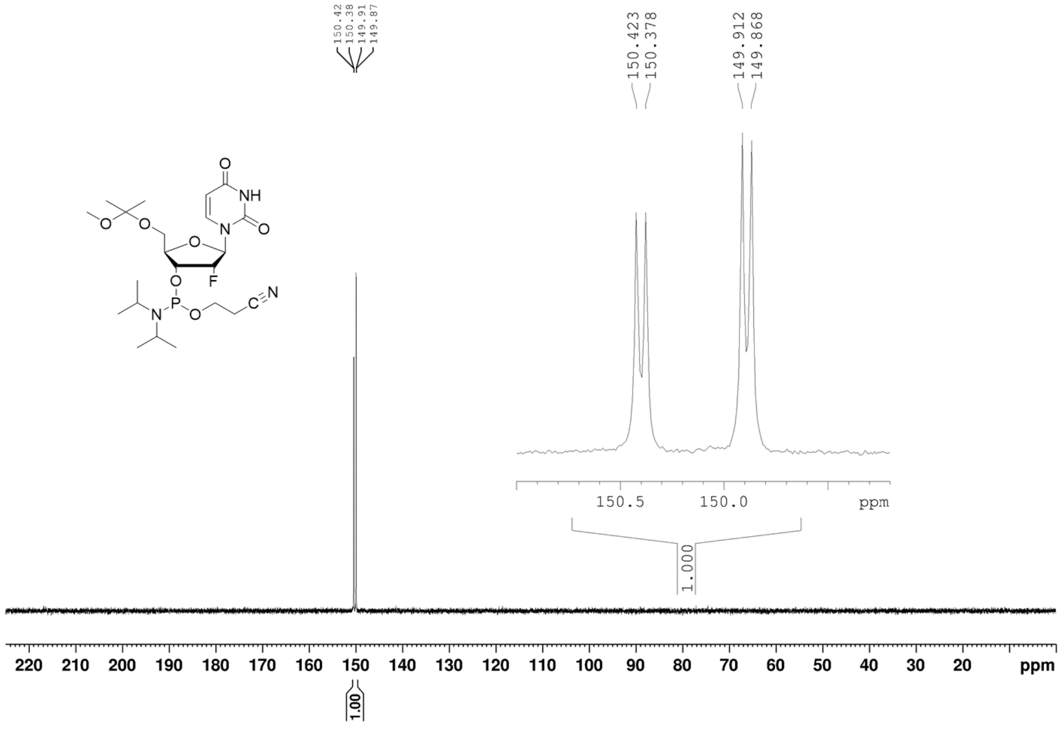

Figure S71. ^{31}P NMR (202 MHz, CD_3CN) spectrum of **10e**

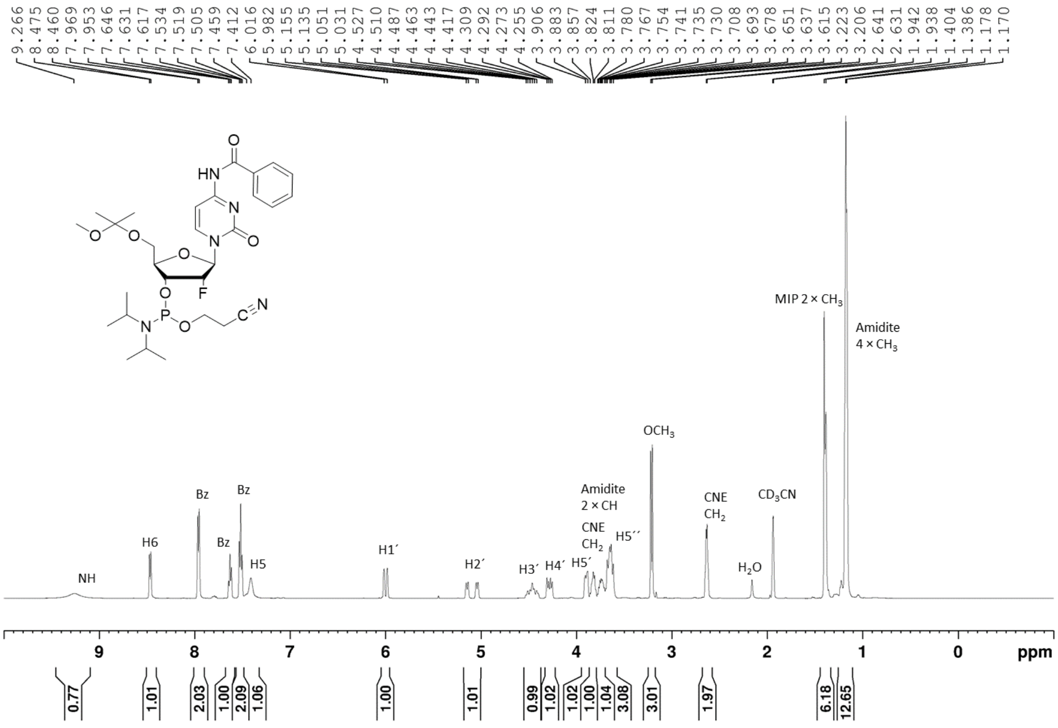

Figure S72. ^{1}H NMR (500 MHz, CD_3CN) spectrum of **10f**

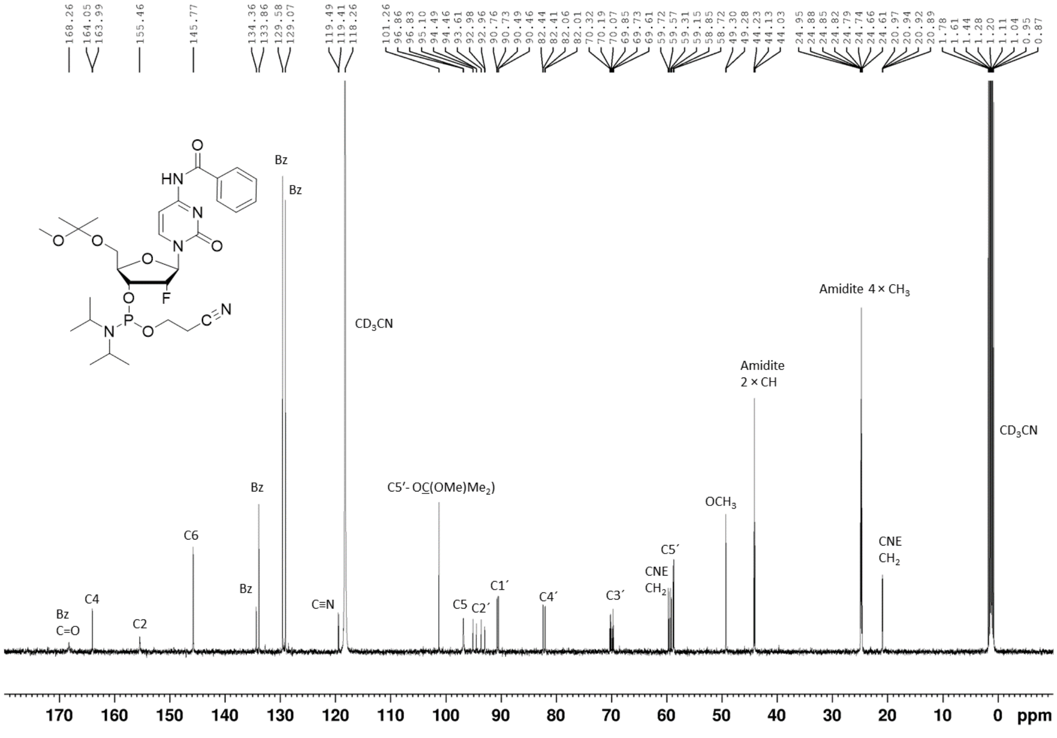

Figure S73. ^{13}C NMR (126 MHz, CD_3CN) spectrum of **10f**

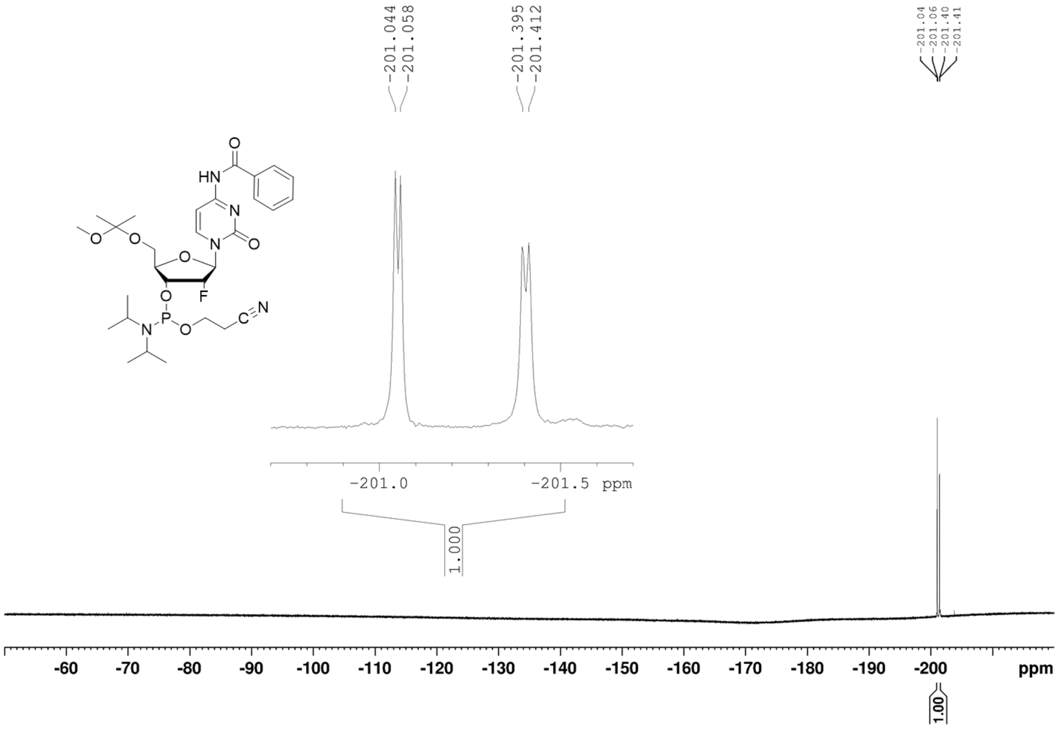

Figure S74. ^{19}F NMR (471 MHz, CD_3CN) spectrum of **10f**

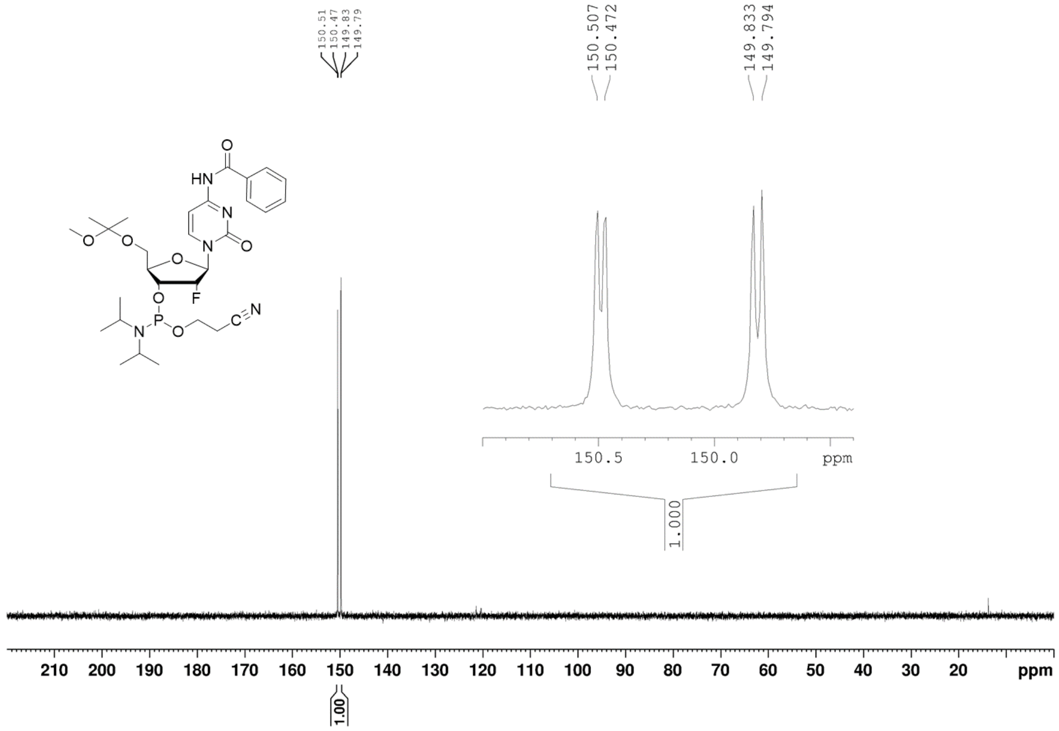

Figure S75. ^{31}P NMR (202 MHz, CD_3CN) spectrum of **10f**

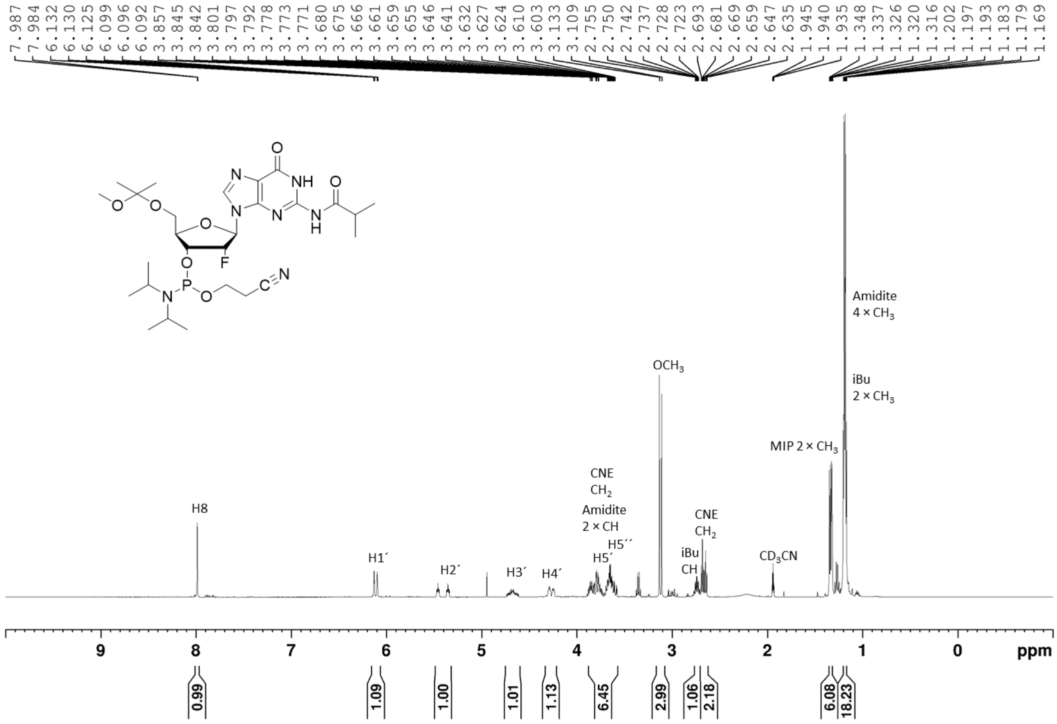

Figure S76. ^{1}H NMR (500 MHz, CD_3CN) spectrum of **10g**

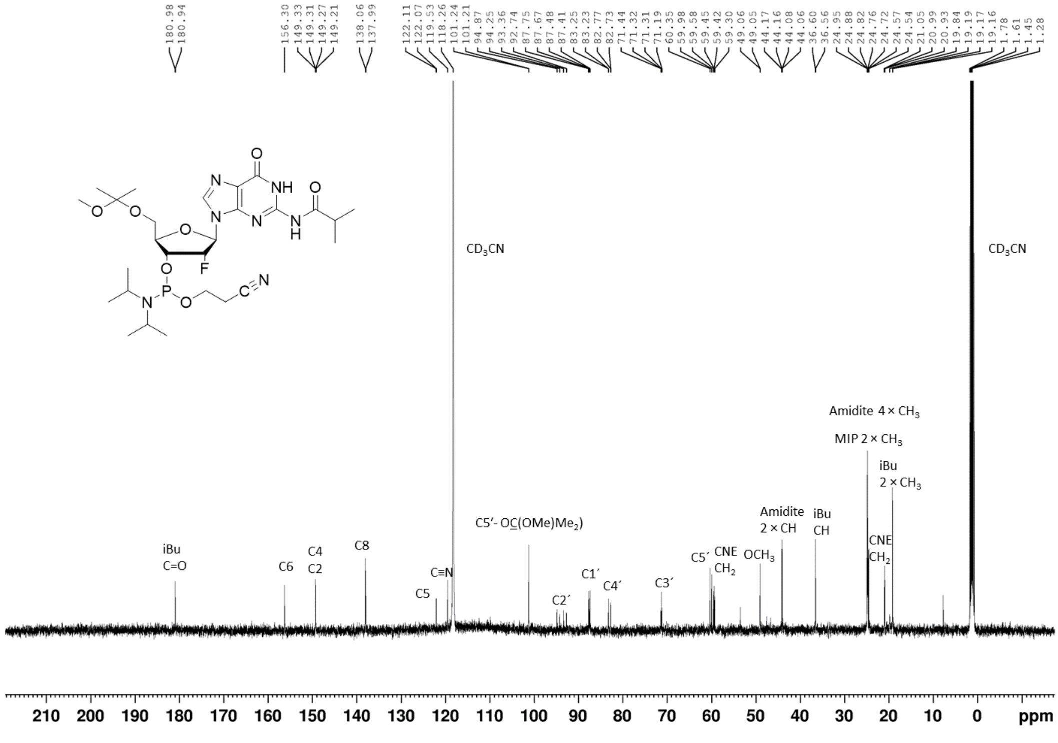

Figure S77. ^{13}C NMR (126 MHz, CD_3CN) spectrum of **10g**

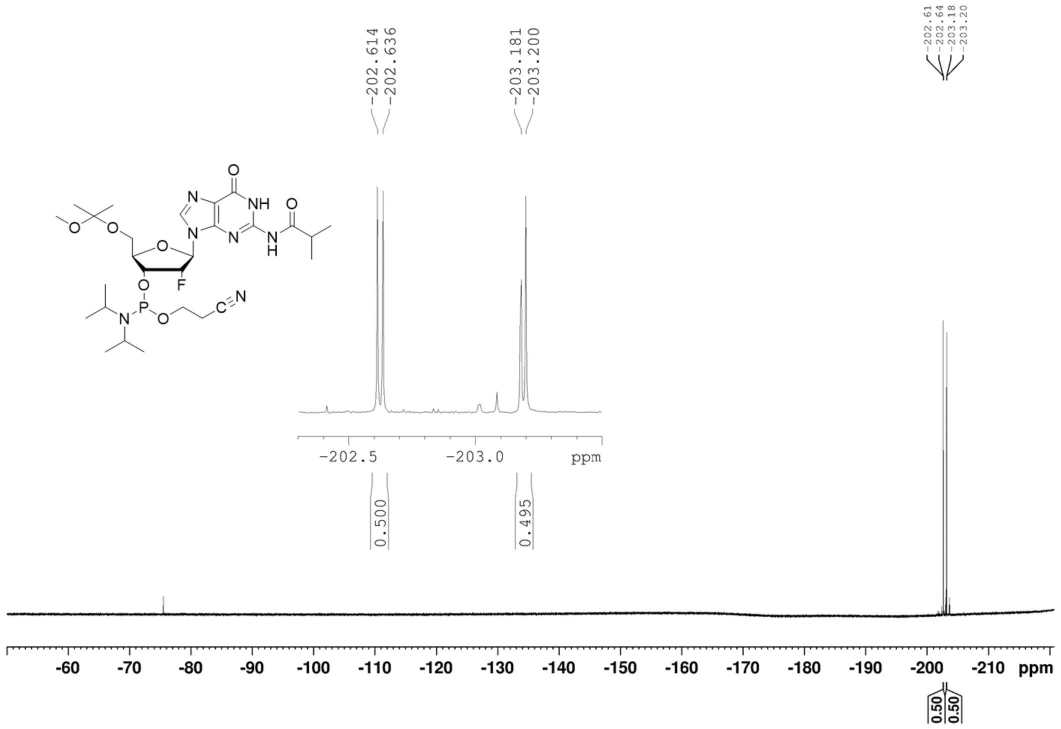

Figure S78. ^{19}F NMR (471 MHz, CD_3CN) spectrum of **10g**

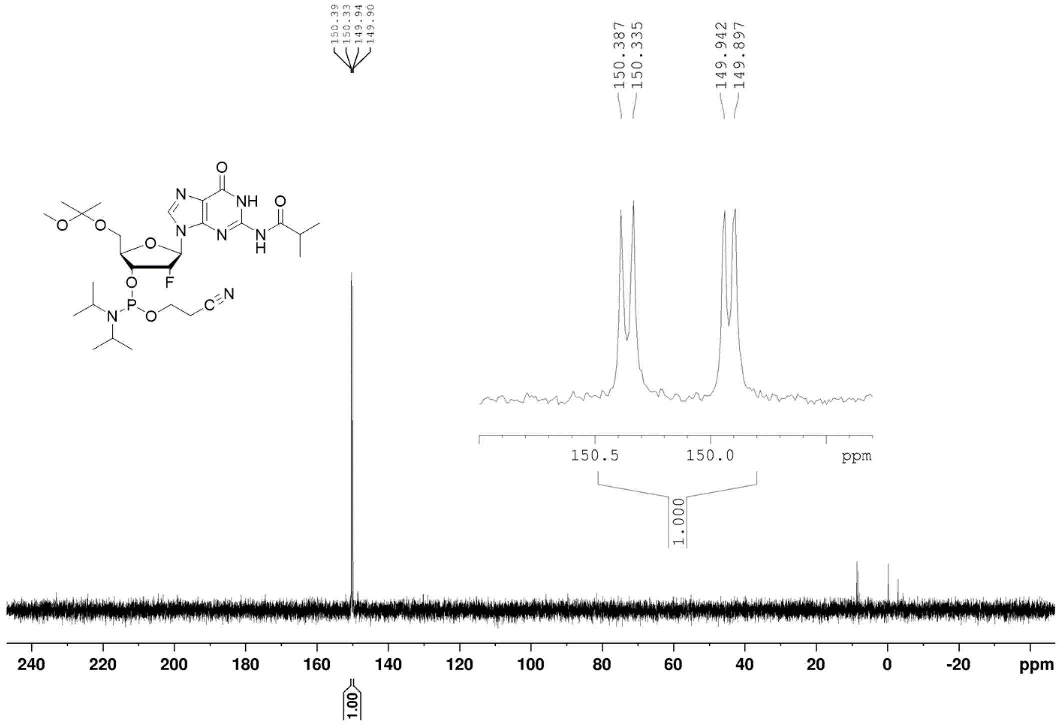

Figure S79. ^{31}P NMR (202 MHz, CD_3CN) spectrum of **10g**

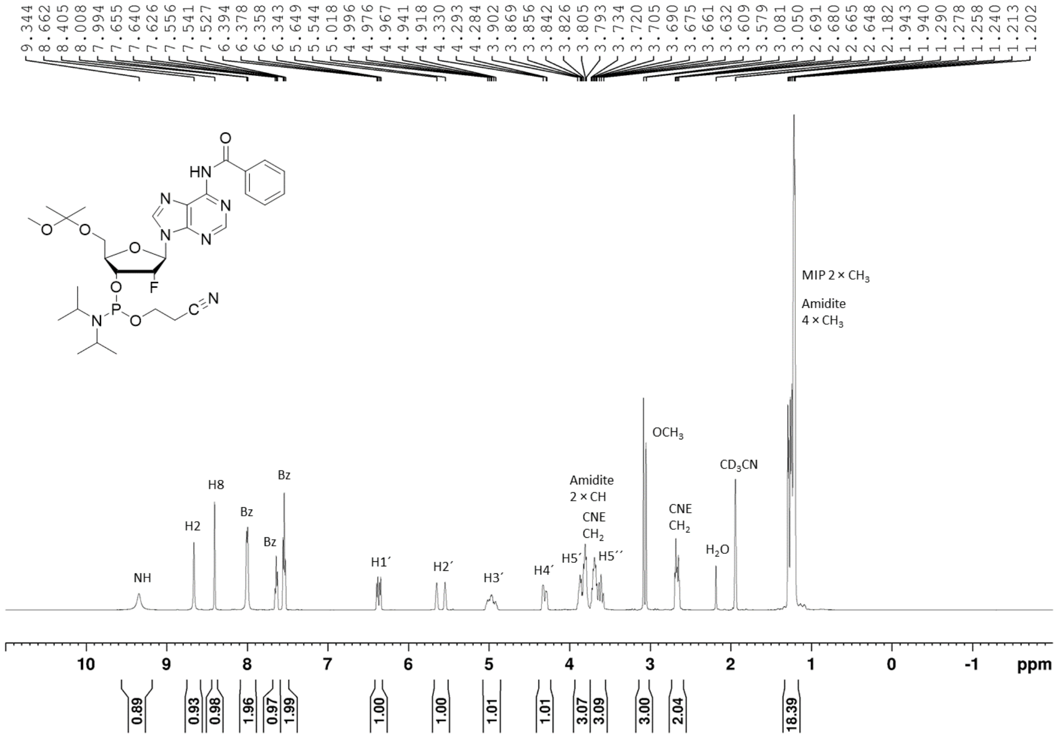

Figure S80. ^{1}H NMR (500 MHz, CD_3CN) spectrum of **10h**

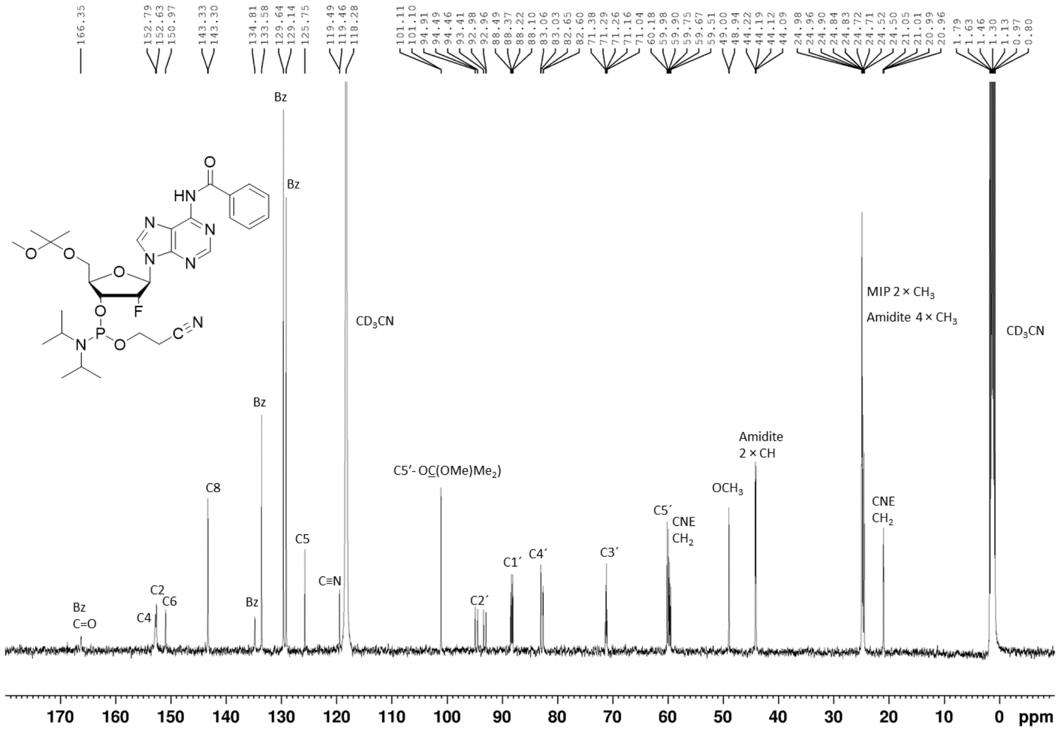

Figure S81. ^{13}C NMR (126 MHz, CD_3CN) spectrum of **10h**

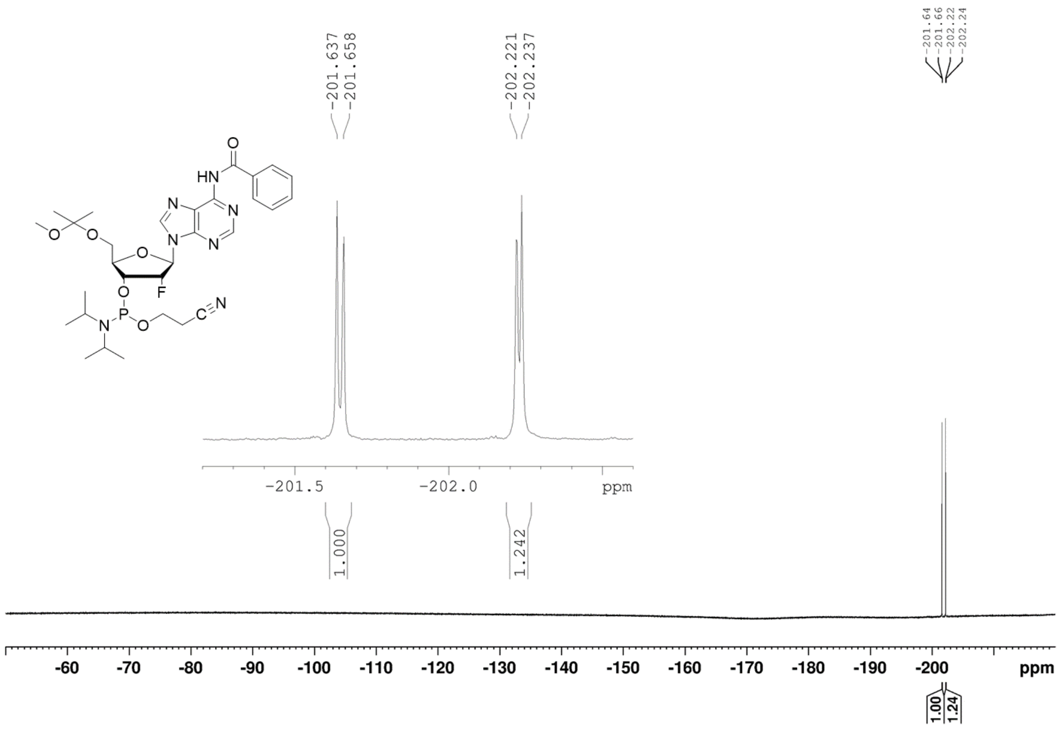

Figure S82. ^{19}F NMR (471 MHz, CD_3CN) spectrum of **10h**

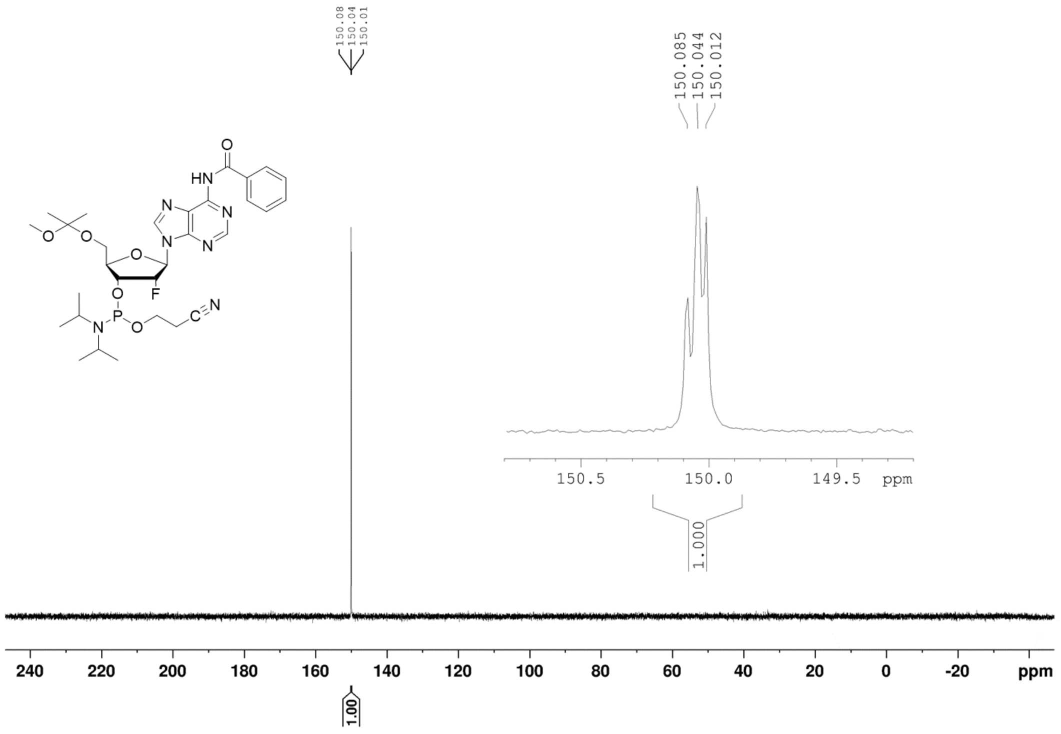

Figure S83. ^{31}P NMR (202 MHz, CD_3CN) spectrum of **10h**

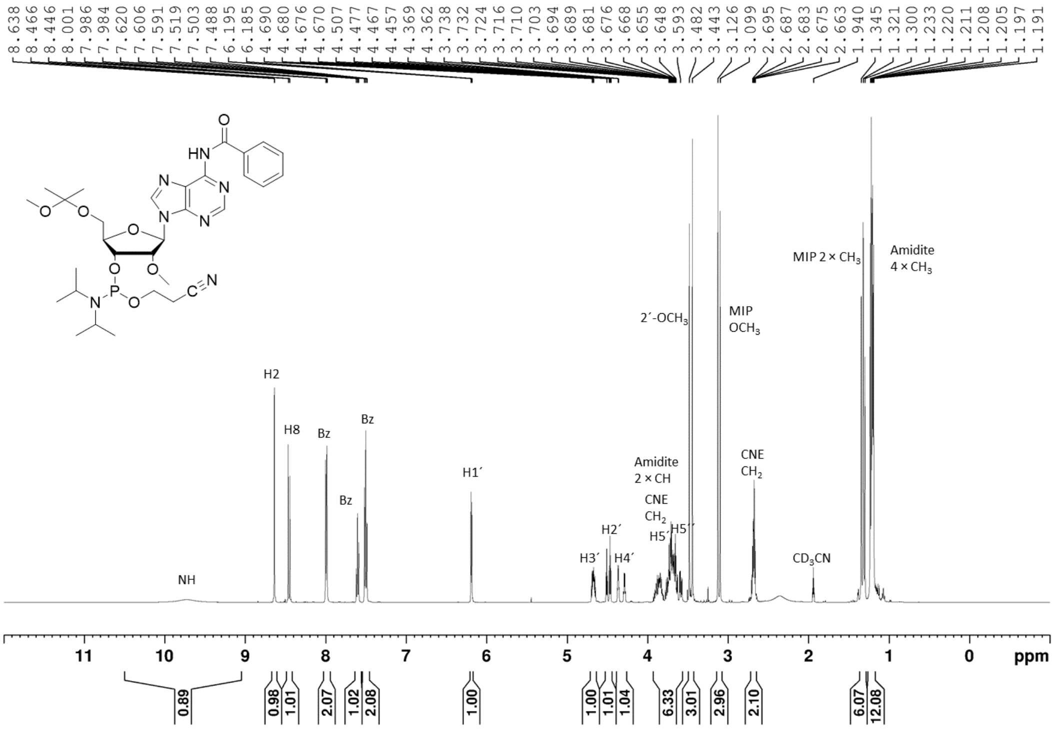

Figure S84. ^{1}H NMR (500 MHz, CD_3CN) spectrum of **10i**

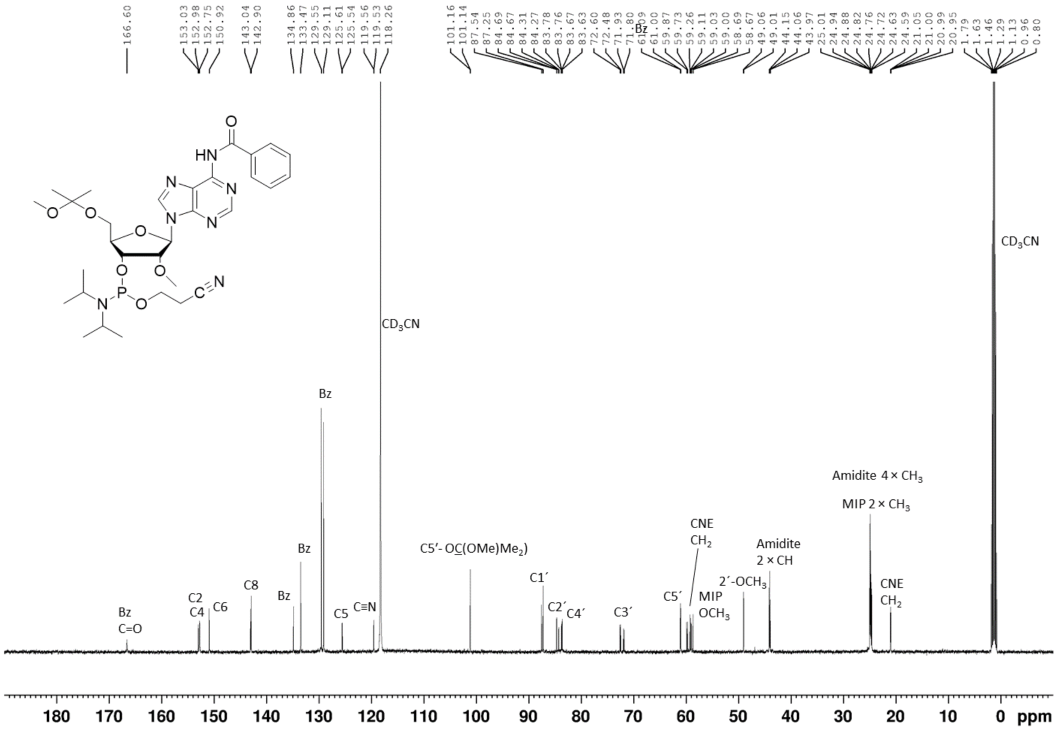

Figure S85. ^{13}C NMR (126 MHz, CD_3CN) spectrum of **10i**

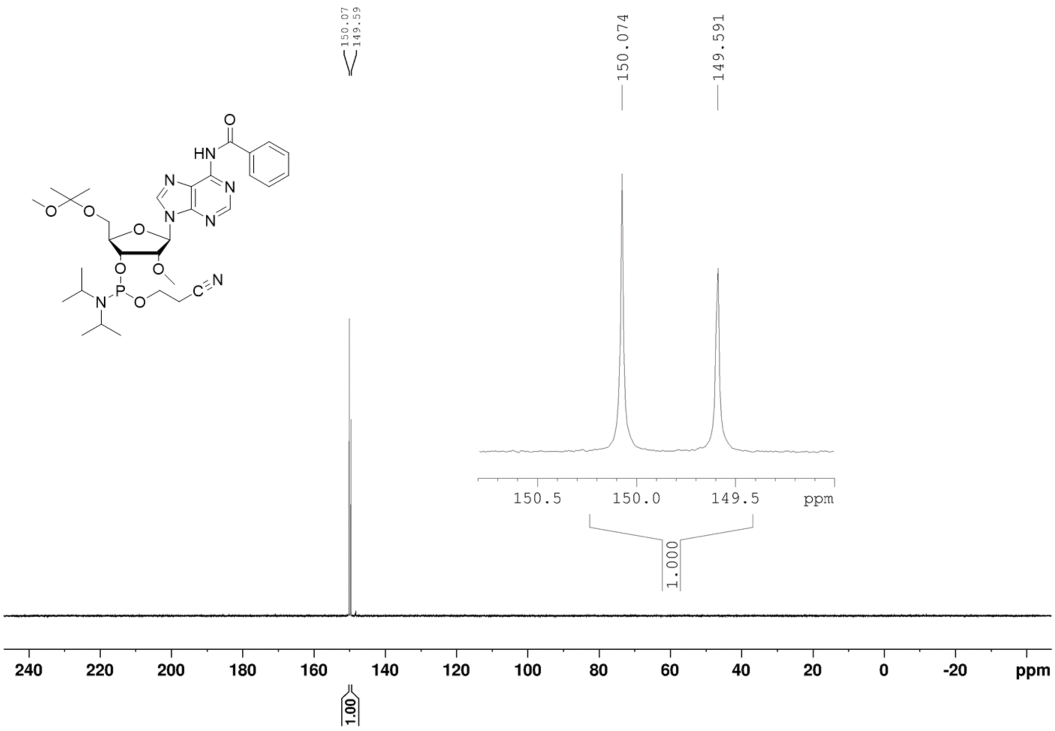

Figure S86. ^{31}P NMR (202 MHz, CD_3CN) spectrum of **10i**

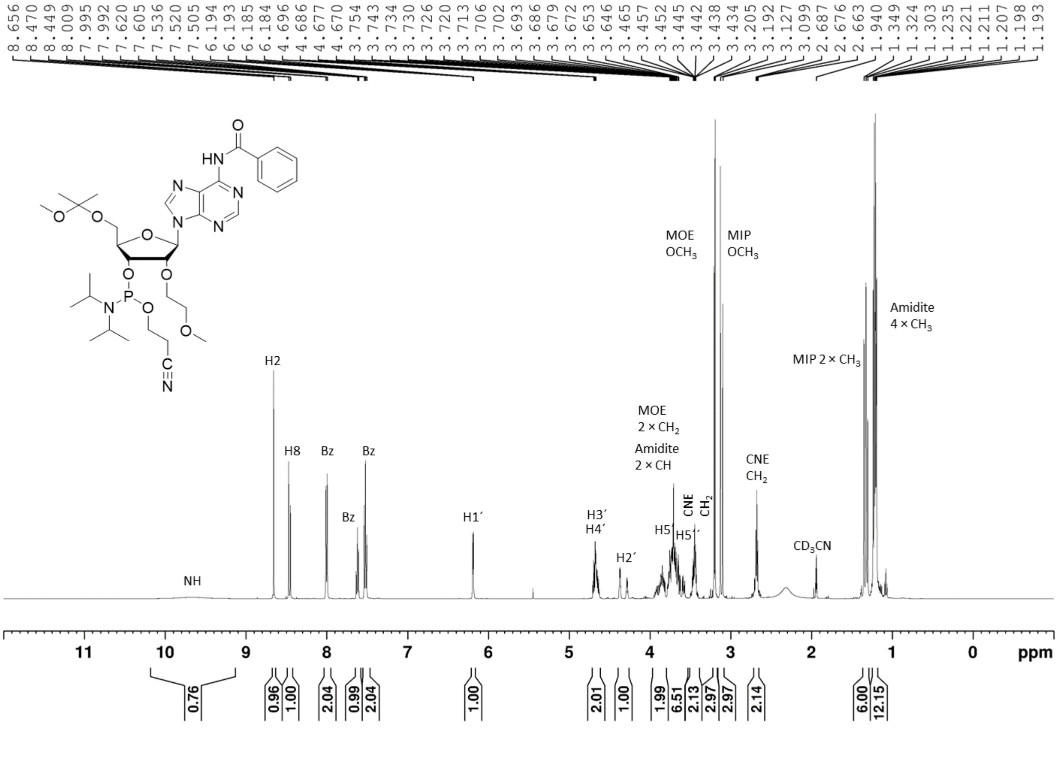

Figure S87. ^{1}H NMR (500 MHz, CD_3CN) spectrum of **10j**

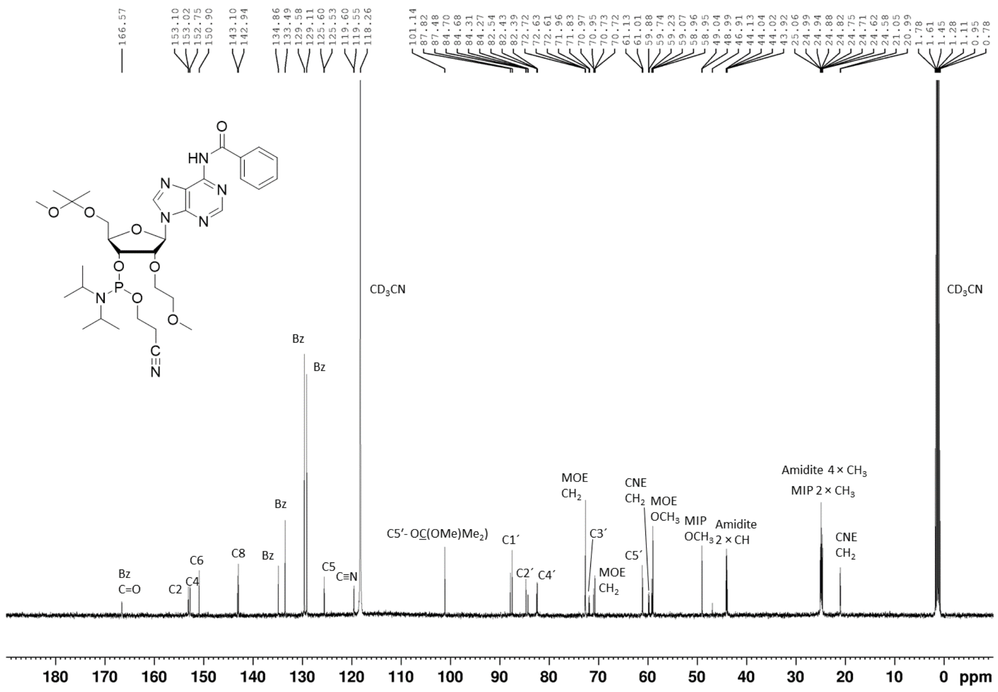

Figure S88. ^{13}C NMR (126 MHz, CD_3CN) spectrum of **10j**

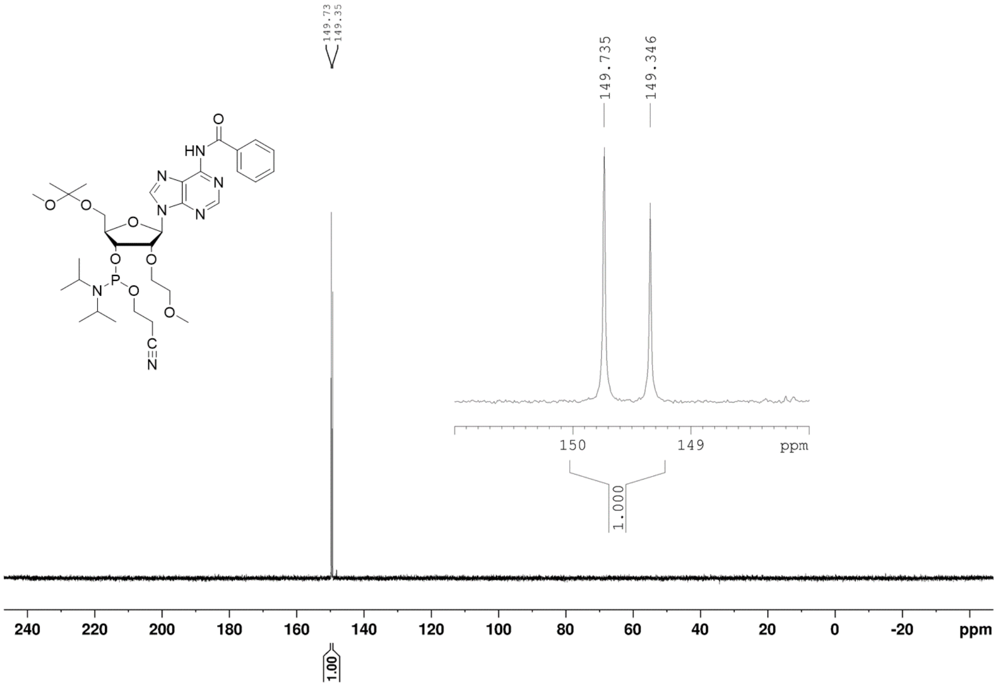

Figure S89. ^{31}P NMR (202 MHz, CD_3CN) spectrum of **10j**

NMR studies of the 2-methoxy-prop-2-yl pyridium (MIPPY) pre-association complexes

NMR studies of the formation of 2-methoxy-prop-2-yl pyridinium complexes were done in various conditions (i–vi). In all cases, the reaction mixture is prepared under nitrogen atmosphere and studied by ^{1}H, ^{13}C, HSQC, HMBC and ^{1}H-^{15}N HMBC NMR experiments at 258 K.

(i) Pyridine (65 µL, 0.80 mmol), 200 µL dried solution of *p*TsO^{-}PyH^{+} in $CDCl_3$ (130 mg/mL, 0.10 mmol), TMSCl (6 µL, 0.05 mmol, added to remove the residual water) and $CDCl_3$ (450 µL) were mixed in an NMR-tube. Reaction was initiated by adding 2-methoxypropene (20 µL, 0.20 mmol) and the tube was immediately transferred into the NMR analysis. The MIPPY-complex was observed (Figures S90–94).

(ii) Pyridine (65 µL, 0.80 mmol), 100 µL dried solution of pyridine·HCl in $CDCl_3$ (120 mg/mL, 0.10 mmol), TMSCl (6 µL, 0.05 mmol, added to remove the residual water) and $CDCl_3$ (550 µL) were mixed in an NMR-tube. Reaction was initiated by adding 2-methoxypropene (20 µL, 0.20 mmol) and the tube was immediately transferred into the NMR analysis. The MIPPY-complex was observed (Figures S95–99).

(iii) Pyridine (65 µL, 0.80 mmol), 100 µL dried solution of pyridine·HCl in $CDCl_3$ (120 mg/mL, 0.10 mmol) and $CDCl_3$ (550 µL) were mixed in the NMR-tube. Reaction was initiated by adding 2-methoxypropene (20 µL, 0.20 mmol) and the tube immediately transferred into the NMR analysis. The MIPPY-complex was observed (Figures S100–104).

(iv) Pyridine (65 µL, 0.80 mmol), trifluoromethanesulfonic anhydride (6 µL, 0.03 mmol) and $CDCl_3$ (650 µL) were mixed in an NMR-tube under nitrogen atmosphere. Reaction was initiated by adding 2-methoxypropene (20 µL, 0.20 mmol). The tube was transferred to freezer (-20 °C) for 24 h, and then to the NMR analysis. The MIPPY-complex was observed (Figures S105–109).

(v) 2,6-Lutidine (94 µL, 0.80 mmol), 200 µL dried solution of *p*TsO^{-}2,6-LuH^{+} in $CDCl_3$ (142 mg/mL, 0.10 mmol), TMSCl (6 µL, 0.05 mmol, added to remove the residual water) and $CDCl_3$ (450 µL) were mixed in an NMR-tube. Reaction was initiated by adding 2-methoxypropene (20 µL, 0.20 mmol) and the tube immediately transferred into the NMR analysis. The pre-association complex with 2,6-lutidine was not observed (Figures S110–114).

(vi) 2,4,6-Collidine (108 µL, 0.80 mmol), 200 µL dried solution of *p*TsO^{-}2,4,6-CoH^{+} in $CDCl_3$ (149 mg/mL, 0.10 mmol), TMSCl (6 µL, 0.05 mmol, added to remove the residual water) and $CDCl_3$ (450 µL) were mixed in an NMR-tube. Reaction was initiated by adding 2-methoxypropene (20 µL, 0.20 mmol) and the tube was immediately transferred into the NMR analysis. The pre-association complex with 2,4,6-collidine was not observed (Figures S115–119).

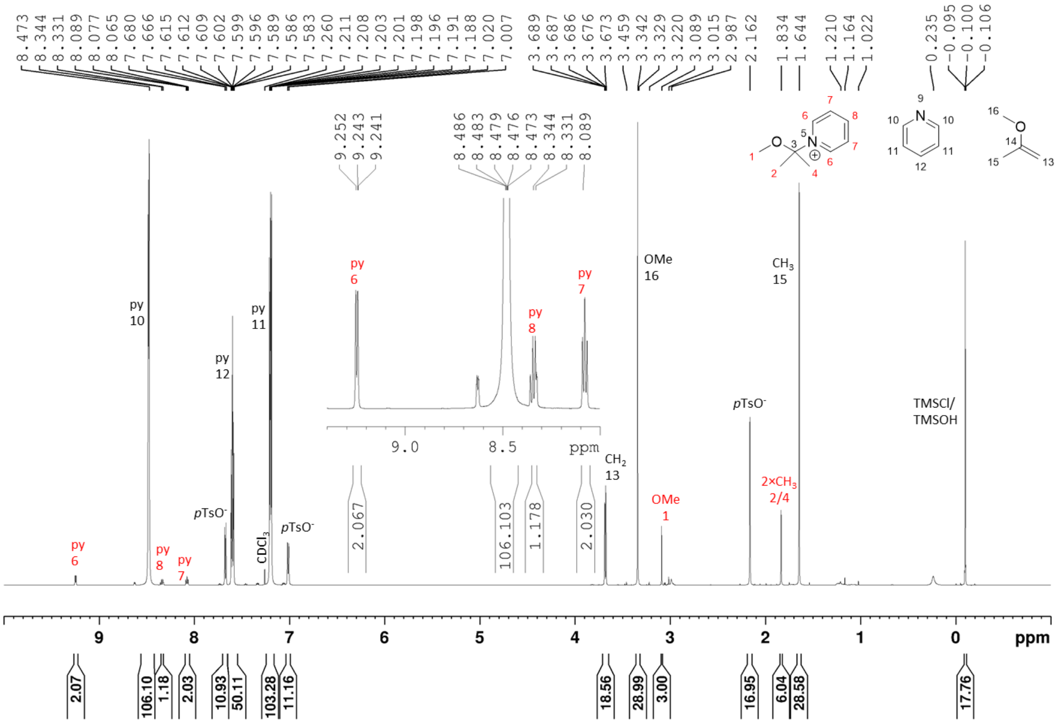

Figure S90. ^{1}H NMR (600 MHz, $CDCl_3$) spectrum of the reaction mixture (i)

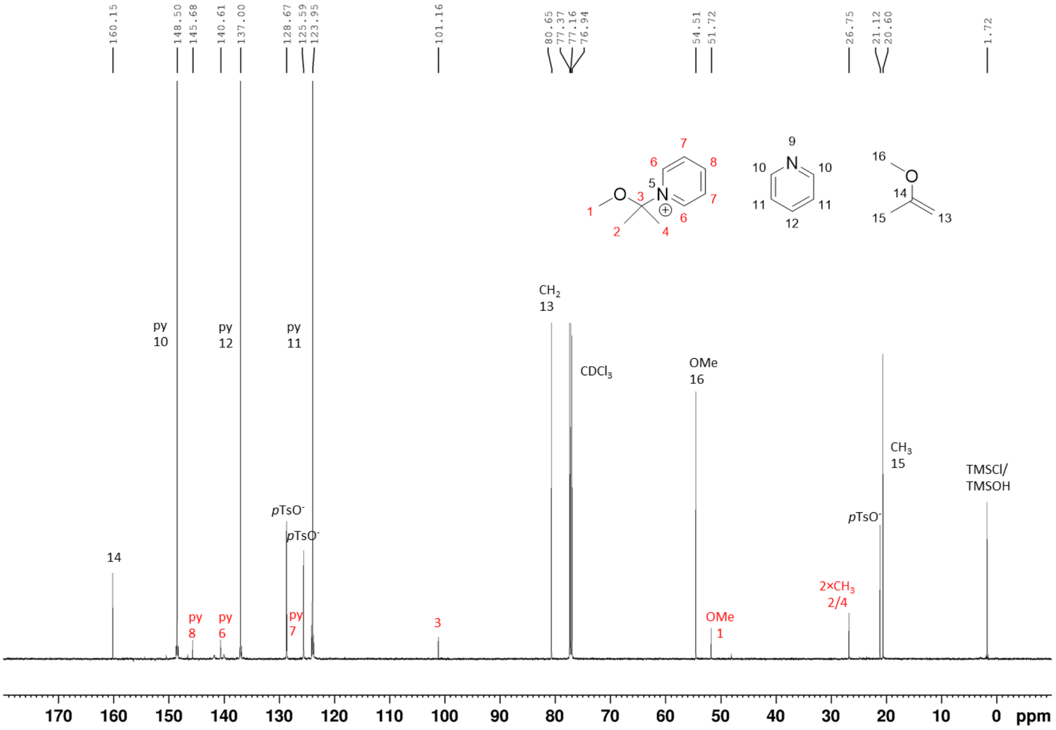

Figure S91. ^{13}C NMR (151 MHz, $CDCl_3$) spectrum of the reaction mixture (i)

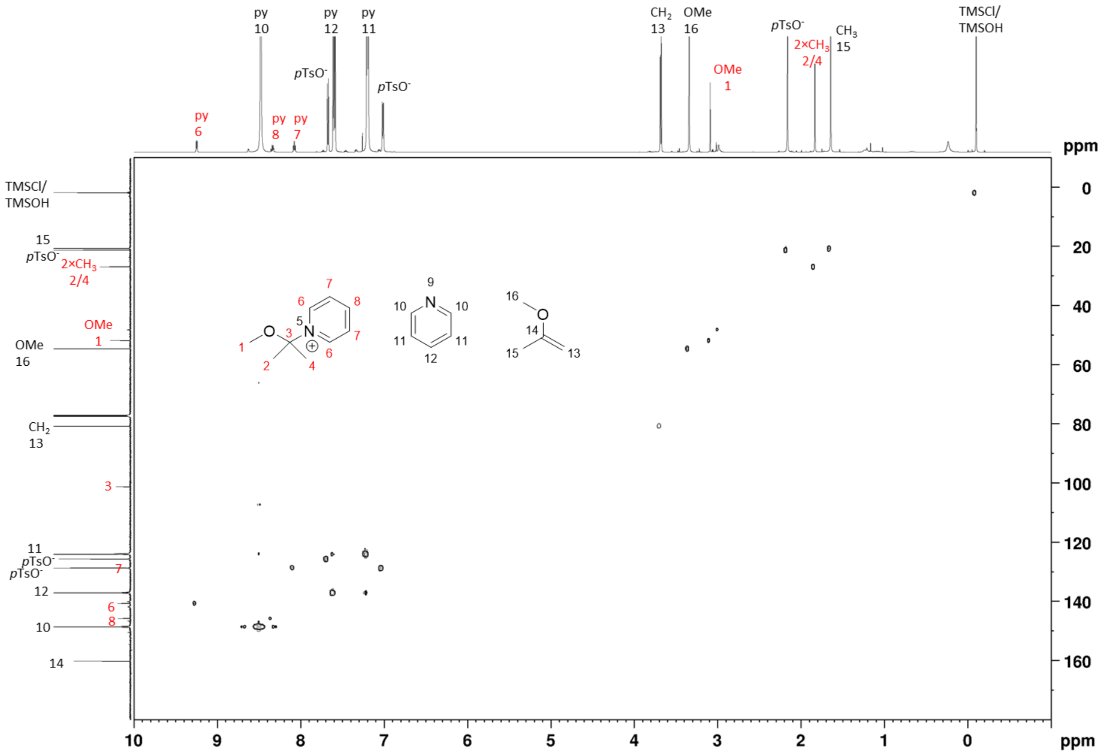

Figure S92. HSQC ($CDCl_3$) spectrum of the reaction mixture (i)

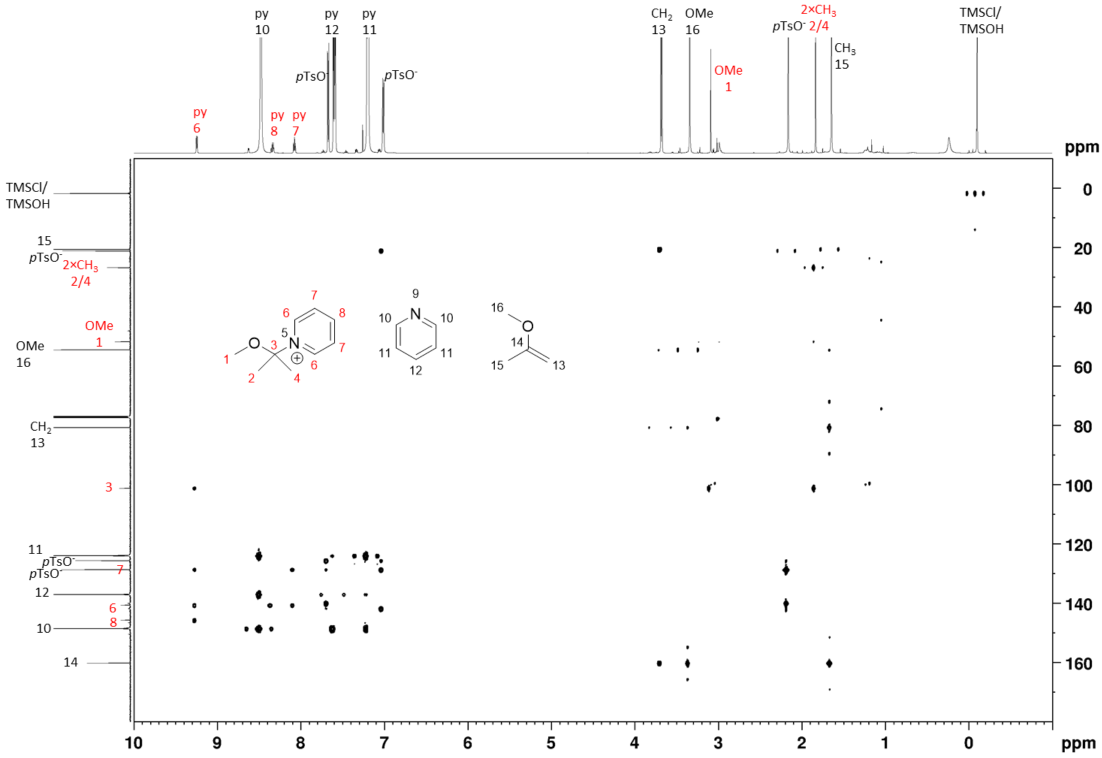

Figure S93. HMBC ($CDCl_3$) spectrum of the reaction mixture (i)

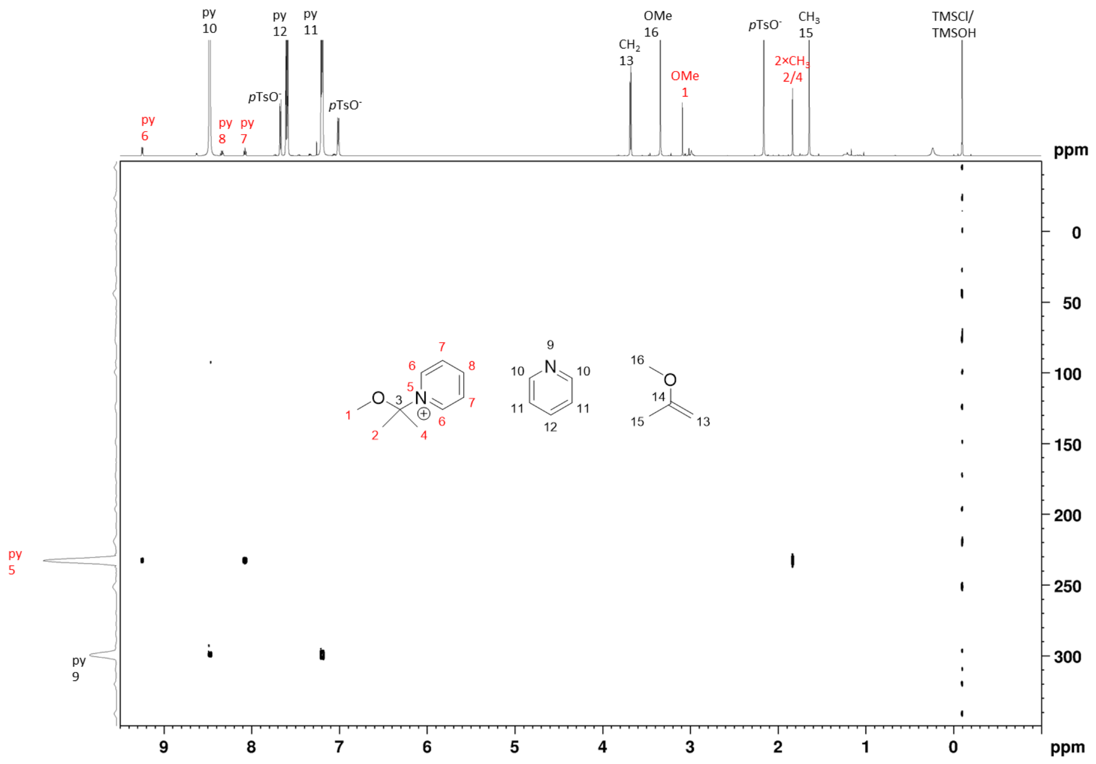

Figure S94. ^{1}H-^{15}N HMBC (600 and 61 MHz, CDCl$_3$) spectrum of the reaction mixture (i)

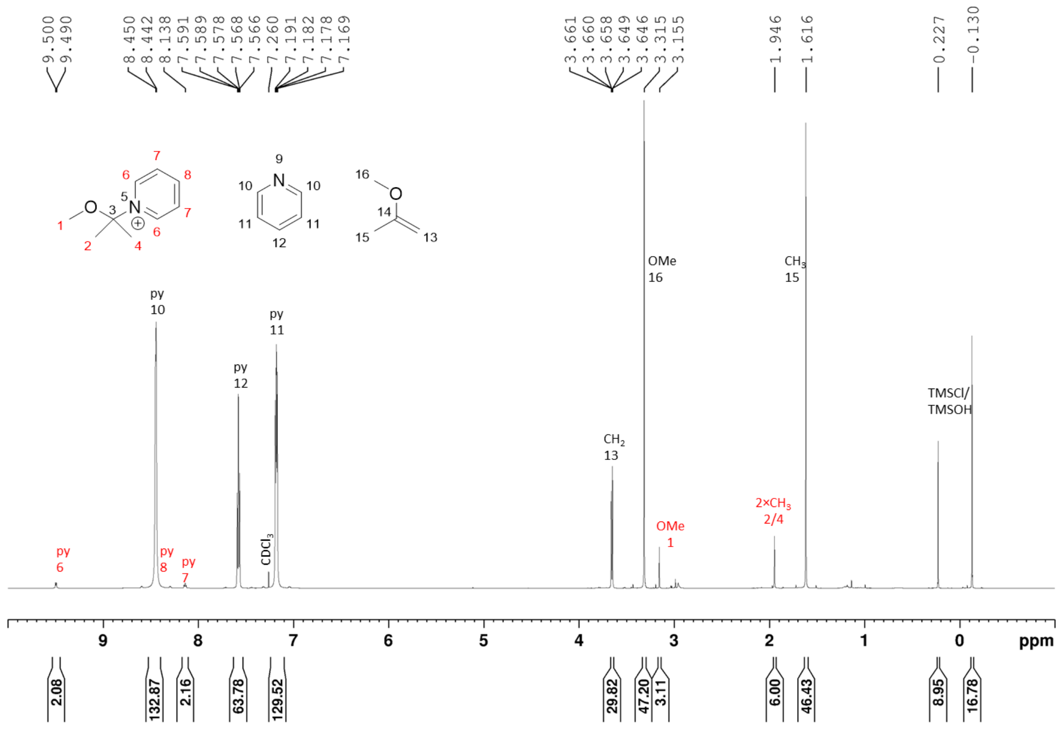

Figure S95. ^{1}H NMR (600 MHz, CDCl$_3$) spectrum of the reaction mixture (ii)

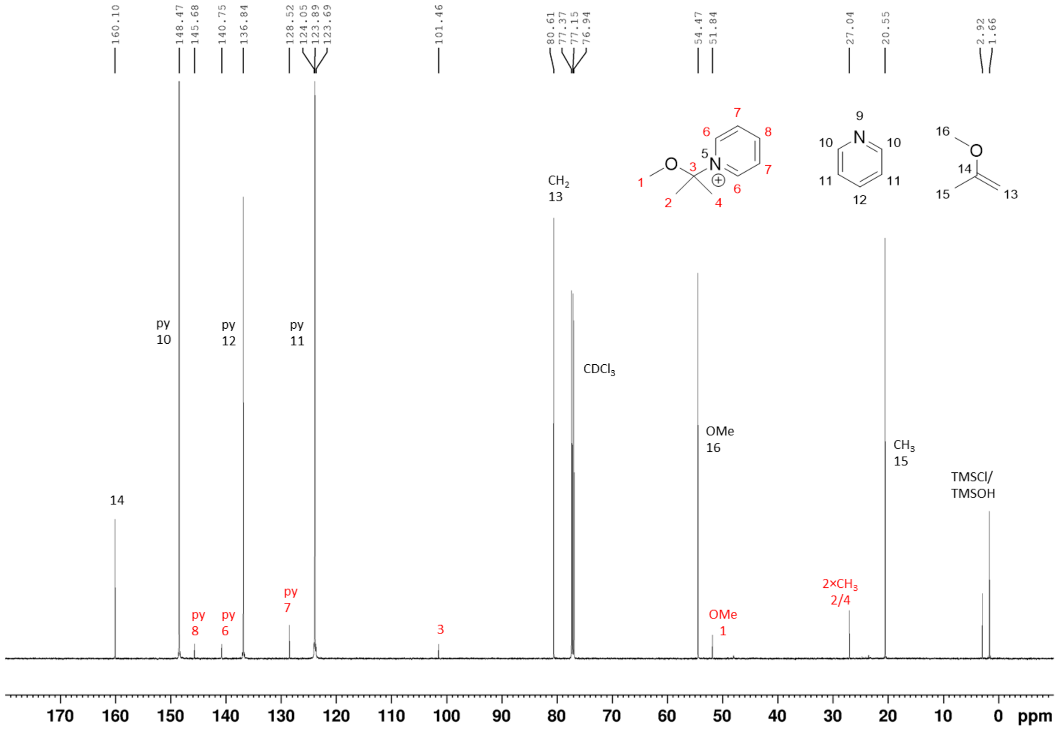

Figure S96. ^{13}C NMR (151 MHz, $CDCl_3$) spectrum of the reaction mixture (ii)

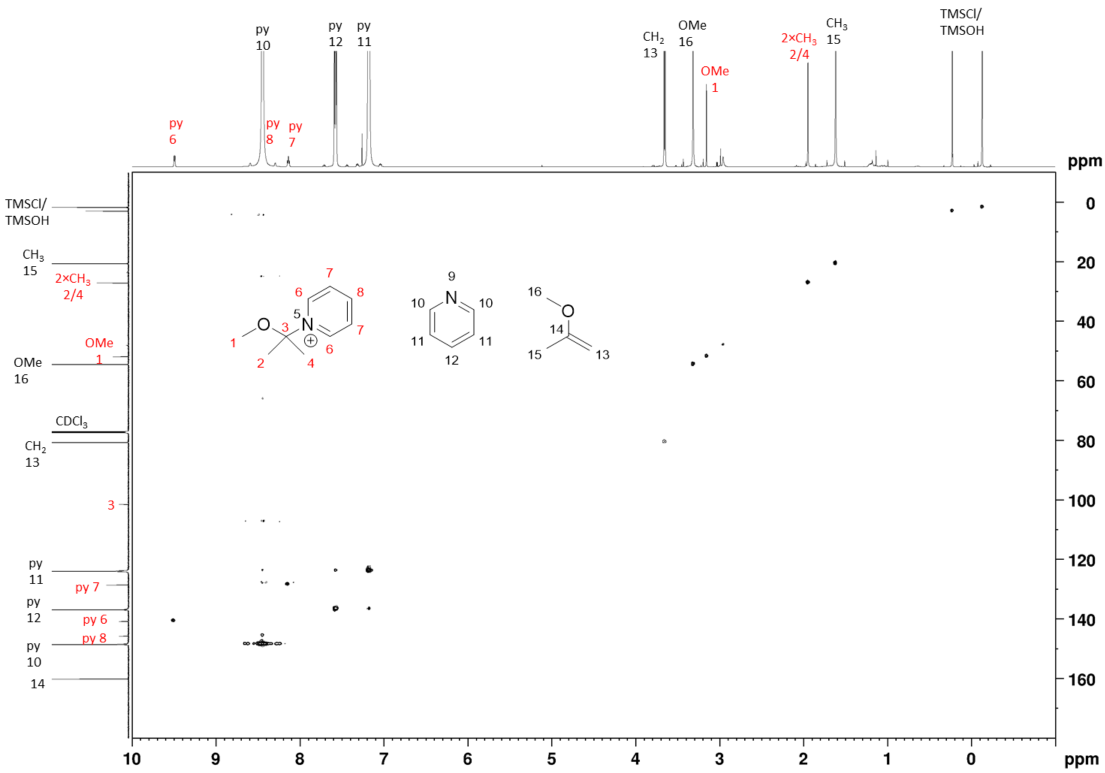

Figure S97. HSQC ($CDCl_3$) spectrum of the reaction mixture (ii)

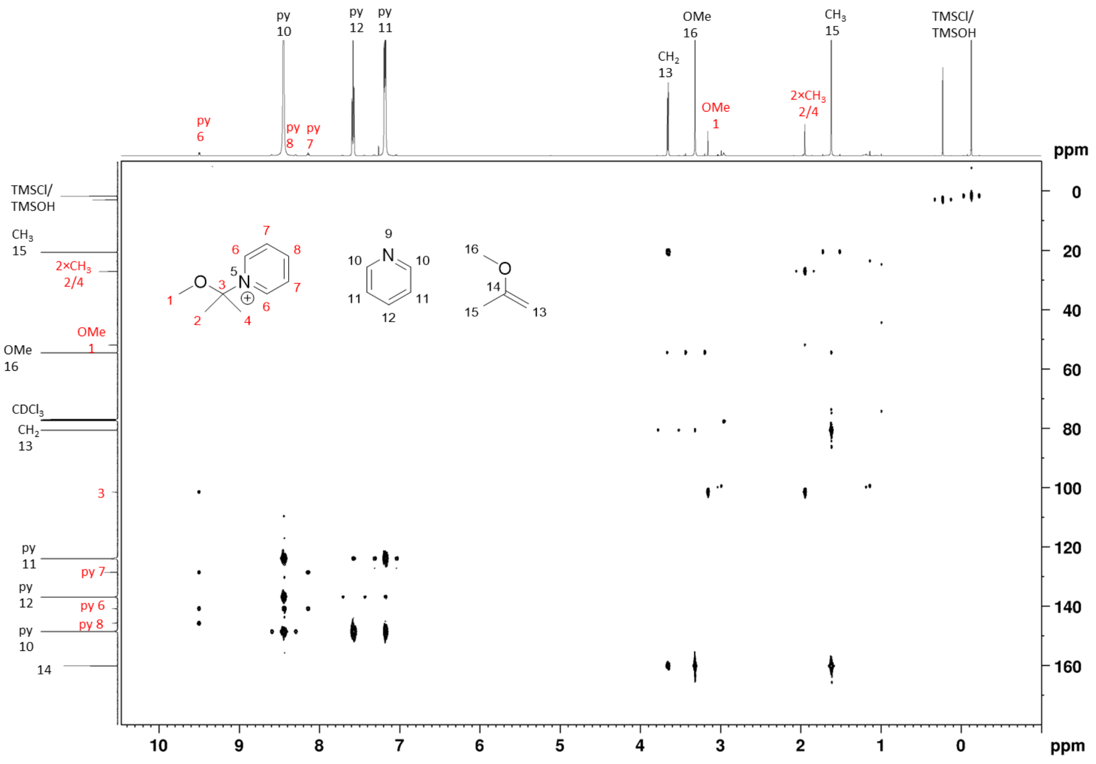

Figure S98. HMBC ($CDCl_3$) spectrum of the reaction mixture (ii)

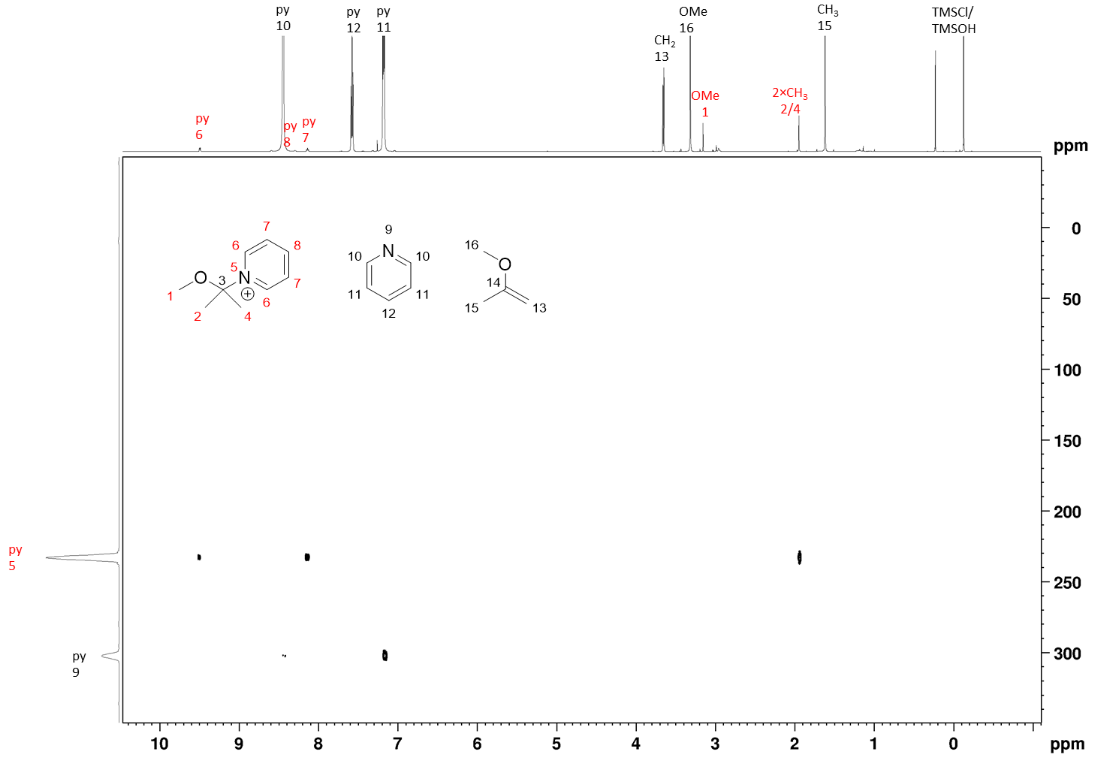

Figure S99. ^{1}H-^{15}N HMBC (600 and 61 MHz, $CDCl_3$) spectrum of the reaction mixture (ii)

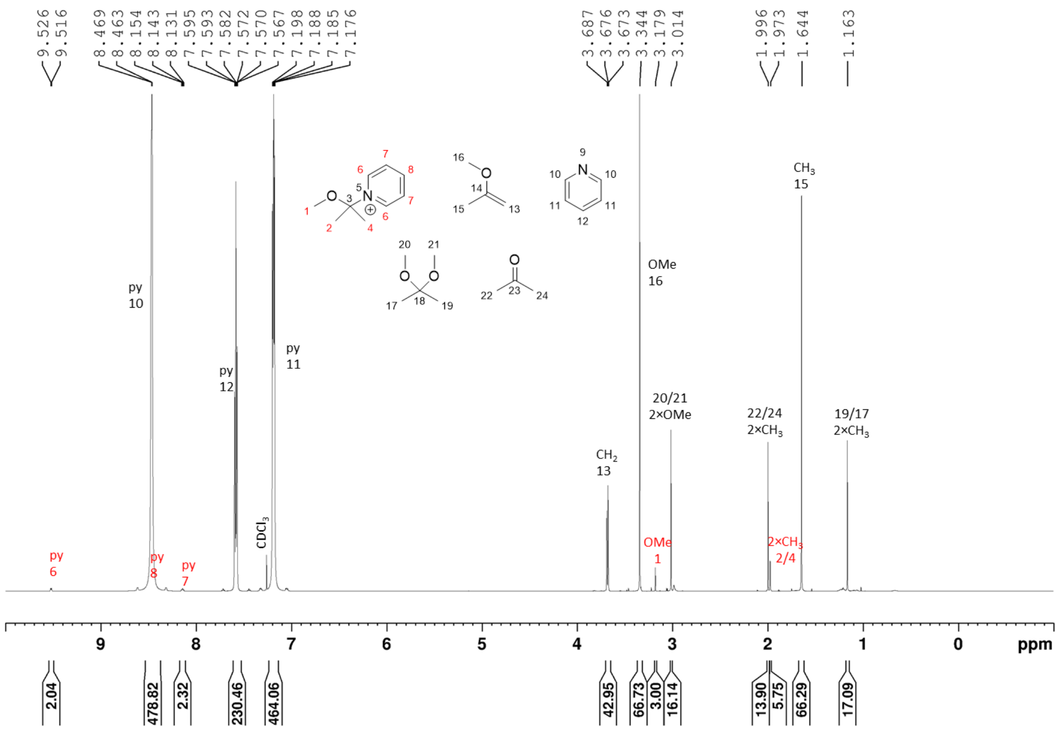

Figure S100. ^{1}H NMR (600 MHz, $CDCl_3$) spectrum of the reaction mixture (iii)

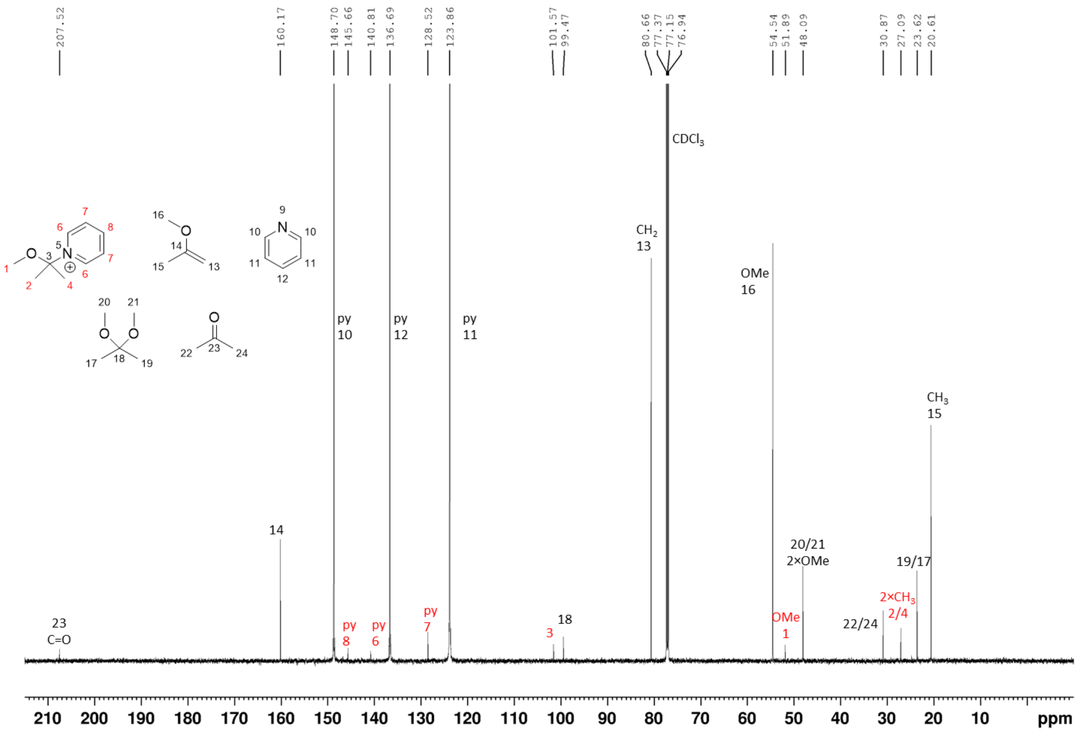

Figure S101. ^{13}C NMR (151 MHz, $CDCl_3$) spectrum of the reaction mixture (iii)

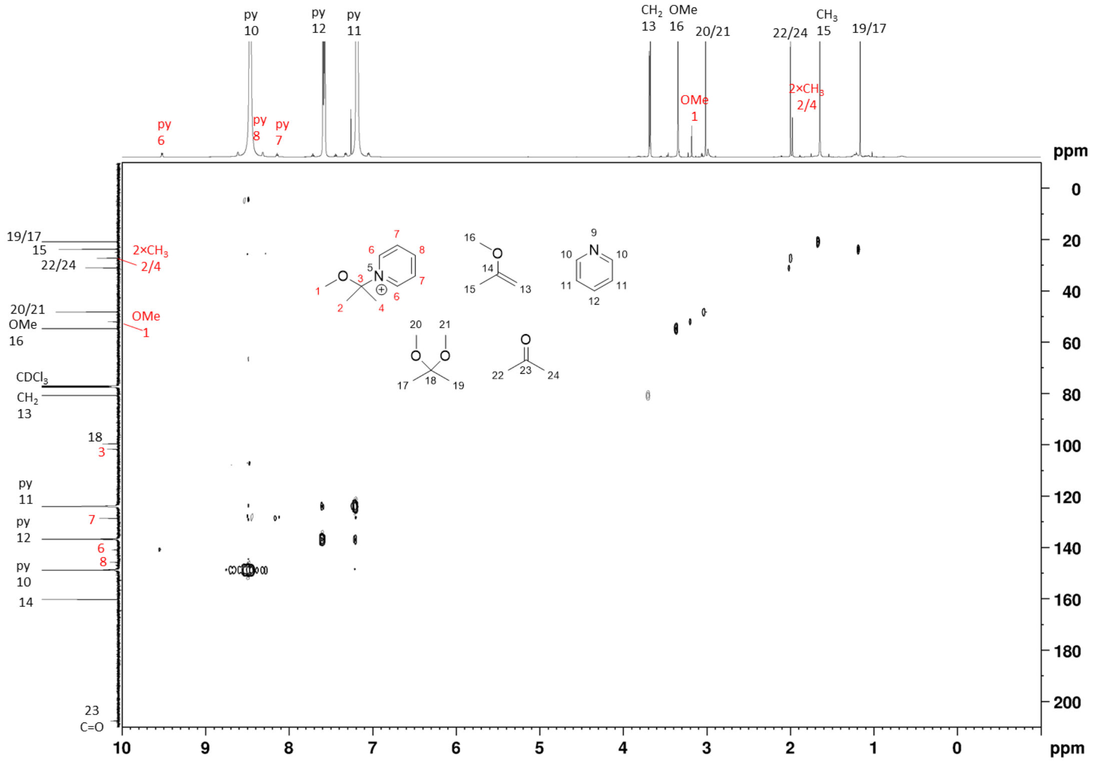

Figure S102. HSQC ($CDCl_3$) spectrum of the reaction mixture (iii)

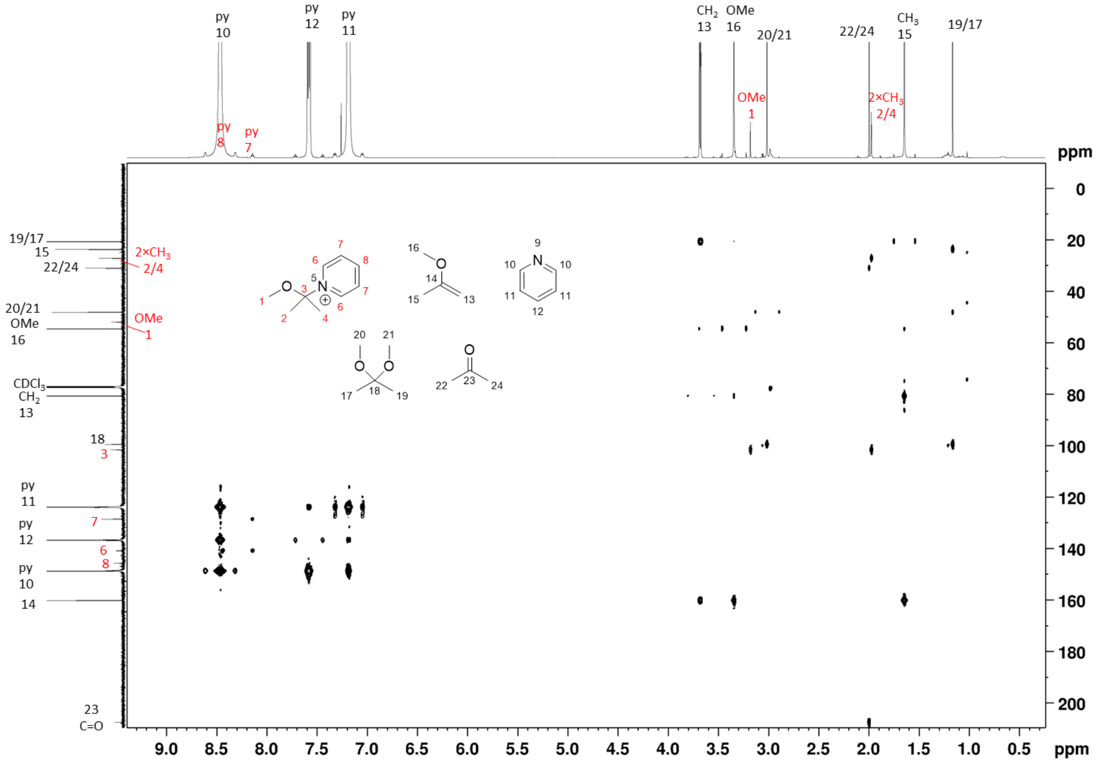

Figure S103. HMBC ($CDCl_3$) spectrum of the reaction mixture (iii)

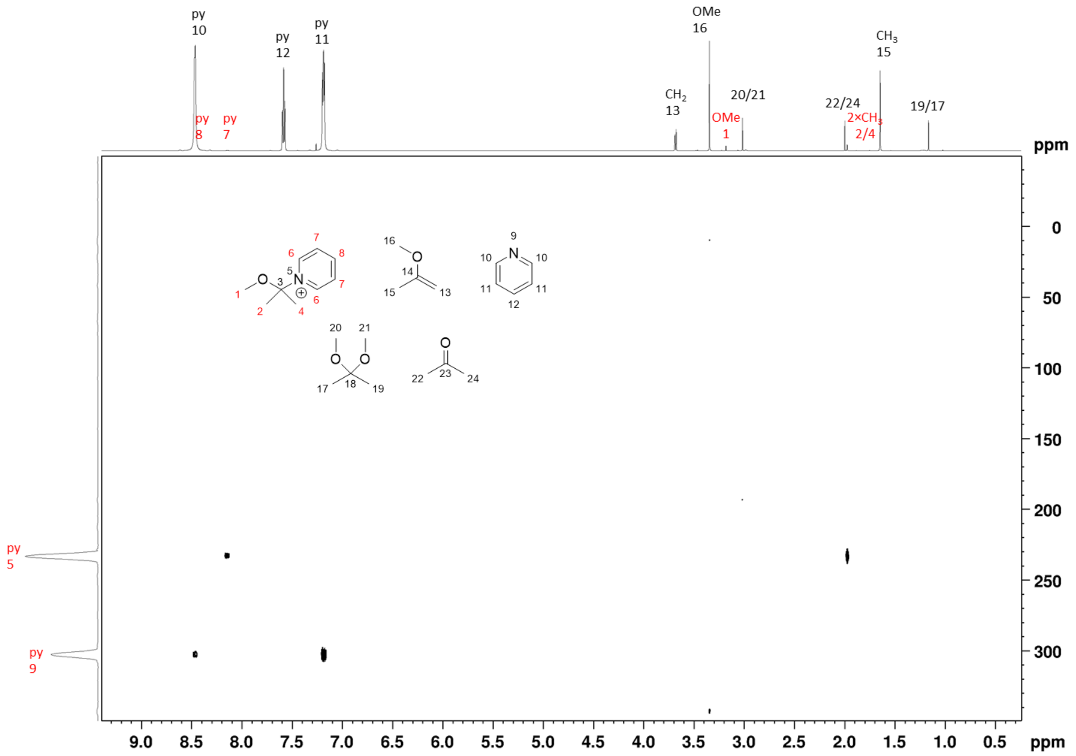

Figure S104. ^{1}H-^{15}N HMBC (600 and 61 MHz, $CDCl_3$) spectrum of the reaction mixture (iii)

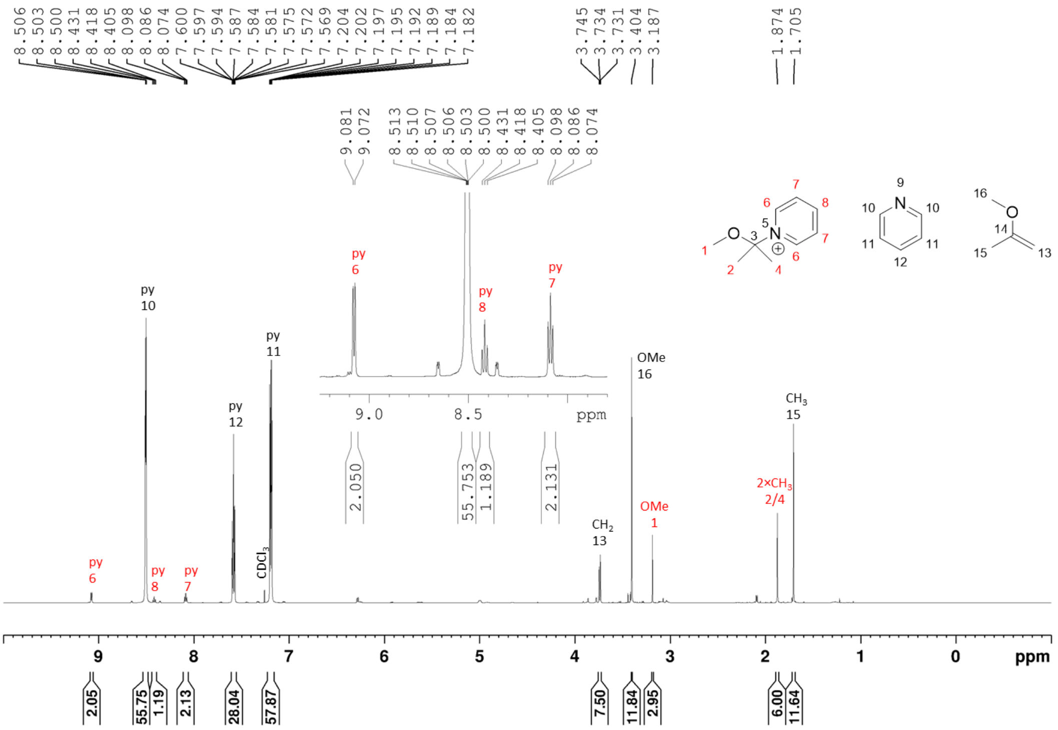

Figure S105. ^{1}H NMR (600 MHz, $CDCl_3$) spectrum of the reaction mixture (iv)

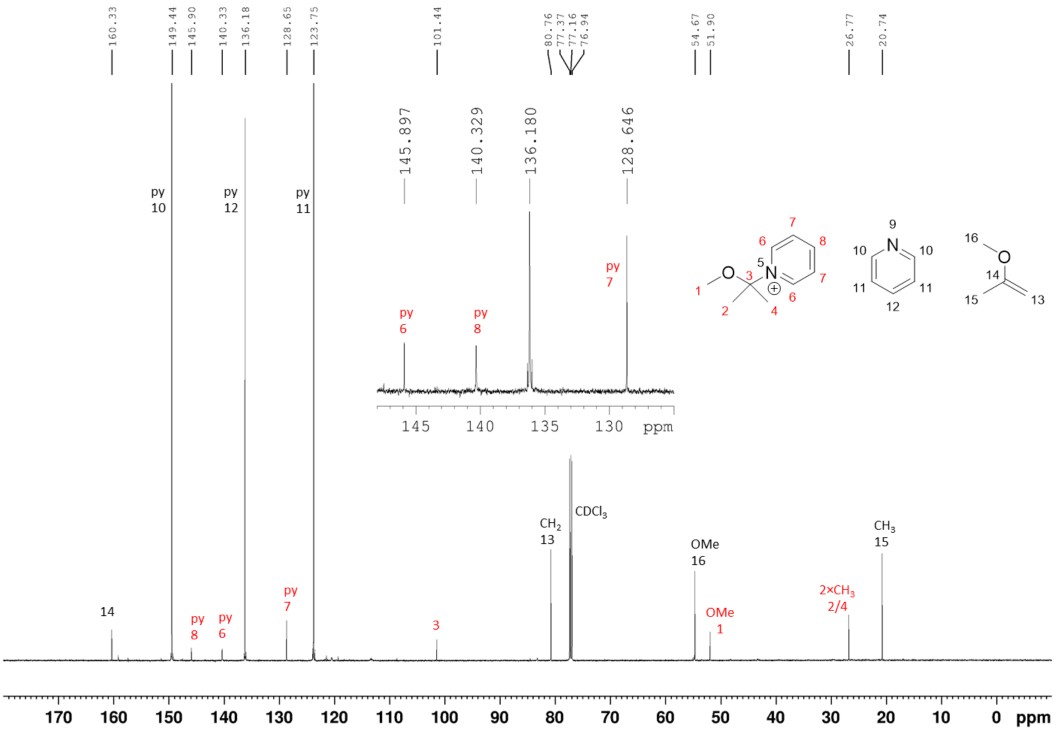

Figure S106. ^{13}C NMR (151 MHz, $CDCl_3$) spectrum of the reaction mixture (iv)

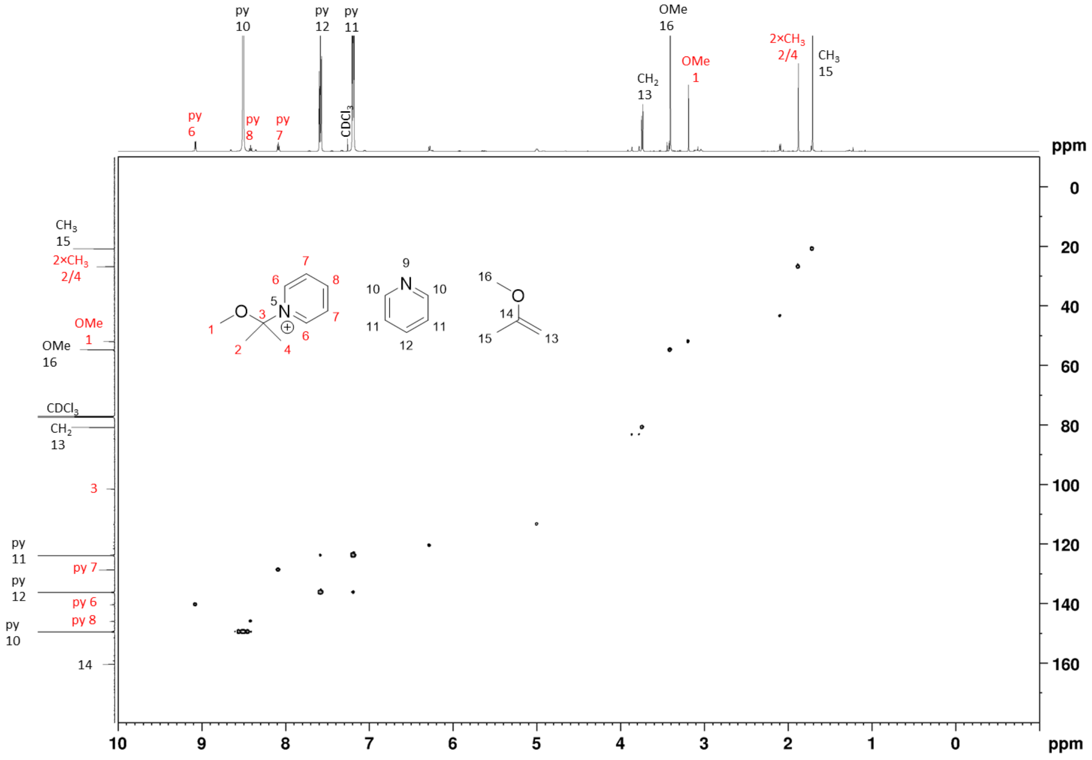

Figure S107. HSQC ($CDCl_3$) spectrum of the reaction mixture (iv)

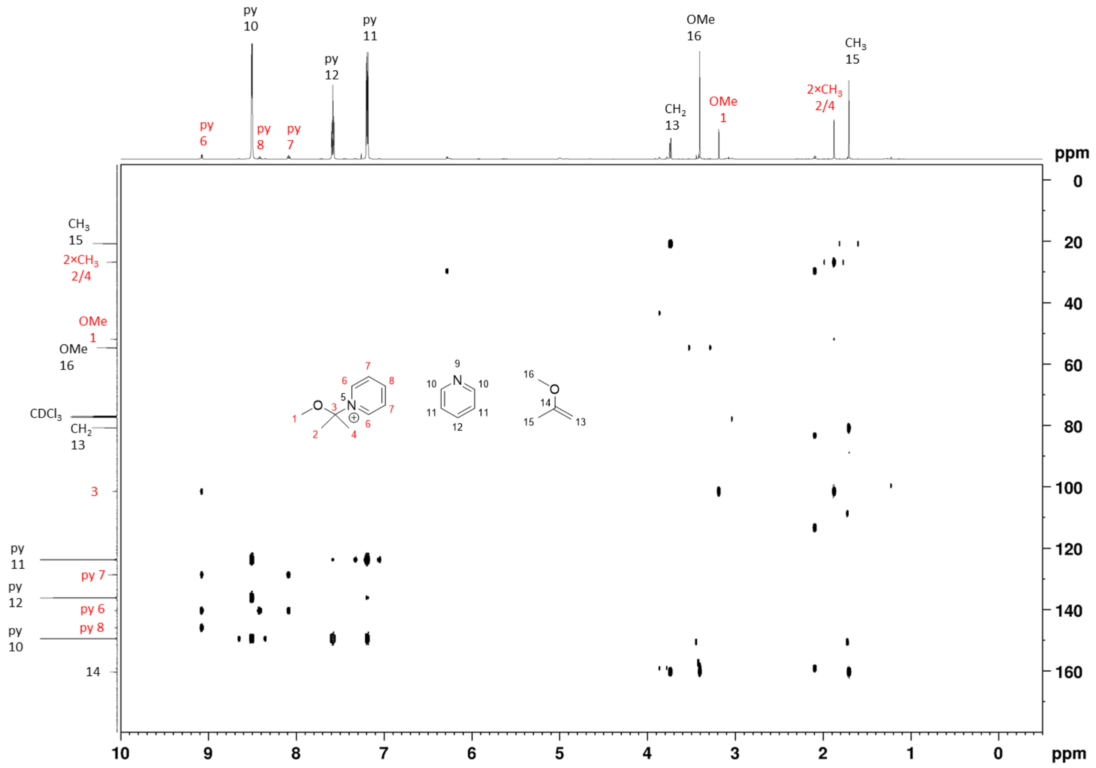

Figure S108. HMBC ($CDCl_3$) spectrum of the reaction mixture (iv)

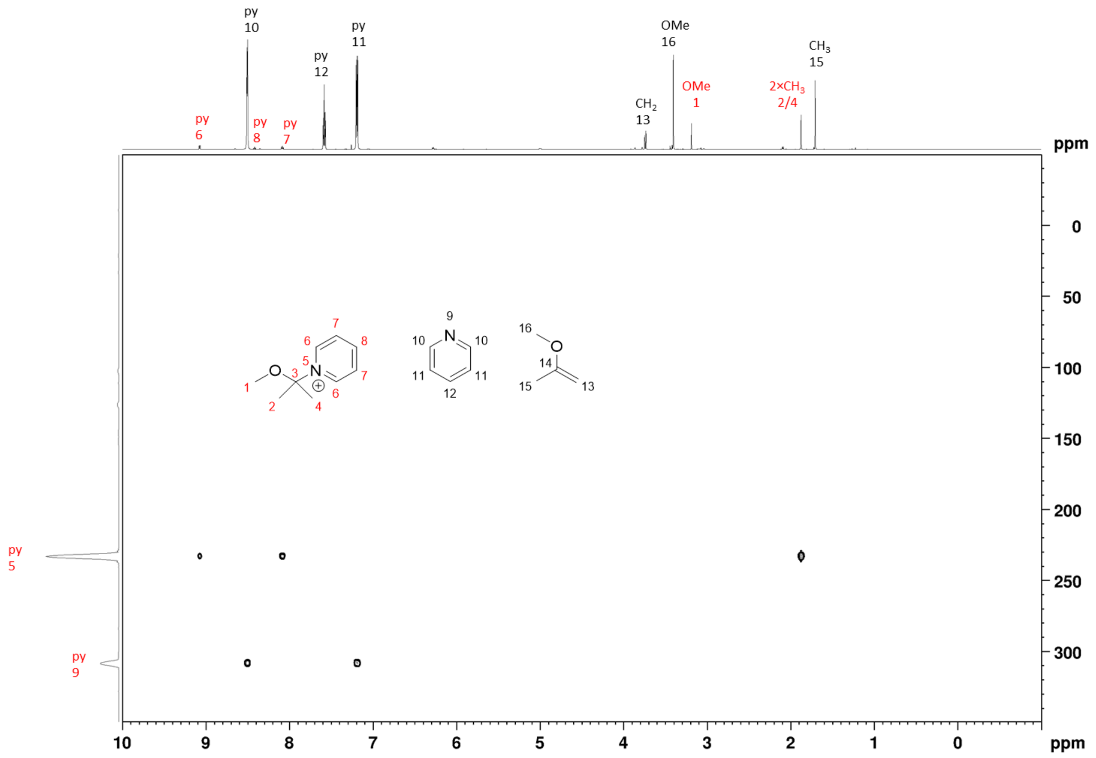

Figure S109. ^{1}H-^{15}N HMBC (600 and 61 MHz, $CDCl_3$) spectrum of the reaction mixture (iv)

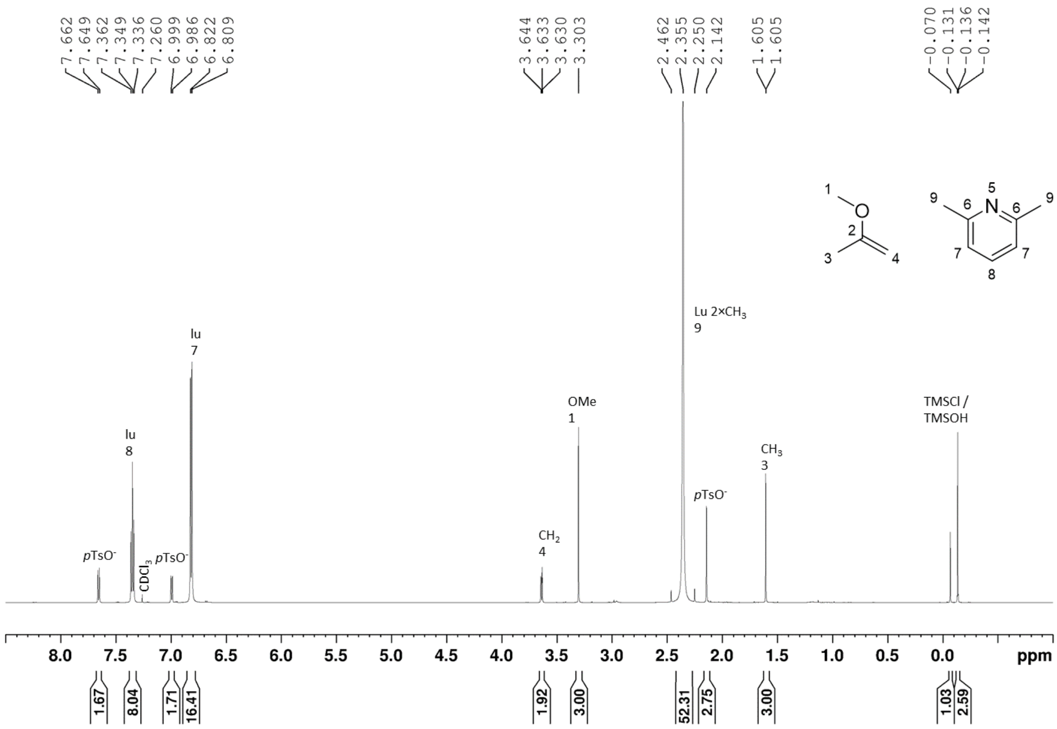

Figure S110. ^{1}H NMR (600 MHz, $CDCl_3$) spectrum of the reaction mixture (v)

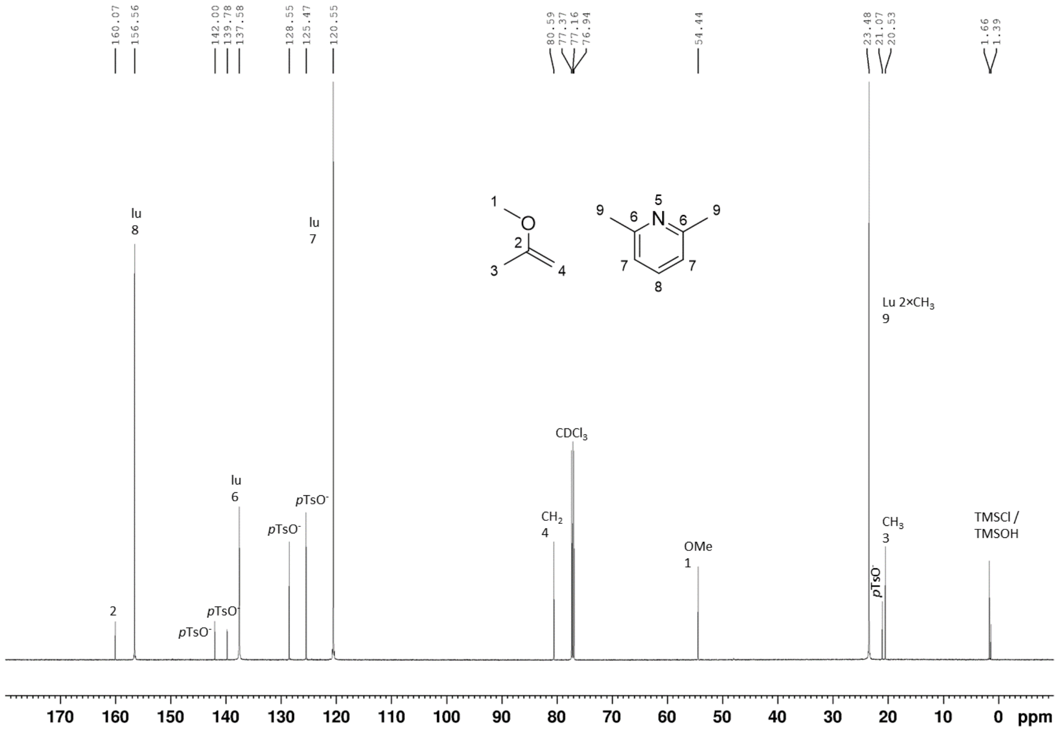

Figure S111. ^{13}C NMR (151 MHz, $CDCl_3$) spectrum of the reaction mixture (v)

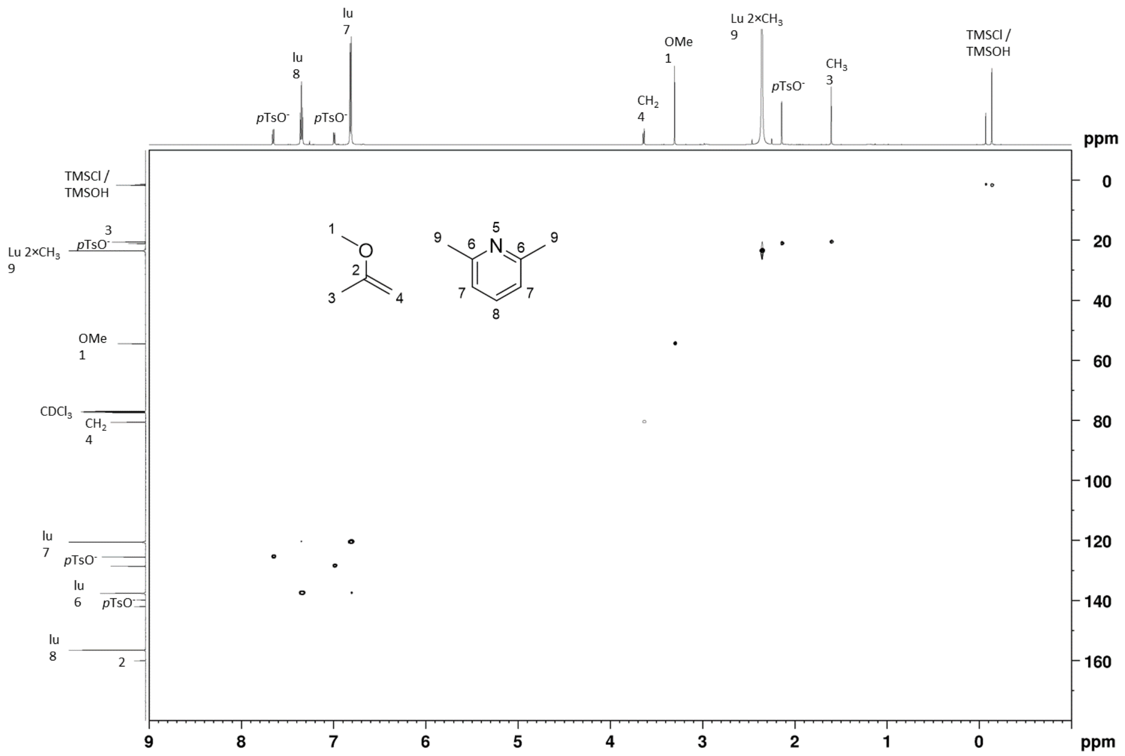

Figure S112. HSQC ($CDCl_3$) spectrum of the reaction mixture (v)

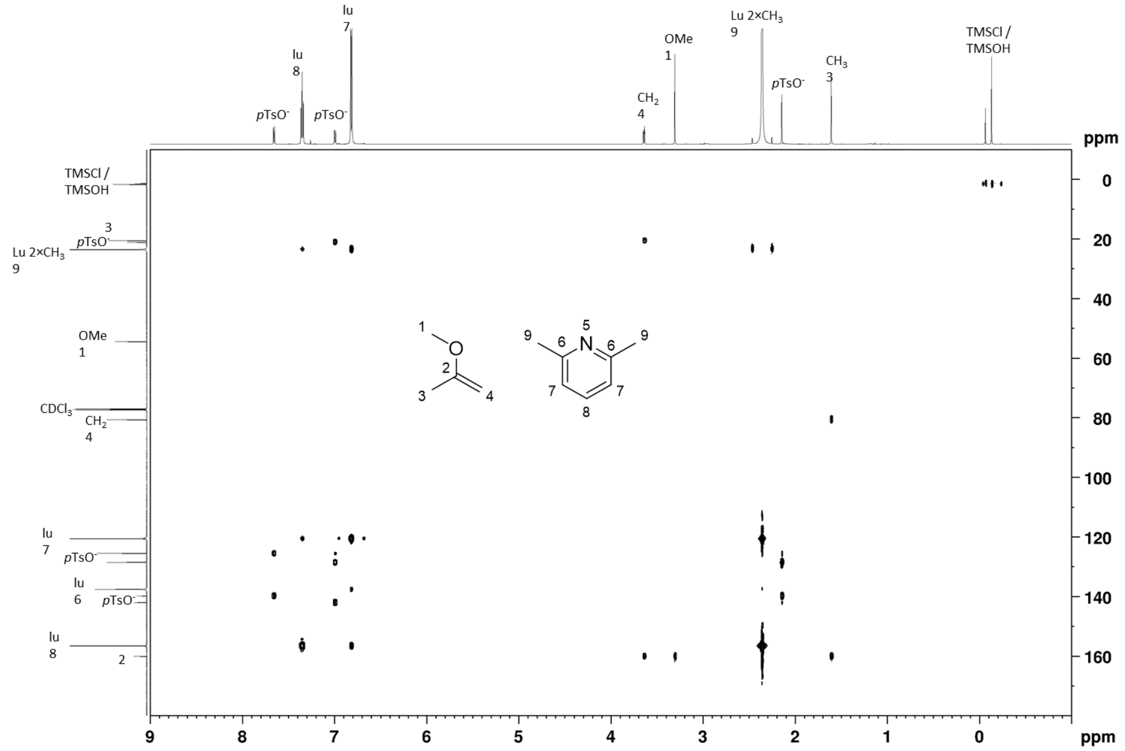

Figure S113. HMBC ($CDCl_3$) spectrum of the reaction mixture (v)

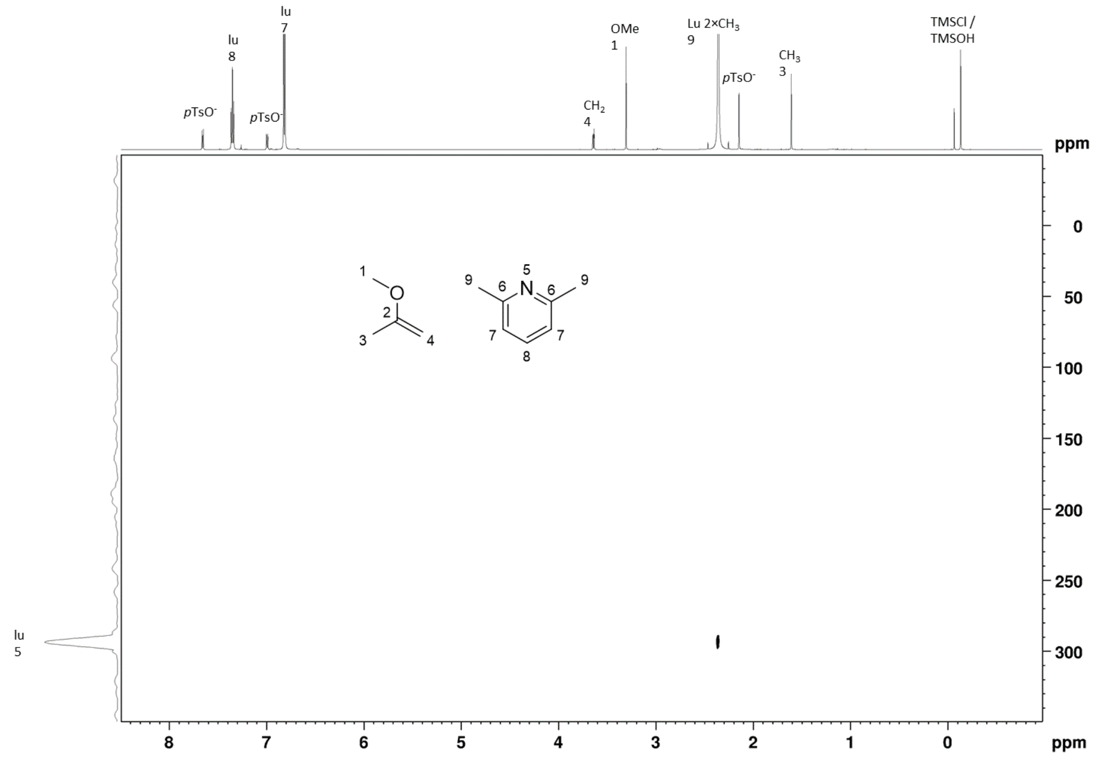

Figure S114. ^{1}H-^{15}N HMBC (600 and 61 MHz, $CDCl_3$) spectrum of the reaction mixture (v)

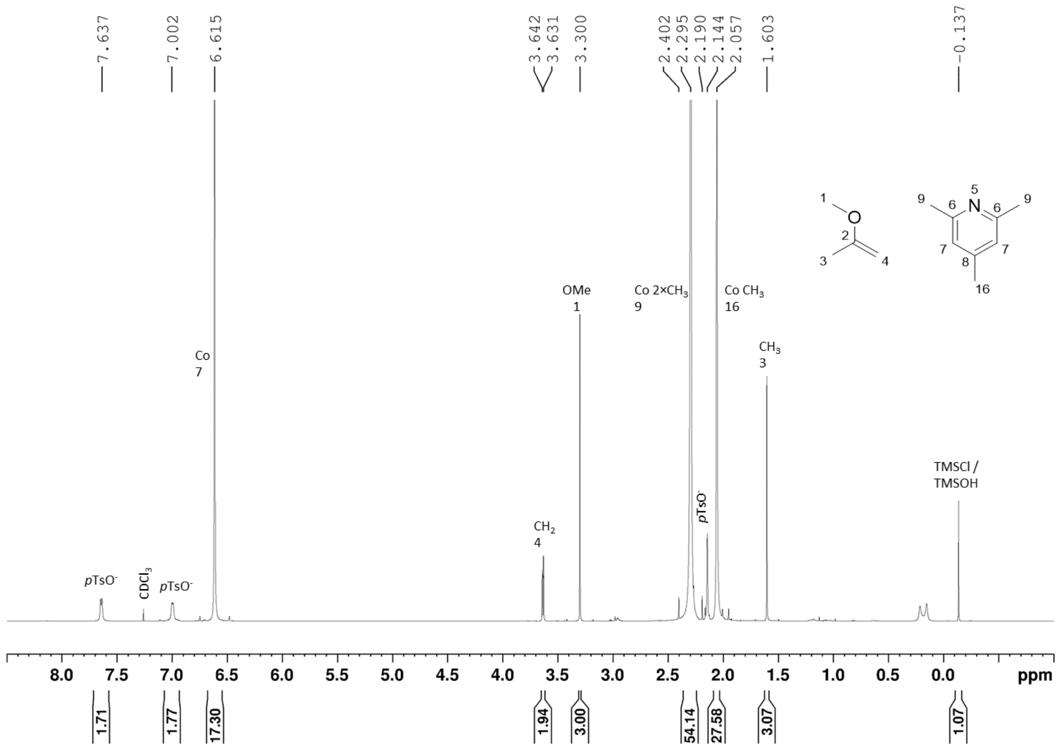

Figure S115. ^{1}H NMR (600 MHz, $CDCl_3$) spectrum of the reaction mixture (vi)

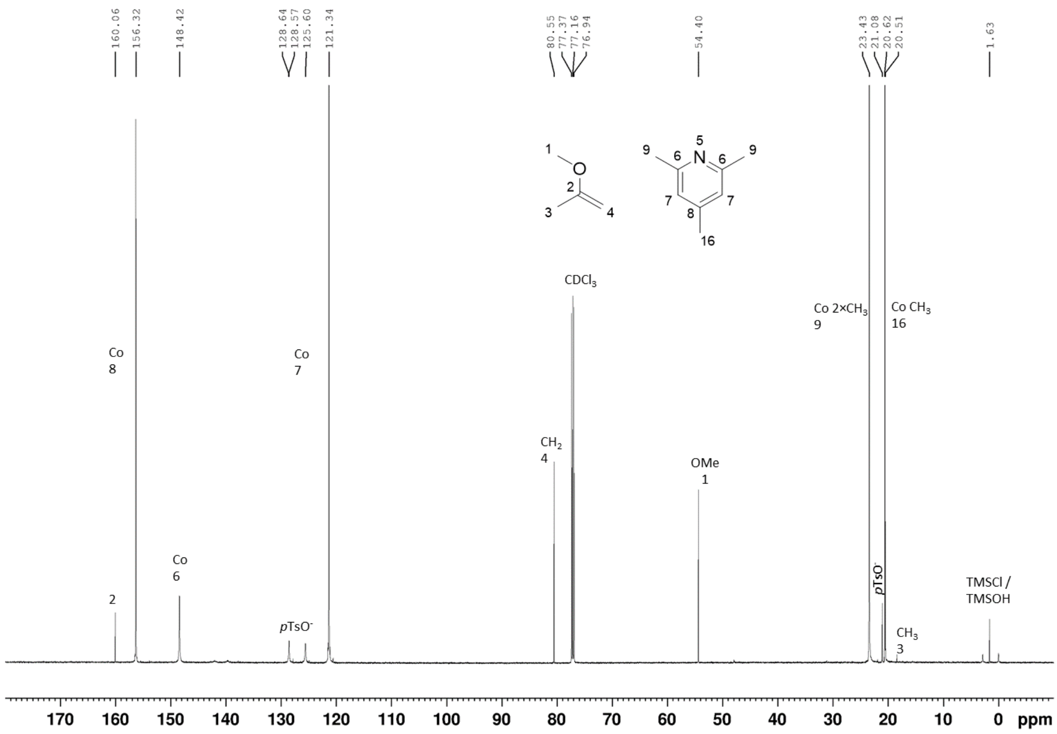

Figure S116. ^{13}C NMR (151 MHz, $CDCl_3$) spectrum of the reaction mixture (vi)

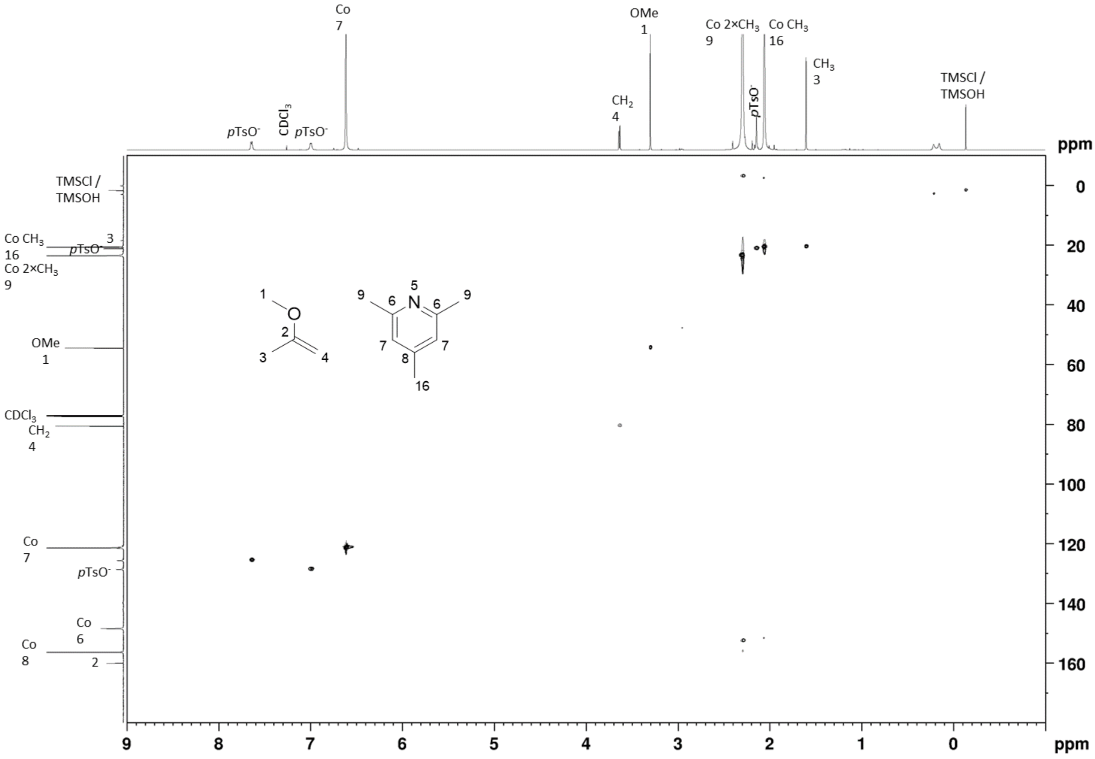

Figure S117. HSQC ($CDCl_3$) spectrum of the reaction mixture (vi)

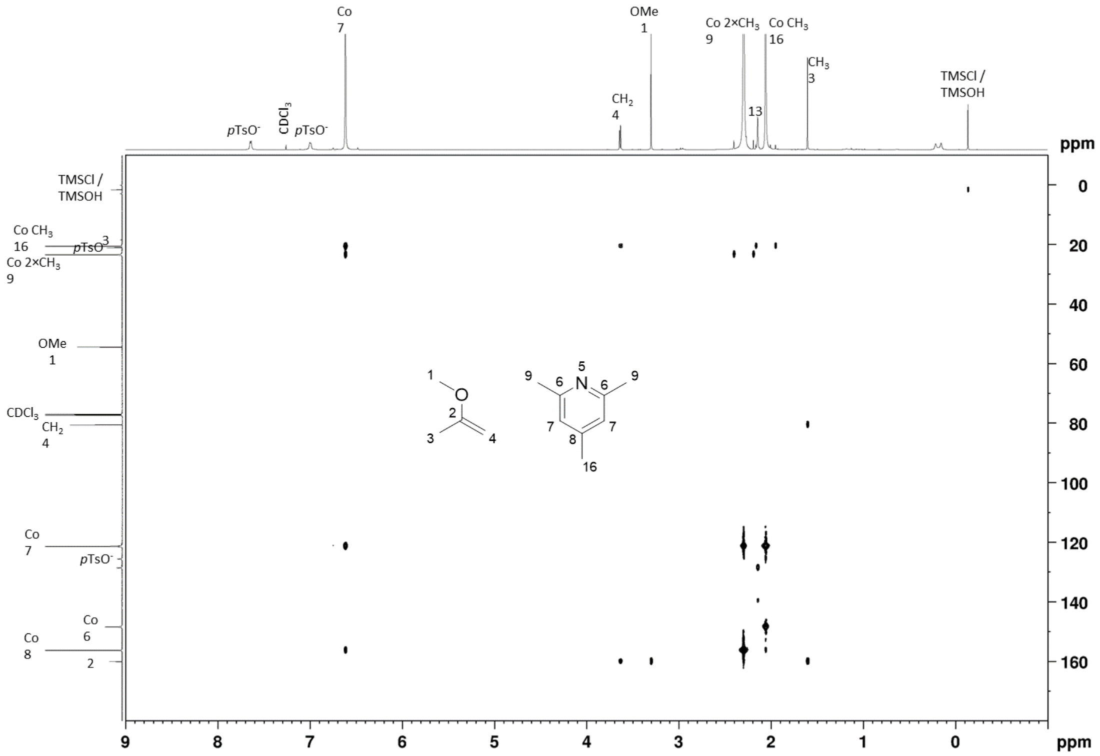

Figure S118. HMBC ($CDCl_3$) spectrum of the reaction mixture (vi)

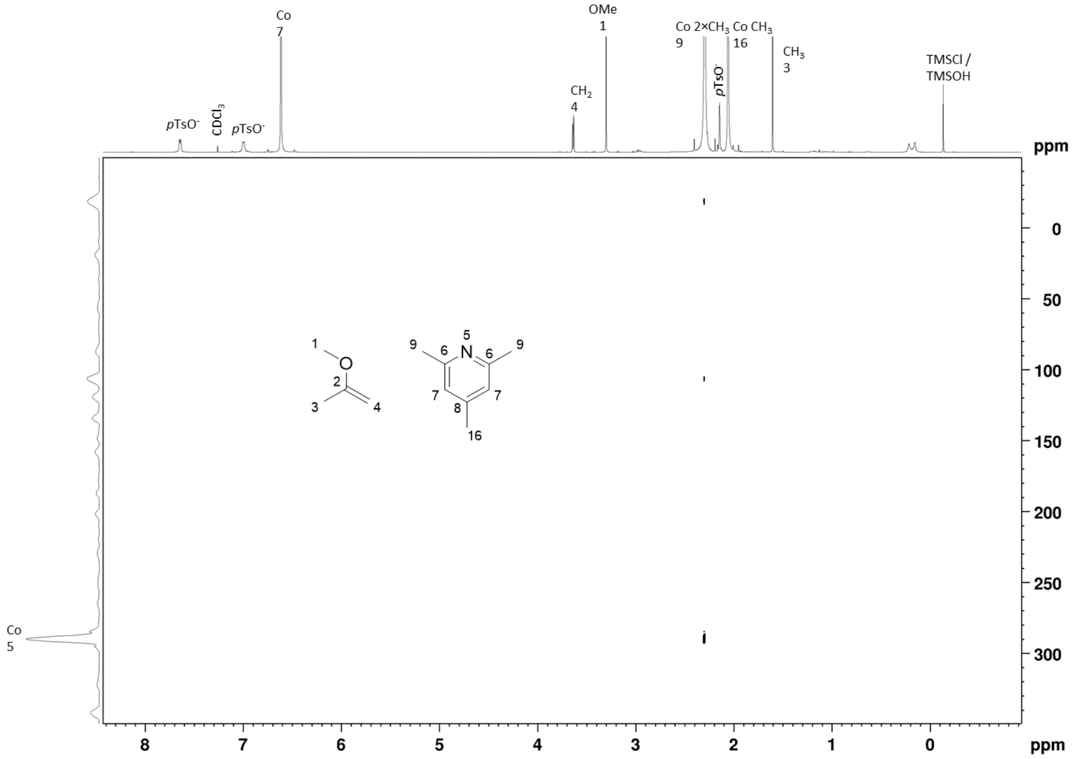

Figure S119. ^{1}H-^{15}N HMBC (600 and 61 MHz, $CDCl_3$) spectrum of the reaction mixture (vi)

Purity documentation for known compounds 2a-d & 10a-d

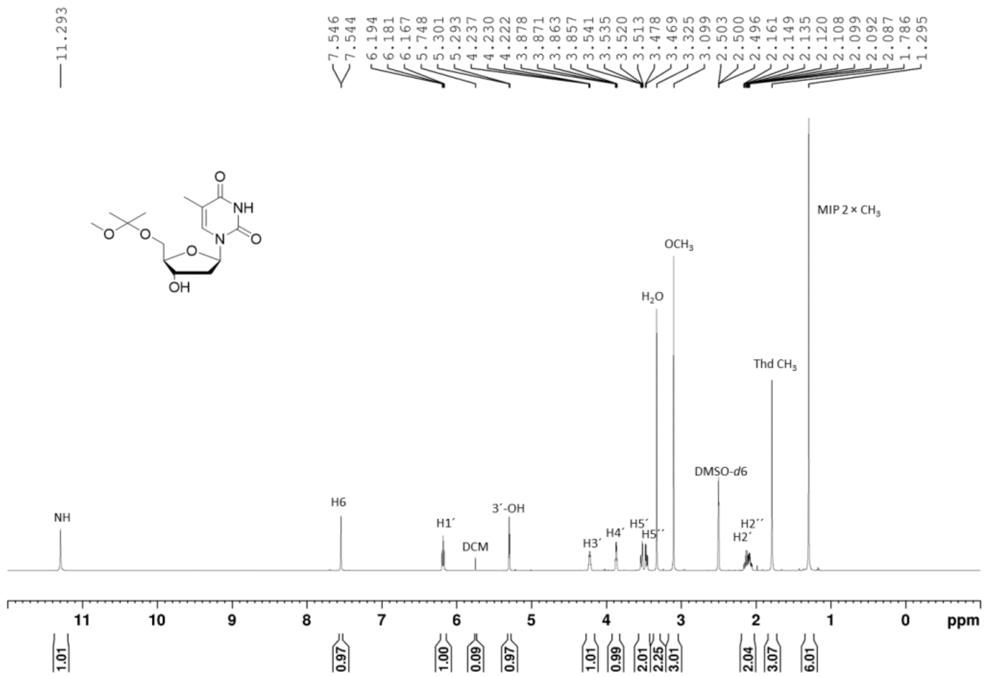

Figure S120. ^{1}H NMR (500 MHz, DMSO-*d*6) spectrum of **2a**

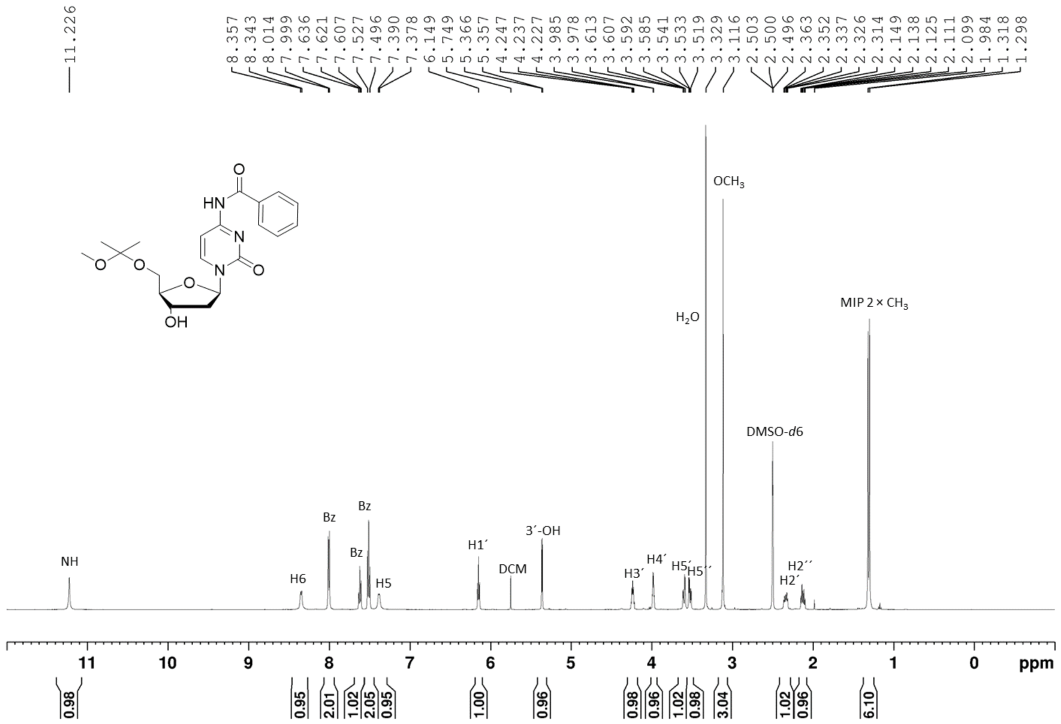

Figure S121. ^{1}H NMR (500 MHz, DMSO-*d*6) spectrum of **2b**

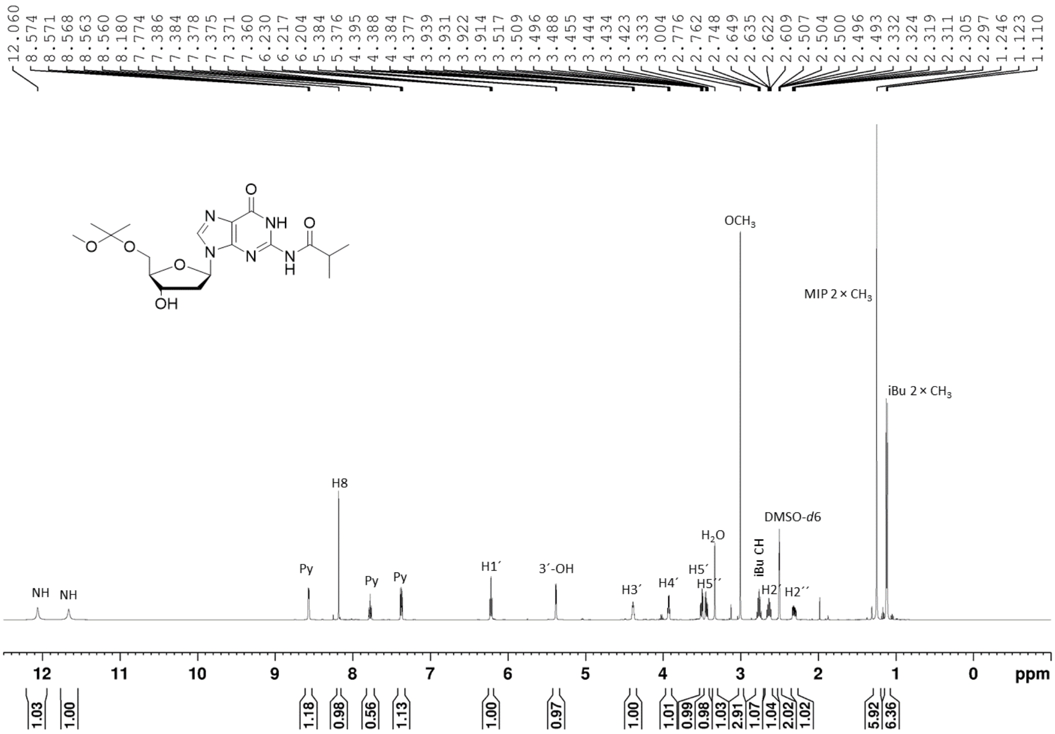

Figure S122. ^{1}H NMR (500 MHz, DMSO-*d*6) spectrum of **2c**

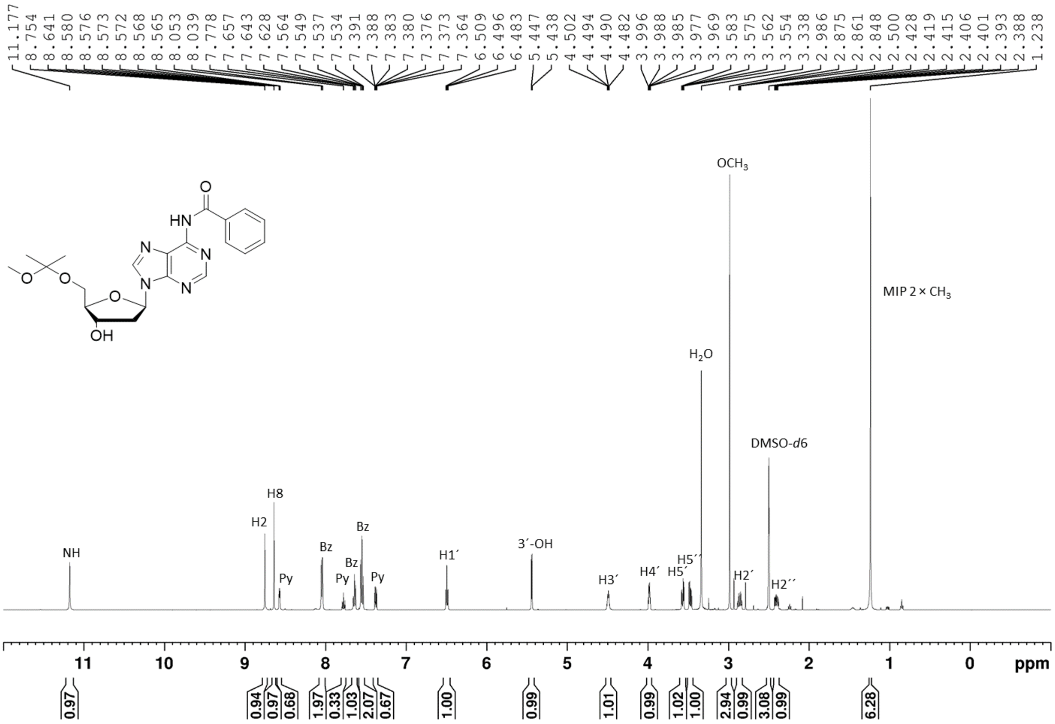

Figure S123. ^{1}H NMR (500 MHz, DMSO-*d*6) spectrum of **2d**

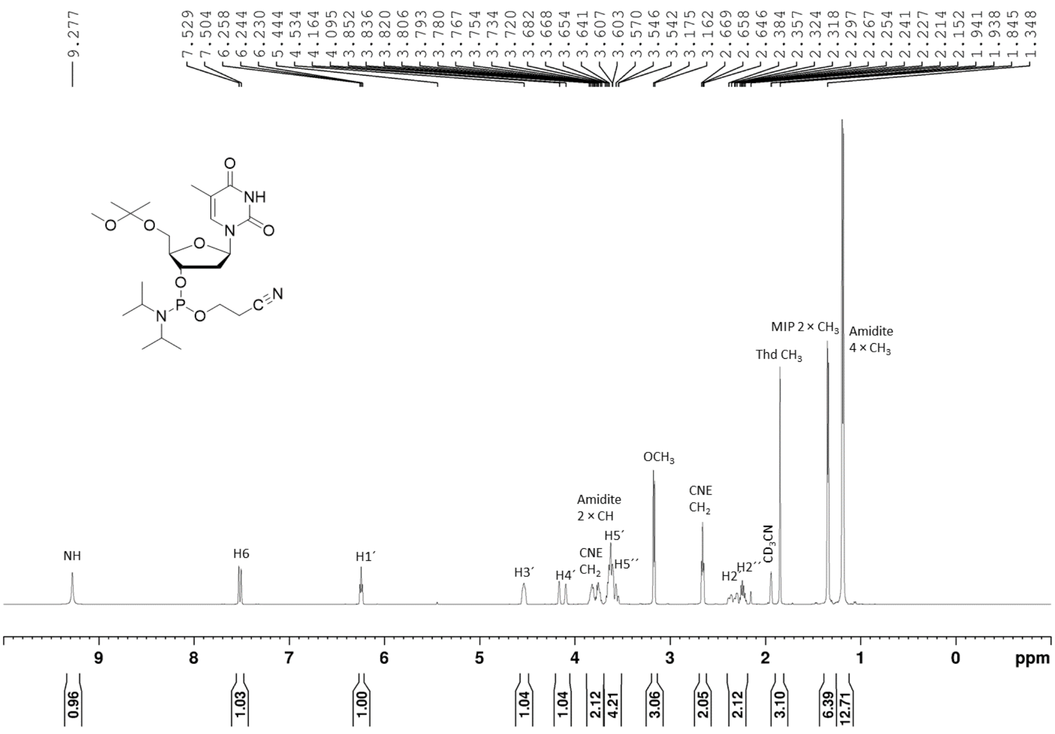

Figure S124. ^{1}H NMR (500 MHz, CD_3CN) spectrum of **10a**

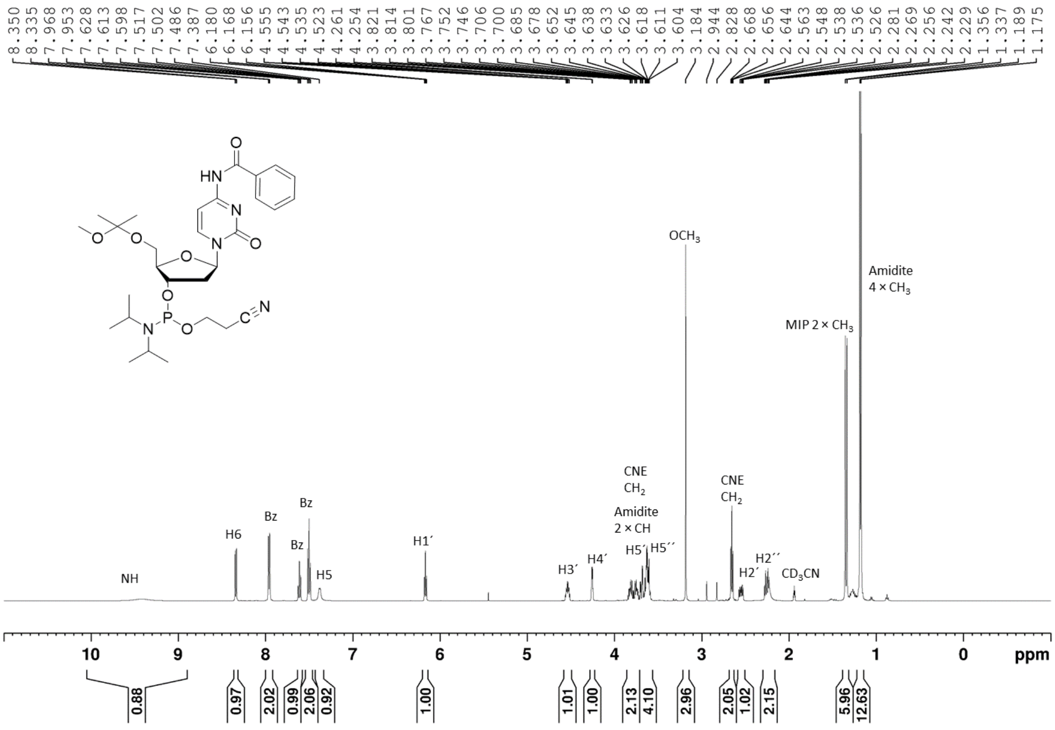

Figure S125. ^{1}H NMR (500 MHz, CD_3CN) spectrum of **10b**

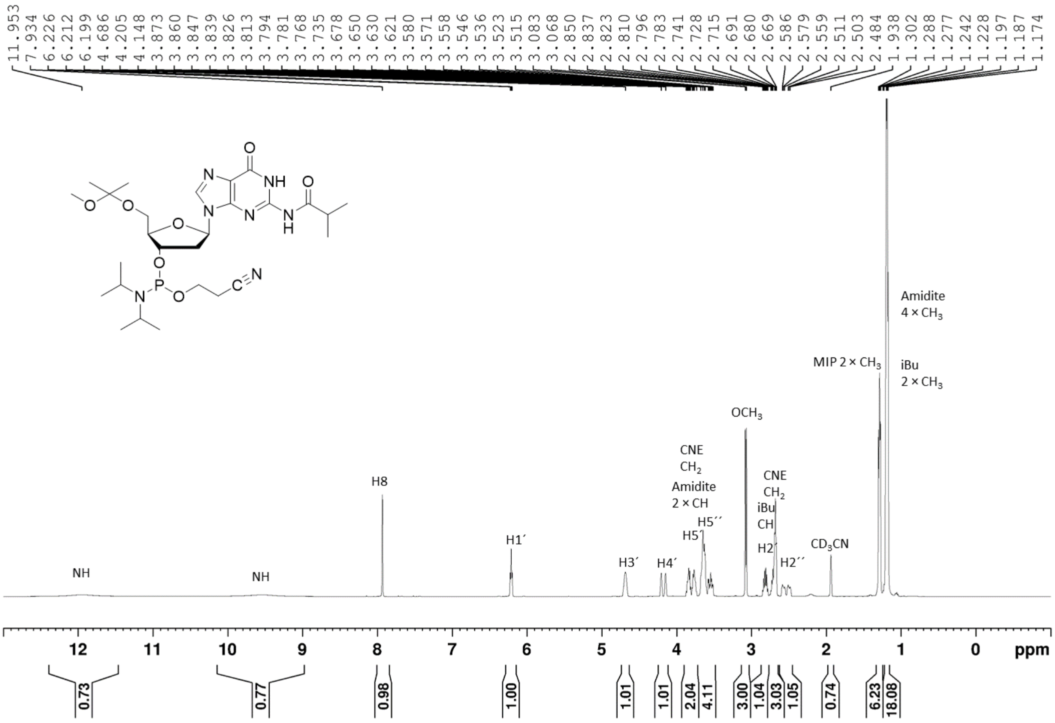

Figure S126. ^{1}H NMR (500 MHz, CD_3CN) spectrum of **10c**

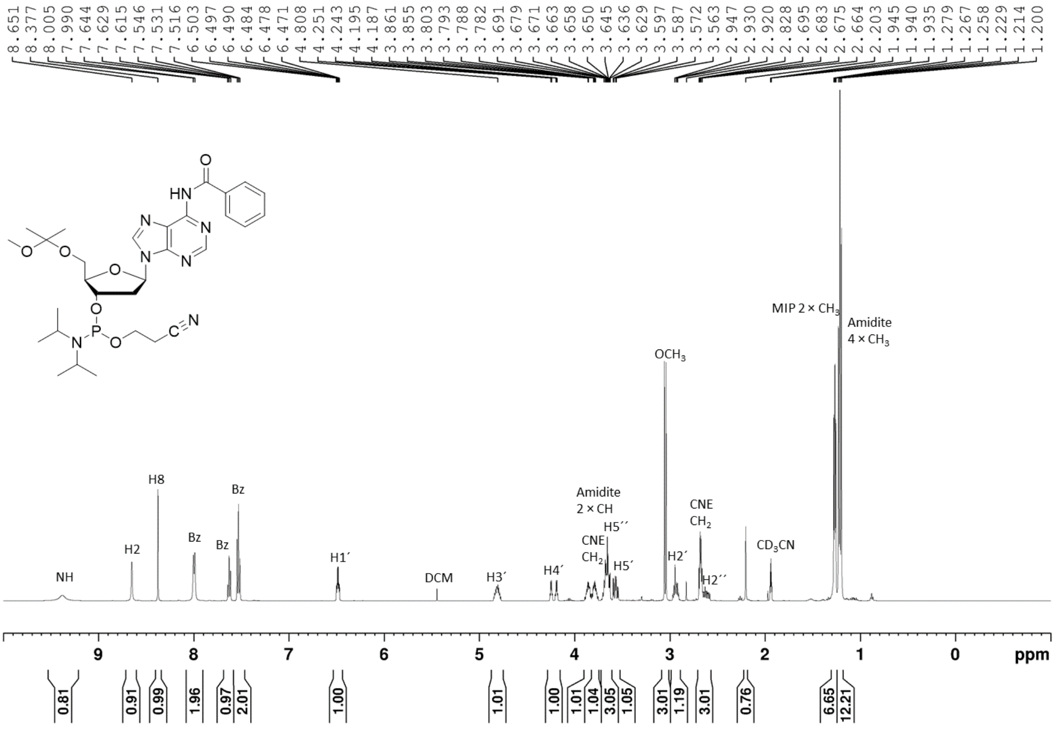

Figure S127. ^{1}H NMR (500 MHz, CD_3CN) spectrum of **10d**

References

(1) Molina, A. G.; Kungurtsev, V.; Virta, P.; Lönnberg, H. Acetylated and Methylated β-Cyclodextrins as Viable Soluble Supports for the Synthesis of Short 2′-Oligodeoxyribo-Nucleotides in Solution. *Molecules*, **2012**, *17*, 12102–12120.

(2) Rosenqvist, P.; Saari, V.; Pajuniemi, E.; Gimenez Molina, A.; Ora, M.; Horvath, A.; Virta, P. Stereo-Controlled Liquid Phase Synthesis of Phosphorothioate Oligonucleotides on a Soluble Support. *J. Org. Chem.* **2023**, *88*, 10156–10163.

(3) Rosenqvist, P.; Saari, V.; Ora, M.; Molina, A. G.; Horvath, A.; Virta, P. Tuning the Solubility of Soluble Support Constructs in Liquid Phase Oligonucleotide Synthesis *J. Org. Chem.* **2024**, *89*, 13005–13015.
